# Multi-omics reveal critical roles of phosphatidylcholine and sphingomyelin in antipsychotic efficacy for schizophrenia

**DOI:** 10.1038/s41392-025-02431-4

**Published:** 2025-10-13

**Authors:** Junyuan Sun, Zhe Lu, Zhewei Kang, Yuyanan Zhang, Yaoyao Sun, Guorui Zhao, Qijing Bo, Wenqiang Li, Zhenghui Yi, Binbin Chen, Yuandong Gong, Zhenhe Zhou, Huiling Wang, Lin Lu, Weihua Yue

**Affiliations:** 1 Peking University Sixth Hospital, Peking University Institute of Mental Health, NHC Key Laboratory of Mental Health (Peking University), National Clinical Research Center for Mental Disorders (Peking University Sixth Hospital), Beijing, China; 2 Beijing Anding Hospital, Capital Medical University, Beijing, China; 3 The Second Affiliated Hospital of Xinxiang Medical College, Xinxiang, China; 4 Shanghai Mental Health Center, Shanghai, China; 5 Xiamen Xianyue Hospital, Xiamen, China; 6 Shandong Mental Health Center, Shandong University, Jinan, China; 7 Wuxi Mental Health Center, Wuxi, China; 8 Renmin Hospital of Wuhan University, Wuhan, China; 9 PKU-IDG/McGovern Institute for Brain Research, Peking University, Beijing, China

**Keywords:** Genomics, Prognostic markers, Therapeutics

## Abstract

Nearly 30% of patients with schizophrenia respond inadequately to current antipsychotics, with unclear markers and mechanisms of antipsychotic efficacy. A total of 208 patients with schizophrenia treated for 6 weeks with oral paliperidone were analyzed through genotyping, mass spectrometry proteomic, and metabolomic profiling to explore underlying markers and mechanisms of antipsychotic efficacy. Machine learning analysis identified 20 proteins and 20 metabolites at baseline predictive of treatment response. Proteomic and metabolomic models achieved a cross-site mean AUC of 0.923 and 0.816, respectively. A multi-omics ensemble model achieved 0.941. GWAS and differential analyses identified 32 loci (*P* < 5 × 10^−5^), 83 proteins, and 31 metabolites associated with efficacy (*P* < 0.05). Trans-omics analysis of these efficacy-related molecules across three omic layers highlighted glycerophospholipid metabolism (*P* = 3.25 × 10^−5^) and sphingolipid metabolism (*P* = 0.039). Key molecules within these pathways exhibited a consistent direction of effect in regulating phosphatidylcholine (PC) and sphingomyelin (SM) metabolism, and higher PC and SM levels were found to correlate with better efficacy. These associations were further genetically validated using polygenic risk scores in two independent cohorts (2281 and 449 patients, respectively). In conclusion, multi-omics modeling is able to accurately identify antipsychotic efficacy, and higher PC and SM levels correlate with better antipsychotic efficacy, suggesting that variations in phospholipid metabolism may underlie the response to antipsychotics.

## Introduction

Schizophrenia (SCZ) is a severe mental disorder with complex mechanisms and a high disability rate, which brings heavy economic cost and health burden.^1,2^ Antipsychotics are the main treatment for SCZ, but individual responses vary significantly, with up to 30% of patients showing poor response to current antipsychotics.^3^ In clinical practice, trial-and-error is common for choosing the optimal antipsychotic treatment, and it is necessary to identify biomarkers related to antipsychotic efficacy. Despite the involvement of various molecular hypotheses, including synaptic neurotransmission,^4^ neuroinflammation,^5,6^ immunity, and neuroendocrine factors,^7^ the molecular mechanisms of antipsychotic treatment response remain unclear, limiting the development of novel targets and therapeutic strategies.

Rapid advances in high-throughput omics technologies have enabled researchers to explore molecular biomarkers associated with SCZ and reveal potential pathological mechanisms. Several studies have identified molecules and pathways associated with antipsychotic efficacy and treatment response through genomics, proteomics, and metabolomics separately. Our prior genome-wide association study (GWAS) has identified genes related to synaptic function, neurotransmitter receptors, and schizophrenia risk that are associated with response to antipsychotics.^8^ Previous proteomic studies identified protein metabolism and the immune system as key biological processes (BPs) for response to olanzapine and risperidone,^9^ and proteins related to complement and coagulation cascades influencing antipsychotic response.^10^ Metabolomic studies revealed baseline phospholipid metabolites such as lysophosphatidylcholine (LPC) and phosphatidylethanolamine (PE) are associated with antipsychotic response.^11^ And changes in post-treatment polyunsaturated fatty acid (PUFA) levels in erythrocyte membranes have also been found to correlate with therapeutic outcomes.^12^

However, these studies were limited to single-omics levels, making it difficult to capture the complex mechanisms underlying antipsychotic efficacy. Multi-omics analysis, especially the trans-omics approach, integrates molecules upstream and downstream of biological pathways, providing a comprehensive overview of BPs.^13^ Additionally, it reduces the noise inherent in single-omics levels, thereby highlighting significant trans-omics pathways relevant to treatment efficacy.^14^ Despite its potential, trans-omics investigations of antipsychotic efficacy in SCZ are still lacking.

Therefore, this study aimed to investigate the mechanisms underlying antipsychotic treatment efficacy using a multi-omics approach. First, we investigated the non-targeted mass spectrometry (MS) proteomic and metabolic differences in plasma between the paliperidone treatment responders (TR) and non-responders (NTR) groups, and developed integrated protein-metabolism models to predict therapeutic responses using machine learning. Second, by incorporating genomic data, we explored the potential gene-protein-metabolite trans-omics pathways. Finally, to validate the MS findings across external populations and different tissues, we applied plasma and brain metabolite polygenic risk scores (PRS) in two independent cohorts. The plasma PRS validation cohort combined two Han Chinese schizophrenia cohorts with a shared genetic background, while the brain PRS validation cohort was from a European schizophrenia cohort.

## Results

### Cohort description and baseline characteristics

**Fig. 1:**
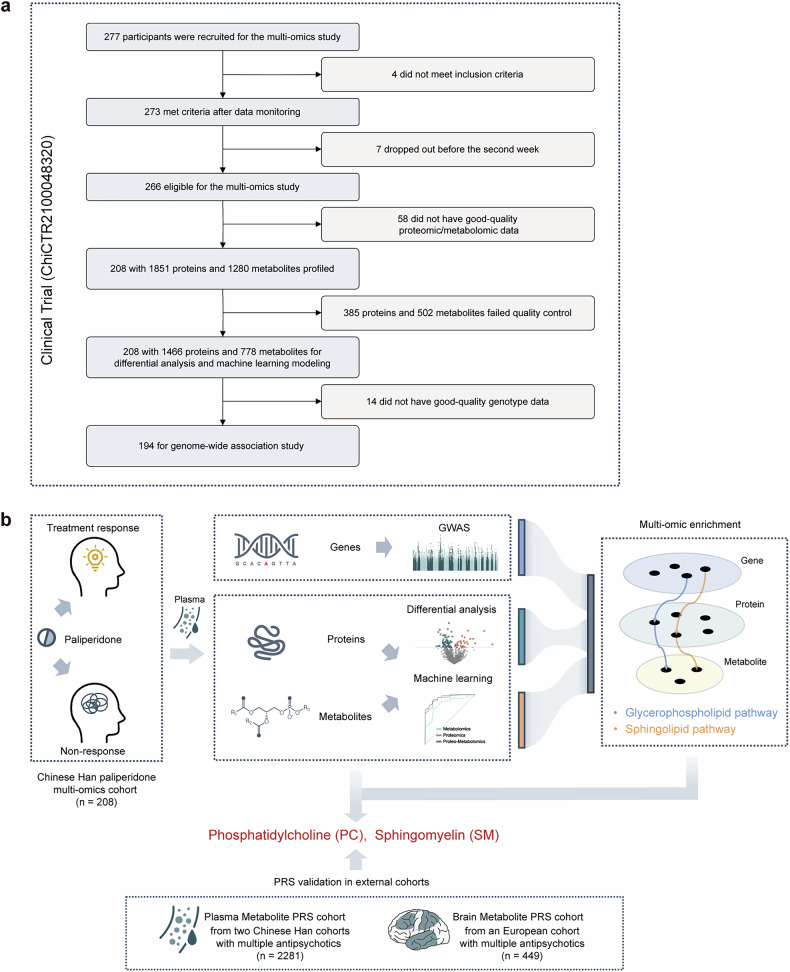
Flow diagram and flowchart. **a** Flow diagram of the multi-omics cohort. **b** Flowchart of the study. First, in the multi-omics cohort, subjects treated with 6-week paliperidone were divided into treatment responder and non-responder groups. Baseline genomic, proteomic, and metabolomic markers associated with antipsychotic efficacy were then explored using GWAS, differential analysis, and machine learning. Multi-omics enrichment was employed to investigate biological pathways affecting the response to antipsychotics. Subsequently, detailed analysis of these pathways identified key molecules related to antipsychotic efficacy. Finally, the association between these key molecules and treatment efficacy was validated in external cohorts by plasma and brain metabolite PRS. GWAS genome-wide association study, PC phosphatidylcholine, PRS polygenic risk score, SM sphingomyelin

To investigate the multi-omics features associated with paliperidone treatment efficacy in patients with schizophrenia, we profiled the multi-omics signatures for the paliperidone cohort. A total of 277 patients were recruited in the paliperidone cohort. After baseline multi-omics data collection and 6 weeks of single paliperidone treatment, 208 patients remained after excluding 4 not meeting inclusion criteria, 7 not completing follow-up, and 58 lacking good quality proteomic or metabolomic data (Fig. 1). In these patients, proteomic profiling using liquid chromatography-tandem mass spectrometry (LC-MS/MS) quantified 1466 proteins, and metabolomic profiling using a triple quadrupole LC-MS/MS system identified 778 metabolites, including 433 lipids, and genomic profiling with the Infinium Asian Screening Array yielded 6919970 quality-controlled single-nucleotide polymorphisms (SNPs) for subsequent analyses. Treatment response was evaluated using the Positive and Negative Syndrome Scale (PANSS). Among the 208 patients, 95 patients with a reduction rate of more than 50% after treatment were assigned to the TR group, and the remaining 113 patients were assigned to the NTR group. The flow diagram and the study design flowchart are shown in Fig. 1 and Supplementary Fig. S1.

To control for potential confounders in subsequent analyses, baseline demographics and clinical characteristics were compared between the two groups. No significant differences were observed (Table 1), supporting the comparability of the two groups.

**Table 1:** Demographic, clinical, and metabolic characteristics of TR and NTR groups in the multi-omics cohort

Variables	TR (*n* = 95)	NTR (*n* = 113)	*χ*^2^ */ U*	*P*
Age (years)	33.81 ± 10.61	32.98 ± 10.03	5560	0.657
Sex (male)	36 (37.89%)	51 (45.13%)	1.111	0.292
Educational level			-	0.266
College and above	34 (35.79%)	29 (25.66%)		
High school	37 (38.95%)	52 (46.02%)		
Middle school	19 (20.00%)	29 (25.66%)		
Primary school and below	5 (5.26%)	3 (2.66%)		
First episode (yes)	29 (30.53%)	28 (24.78%)	0.857	0.355
Family history of mental illness (yes)	18 (18.95%)	27 (23.89%)	0.745	0.388
Smoking history (yes)	10 (10.53%)	18 (15.93%)	1.293	0.255
Drinking history (yes)	7 (7.37%)	10 (8.85%)	0.151	0.698
Disease duration (years)	7.28 ± 6.86	8.09 ± 7.01	4893	0.272
PANSS at baseline	83.66 ± 17.18	78.40 ± 11.53	6178	0.061
Antipsychotics discontinued at baseline			5.448	0.066
Second-generation antipsychotics	10 (10.53%)	16 (14.16%)		
First-generation antipsychotics	4 (4.21%)	14 (12.39%)		
No regular antipsychotics use	81 (85.26%)	83 (73.45%)		
Mood stabilizers use	3 (3.16%)	4 (3.54%)	-	1
Antidepressants use	3 (3.16%)	2 (1.77%)	-	0.662
Benzodiazepines use	31 (32.63%)	38 (33.63%)	0.023	0.879
Metabolic profile at baseline				
BMI	23.17 ± 4.71	23.91 ± 4.53	5970	0.164
Triglyceride (mmol/L)	1.20 ± 0.67	1.29 ± 0.77	5780	0.341
Cholesterol (mmol/L)	4.64 ± 1.65	4.48 ± 0.99	5160	0.632
LDL cholesterol (mmol/L)	2.68 ± 0.73	2.68 ± 0.79	5330	0.931
HDL cholesterol (mmol/L)	1.27 ± 0.36	1.21 ± 0.33	4844	0.226
Fasting blood glucose (mmol/L)	5.01 ± 0.96	4.99 ± 1.01	5100	0.537

Continuous data are presented as means with standard deviations, and categorical data are presented as counts with percentages. *U* indicates the *U* statistic of the Mann–Whitney *U* test

*BMI* body mass index, *HDL* high-density lipoprotein, *LDL* low-density lipoprotein, *NTR* treatment non-responders, *PANSS* Positive and Negative Syndrome Scale, *TR* treatment responders

The *P* values of educational level, mood stabilizer, and antidepressant use were calculated with Fisher’s exact test

Of note, only one participant was enrolled from the Xiangya site. Therefore, data from eight sites (*n* = 207) were used for cross-site validation, while all 208 patients from nine sites were included in the least absolute shrinkage and selection operator (*LASSO*) and differential analysis for comprehensiveness.

### Multi-omics models predict antipsychotic efficacy

**Fig. 2:**
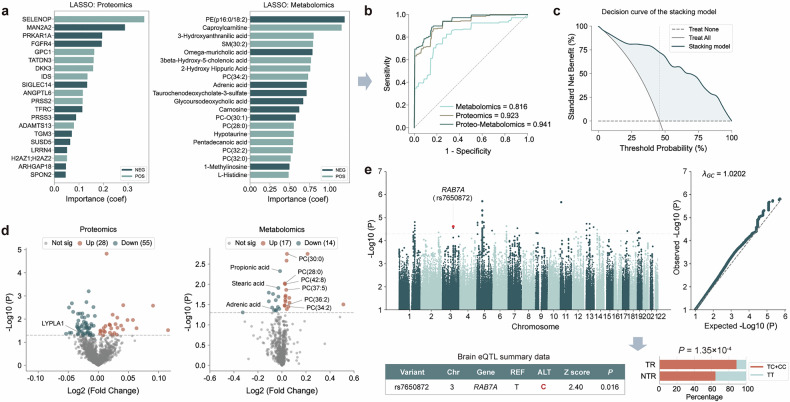
Genomic, proteomic, and metabolomic analyses identified molecules related to antipsychotic efficacy, with multi-omics enrichment highlighting glycerophospholipid and sphingolipid metabolism pathways, including PC and SM as key molecules. **a** *LASSO* regression identified proteins and metabolites associated with treatment efficacy. **b** ROC curve for cross-site validation of proteomic, metabolomic, and stacking models. **c** Decision curve analysis plot of the stacking model. **d** Differentially expressed proteins and metabolites between TR and NTR. **e** Manhattan and Q-Q plots of the GWAS, and *RAB7A* brain expression based on eQTL summary data. GWAS genome-wide association study, *LASSO* least absolute shrinkage and selection operator, NEG negative effect on treatment response, NTR non-responders, PC phosphatidylcholine, PC-O ether-linked phosphatidylcholine, PE phosphatidylethanolamine, POS positive effect on treatment response, SM sphingomyelin, TR treatment responders

To develop predictive models and to identify proteomic and metabolomic features nonlinearly associated with paliperidone efficacy, we first performed feature selection using *LASSO* regression to identify the most informative variables. The top 20 proteins and metabolites were selected based on the largest absolute coefficients from 137 non-zero metabolites and 46 non-zero proteins (Fig. 2a; Supplementary Table S1). This selection yielded the best balance between predictive performance evaluated by cross-validation area under the curve (AUC), and model simplicity (fewer input features), as determined by comparison with models using 5, 10, 15, 20, and 25 features (see Supplementary Fig. S2 for details).

To evaluate the predictive performance of the selected protein features, we built a *Logistic* model using the top 20 proteins. The mean AUC for the *Logistic* model was 0.923 in cross-site validations (Fig. 2b, accuracy = 0.862, sensitivity = 0.834, specificity = 0.870, and balanced accuracy = 0.852). Similarly, the top 20 metabolites were identified in the metabolomic model (Fig. 2a), and the mean AUC reached 0.816 (Fig. 2a, accuracy = 0.760, sensitivity = 0.758, specificity = 0.772, and balanced accuracy = 0.765). The proteomic and metabolomic models were integrated using the stacking approach, with a mean AUC of 0.941 (Fig. 2b, accuracy = 0.853, sensitivity = 0.868, specificity = 0.809, and balanced accuracy = 0.839). Detailed performance is provided in Supplementary Table S2. DeLong’s test revealed significant AUC differences between the proteomic and metabolomic models (*P* = 0.0094), and between the stacking and metabolomic models (*P* = 7.70 × 10^−6^), while no significant difference was observed between the stacking and proteomic models (*P* = 0.13).

The potential clinical net benefit of the proteomic and metabolomic models was assessed using decision curve analysis (DCA), as shown in Supplementary Fig. S3. The standardized net benefit of the stacking model was 0.723 (Fig. 2c). The calibration plot closely follows the diagonal, indicating adequate calibration (Supplementary Fig. S3).

### Multi-omics features for antipsychotic efficacy

To identify molecular differences between TR and NTR groups, we performed differential analysis for proteomic and metabolomic data and GWAS for genomic data. The TR group showed 28 upregulated and 55 downregulated differentially expressed proteins (DEPs) compared to the NTR group (*P* < 0.05, Fig. 2d and Supplementary Table S3). 17 upregulated and 14 downregulated differentially expressed metabolites were identified (*P* < 0.05, Fig. 2d and Supplementary Table S4).

Totally, 194 patients had quality-controlled genotype data. Figure 2e shows the Manhattan and Q-Q plots of the GWAS for antipsychotic efficacy. There were 32 SNPs, physically mapped to 32 genes, at a suggestive level (*P* < 5 × 10^−5^) after clumping (Supplementary Table S5).

### Trans-omics analysis highlights glycerophospholipid and sphingolipid metabolism pathways involved in antipsychotic efficacy

To further explore the potential molecules related to antipsychotic-specific treatment response, we performed a trans-omics analysis of genomics, proteomics, and metabolomics based on the organism-specific data from the Kyoto Encyclopedia of Genes and Genomes (KEGG) database (metabolite-protein interaction data from all KEGG reactions).

We constructed a Gene-Protein-Metabolite Interaction (GPMI) network based on shared nodes from protein–protein interactions (PPI) and KEGG data using distinguishing genes, proteins, and metabolites identified by differential analysis and machine learning approaches. Combining the input of 32 genes, 91 proteins, and 44 metabolites, we unveiled a complex GPMI network of 1896 nodes and 2709 edges. The final network was trimmed through Prize-collecting Steiner Forest (PCSF).

**Fig. 3:**
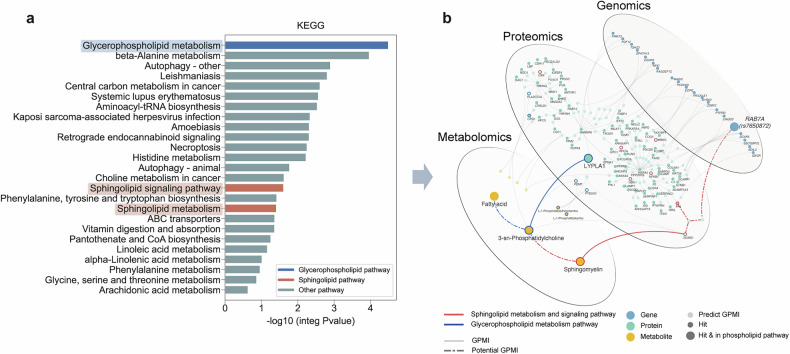
Trans-omics analysis highlighted glycerophospholipid and sphingolipid metabolism pathways involved in antipsychotic efficacy. **a** KEGG analysis based on the GPMI network. Blue bars and borders indicate glycerophospholipid-related pathways, and red bars and borders indicate sphingolipid pathways. **b** GPMI network plot. Blue circles represent genes, green represents proteins, and yellow represents metabolites. Solid small circles indicate the genes, proteins, and metabolites associated with treatment response identified in this study. Solid large circles indicate the genes, proteins, and metabolites associated with treatment response identified in this study and also in the phospholipid pathway. Translucent small circles indicate genes, proteins, and metabolites predicted based on Protein–protein interactions and KEGG databases. Solid connections indicate those generated by the OmicsNet database. Dashed connections indicate those generated by previous literature. Red borders and connections indicate Sphingolipid metabolism and signaling pathways. Blue borders and connections indicate Glycerophospholipid metabolism pathways. GPMI Gene-Protein-Metabolite Interaction, KEGG Kyoto Encyclopedia of Genes and Genomes

KEGG analysis (Gene-Protein-Metabolite) on the trimmed GPMI network identified 19 significantly overexpressed pathways (Fig. 3a, b). These pathways were primarily classified into four biological functional categories: phospholipid metabolism, amino acid metabolism, autophagy and cell death, and immune and infectious disease–related processes. Among these, phospholipid-related pathways, including glycerophospholipid metabolism (*P* = 3.25 × 10^*−*^^5^)*,* sphingolipid metabolism (*P* = 0.039)*,* and signaling (*P* = 0.025)*,* were highlighted. Glycerophospholipid metabolism was the most significantly enriched pathway, and phospholipids are potentially biologically relevant to membrane dynamics and neurotransmission.^15–17^ Therefore, we focused on phospholipid-related processes in this study, although other pathways were also significantly enriched. Full enrichment results are provided in Supplementary Table S6.

Gene Ontology (GO) analyses (Gene-Protein) highlighted 50 overexpressed BP terms linked to cellular regulation and metabolic processes, 37 overexpressed cellular component terms associated with intracellular vesicles and transport structures, and 43 overexpressed Molecular Function (MF) terms associated with enzymatic and inhibitor activities (all *P* < 0.05, detailed in Supplementary Tables S7–S9).

Based on the results of KEGG and GO enrichment, we focused on the differences in biomolecules related to glycerophospholipid and sphingolipid metabolism (Fig. 3b). Key molecules within these pathways exhibited a consistent direction of effect in regulating PC and SM metabolism, and higher PC and SM levels were found to correlate with better antipsychotic efficacy. For sphingolipid metabolism, we found lower *RAB7A* expression in the brain among the NTR compared to the TR group, based on brain expression quantitative trait loci (eQTL) summary data and proportion of rs7650872 effect allele (*P* = 1.35 × 10^−4^, Fig. 2e). And lower SM levels were associated with a poorer response (*β*_*LASSO*_ = 0.79, Fig. 2a). In glycerophospholipid metabolism, the NTR group had higher LYPLA1 and fatty acid (stearic acid, adrenic acid, propionic acid and pentadecanoic acid) levels, and lower PC levels compared to the TR group (all *P* < 0.05, Fig. 2a, d).

Sensitivity analyses by first-episode status showed relatively consistent associations for PC and SM: higher PC and SM levels were correlated with better treatment efficacy among first-episode patients, whereas in the recurrent group, the association for SM did not remain, but the association for PC persisted (see Supplementary Table S10).

### Proteomic and metabolomic changes after treatment

To evaluate longitudinal changes following treatment, we analyzed post-treatment changes in significant proteins and metabolites at baseline (Supplementary Fig. S4). For the glycerophospholipid pathway, LYPLA1 levels decreased after a 6-week treatment in the TR group (*P* = 0.043), with no significant changes in the NTR group. PC (34:2) levels increased post-treatment in the TR group (*P* = 0.031), while other baseline PCs showed no significant changes in either group. Additionally, most baseline fatty acids remained unchanged in both groups, except for a significant increase in adrenic acid in the TR group (*P* = 0.001). For the sphingolipid pathway, no significant changes in SM (30:2) were found in either the TR or NTR groups after treatment.

### Metabolite polygenic risk score validates the association between PC and SM with antipsychotic efficacy

**Fig. 4:**
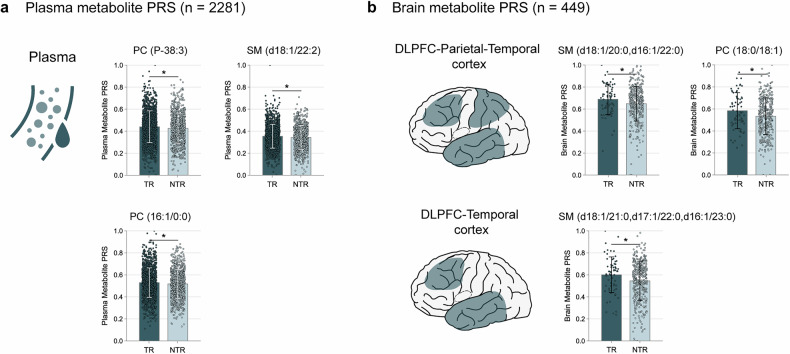
Genetic validation of PC and SM using plasma and brain metabolite PRS in independent datasets. **a** Plasma phospholipids PRS between TR and NTR. Data are shown as mean ± SD (*n* = 2281). **b** Brain phospholipids PRS between TR and NTR. Data are shown as mean ± SD (*n* = 449). Brain phospholipids PRS for DLPFC-Parietal-Temporal and DLPFC-Temporal cortices are shown separately. DLPFC dorsolateral prefrontal cortex, PC phosphatidylcholine, PRS polygenic risk score, SM sphingomyelin. *P < 0.05

To validate the MS findings, we calculated plasma and brain metabolite PRS in two independent cohorts. The plasma PRS validation cohort includes 2281 patients with SCZ from two Han Chinese schizophrenia antipsychotic treatment cohorts. Consistent with MS results in the paliperidone cohort, significantly higher PRSs of plasma PC and SM were found in the validation TR group compared to the NTR group (Fig. 4a and Supplementary Table S11).

The brain PRS validation cohort includes 449 European patients with SCZ from the Clinical Antipsychotic Trials of Intervention Effectiveness in Schizophrenia (CATIE) cohort. Consistent with plasma results, the validation TR group showed significantly higher PRSs for PC and SM in the dorsolateral prefrontal cortex (DLPFC), parietal, and temporal lobes compared to the NTR group (Fig. 4b and Supplementary Table S12).

**Fig. 5:**
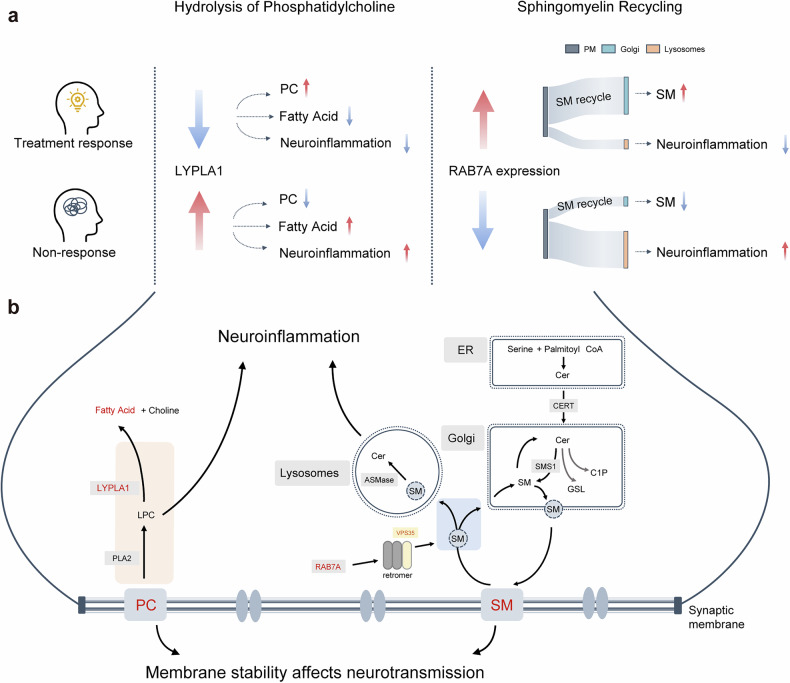
Hypothesized mechanisms for differential phospholipid metabolism and response to antipsychotics. **a** Potential difference in the hydrolysis of phosphatidylcholine and sphingomyelin recycling between treatment responders and non-responders. Red arrows indicate increases, blue arrows indicate decreases. Large flow in the Sankey diagram represents increased recycling, and small flow indicates decreased recycling. **b** Hypothetical molecular mechanism of phosphatidylcholine and sphingomyelin metabolism differences with varied antipsychotic responses. For non-responders, the upregulation of phosphatidylcholine hydrolysis and the decrease in sphingomyelin recycling may lead to potential neuroinflammation and impaired neurotransmission, potentially related to poor antipsychotic response. And treatment responders change in reverse. Red text indicates genes, proteins, and metabolites associated with treatment response identified in this study. Red background indicates the critical steps in phosphatidylcholine hydrolysis, and blue background indicates critical steps in sphingomyelin recycling. ASMase acid sphingomyelinase, C1P ceramide 1-phosphate, Cer ceramide, CERT ceramide transfer protein, ER endoplasmic reticulum, Golgi golgi apparatus, GSL glycosphingolipid, LPC lysophosphatidylcholine, PC phosphatidylcholine, PM plasma membrane, SM sphingomyelin

The results across the three cohorts support the hypothesized mechanisms underlying the antipsychotic treatment response shown in Fig. 5.

## Discussion

This study integrated the genomic, proteomic, and metabolomic features that affect the treatment response of antipsychotics. We highlighted the phospholipid metabolism pathways (phosphatidylcholine and sphingomyelin) as key biological mechanisms potentially driving the efficacy of antipsychotics, through integrating differential analysis and machine learning. Machine learning models based on significant proteins and metabolites demonstrated good distinguishing performance in cross-site validation (average AUC: metabolites 0.816, proteins 0.923, integrated model 0.941) and good clinical net benefit of around 70%, suggesting these models serve as potential tools for guiding clinical use of antipsychotics. Furthermore, this study highlights the potential efficacy of the ensemble approach, as the multi-omics stacking model achieved the highest AUC, although its advantage over the proteomic model did not reach statistical significance.

Due to the distinct heterogeneity and complexity of antipsychotic response, researchers have been searching for its biomarkers for decades. Our study is based on the assumption that SCZ patients responding to antipsychotics have a specific biological status. Integrated multi-omics enrichment analysis combining differential analysis and machine learning in this study has revealed a potential membrane phospholipid mechanism related to the treatment response to antipsychotics. Specifically, the TR group exhibited significantly higher levels of phosphatidylcholine and sphingomyelin compared to the NTR group. Significant changes in genes and proteins associated with phospholipid metabolism and signaling also suggest the metabolomic changes in TR may be due to reduced metabolism of phosphatidylcholine and enhanced recycling of sphingomyelin. These findings align with the potential pathology of schizophrenia demonstrated in previous studies, i.e., schizophrenia may be related to abnormalities in membrane phospholipid metabolism.^18–20^

We identified higher baseline LYPLA1 levels in the NTR group, alongside decreased PCs and increased fatty acids. LYPLA1 is an enzyme involved in the LPC decomposition within the PC metabolism pathway. These findings suggest an upregulation of the PC metabolism pathway in the NTR group (see Hydrolysis of Phosphatidylcholine in Fig. 5). These molecular changes may contribute to poor antipsychotic response, as supported by previous findings. A lipidomic study found baseline LPC, PE, and diacylglycerols were associated with antipsychotic treatment response.^11^ In addition, a proton magnetic resonance spectroscopy meta-analysis revealed excessive PC metabolism in the anterior cingulate cortex of the treatment-resistant schizophrenia (TRS) group, evidenced by elevated levels of PC products (phosphocholine and glycerophosphocholine) compared to the non-TRS group and healthy controls.^21^ Patients with schizophrenia in previous case-control studies also exhibited similar patterns to the NTR group in this study. A postmortem brain tissue study found significantly lower phosphatidylcholine levels in patients with schizophrenia compared to controls.^20^ And higher fatty acid levels,^22^ particularly stearic acid,^11^ have also been observed in patients with schizophrenia, which may be related to abnormal phospholipid metabolism.

Beyond disturbances in PC metabolism, dysregulation of sphingomyelin metabolism represents another potential mechanism underlying poor treatment response. We found the potential low expression of the *RAB7A* gene in the brain and reduced baseline sphingomyelin in the NTR group. RAB7 dissolves α-synuclein aggregates, thus regulating SM reuptake, an essential process for meeting membrane phospholipid needs during rapid membrane expansion.^23,24^ The loss of RAB7 inhibition of α-synuclein overexpression, by inhibiting retromer function, could lead to more endosomes being routed towards the lysosomal acidic degradation pathway,^23^ may inhibit the reuptake and recycling of membrane SM, thereby reducing membrane sphingomyelin levels^24^ (see Sphingomyelin Recycling in Fig. 5). Decreased SM may thus destabilize membranes, affect lipid raft functions, and impair neural transmission.^16,17,25^ Additionally, enhanced lysosomal pathways could elevate acid sphingomyelinase (ASM) activity and ceramide production, exacerbating neuroinflammation, a known pathogenic contributor to schizophrenia.^26^

These disturbances in PC and SM metabolism not only compromise membrane stability and induce neuroinflammation but may further affect neurotransmitter systems such as dopaminergic, glutamatergic, and serotonergic pathways. These pathways underlie the pathogenesis of schizophrenia and are targeted by antipsychotic therapies. Specifically, increased hydrolysis of PC elevates arachidonic acid levels, altering membrane fatty acid composition and receptor dynamics, potentially affecting neurotransmitter signaling.^15,27^ Reduced sphingolipids disrupt lipid rafts, affecting the function of G protein-coupled serotonin receptors and potentially influencing downstream signaling.^17^ Alterations in sphingolipid metabolism may also alter α7 nicotinic acetylcholine receptor kinetics, affecting glutamatergic and GABAergic neurons, and subsequently affecting dopamine release and neurotransmission.^28^ Changes in membrane sphingomyelin/ceramide balance may also affect neurotransmitter reuptake.^29^ Furthermore, as an inflammatory product of PC, LPC may disrupt neurotransmitter homeostasis by activating glial cells and impairing synaptic function.^30–32^ Phospholipid metabolites, such as PUFAs, also modulate dopamine, serotonin, and glutamate signaling.^33^ Notably, supplementation with Omega-3 PUFAs has been reported to regulate membrane phospholipid composition, thereby modulating neurotransmitter systems and inflammation, and potentially alleviating psychotic symptoms.^33,34^ Therefore, nutritional or pharmacological interventions targeting phospholipid metabolism may offer new therapeutic options for patients with poor response to antipsychotics.

Furthermore, associations between higher phospholipids and better antipsychotic efficacy observed in the multi-omics cohort were independently validated at the polygenic level in the plasma and brain PRS validation cohorts. These findings suggest that differences in phospholipid-related antipsychotic efficacy may be more genetically determined. Notably, the relatively higher PC and SM levels in the TR group compared to the NTR group remained unaffected by antipsychotic treatment (remained higher post-treatment), indicating that PC and SM levels may reflect stable genetic or physiological traits that influence long-term treatment response. This was supported by PRS-based validation in two validation datasets. Finally, the sensitivity analyses revealed that only in the recurrent group, the association between plasma SM and treatment response was not maintained, which may be due to long-term use of antipsychotics and their metabolic side effects affecting SM metabolism. Previous studies have found that antipsychotic use may lower SM levels.^35,36^ Additionally, weight gain has also been associated with reduced SM levels following antipsychotic use in a previous study.^36^ The mechanism of sphingolipid disruption by antipsychotics remains unclear, but may involve facilitating the conversion of sphingolipids to other metabolites or inhibiting their synthesis in the endoplasmic reticulum.^37^ Therefore, long-term use of antipsychotics in recurrent patients may affect SM metabolism, weakening the association between baseline plasma SM levels and antipsychotic efficacy.

The multi-omics cohort is based on a prospective, multicenter clinical trial of a single antipsychotic, with the benefit of a large sample size and three types of omics data compared to previous studies. This study also has several limitations. First, we applied a more lenient threshold (*P* values for proteomic and metabolomic differential analyses were not corrected for multiple comparisons) in this study. This method was chosen because, as a complex polygenic disorder, schizophrenia shows subtler molecular differences than somatic diseases. Moreover, all subjects in this study were patients with schizophrenia, and molecular differences between the TR and NTR groups were also less significant than in typical schizophrenia case-control studies. Therefore, to incorporate additional potential molecules that may correlate to treatment efficacy, a more lenient threshold was employed. Correspondingly, we performed a trans-omics integration enrichment analysis, focusing on upstream and downstream biological pathways across multi-omics layers. Support from multiple omics of the same biological pathway is likely to help reduce the risk of high false positive rates in individual omics analyses, thereby enabling a more reliable exploration of pathways potentially associated with treatment efficacy. Second, although the use of validation cohorts with different samples and genotyping methods may have affected PRS calculation. We applied consistent quality control procedures and the imputation database across cohorts to minimize this impact. Another potential limitation is that first-episode and recurrent patients with acute episodes were combined in the multi-omics analysis. Although sensitivity analyses showed relatively consistent results, differences in underlying biology and treatment response may exist due to the chronic course. Future studies with larger sample sizes should analyze these subgroups separately to explore potential biological differences in treatment efficacy. Additionally, our omics data from plasma may not accurately reflect the proteomic and metabolomic status within the brain, cerebrospinal fluid, or brain tissue.^38,39^ Future studies should utilize these tissues to provide more precise insights, and incorporating single-cell data may also enhance predictive performance and deepen mechanism insights.

In conclusion, multi-omics analysis in patients treated with paliperidone demonstrated that higher PC and SM were associated with better antipsychotic efficacy, and targeted fatty acid supplementation or pharmacological interventions in phospholipid metabolism may offer new treatment strategies, which require further investigation.

## Materials and methods

### Ethic approval statements

This study complied with the Declaration of Helsinki and the protocol of paliperidone cohort was approved by the Clinical Research Ethics Committees at Peking University Sixth Hospital (2021-LunShen-No.40), Beijing Anding Hospital (2021-KeYan-No.106), the Second Affiliated Hospital of Xinxiang Medical College (XYEFYLL-KeYan-2021-47), Shanghai Mental Health Center (2022-40), Xiamen Xianyue Hospital (2021-LL-005-01), Shandong Mental Health Center (2022-LunShen-No.44), Wuxi Mental Health Center (WXMHCIRB2021LLky141), Renmin Hospital of Wuhan University and the Second Xiangya Hospital (WDRY2021-K163). The validation cohort protocols were approved by the Ethics Committees at each center, as reported previously. Written informed consent was obtained prior to the study procedures.

### Study design and participants

This study was a secondary analysis of data from multiple prospective clinical cohorts, including a primary paliperidone cohort and three independent validation cohorts involving various antipsychotics.

The main cohort (multi-omics cohort) was from a prospective, multicenter clinical trial conducted from 2021 to 2023 (the Clinical registration number: ChiCTR2100048320), mainly designed to explore the relationship between previously identified candidate genetic loci and the efficacy and tolerability of paliperidone. A total of 277 patients with schizophrenia were recruited from nine clinical centers to receive a 6-week course of oral paliperidone (Invega®, Xian Janssen Pharmaceutical Ltd, China). Patients were included if they were diagnosed with schizophrenia based on the Mini International Neuropsychiatric Interview according to the Diagnostic and Statistical Manual of Mental Disorders (fourth edition), were 18–45 years old, of Han Chinese ancestry with both biological parents also Han Chinese, and included both genders. They were required to be either first-episode unmedicated or in a chronic course with an acute episode, to have a total PANSS score ≥60 with scores ≥4 on at least three of the seven positive symptoms, and to provide written informed consent from themselves or their legal guardian. Patients were excluded if they were pregnant, breastfeeding, or planning pregnancy; had contraindications to paliperidone; had unstable physical diseases; or had cardiac conditions, including prolonged QTc, decompensated congestive heart failure, or complete left bundle branch block. Clinical centers, the detailed inclusion and exclusion criteria, and dosing regimen of the study are provided in the Supplementary Methods.

The plasma PRS validation cohort was from two previously collected Han Chinese schizophrenia cohorts (ChiCTR-TRC-10000934 and ChiCTR-RNC-09000522), primarily designed to investigate the relationship between the common variants and the efficacy of acute-stage antipsychotics treatment and the side effects of multiple antipsychotics. These cohorts were merged as the first validation dataset, totaling 2281 participants with complete genotype and outcome data.

The brain PRS validation cohort was from the European CATIE cohort, primarily designed to evaluate antipsychotic effectiveness in patients with schizophrenia. This cohort served as the second validation dataset, totaling 449 participants with complete genotype and outcome data (approved by NIMH REPOSITORY & GENOMICS RESOURCE, Requested ID: 63084551a4921; analysis was completed before January 1, 2025; in accordance with NIH policy, all NRGR data were destroyed before the termination date, and written certification was provided as required).

The detailed information for the validation cohorts is described in the Supplementary Methods and previous articles.^40^

### Multi-omics cohort intervention regimen

After enrollment in the multi-omics cohort, participants were required to taper and discontinue any previous psychiatric medications within the first week. Simultaneously, oral paliperidone extended-release (ER) was initiated. The dosage of paliperidone reached at least the minimum dose within the first week, and was adjusted to a stable level (between 3 and 12 mg per day) within the first 2 weeks. No further dose adjustments were permitted thereafter.

### Treatment response

PANSS was evaluated by psychiatrists who received standardized consistency training. PANSS reduction rate was used as treatment response and was calculated using the following formula:

$${\text{PANSS}}\,{\text{reduction}}\,{\text{rate}}=\frac{PANSS\,baseline\,scores-PANSS\,endpoint\,scores}{PANSS\,baseline\,scores-30}\times 100$$

In the multi-omics cohort, TR was defined as greater than 50% PANSS reduction rate after 6-week treatment. In the plasma PRS validation cohort, which included two Han Chinese schizophrenia cohorts, TR was defined as greater than 50% PANSS reduction rate after 6-week treatment in the first cohort and after 8 weeks in the second cohort. In the brain PRS validation cohort, TR was defined as greater than 50% PANSS reduction rate after 12-week treatment.

### Proteomic and metabolomic profiling

In the multi-omics cohort, proteomic profiling was performed using LC-MS/MS using an EASY-nLC 1200 HPLC (Thermo Scientific) coupled with a Q Exactive HF-X (Thermo Scientific) mass spectrometer. Metabolomic profiling was conducted with an AB SCIEX 5500+ triple quadrupole LC-MS/MS system, enabling absolute quantification of 1800 metabolites (600 endogenous metabolites and 1200 lipids). Detailed proteomic and metabolomic profiling methods and processing are provided in Supplementary Methods.

### Genomic quality control and genome-wide association study

In the multi-omics cohort, samples were genotyped with Infinium Asian Screening Array (Illumina, San Diego, CA, USA). Detailed descriptions for genotyping, quality control, and imputation are provided in the Supplementary Methods. GWAS analysis was conducted using PLINK 1.9 with *Logistic* regression to identify treatment response-related SNPs between TR and NTR. Sex, age, and the first five principal components of population structure (PCA) were included as covariates. Due to limited sample sizes and low effect sizes, treatment response-related SNPs were defined as Linkage Disequilibrium (LD)-independent SNPs with suggestive associations (*P* < 5 × 10^−5^).^41^ We investigated the potential expression of target genes physically mapped in the brain, based on eQTL summary data from human brains^42^ and the proportion of effect alleles across groups.

For the validation cohorts, details of genotyping, quality control, and imputation are provided in the Supplementary Methods.

### Trans-omics analysis

To address the limited comprehensiveness and the lack of information on interactions across multiple omic layers, trans-omics has been proposed to reconstruct molecular networks using prior knowledge from public molecular interaction databases.^43^ The trans-omics network inherently provides causality relationships at a molecular level, facilitating the interpretation of complex BPs.^13^ Accordingly, in this study, we prioritized functional pathways enriched with molecules that were real hits and showed consistent signals across multiple omics layers, to better capture underlying biological mechanisms. These pathways and their key molecules were considered to be associated with treatment response.

An integrated multi-omics enrichment analysis was performed using OmicsNet 2.0 (version released in January 2022; https://www.omicsnet.ca).^44^ OmicsNet combined statistically significant differences with nonlinear machine learning features (consider molecular interactions), potentially enhancing the effectiveness of enrichment analysis.

Specifically, three omics features (genomics, proteomics, and metabolomics) were input into OmicNet and constructed into a GPMI network (trans-omics network) based on shared nodes using PPI and metabolic databases (InnateDB and KEGG in OmicNet).^44^ To reduce noise and enhance interpretability for trans-omics networks, PCSF (a graph-based filtering approach^45^) in OmicNet was applied to eliminate redundant information and focus on core nodes. Finally, we identified the important biological pathways among the GPMI network through built-in KEGG and GO analysis in the OmicNet, and further narrowed our focus to key molecules within these pathways with significant enrichment.

### Metabolite polygenic risk score

We calculated the plasma metabolite PRSs for the plasma PRS validation cohort based on Metabolic GWAS summary statistics from the jMorp project (https://jmorp.megabank.tohoku.ac.jp/gwas-studies, Japan populations), including a total of 252 plasma metabolites (43 in TGA000003 and 206 in TGA000005). The *PRS C*+*T* method using PLINK 1.9 selects SNPs by LD clumping and *P* value thresholding. LD clumping (*r*^2^ < 0.99 in 250 kb window) used 1000 Genomes Project East Asian samples as LD reference. Genotype data from the plasma PRS validation cohort served as the target dataset for PRS calculation, with quality control applied. Considering multiple metabolite GWAS, the metabolite PRS threshold was uniformly set to a more stringent *P* < 1 × 10^−4^ (close to 0.05/252). Prior to differential analysis, the PRSs were normalized to a range of 0–1.

In addition, we calculated the brain metabolite PRSs for the brain PRS validation cohort based on Brain Metabolic GWAS summary statistics from the GWAS catalog (https://www.ebi.ac.uk/gwas/, accession numbers GCST90317902–GCST90319303, European populations).^46^ To further validate the plasma findings, we analyzed only 66 brain phosphatidylcholine (PC) and sphingomyelin (SM) PRS, including 35 PCs (12 from cerebrospinal fluid (CSF), 23 from DLPFC-Parietal-Temporal lobe), and 31 SMs (9 from CSF, 22 from DLPFC-Parietal-Temporal lobe). 1000 Genomes Project European samples were used as the LD reference; other methods were identical to those in plasma metabolite analysis.

### Statistical analysis

For all samples, descriptive statistics were provided using mean (SD) and percentages. For continuous variables, the *t*-test/*Mann–Whitney* *U* test was used as appropriate to examine differences, and the *chi-square* test/*Fisher’s exact* test was used as appropriate for differences in categorical variables. The proteomic and metabolomic changes after antipsychotic treatment in the multi-omics cohort were analyzed using paired samples test (*Wilcoxon signed rank* test or *t*-test) as appropriate.

For the multi-omics cohort, variable selection using *LASSO Logistic* regression identified key proteins and metabolites distinguishing TR from NTR groups. *LASSO* was applied using varying numbers of top-ranked features (5, 10, 15, 20, and 25), and the number of features was selected based on the best trade-off between higher cross-validated predictive performance and a smaller feature set. Single-omics *Logistic* models were then developed using the selected proteomic and metabolomic features, respectively. Finally, to integrate multi-omics information,^47^ a *Logistic* generalizer combined predictions from individual proteomic and metabolomic models to develop the final multi-omics stacking model. To avoid overfitting, the classification accuracy of both single-omics and multi-omics models was assessed using the mean AUC from cross-site validation, where one of eight centers (excluding the Xiangya site, which included only one participant) is rotated as the independent validation set and the remaining seven are combined as the discovery set (see Supplementary Fig. S5), as suggested in a prior study.^48^ The mean AUC was used as the primary metric, along with additional measures including mean accuracy, balanced accuracy, sensitivity, and specificity. DCA was performed to explore potential clinical net benefit,^49^ and model calibration was also assessed. Details of machine learning modeling are provided in Supplementary Methods.

The analyses were conducted using *Python* (Version 3.9, pingouin 0.5.3 for statistical analysis, scikit-learn 1.3.0 for variable selection and machine learning modeling) and *R* (Version 4.3.3, pROC 1.18.5 for DeLong’s test of AUCs).

## Supplementary information

Supplementary Materials Revision 2 clean

Study Protocol

## Data Availability

The multi-omics models (metabolomic, proteomic, and stacking models for each of the eight sites, developed with cross-site validation) are available at https://github.com/sjyzhu-bjmu/Pal_treatment_prediction. The CATIE cohort datasets are available from their original sources. For the prospective cohorts (ChiCTR2100048320, ChiCTR-TRC-10000934, and ChiCTR-RNC-09000522), the multi-omics data used in this study are stored in the Peking University Open Research Data Platform (doi:10.18170/DVN/FTOJ8I). In accordance with the Regulation of the People’s Republic of China on the Administration of Human Genetic Resources and the constraints of Peking University, access to individual-level human genetic resource data requires submission of a data access request, which will be approved upon meeting the necessary criteria. Data requests should first contact Prof. Weihua Yue (dryue@bjmu.edu.cn) and will be reviewed by an independent committee on a case-by-case basis. A signed data access agreement with the sponsor is required prior to sharing.
