# Supplementary material for: Multi-omics reveal critical roles of phosphatidylcholine and sphingomyelin in antipsychotic efficacy for schizophrenia
Source: Signal Transduct Target Ther. 2025 Oct 13;10:343. doi: 10.1038/s41392-025-02431-4 (PMC12518867; doi:10.1038/s41392-025-02431-4)
Supplement: Supplementary file 1 — Supplementary Materials Revision 2 clean [file 41392_2025_2431_MOESM1_ESM.docx]

Supplementary Materials for

Multi-omics reveal critical roles of phosphatidylcholine and sphingomyelin in antipsychotic efficacy for schizophrenia

Junyuan Sun^1†^; Zhe Lu^1†^; Zhewei Kang^1^; Yuyanan Zhang^1^; Yaoyao Sun^1^; Guorui Zhao^1^; Qijing Bo^2^; Wenqiang Li^3^; Zhenghui Yi^4^; Binbin Chen^5^; Yuandong Gong^6^; Zhenhe Zhou^7^; Huiling Wang^8^; Lin Lu^1,9^**, Weihua Yue^1,9^*

^†^These authors contributed equally: Junyuan Sun, Zhe Lu

* Correspondence: dryue@bjmu.edu.cn (W.Y.)

** Co-correspondence: linlu@bjmu.edu.cn (L.L.)

**This PDF file includes:**

Supplementary Methods

Supplementary Figures S1-S6

Supplementary Tables S1-S14

Supplementary Methods

1. Clinical information in the multi-omics cohort

Nine clinical centers including Peking University Sixth Hospital, Beijing Anding Hospital, The Second Affiliated Hospital of Xinxiang Medical College, Shanghai Mental Health Center, Xiamen Xianyue Hospital, Shandong Mental Health Center, Wuxi Mental Health Center, Renmin Hospital of Wuhan University and the Second Xiangya Hospital. The protocol was approved by the institutional ethics review boards at each site, with central supervision by the Medical Ethical Committee of the Sixth Hospital of Peking University (approval number: 2021-LunShen-No.40). Baseline and post-treatment PANSS total and subscale scores are provided in Supplementary Table S14. The study protocol for the multi-omics cohort is provided in Supplementary Files.

1. Inclusion and exclusion criteria of the multi-omics cohort

**Inclusion criteria:** (1) Diagnosed with schizophrenia based on the Mini International Neuropsychiatric Interview (MINI-Plus) of the Diagnostic and Statistical Manual of Mental Disorders, fourth edition (DSM-IV); (2) Aged 18 to 45 years, any genders, Han Chinese ancestry and both biological parents are Han Chinese; (3) First-episode with unmedicated or chronic course currently in an acute episode; (4) A total PANSS score of ≥60 (on a scale of 1-7)^1,2^, requiring scores of ≥4 on at least 3 of the 7 positive symptoms; (5) Obtain written informed consent from the patient or their legal guardian.

**Exclusion criteria:** (1) Pregnant, breastfeeding or pregnancy planning; (2) Contraindication to paliperidone (3) With unstable physical diseases; (4) With the following heart conditions: prolonged QTc interval (QTc ≥450 ms in males or QTc ≥470 ms in females on ECG); decompensated congestive heart failure; complete left bundle branch block.

1. *Genomic, Proteomic and metabolomic profiling*

All blood samples were transported via cold chain from the other participating centers and stored at Peking University Sixth Hospital. After aggregation, genomic, proteomic, and metabolomic profiling was conducted under standardized conditions using the same platforms, to controlling batch effects.

**(1) Genotyping, imputation, and quality control**

For the multi-omics cohort, samples were genotyped with Infinium Asian Screening Array (Illumina, San Diego, CA, USA), which were designed for East Asian populations. A total of 743,722 SNPs were successfully genotyped. Before imputation, several quality control measures were applied. Samples with call rate < 0.98, mismatched sex information, first-degree or second-degree relatives, and those with heterozygosity ≥ three standard deviations from the mean were excluded. Variants were filtered out if they had a call rate < 0.98, Hardy-Weinberg equilibrium deviations P < 1×10^-10^, or a minor allele frequency (MAF) < 0.01. After these steps, 224 qualified samples and 289,525 variants were retained for imputation. The qualified samples and genotypes were phased using SHAPEIT^3–5^ (Version 2). Imputation was performed using IMPUTE (Version 2) with the 1000 Genomes Project East Asian reference panel (1000G EAS). This resulted in a total of 37,662,208 variants being successfully imputed. After filtering for well-imputed variants with imputation quality scores > 0.6, 10,507,772 variants were retained. The present study additionally performed the genomic quality control for the imputed variants. Variants with a call rate < 0.05 or MAF < 0.01 were excluded, leaving a total of 3,496,532 variants for subsequent analysis. Clumping was performed in PLINK with a cutoff *r^2^* of 0.2 within a 250-kb window. Genes were annotated by SNPs based on Ensembl database (http://www.ensembl.org) ^6^.

The plasma PRS validation cohort includes patients from two Chinese Han schizophrenia cohorts. For the first Chinese Han cohort, the genotyping of samples was performed on Illumina Human OmniZhongHua BeadChips (Illumina, San Diego, CA, USA) designed for the Chinese population. For the second Chinese Han cohort, the genotyping of samples was performed on the Illumina HumanHap610Quad BeadChips (Illumina, San Diego, CA, USA). For quality control in these Chinese cohorts, samples were excluded if the genotype call rate was less than 98%, in the case of gender discordance, if they were first-degree or second-degree relatives, or if they were genetic outliers. SNPs were excluded if minor allele frequency was less than 0·01, the genotype call rate was less than 98%, or p values for Hardy-Weinberg equilibrium were less than 1×10^-^⁵. We did principal component analyses (following the methods of EIGENSTRAT18 software) to identify genetic outliers, which were defined as individuals whose ancestry was more than 6 SDs from the mean on one of the top two inferred axes of variation. Genotype imputation for the discovery sample was done with the pre-phasing imputation stepwise approach implemented in IMPUTE2 and SHAPEIT (version 2.r727).20,21 Haplotypes derived from phase I of the 1000 Genomes Project (release version 3) were used as references. SNPs with imputation quality scores below a set threshold (info score <0.9) were excluded from further analyses. Details were showed in our previous studies^1^.

The brain PRS validation cohort includes patients from the CATIE cohort, the genotyping of samples was performed on Affymetrix (Santa Clara, CA, USA) 500 K “A” chipset and the Perlegen custom 164 K chip. Missing SNPs of CATIE samples were imputed using the Gimpute R package^7^ and 1000 Genome Phase 3 as reference. Before imputation, QC steps were performed using default settings to check for genotyping quality, sex chromosome heterozygosity, missingness, and deviation from Hardy-Weinberg equilibrium. After imputation, additional QC steps were performed to ensure that variants had an imputation INFO score greater than 0.8 and were successfully imputed in more than 20 samples.

**(2) Proteomic profiling**

**Depletion of Highly Abundant Proteins from Serum Samples**

High-Select Top14 Abundant Protein Depletion of serum samples was conducted according to the manufacturer instructions for the Multi Affinity Removal Column, Human-14 (Agilent Technologies). Briefly, 20 μL of serum and 60 μL of buffer A were mixed evenly, transferred into a Spin-X centrifuge tube filter 0.22 μm cellulose acetate certified tube (COSTOR), and subsequently centrifuged at 16,000g for 1 min. The filtrate was transferred into preassembled plastic springs measuring 29 × 5 mm for High-Select Top14 Abundant Protein Depletion using a liquid chromatography system (Agilent Technologies 1290). The depleted samples were collected, and the protein concentration was measured using a BCA Protein Assay Kit (Thermo Scientific), following the manufacturer’s instructions.

**Sample Preparation for DIA-MS**

The depleted serum sample (50 μg) was precipitated with 1/3 volume of trichloroacetic acid (TCA) solution at 4 °C overnight and then centrifuged at 16,000g for 30 minutes at 4 °C. The precipitate was washed three times with acetone and then dried with a vacuum concentrator (Labconco, USA). The dried precipitate was dissolved in 40μL of 8 M urea (pH 8.5), incubated with 20 mM (2-carboxyethyl) phosphinehydro chloride (TCEP), and alkylated with 40 mM IAA. The mixture was diluted with 200 μL of 100 mM Tris−HCl buffer (pH 8.5) to a final concentration of 1.3 M urea and then digested with 3 μg of trypsin protease at 37 °C for 16 hours. The sample was desalted using a Monospin C18 column (GL Science, Tokyo, Japan). The desalted peptides were vacuum-centrifuged to dryness and then reconstituted in Milli-Q water containing 0.1% formic acid (FA) for LC−MS analysis. Indexed retention time (iRT) calibration peptides were spiked into the samples prior to DIA analysis.

**LC-MS/MS analysis**

The LC-MS/MS analysis was performed on an EASY-nLC 1200 HPLC (Thermo Scientific) coupled to a Q Exactive HF-X (Thermo Scientific) mass spectrometer. All samples were separated within 90 min on a reversed-phase C18 column (25 cm×75μm, 1.7μm, IonOpticks). The elute gradient of B was linearly increased from 9 % to 27 % within 70 min, followed by an increase to 45 % within 8 min, an increase to 95% for 5 min, and continued for another 7 min at a flow rate of 300 nL/min at 50℃ (Mobile phases A: 0.1% formic acid in water; Mobile phases B: 0.1% formic acid in 80% acetonitrile).

The mass spectrometer was operated in positive mode using DIA modes. The MS1 scan resolution was set to 60 000. The MS1 scan range was 350−1200 m/z, the AGC target was 3 x106, and the maximum injection time was 50 ms. Precursors were isolated with a dynamic isolation width covering 350−1200 m/z. Precursors were fragmented by HCD at an NCE of 28. MS2 scans were acquired by Orbitrap at resolution of 30 000. The maximum injection time was set to auto, AGC target was set to 1x106.

**DIA Data Analysis**

Data analysis was using Spectronaut version 17.0 (Biognosys). The MS/MS spectra were matched against the human UNIPROT database (20,230 human entries, downloaded in July 2019). DIA files were processed in default mode. Briefly, Searches used carbamidomethylation as fixed modification and acetylation of the protein N-terminus and oxidation of methionine as variable modifications. The Trypsin/P proteolytic cleavage rule was used, permitting a maximum of 2 missed cleavages, a minimum peptide length of 7 amino acids and a maximum peptide length of 52 amino acids. The FDR for PSM and protein quantification were both set to 0.01. The protocols described above referred to previously published protocols^8,9^

**(3) Metabolomic profiling**

A combination of six LC–MS/MS methods were used to profile metabolites and lipids in all plasma samples. Additionally, pairs of pooled reference samples were inserted into the queue at intervals of approximately 20 samples for quality control and data standardization. Samples were prepared for each method using extraction procedures that are matched for use with the chromatography conditions. AB SCIEX 5500+ triple quadrupole LC-MS/MS system and stable-isotope labelled internal standard calibration was employed for assay 1~4, on the other hand, AB SCIEX 5500+ triple quadrupole LC-MS/MS system and single-point calibration with stable-isotope labelled lipids was used for assay 5 and 6. Selective response detection mode SRM was used to collect data. Method details are summarized below^10–12^.

**LC-MS/MS Assay 1**

Accurately transfer 40 μL plasma to 96-well plate (Waters Corporation, USA), add 10 μL isotope internal standard liquid (internal standard concentration 5.0 μg/mL) and 150 μL methanol, and then swirl for 1500 rpm × 3 min. Centrifugation at 2000 g × 20 min was supernatants injected directly onto a 1.7 μm, 2.1 × 100 mm Acquity UPLC BEH Amide column. The column was eluted isocratically at a flow rate of 0.3 mL/min with 100% mobile phase A (10mM ammonium formate and 0.1% formic acid in 75:25 v/v water/acetonitrile) for 0.5 min followed by a linear gradient to 70% mobile phase B (10mM ammonium formate and 0.1% formic acid in 50:50 v/v water/acetonitrile) over 16 min. Ionization parameters: Turbo V electrospray Ion Source Negative IonSpray Voltage -4.5kV, Positive IonSpray Voltage 5.5 kV, Temperature 550℃, Curtain Gas 35 psi, Collision Gas 9, on Source Gas1 50 psi, Ion Source Gas2 50 psi.

**LC-MS/MS Assay 2**

Precise transfer of 40 μL plasma to 96-well plate (Waters Corporation, USA), adding 20 μL isotope internal standard mixing liquid and 140 μL methanol/acetonitrile (1/1, V/V, containing isotope internal standard), and vortex oscillation at 1500 rpm × 3 min, Centrifuge at 2000 g × 20 min was supernatants injected directly onto a 1.9 μm, 2.1 × 100 mm Thermo Hypersil GOLD column. The column was eluted isocratically at a flow rate of 0.4 mL/min with 83% mobile phase A (2mM ammonium acetate in water) for 0.5 min followed by a linear gradient to 95% mobile phase B (acetonitrile) over 27 min. Ionization parameters: Turbo V electrospray Ion Source Negative IonSpray Voltage -4.5kV, Positive IonSpray Voltage 5.5 kV, Temperature 550℃, Curtain Gas 35 psi, Collision Gas 9, on Source Gas1 50 psi, Ion Source Gas2 50 psi.

**LC-MS/MS Assay 3**

Accurately transfer 20 μL plasma into 1.5 mL EP tube (Corning Axygen, USA), add 80 μL methanol/acetonitrile (1/1, V/V), swirl at 1500 rpm × 3 min, centrifuge at 12000 g × 20 min. Transfer 40 μL of supernatant into another 1.5mL EP tube, Then, 5 μL internal standard mixture (internal standard concentration of isotope 0.4~400 μg/mL), 20 μL 200 mM 3-nitrophenylhydrazine and 20 μL 200 mM 1-ethyl -(3-dimethylaminopropyl) carbodiimide were added, mixed and reacted in a 30℃ incubator for 30 min. After vacuum vacuum drying (Labconco Corporation, USA), the reaction product was re-dissolved with 800 μL 50% methanol,and the reconstitution solution injected directly onto a 1.8 μm, 2.1 × 100 mm ACQUITY UPLC® HSS C18 column. The column was eluted at a flow rate of 0.3 mL/min with initial conditions of 85% mobile phase A (0.01% formic acid in water) and 15% mobile phase B (acetonitrile) followed by a 26-min linear gradient to 95% mobile phase B. Ionization parameters: Turbo V electrospray Ion Source Negative IonSpray Voltage -4.5kV, Positive IonSpray Voltage 5.5 kV, Temperature 550℃, Curtain Gas 35 psi, Collision Gas 9, on Source Gas1 50 psi, Ion Source Gas2 50 psi.

**LC-MS/MS Assay 4**

Accurately transfer 50 μL plasma to 96-well plate (Waters Corporation, USA), add 10 μL internal standard liquid (internal standard concentration range 0.2-8.0 μg/mL) and 190 μL methanol/acetonitrile (1/1, V/V), After swirling for 1500 rpm × 3 min, centrifuge for 2000 g × 20 min, transfer 120 μL supernatant to another 96-well plate (Thermo Scientific, USA), and add 120 μL ultra-pure water to dilute. After swirling at 1500 rpm × 3 min and centrifugation at 2000 g × 20 min, and the supernatants injected directly onto a 2 μm, 2.1 × 100 mm ACE Excel PFP C18 column. The column was eluted at a flow rate of 0.3 mL/min with initial conditions of 95% mobile phase A (0.01% formic acid in water) and 5% mobile phase B (acetonitrile) followed by a 12-min linear gradient to 95% mobile phase B. Ionization parameters: Turbo V electrospray Ion Source Negative IonSpray Voltage -4.5kV, Positive IonSpray Voltage 5.5 kV, Temperature 550℃, Curtain Gas 35 psi, Collision Gas 9, on Source Gas1 50 psi, Ion Source Gas2 50 psi.

**LC-MS/MS Assay 5**

Accurately transfer 50 μL plasma to 96-well plate (Waters Corporation, USA), add 50 μL isotope internal standard liquid (internal standard concentration range 0.5~1.0 μg/mL) and 150 μL methanol, and then swirl for 1500 rpm × 3 min. After centrifugation at 2000 g × 20 min, and the supernatants injected directly onto a 2 μm, 2.1 × 100 mm ACE Excel PFP C18 column. The column was eluted isocratically at a flow rate of 0.4 mL/min with 98% mobile phase A (0.1% formic acid in water) for 0.5 min followed by a linear gradient to 98% mobile phase B (acetonitrile) over 13.5 min. Ionization parameters: Turbo V electrospray Ion Source Negative IonSpray Voltage -4.5kV, Positive IonSpray Voltage 5.5 kV, Temperature 550℃, Curtain Gas 35 psi, Collision Gas 9, on Source Gas1 50 psi, Ion Source Gas2 50 psi.

**LC-MS/MS Assay 6**

Accurately transfer 20 μL plasma into the first 1.5mL EP tube (Corning Axygen, USA), add 120 μL isotope internal standard mixed solution, swirl at 1500 rpm × 3 min, add 360 μL methyl tert-butyl ether and 100 μL ultra-pure water. Swirl and shake at 1500 rpm × 3 min, leave for 3 min in a refrigerator at 4℃, centrifuge at 12000 g × 15 min, and transfer 300 μL of the upper layer extract into the second EP tube (Corning Axygen, USA). 360μL methyl tert-butyl ether was added again into EP tube No. 1, and the mixture was vortex shaken at 1500 rpm × 3 min, then left for 3 min in refrigerator at 4℃, centrifuged at 12000 g × 15 min, and then 300 μL of upper extract was transferred again to EP tube No. 2, and vortex mixed well. After vacuum drying (Labconco Corporation, USA), the extract was re-dissolved with 80 μL 50% isopropyl alcohol/acetonitrile mixture (50/50, V/V),and the reconstitution solution injected directly onto a 1.8 μm, 2.1 × 100 mm ACQUITY UPLC® HSS C18 column. The column was eluted isocratically at a flow rate of 0.25 mL/min with 80% mobile phase A (5 mM ammonium acetate in 40:60 v/v water/acetonitrile) for 2 min followed by a linear gradient to 98% mobile phase B (5 mM ammonium acetate in 90:10 v/v isopropanol/acetonitrile) over 21.5 min. Ionization parameters: Turbo V electrospray Ion Source Negative IonSpray Voltage -4.5kV, Positive IonSpray Voltage 5.5 kV, Temperature 550℃, Curtain Gas 35 psi, Collision Gas 9, on Source Gas1 50 psi, Ion Source Gas2 50 psi.

Metabolites or lipids with a coefficient of variation greater than 30% in QC samples were excluded for further data analysis. Analyst software 1.7 and OS-MQ software (AB SCIEX, Singapore) were employed for metabolites and lipids quantitative data processing. The metabolites were quantified by stable isotope internal standard curve method. The lipids were quantified by single point correction of stable isotope lipids internal standard. The m/z window for extracting product ions were set at 10 ppm.

**(3) Preparing for analysis**

To maximize molecule retention for further exploration, molecules in the original dataset with retention > 25% across all schizophrenia samples were retained. According to previous studies^13^, missing values were filled with the median value of all samples. Finally, data were *Log2* transformed for further differential analysis.

1. Machine learning modeling

To follow methodological and transparency recommendations for clinical prediction studies, the TRIPOD checklist (METHODS and RESULTS) for machine learning in the multi-omics cohort is provided in Supplementary Table S13. Logistic Regression (LR) models with a LASSO penalty were employed for variable selection, identifying proteomic and metabolomic features that effectively distinguish between treatment responders (TR) and non-responders (NTR). As only one participant was from the Xiangya site, cross-site validation included eight sites (n=207), while all 208 patients from nine sites were included in LASSO and differential analyses for comprehensiveness.

For LASSO logistic regression, optimal λ values were determined via 5-fold cross-validations. We tested logistic LASSO models across a range of regularization strengths (logE from –3 to 3), selecting parameters that yielded at least 25 informative features to balance model complexity and predictive performance, particularly in light of the relative sample size of 208 participants in this study. Subsequently, LASSO was applied using varying numbers of top-ranked features (5, 10, 15, 20, and 25), and the number of features was selected based on the best trade-off between higher cross-validated predictive performance and smaller feature set. Single-omics Logistic models were then developed using the selected proteomic and metabolomic features, respectively. Top 20 variables with non-zero coefficients features were applied for further machine learning modeling (see Supplementary Fig. S2).

Using optimal classification features, we developed machine learning models to distinguish between TR and NTR, based on proteomic and metabolomic data respectively. To minimize overfitting or underfitting, model classification accuracy was assessed using the mean AUC from cross-site validation, in which each of the eight centers (with sample sizes of 14, 24, 75, 18, 8, 18, 30, and 20) was selected in rotation as the independent testing set, while the remaining seven centers were combined as the training set. Specifically, model hyperparameters were optimized in the training set (remaining seven sites) using 5-fold cross-validated grid search methods. The parameter grid included penalty (L1/L2), regularization strength C (log-scale from 1e-6 to 1e6), and solver (liblinear). After hyperparameter optimization and model training, performance was further validated in the independent testing set (each of the eight centers in rotation). For each subject in the validation cohort, predicted probabilities were obtained using the *predict_proba()* method from scikit-learn applied to the fitted model object.

In each fold of cross-site validation, the proteomic and metabolomic models were combined using a stacking approach to leverage the strengths of each model, enhancing overall performance^14^. The LR algorithms were selected as generalizers for the ensemble models, and their predictive performance was also measured using the mean AUC from cross-site validation (Supplementary Fig. S6).

For statistic power, we assessed the classification accuracy of models using AUC. Power calculations performed with MedCalc (version 23.0) indicated that a sample size of 22 participants would achieve a power of 0.99, assuming an AUC > 0.90, a null hypothesis value of 0.50, and a type I error rate (two-tailed alpha) of 0.05.

1. Validation datasets
2. ***Plasma metabolites PRS validation dataset from two Chinese Han cohorts***

A total of 2281 patients with complete genotype and outcome data were included in the first validation dataset, from two Han Chinese schizophrenia cohorts collected previously^1^.

The first cohort consisted of 1999 patients (1037 males and 962 females; mean age 31.47 ± 7.91 years) enrolled in a randomized clinical trial (ChiCTR-TRC-10000934) conducted by the Chinese Antipsychotics Pharmacogenomics Consortium (CAPOC). Patients were randomized to receive aripiprazole, olanzapine, quetiapine, risperidone, ziprasidone, haloperidol and perphenazine for 6 weeks. For the NTR (n=760) and TR (n=1239) groups, the mean ages were 31.50 ± 7.83 and 31.46 ± 7.95 years, with 50.8% and 52.5% male, illness durations of 7.36 ± 5.84 and 6.20 ± 6.03 years, and second-generation antipsychotic use in 83.6% and 84.2% of patients, respectively.

The second cohort included 282 patients (126 males and 156 females; mean age 32.94 ± 10.76 years) who participated in another randomized clinical trial (ChiCTR-RNC-09000522). Patients were assigned to receive olanzapine, aripiprazole, risperidone, quetiapine, clozapine, or ziprasidone for 8 weeks. Institutional ethics review boards approved all study protocols, and informed consent was obtained. For the NTR (n=75) and TR (n=207) groups, the mean ages were 37.21±11.43 and 30.83±9.94 years, with 26.7% and 51.2% male, illness durations of 5.23±6.50 and 2.64±5.33 years, and second-generation antipsychotic use in 93.3% and 94.7% of patients, respectively.

The plasma metabolites PRS validation cohort included 835 non-responders and 1446 responders based on 50% PANSS reduction rate. Detailed information of these two cohorts provided below.

*a) The first Chinese Han cohort:*

The first cohort was from the Chinese Antipsychotics Pharmacogenomics Consortium (including five research centers: Peking University Sixth Hospital, West China Hospital of Sichuan University, the Second Xiangya Hospital of Central South University, Beijing Anding Hospital Affiliated to Capital Medical University, and Beijing HuiLongGuan Hospital; the Consortium leads 32 psychiatric hospitals in China in total).

This study was conducted in accordance with the Declaration of Helsinki. The protocol was approved by the institutional ethics review boards at each site, with central supervision by the Medical Ethical Committee of the Sixth Hospital of Peking University (approval number: 2009-LunShen-No.23). And the written informed consent was obtained.

Inclusion criteria: 1) Diagnosed with schizophrenia based on the Structured Clinical Interview of the Diagnostic and Statistical Manual of Mental Disorders, fourth edition (DSM-IV); 2) Aged from 18 to 45 years; 3) Han Chinese ancestry; 4) Total scores of the Positive and Negative Syndrome Scale (PANSS) were more than 60, and three positive items scored more than four at least; 5) Physically healthy with all laboratory parameters within normal limits; 6) Could be treated with oral medication; 7) Provide informed consent.

Exclusion criteria: 1) Diagnosed with other mental disorders met the criteria of DSM-IV; 2) With unstable physical diseases, malignant syndrome or acute dystonia, well documented histories of epilepsy and hyperpyretic convulsion; 3) Required long-acting injectable antipsychotics; 4) Regularly toke with clozapine during the past month; 5) Treated with electroconvulsive therapy during the last month; 6) Had previously attempted suicide, or had experienced the symptoms of severe excitement and agitation; 7) Abnormal liver or renal function; 8) without legal guardian; 9) Had QTc prolongation, a history of congenital QTc prolongation, or myocardial infarction within the past 6 months; 10) Pregnant or breastfeeding; 11) Had a contraindication to any of the drugs to which they could be assigned.

Eligible patients were randomly assigned (2:2:2:2:2:1:1) to seven groups (aripiprazole, olanzapine, quetiapine, risperidone, ziprasidone, haloperidol and perphenazine) and received 6-week antipsychotic treatment. Group assignment was determined by a Microsoft Excel randomization generator. The random allocation sequence was generated by a trained research assistant. Then, we performed several baseline assessments at the start of the study. The patients who meet the criterion were followed for up to six weeks or until treatment was discontinued for any reason.

All patients were given a screening questionnaire, which recorded the demographic and clinical information. We did baseline assessments to ensure that participants met inclusion criteria. Patients who were already taking antipsychotic medications were obliged to switch to their newly assigned drug within 1 week of randomization. Within 2 weeks of randomization, psychiatrists from the study adjusted the dosages on the basis of treatment effectiveness, in keeping with the study protocol (olanzapine doses could range from 5 mg to 20 mg per day, risperidone from 2 mg to 6 mg per day, quetiapine from 400 mg to 750 mg per day, aripiprazole from 10 mg to 30 mg per day, ziprasidone from 80 mg to 160 mg per day, haloperidol from 6 mg to 20 mg per day, and perphenazine from 20 mg to 60 mg per day). The dosage of antipsychotics then remained unchanged throughout the study. If the psychiatrists decided that a patient’s response was not adequate or the patient decided to drop out of the study, treatment was discontinued and the last observation was carried forward to represent treatment response. Patients with adequate responses continued treatment until the end of the study.

Demographic information was collected by the self-designed report form, which including age, sex, course of schizophrenia (month), dosage of antipsychotics, height, weight at each viewpoint, waist circumference and so on. Laboratory test was conducted at baseline, the 4th week and the 6th week, including the blood routine, blood lipids (including cholesterol, triglycerides, high density lipoprotein, low density lipoprotein) and blood sugar. The severity was evaluated by the Positive and Negative Syndrome Scale (PANSS).

*b) The second Chinese Han cohort:*

The second cohort was from the Chinese Antipsychotics Pharmacogenetics Consortium (CAPEC, including Peking University Sixth Hospital, Beijing Huilongguan Hospital, the Sixth Hospital of Hebei Province, Jinzhou Kangning Hospital, and Xi’an Mental Health Centre). This study was conducted in accordance with the Declaration of Helsinki. The protocol was approved by the institutional ethics review boards at each site, with central supervision by the Medical Ethical Committee of the Sixth Hospital of Peking University (approval number: 2009-LunShen-No.23). And the written informed consent was obtained.

Individuals included in the CAPEC cohort had a diagnosis of schizophrenia based on the Structured Clinical Interview of DSM-IV, were aged 18–45 years, were of Han Chinese ancestry, scored more than 60 on the PANSS (and scored more than four on at least three positive items), were physically healthy with all laboratory parameters within normal limits, had a condition that could be treated with oral medication, and were able to provide informed consent. Both first-episode and relapsed patients with schizophrenia were enrolled from the inpatient departments of the psychiatric hospitals.

Patients were excluded from the study if they were diagnosed with schizoaffective disorder, delusional disorder, brief psychotic disorder, schizophreniform disorder, psychosis associated with substance use or medical conditions, learning disability, pervasive develop-mental disorder, delirium, dementia, amnesia, or other cognitive disorders; had severe, unstable physical diseases (such as diabetes, thyroid diseases, hypertension, and cardiac diseases), malignant syndrome or acute dystonia, well documented histories of epilepsy and hyperpyretic convulsion, a DSM-IV diagnosis of alcohol or drug dependence, or a history of drug-induced neuroleptic malignant syndrome; required long-acting injectable medication to maintain treatment adherence; were regularly treated with clozapine for treatment resistance during the past month (patients who had taken clozapine for reasons other than treatment resistance were eligible); were treated with electroconvulsive therapy during the last month; had previously attempted suicide, or had experienced the symptoms of severe excitement and agitation; had abnormal liver or renal function (ie, aspartate aminotransferase ≥80 U/L, alanine aminotransferase ≥80 U/L, blood urea nitrogen ≥9·75 mmol/L, urine creatinine ≥21·6 mmol per day); did not have a legal guardian (it was a hospital stipulation that written informed consent was required from the patient's legal guardian); had QTc prolongation, a history of congenital QTc prolongation, or recent (ie, within the past 6 months) myocardial infarction; were pregnant or breastfeeding; or had a contraindication to any of the drugs to which they could be assigned. Inclusion and exclusion criteria were the same for the discovery and validation cohorts.

In the CAPEC cohort, patients were randomly assigned to aripiprazole, olanzapine, quetiapine, risperidone, or ziprasidone. The CAPEC study was launched with an 8-week observation period. Within 2 weeks of enrolment, participating psychiatrists in the CAPEC study adjusted drug dosages on the basis of treatment effectiveness, in keeping with the study protocol (olanzapine doses could range from 5 mg to 20 mg per day, risperidone from 2 mg to 6 mg per day, quetiapine from 400 mg to 750 mg per day, aripiprazole from 10 mg to 30 mg per day, ziprasidone from 80 mg to 160 mg per day). The dosage of antipsychotics then remained unchanged throughout the study period. Patients were seen by a participating psychiatrist at baseline and at weeks 4 and 8, and their PANSS scores were recorded.

***(2) Brain metabolites PRS validation dataset from a European cohort***

The brain PRS validation cohort consisted of 449 patients (348 males and 101 females; mean age 41.11 ± 11.40 years) with complete genotype and outcome data from the European population of the Clinical Antipsychotic Trials of Intervention Effectiveness in Schizophrenia (CATIE) cohort. The CATIE schizophrenia trial was a multi-site, multi-phase randomized controlled trial involving patients chronically suffering from schizophrenia. For the NTR (n=384) and TR (n=65) groups, the mean ages were 41.64±11.40 and 37.97±10.94 years, with 78.1% and 73.8% male, illness durations of 14.64±11.22 and 10.54±8.80 years, and second-generation antipsychotic use in 83.1% and 73.8% of patients, respectively.

The CATIE study was approved by the institutional review board at each site, and written informed consent was obtained from the subjects or their legal guardians. Additional details are provided at https://clinicaltrials.gov/study/NCT00014001. Patients were followed for up to 18 months. Phase 1/1A involved a randomized, controlled, double-blind comparison of five different treatments, including olanzapine, quetiapine, risperidone, and ziprasidone as second-generation antipsychotics, and perphenazine as a first-generation antipsychotic. To validate the findings from other cohorts, we extracted data at baseline and the 12th week in the CATIE. 384 non-responders and 65 responders were included based on a 50% PANSS reduction rate. Responders were fewer in the CATIE cohort compared to the first dataset, as the CATIE cohort primarily consisted of individuals chronically suffering from schizophrenia. Detailed information about the CATIE cohort can be found in a previous publication^15^.

**References**

1. Yu, H. *et al.* Five novel loci associated with antipsychotic treatment response in patients with schizophrenia: a genome-wide association study. *Lancet Psychiatry* **5**, 327–338 (2018).

2. Wang, Q. *et al.* Effect of damaging rare mutations in synapse-related gene sets on response to short-term antipsychotic medication in Chinese patients with schizophrenia: a randomized clinical trial. *Jama Psychiatry* **75**, 1261–1269 (2018).

3. Delaneau, O., Marchini, J. & Zagury, J.-F. A linear complexity phasing method for thousands of genomes. *Nat. Methods* **9**, 179–181 (2012).

4. Howie, B. N., Donnelly, P. & Marchini, J. A flexible and accurate genotype imputation method for the next generation of genome-wide association studies. *PLoS Genet.* **5**, e1000529 (2009).

5. Consortium, 1000 Genomes Project. *A Global Reference for Human Genetic Variation*. *Nature* vol. 526 68 (Nature Publishing Group, 2015).

6. Huang, D. *et al.* Ultrafast and scalable variant annotation and prioritization with big functional genomics data. *Genome Res.* **30**, 1789–1801 (2020).

7. Chen, J. *et al.* Gimpute: an efficient genetic data imputation pipeline. *Bioinformatics* **35**, 1433–1435 (2019).

8. Tian, W. *et al.* Immune suppression in the early stage of COVID-19 disease. *Nat. Commun.* **11**, 5859 (2020).

9. Zhang, Y. *et al.* Proteomics of Serum Samples for the Exploration of the Pathological Mechanism of Obstetric Antiphospholipid Syndrome. *J. Proteome Res.* **23**, 289–300 (2024).

10. Züllig, T., Trötzmüller, M. & Köfeler, H. C. Lipidomics from sample preparation to data analysis: a primer. *Anal. Bioanal. Chem.* **412**, 2191–2209 (2020).

11. Han, J., Lin, K., Sequeira, C. & Borchers, C. H. An isotope-labeled chemical derivatization method for the quantitation of short-chain fatty acids in human feces by liquid chromatography–tandem mass spectrometry. *Anal. Chim. Acta* **854**, 86–94 (2015).

12. Lv, W. *et al.* Comprehensive metabolite quantitative assay based on alternate metabolomics and lipidomics analyses. *Anal. Chim. Acta* **1215**, 339979 (2022).

13. Berkelmans, G. F. *et al.* Population median imputation was noninferior to complex approaches for imputing missing values in cardiovascular prediction models in clinical practice. *J. Clin. Epidemiol.* **145**, 70–80 (2022).

14. Chen, Y. *et al.* Human gut microbiome aging clocks based on taxonomic and functional signatures through multi-view learning. *Gut Microbes* **14**, 2025016 (2022).

15. Stroup, T. S. *et al.* The National Institute of Mental Health clinical antipsychotic trials of intervention effectiveness (CATIE) project: schizophrenia trial design and protocol development. *Schizophr. Bull.* **29**, 15–31 (2003).

Supplementary Figures


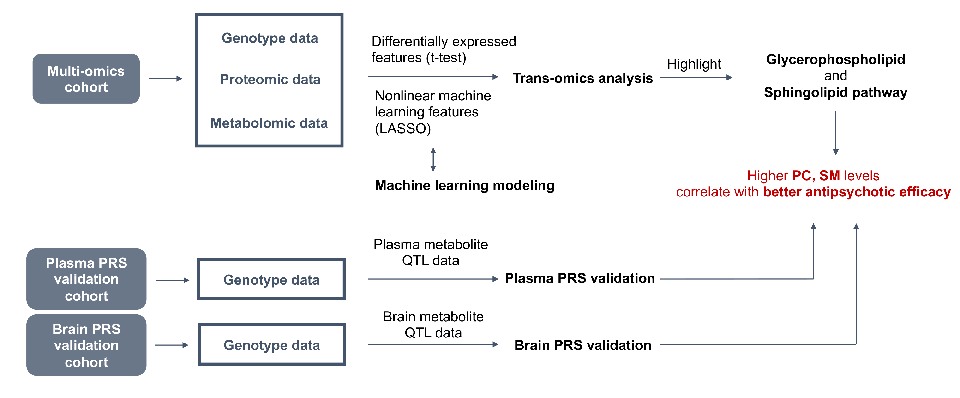


**Supplementary Fig. S1**. The main processes involved in the multi-omics analysis and independent PRS validation. Multi-omics data from the multi-omics cohort were analyzed to identify differentially expressed features and nonlinear machine learning predictive features. These features were integrated via trans-omics analysis, highlighting key pathways including glycerophospholipid and sphingolipid metabolism. Higher levels of PC and SM were found to be associated with better antipsychotic efficacy, and this association was further validated in the independent plasma and brain PRS validation cohorts using metabolite QTL data (from Metabolic GWAS summary statistics). LASSO, Least Absolute Shrinkage and Selection Operator; PC, phosphatidylcholine; PRS, polygenic risk score; QTL, quantitative trait loci; SM, sphingomyelin.

**Supplementary Fig. S2**. Cross-validation AUCs of models using different numbers of top-ranked features. Comparison of models using top 5, 10, 15, 20 and 25 features, the top 20 proteins and metabolites were selected as they provided the best balance between predictive performance and model simplicity.


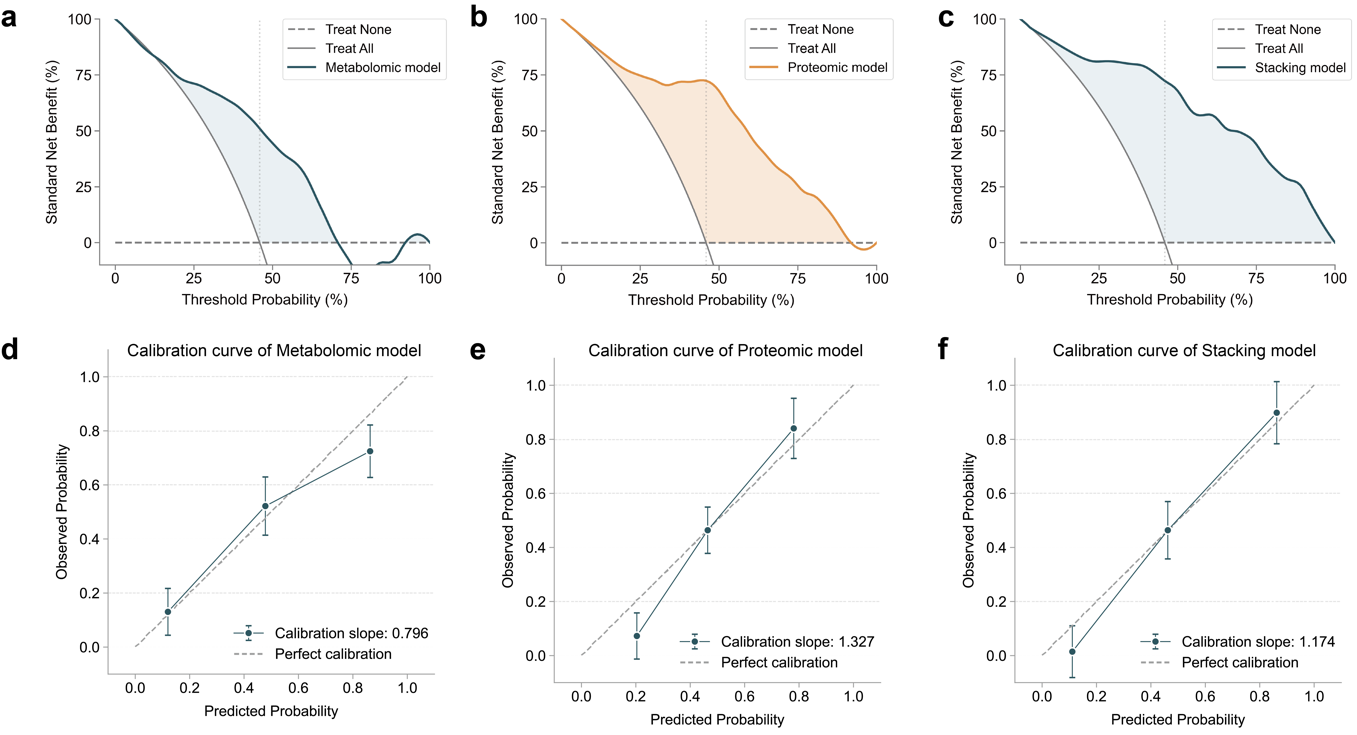


**Supplementary Fig. S3.** DCA and calibration curve plot of metabolomic, proteomic and stacking models. **a** DCA plot of metabolomic models. **b** DCA plot of proteomic models. **c** DCA plot of stacking models. **d** Calibration curve plot of metabolomic models. **e** Calibration curve plot of proteomic models. **f** Calibration curve plot of stacking models. Due to the limited sample size, three probability bins were used for calibration. DCA, Decision curve analysis; PC, Phosphatidylcholine; PC-O, ether-linked phosphatidylcholine; PE, phosphatidylethanolamine; SM, Sphingomyelin.

**
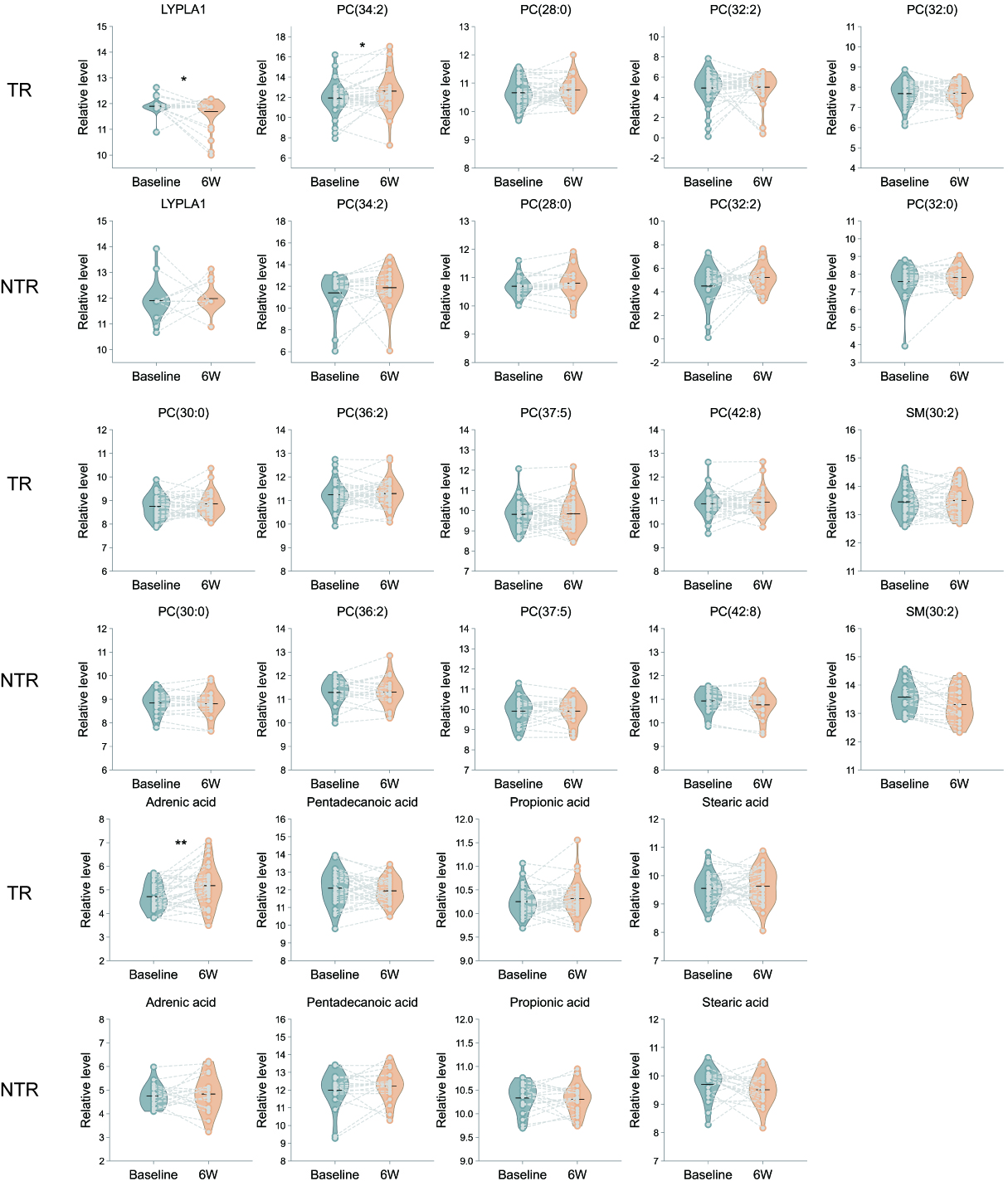
**

**Supplementary Fig. S4.** Other post-treatment changes in baseline significant phospholipid metabolism-related proteins and metabolites. 6W, after 6-week treatment; NTR, non-responders; PC, phosphatidylcholine; SM, sphingomyelin; TR, treatment responders. *****, *P* < 0.05; **, *P* < 0.01.


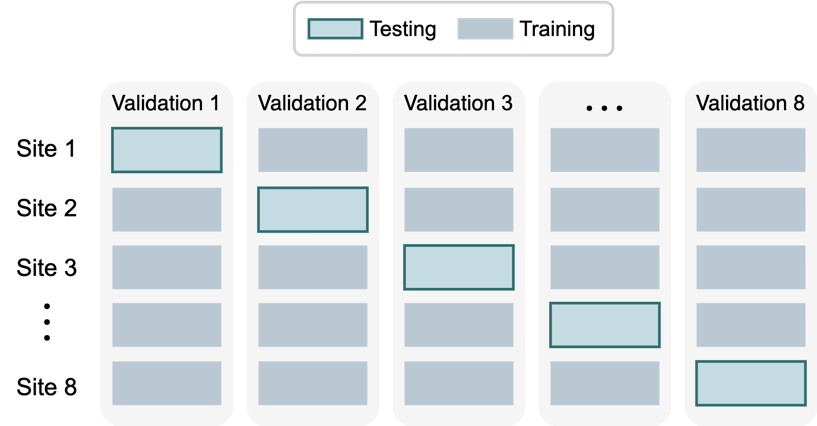


**Supplementary Fig. S5**. The diagram for cross-site validation in machine learning. In each round, the data from one site are held out as an independent validation set and are not used for training, while the data from the remaining sites are combined to form the discovery set for model development. This process is repeated until each site has been used as the validation set.


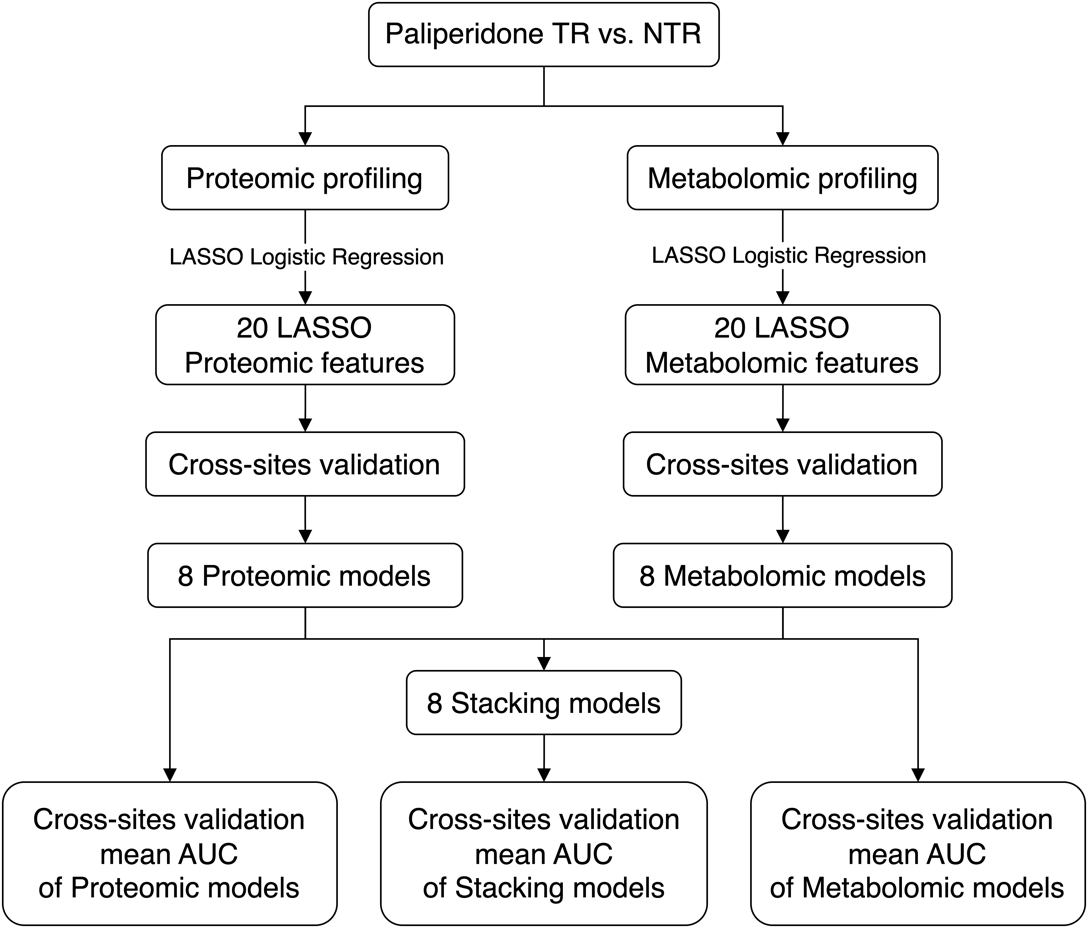


**Supplementary Fig. S6**. Proteomic and metabolomic machine learning modeling for paliperidone treatment response. Schematic overview of the machine learning workflow for distinguishing treatment responders and non-responders to paliperidone. Proteomic and metabolomic profiling data were analyzed separately. For each omics layer, LASSO logistic regression was used to select the top 20 features. Eight site-based models were developed for both proteomic and metabolomic data, and cross-sites validation was performed to assess model performance. Stacking models combining proteomic and metabolomic predictions were also constructed, with cross-sites validation to evaluate their performance. AUC, area under the curve; LASSO, Least Absolute Shrinkage and Selection Operator; NTR, non-responder; TR, treatment responder.

Supplementary Tables

| Supplementary Table S1. Metabolites and proteins identified with non-zero LASSO coefficients | |
| --- | --- |
| **LASSO Metabolites** | **coefficients** |
| PE(p16:0/18:2) | -1.19042049 |
| Hexanoylcarnitine (AcCa(6:0)) | 1.152277482 |
| 3-Hydroxyanthranilic acid | 0.800930957 |
| SM(30:2) | 0.791990961 |
| Omega-muricholic acid | -0.785929866 |
| 3beta-Hydroxy-5-cholenoic acid | 0.767414278 |
| 2-Hydroxy Hippuric Acid | 0.758750659 |
| PC(34:2) | 0.732239998 |
| cis-7,10,13,16-Docosatetraenoic acid | -0.714398726 |
| Taurochenodeoxycholic acid 3-sulfate | -0.711825909 |
| Glycoursodeoxycholic acid | -0.669674794 |
| Carnosine | -0.620397422 |
| PC-O(30:1) | -0.576202671 |
| PC(28:0) | 0.551824255 |
| Hypotaurine | 0.5517733 |
| Pentadecanoic acid | 0.542782992 |
| PC(32:2) | 0.538608556 |
| PC(32:0) | 0.512992416 |
| 1-Methylinosine | -0.497625756 |
| L-Histidine | 0.486354081 |
| Linoleic acid | -0.477079933 |
| TG(50:3-14:0) | 0.462247348 |
| PA(18:0/22:6) | -0.441779072 |
| PS(20:0/18:2) | 0.425542681 |
| Butyrylcarnitine (AcCa(4:0)) | -0.425385927 |
| PC(33:2) | 0.419617149 |
| Cholesterol | 0.41851545 |
| Linoleylcarnitine (AcCa(18:2)) | 0.406490317 |
| L-Valine | 0.406313047 |
| 3-Hydroxyglutaric acid | 0.40531294 |
| Fumaric acid | -0.398836018 |
| PE(p20:1/22:6) | -0.376935408 |
| Sarcosine | 0.37572412 |
| Indoleacetic Acid | 0.375690515 |
| PS(18:0/18:0) | -0.375443212 |
| SM(38:3) | -0.364380271 |
| PE(p18:1/20:4) | -0.360462614 |
| PC(38:6) | -0.347924299 |
| TG(50:1-14:0) | -0.344294868 |
| TG(46:2-14:0) | -0.343454923 |
| TG(50:5-14:0) | -0.328743366 |
| Pimelic acid | -0.328724815 |
| 4-Acetamidobutanoic acid | -0.31578627 |
| SM(38:2) | 0.311525991 |
| DihydroCer(24:0) | -0.304613995 |
| Arachidonoylcarnitine (AcCa(20:4)) | -0.293499008 |
| DG(18:0/18:1) | 0.288520938 |
| TG(54:2-18:2) | -0.285695947 |
| PE-O(38:5) | -0.283444192 |
| PE(38:2) | 0.280516262 |
| Eicosatrienoylcarnitine (AcCa(20:3)) | 0.272776318 |
| Propionic acid | -0.270079393 |
| PS(20:0/20:3) | 0.259873742 |
| PC(32:3) | -0.256938616 |
| PE(p18:0/22:6) | 0.2552458 |
| TG(52:6-16:0) | 0.25202132 |
| Deoxycholic acid | 0.242476967 |
| Nervonic acid | -0.234918484 |
| Imidazolepropionic acid | -0.234009553 |
| TG(52:5-16:0) | 0.233707105 |
| PA(20:0/20:4) | -0.228025644 |
| TG(52:5-16:1) | 0.221540282 |
| L-Leucine | 0.220058157 |
| TG(48:0-14:0) | 0.213083763 |
| TG(53:3-16:0) | -0.212555746 |
| 7-Methylguanine | 0.210546178 |
| Dihydrothymine | -0.20969269 |
| Glyoxylic acid | -0.209291845 |
| TG(54:4-16:1) | 0.208969182 |
| Butyric acid | -0.202765452 |
| Ornithine | -0.202113676 |
| 3-Amino-2-piperidone | -0.201695689 |
| Indole-3-methyl acetate | -0.194844983 |
| L-Aspartic acid | 0.192287057 |
| PC-O(36:2) | 0.183040591 |
| Ethylmalonic Acid | -0.18237625 |
| SM(42:1) | -0.162799053 |
| Oleoylethanolamide | -0.162249967 |
| 3-Hydroxymyristic acid | 0.161830499 |
| Deoxycholic acid 3-sulfate | 0.152109896 |
| TG(52:4-18:3) | 0.143833552 |
| PE(p18:0/20:3) | -0.143323115 |
| Vaccenic acid | 0.140519364 |
| Arachidonic acid | 0.133112953 |
| N-Acetylcadaverine | -0.133059416 |
| PE(p16:0/22:5) | 0.12418838 |
| 3-Hydroxybutyric acid | 0.12247175 |
| SM(42:3) | -0.122401855 |
| Taurolithocholic acid 3-sulfate | -0.120245254 |
| Pantothenic acid | -0.118680996 |
| PE(34:3) | 0.113822286 |
| Adipoylcarnitine (AcCa(6:0-DC)) | 0.113711977 |
| CE(22:6) | 0.112576883 |
| TG(40:0-14:0) | 0.110513354 |
| Biliverdin | -0.100326432 |
| 3-hydroxydodecanoic acid | -0.09497206 |
| L-Isoleucine | 0.094803706 |
| PI(16:0/18:3) | 0.094512631 |
| N-Acetyl-beta-alanine | -0.094352455 |
| Stearic acid | -0.087811688 |
| 3-Hydroxyhippuric acid | 0.087173025 |
| D-Mannose | 0.086436056 |
| PE(p18:0/22:5) | 0.083623423 |
| 3R-hydroxydecanoic acid | 0.081933635 |
| SM(40:5) | 0.0808975 |
| Taurine | 0.078211462 |
| TG(46:0-16:0) | -0.076900376 |
| Alpha-ketoisovaleric acid | 0.069875396 |
| Caprylic acid(FFA(8:0) | 0.069760308 |
| TG(50:1-18:0) | 0.065411821 |
| Taurocholic acid | -0.062774386 |
| 3-Aminobutanoic acid | -0.061700951 |
| PE-O(36:2) | 0.056041574 |
| PS(18:0/20:0) | -0.053612614 |
| Hydroxy-hexadecenoylcarnitine (AcCa(16:1-OH)) | -0.053383973 |
| 2-Hydroxycaproic acid | 0.052480096 |
| 4-hydroxyphenyllactate | 0.049773421 |
| Ribonic acid | 0.047342292 |
| 2-Hydroxy-2-methylbutyric acid | -0.046771475 |
| CE(18:3) | 0.045137242 |
| Glycodeoxycholic acid 3-sulfate | -0.031673626 |
| TG(54:8-18:3) | -0.031268834 |
| Hyocholic acid | 0.027875251 |
| PE(p18:0/20:5) | -0.021537958 |
| N2,N2-Dimethylguanosine | -0.019645941 |
| Glycolate | -0.01948419 |
| p-Cresol sulfate | 0.018196097 |
| PG(18:1/16:1) | -0.017778408 |
| PE-O(38:6) | -0.010269755 |
| Docosadienoate(22:2n6) | -0.008628947 |
| TG(50:2-16:1) | 0.007791467 |
| SM(42:6) | 0.007501032 |
| TG(47:0-14:0) | 0.006102841 |
| TG(52:3-18:2) | 0.005636258 |
| Caproic acid | 0.003987998 |
| Leucyl-leucine | -0.000673553 |
| Cytidine | -0.00025658 |
| **LASSO Proteins** | **coefficients** |
| SELENOP | 0.367003462 |
| MAN2A2 | -0.288268644 |
| PRKAR1A | -0.195620722 |
| FGFR4 | -0.193615743 |
| GPC1 | 0.163531218 |
| TATDN3 | 0.160960887 |
| DKK3 | 0.15864697 |
| IDS | 0.135749678 |
| SIGLEC14 | -0.134648182 |
| ANGPTL6 | 0.116731246 |
| PRSS2 | 0.115711943 |
| TFRC | -0.114798962 |
| PRSS3 | -0.089401112 |
| ADAMTS13 | 0.080451417 |
| TGM3 | -0.072095493 |
| SUSD5 | -0.065515089 |
| LRRN4 | -0.052509718 |
| H2AZ1;H2AZ2 | 0.052361867 |
| ARHGAP18 | -0.048193982 |
| SPON2 | -0.047041359 |
| EXT2 | 0.046709178 |
| PDCD1LG2 | -0.039624543 |
| LILRB1 | 0.036916277 |
| LEAP2 | 0.034611735 |
| CAVIN2 | 0.03205342 |
| NELL2 | -0.031959784 |
| GGT7 | 0.029463924 |
| PLXNA1 | -0.028941422 |
| DNAH17 | -0.026914023 |
| HNRNPK | -0.026218349 |
| GKN1 | -0.025610361 |
| REG1A | -0.024155055 |
| PKHD1L1 | 0.023632914 |
| HBG2 | 0.023349884 |
| FCGR2A | -0.022921895 |
| IGHV3-43 | 0.022651654 |
| TAGLN | 0.022141879 |
| SERPINF1 | -0.021073233 |
| HAGH | -0.020893564 |
| PSME2 | 0.019088307 |
| ANGPTL3 | 0.014527796 |
| VASP | 0.012414234 |
| SCARF1 | 0.011832084 |
| APOA4 | -0.010092881 |
| CD48 | -0.009704301 |
| IGKV1-12;IGKV1D-12 | -0.003044492 |
| Note: All listed metabolites and proteins have non-zero LASSO coefficients. Features are ranked by absolute coefficient values. | |

| Supplementary Table S2. Performance Metrics of machine learning models | | |
| --- | --- | --- |
| Variables | Mean | 95% CI |
| **Proteomic models** |  |  |
| AUC | 0.923 | [0.865, 0.980] |
| Accuracy | 0.862 | [0.835, 0.889] |
| Sensitivity | 0.834 | [0.698, 0.970] |
| Specificity | 0.870 | [0.767, 0.972] |
| Balanced Accuracy | 0.852 | [0.787, 0.917] |
| **Metabolomic models** |  |  |
| AUC | 0.816 | [0.766, 0.866] |
| Accuracy | 0.761 | [0.689, 0.833] |
| Sensitivity | 0.758 | [0.674, 0.842] |
| Specificity | 0.772 | [0.647, 0.897] |
| Balanced Accuracy | 0.765 | [0.698, 0.832] |
| **Stacking models** |  |  |
| AUC | 0.941 | [0.902, 0.980] |
| Accuracy | 0.853 | [0.815, 0.891] |
| Sensitivity | 0.868 | [0.797, 0.938] |
| Specificity | 0.809 | [0.680, 0.939] |
| Balanced Accuracy | 0.839 | [0.777, 0.900] |
| AUC, area under the curve; CI, confidence interval. | | |

| Supplementary Table S3. Mass Spectrometry based proteins between treatment responders and non-responders in the multi-omics cohort | | | | | | |
| --- | --- | --- | --- | --- | --- | --- |
| **Proteins** | **T** | **dof** | ***P-*value** | **95% CI** | **Log2FC** | **FDR *P-value*** |
| IGLV4 | -1.009 | 189.461 | 0.314 | [-0.45, 0.15] | -0.016 | 0.892 |
| IGLV8 | 1.507 | 205.941 | 0.133 | [-0.11, 0.79] | 0.030 | 0.833 |
| IGLV4 | 0.157 | 200.392 | 0.875 | [-0.35, 0.41] | 0.003 | 0.973 |
| IGLV10 | -0.328 | 205.843 | 0.743 | [-0.41, 0.29] | -0.006 | 0.946 |
| IGLV7 | -0.332 | 202.173 | 0.740 | [-0.35, 0.25] | -0.005 | 0.946 |
| IGLV3 | -0.097 | 176.606 | 0.923 | [-0.29, 0.26] | -0.001 | 0.979 |
| IGLV2 | -0.001 | 190.615 | 0.999 | [-0.28, 0.28] | 0.000 | 1.000 |
| IGLV3 | -0.431 | 197.762 | 0.667 | [-0.32, 0.2] | -0.006 | 0.941 |
| IGLV3 | -0.281 | 205.721 | 0.779 | [-0.3, 0.23] | -0.003 | 0.954 |
| IGLV3 | 0.847 | 203.368 | 0.398 | [-0.15, 0.37] | 0.010 | 0.917 |
| IGLV4 | -1.023 | 189.530 | 0.307 | [-0.31, 0.1] | -0.013 | 0.892 |
| IGKV2 | -0.198 | 197.213 | 0.843 | [-0.39, 0.32] | -0.004 | 0.971 |
| IGHV3 | -0.158 | 198.827 | 0.874 | [-0.29, 0.25] | -0.002 | 0.973 |
| IGHV4 | 0.488 | 202.780 | 0.626 | [-0.25, 0.41] | 0.008 | 0.935 |
| IGKV2D | 0.672 | 198.854 | 0.502 | [-0.16, 0.32] | 0.009 | 0.921 |
| IGKV1 | 0.070 | 188.665 | 0.945 | [-0.27, 0.29] | 0.001 | 0.988 |
| IGKV2D | -1.369 | 164.343 | 0.173 | [-0.56, 0.1] | -0.023 | 0.857 |
| IGLV5 | -0.024 | 205.888 | 0.981 | [-0.32, 0.31] | 0.000 | 0.998 |
| IGKV3D | 0.230 | 200.479 | 0.818 | [-0.28, 0.36] | 0.003 | 0.966 |
| IGKV1D | -1.968 | 171.864 | 0.051 | [-0.69, 0] | -0.040 | 0.808 |
| IGKV2 | 0.443 | 201.078 | 0.659 | [-0.22, 0.35] | 0.007 | 0.937 |
| IGLV5 | -0.912 | 198.600 | 0.363 | [-0.3, 0.11] | -0.012 | 0.913 |
| IGHV1 | -1.224 | 183.412 | 0.222 | [-0.46, 0.11] | -0.020 | 0.885 |
| IGHV3 | 0.220 | 196.425 | 0.826 | [-0.27, 0.34] | 0.003 | 0.970 |
| IGKV6D | 1.315 | 202.546 | 0.190 | [-0.11, 0.57] | 0.023 | 0.870 |
| IGLV1 | 0.688 | 184.586 | 0.492 | [-0.14, 0.28] | 0.008 | 0.918 |
| IGHV6 | 0.336 | 203.161 | 0.737 | [-0.23, 0.33] | 0.004 | 0.946 |
| IGHV3 | 0.132 | 203.019 | 0.895 | [-0.2, 0.23] | 0.001 | 0.976 |
| IGHV3 | 1.203 | 194.020 | 0.230 | [-0.11, 0.47] | 0.020 | 0.885 |
| IGHV2 | -0.877 | 172.746 | 0.382 | [-0.33, 0.13] | -0.010 | 0.917 |
| IGHV3 | 0.079 | 205.991 | 0.937 | [-0.3, 0.32] | 0.001 | 0.984 |
| IGHV3 | -0.166 | 206.000 | 0.868 | [-0.31, 0.27] | -0.002 | 0.972 |
| IGHV3 | 2.567 | 200.291 | 0.011 | [0.11, 0.83] | 0.048 | 0.740 |
| IGLV9 | 0.464 | 204.577 | 0.643 | [-0.22, 0.36] | 0.007 | 0.935 |
| IGHV3 | -0.314 | 202.146 | 0.754 | [-0.35, 0.25] | -0.004 | 0.951 |
| IGKV1D | 0.194 | 194.227 | 0.846 | [-0.27, 0.33] | 0.003 | 0.971 |
| IGKV6 | -0.970 | 185.727 | 0.333 | [-0.65, 0.22] | -0.022 | 0.892 |
| IGKV3D | 0.382 | 204.268 | 0.703 | [-0.18, 0.27] | 0.004 | 0.946 |
| IGHV1 | -0.237 | 205.956 | 0.813 | [-0.29, 0.23] | -0.003 | 0.964 |
| IGHV1 | 0.268 | 204.655 | 0.789 | [-0.22, 0.29] | 0.004 | 0.954 |
| IGHV3 | -0.167 | 191.248 | 0.868 | [-0.23, 0.19] | -0.002 | 0.972 |
| IGHV1 | -0.351 | 179.698 | 0.726 | [-0.33, 0.23] | -0.006 | 0.946 |
| IGHV4 | -1.688 | 192.452 | 0.093 | [-0.57, 0.04] | -0.027 | 0.830 |
| IGHV5 | 0.585 | 205.957 | 0.559 | [-0.2, 0.36] | 0.007 | 0.925 |
| IGKV3D | -0.153 | 204.199 | 0.879 | [-0.31, 0.27] | -0.002 | 0.973 |
| IGKV1 | 0.377 | 203.776 | 0.707 | [-0.19, 0.28] | 0.004 | 0.946 |
| IGKV2 | 0.863 | 203.106 | 0.389 | [-0.19, 0.49] | 0.013 | 0.917 |
| IGKV1 | -0.836 | 205.854 | 0.404 | [-0.38, 0.16] | -0.010 | 0.917 |
| IGLV5 | -1.327 | 195.155 | 0.186 | [-0.4, 0.08] | -0.015 | 0.863 |
| IGHV7 | -1.626 | 165.613 | 0.106 | [-0.34, 0.03] | -0.016 | 0.830 |
| IGHV3 | 0.585 | 205.964 | 0.559 | [-0.19, 0.36] | 0.008 | 0.925 |
| IGHV5 | 0.427 | 204.099 | 0.670 | [-0.42, 0.65] | 0.011 | 0.941 |
| IGLC7 | 0.118 | 205.263 | 0.906 | [-0.28, 0.32] | 0.001 | 0.976 |
| SSC5D | 0.612 | 205.340 | 0.541 | [-0.1, 0.19] | 0.005 | 0.924 |
| IGKV2 | -0.548 | 205.698 | 0.584 | [-0.43, 0.25] | -0.007 | 0.930 |
| VWA3A | 0.390 | 202.960 | 0.697 | [-0.12, 0.18] | 0.003 | 0.946 |
| C2orf78 | -1.462 | 179.594 | 0.146 | [-0.5, 0.07] | -0.022 | 0.833 |
| GGT3P | 0.367 | 192.994 | 0.714 | [-0.19, 0.28] | 0.006 | 0.946 |
| GLTPD2 | 0.539 | 205.959 | 0.590 | [-0.08, 0.14] | 0.004 | 0.930 |
| TMEM212 | 0.212 | 201.931 | 0.832 | [-0.27, 0.33] | 0.003 | 0.971 |
| IGLL5 | 0.752 | 205.612 | 0.453 | [-0.16, 0.37] | 0.006 | 0.918 |
| TK2 | 0.804 | 203.096 | 0.422 | [-0.1, 0.25] | 0.007 | 0.918 |
| DDX39A | 1.970 | 204.570 | 0.050 | [-0., 0.4] | 0.022 | 0.808 |
| PDLIM1 | 1.164 | 193.336 | 0.246 | [-0.19, 0.73] | 0.028 | 0.885 |
| MYO1F | -0.304 | 192.634 | 0.761 | [-0.23, 0.17] | -0.004 | 0.952 |
| MASP2 | -0.320 | 196.645 | 0.749 | [-0.13, 0.1] | -0.001 | 0.951 |
| RAB27B | 0.339 | 165.094 | 0.735 | [-0.2, 0.28] | 0.005 | 0.946 |
| PSMD9 | -0.067 | 161.477 | 0.946 | [-0.24, 0.22] | -0.001 | 0.989 |
| CLIC1 | -1.147 | 196.030 | 0.253 | [-0.86, 0.23] | -0.033 | 0.885 |
| QSOX1 | -1.743 | 204.088 | 0.083 | [-0.11, 0.01] | -0.004 | 0.818 |
| DNM1L | 0.988 | 195.591 | 0.325 | [-0.19, 0.56] | 0.021 | 0.892 |
| GFRA2 | 0.288 | 201.008 | 0.774 | [-0.14, 0.19] | 0.003 | 0.954 |
| MANBA | -0.699 | 186.928 | 0.486 | [-0.14, 0.07] | -0.004 | 0.918 |
| AGRN | -0.638 | 172.092 | 0.524 | [-0.2, 0.1] | -0.006 | 0.924 |
| CHL1 | 0.702 | 203.266 | 0.484 | [-0.06, 0.13] | 0.003 | 0.918 |
| PES1 | -0.532 | 203.318 | 0.595 | [-0.15, 0.08] | -0.004 | 0.930 |
| RNASET2 | 0.117 | 205.874 | 0.907 | [-0.15, 0.17] | 0.001 | 0.976 |
| CCL21 | -0.176 | 205.219 | 0.860 | [-0.2, 0.17] | -0.002 | 0.971 |
| PODXL | -1.208 | 199.444 | 0.228 | [-0.26, 0.06] | -0.009 | 0.885 |
| FCN1 | -1.730 | 189.883 | 0.085 | [-0.51, 0.03] | -0.028 | 0.828 |
| CES2 | -0.711 | 200.814 | 0.478 | [-0.21, 0.1] | -0.008 | 0.918 |
| MAN2B1 | 0.335 | 189.627 | 0.738 | [-0.15, 0.21] | 0.004 | 0.946 |
| ISLR | -1.542 | 196.510 | 0.125 | [-0.27, 0.03] | -0.012 | 0.830 |
| MAP2K7 | 0.250 | 205.961 | 0.802 | [-0.29, 0.37] | 0.003 | 0.960 |
| NHERF1 | -0.083 | 188.316 | 0.934 | [-0.2, 0.18] | -0.001 | 0.983 |
| TPP1 | 1.188 | 173.848 | 0.236 | [-0.09, 0.36] | 0.018 | 0.885 |
| NRP1 | 0.788 | 205.801 | 0.431 | [-0.06, 0.15] | 0.004 | 0.918 |
| APOL1 | -0.546 | 205.232 | 0.586 | [-0.17, 0.1] | -0.003 | 0.930 |
| MSTN | -1.075 | 203.777 | 0.284 | [-0.29, 0.08] | -0.013 | 0.888 |
| PSMA7 | -0.943 | 204.233 | 0.347 | [-0.33, 0.12] | -0.013 | 0.897 |
| LECT2 | -0.916 | 193.912 | 0.361 | [-0.26, 0.09] | -0.009 | 0.912 |
| ARHGEF10 | -0.340 | 197.536 | 0.734 | [-0.19, 0.14] | -0.004 | 0.946 |
| PLXNB2 | -0.233 | 200.819 | 0.816 | [-0.14, 0.11] | -0.002 | 0.965 |
| PFAS | -1.003 | 194.455 | 0.317 | [-0.84, 0.27] | -0.026 | 0.892 |
| FYB1 | -0.382 | 205.197 | 0.703 | [-0.2, 0.13] | -0.004 | 0.946 |
| ARPC1B | -0.376 | 202.101 | 0.708 | [-0.47, 0.32] | -0.009 | 0.946 |
| ARPC2 | -0.824 | 195.220 | 0.411 | [-0.54, 0.22] | -0.019 | 0.918 |
| ARPC3 | -0.186 | 205.831 | 0.852 | [-0.36, 0.3] | -0.003 | 0.971 |
| ADAMDEC1 | -1.981 | 204.677 | 0.049 | [-0.27, 0] | -0.014 | 0.808 |
| LAMA5 | 0.694 | 169.601 | 0.489 | [-0.11, 0.22] | 0.008 | 0.918 |
| CHAD | -1.049 | 199.709 | 0.295 | [-0.31, 0.1] | -0.012 | 0.888 |
| NCAM2 | 0.649 | 204.676 | 0.517 | [-0.07, 0.13] | 0.003 | 0.924 |
| STX7 | 0.162 | 174.002 | 0.872 | [-0.17, 0.19] | 0.002 | 0.972 |
| CCL16 | -0.752 | 203.828 | 0.453 | [-0.34, 0.15] | -0.011 | 0.918 |
| ARPC5 | -0.966 | 198.913 | 0.335 | [-0.74, 0.25] | -0.026 | 0.892 |
| PLXNB1 | -0.724 | 179.427 | 0.470 | [-0.12, 0.06] | -0.003 | 0.918 |
| SPINT1 | -1.230 | 205.965 | 0.220 | [-0.24, 0.06] | -0.012 | 0.885 |
| TXNL1 | -0.253 | 203.487 | 0.801 | [-0.24, 0.19] | -0.003 | 0.960 |
| COCH | 0.657 | 205.466 | 0.512 | [-0.08, 0.16] | 0.005 | 0.924 |
| AKR7A2 | -1.835 | 196.422 | 0.068 | [-0.45, 0.02] | -0.026 | 0.818 |
| TGOLN2 | 0.567 | 204.904 | 0.572 | [-0.14, 0.25] | 0.008 | 0.926 |
| B4GAT1 | 0.500 | 202.780 | 0.618 | [-0.08, 0.13] | 0.003 | 0.935 |
| CHST10 | 0.452 | 203.195 | 0.652 | [-0.07, 0.12] | 0.003 | 0.935 |
| DNPH1 | -0.813 | 155.901 | 0.417 | [-0.23, 0.09] | -0.009 | 0.918 |
| RGS10 | -0.215 | 184.511 | 0.830 | [-0.23, 0.18] | -0.003 | 0.970 |
| ACTN4 | -0.641 | 165.686 | 0.522 | [-0.4, 0.2] | -0.012 | 0.924 |
| KRT86 | 0.356 | 193.499 | 0.722 | [-0.34, 0.49] | 0.008 | 0.946 |
| CD5L | 0.682 | 176.915 | 0.496 | [-0.11, 0.22] | 0.005 | 0.918 |
| XPNPEP2 | -0.764 | 201.446 | 0.446 | [-0.34, 0.15] | -0.010 | 0.918 |
| KALRN | -0.903 | 189.535 | 0.368 | [-0.24, 0.09] | -0.007 | 0.913 |
| GMFG | 0.195 | 202.033 | 0.846 | [-0.33, 0.4] | 0.004 | 0.971 |
| KIAA0513 | -0.506 | 190.263 | 0.614 | [-0.71, 0.42] | -0.013 | 0.935 |
| SUSD5 | -1.977 | 192.212 | 0.049 | [-0.45, 0] | -0.025 | 0.808 |
| NRP2 | -0.721 | 170.639 | 0.472 | [-0.16, 0.07] | -0.005 | 0.918 |
| MAN1A2 | -0.386 | 198.924 | 0.700 | [-0.3, 0.2] | -0.005 | 0.946 |
| SNX3 | 0.010 | 204.925 | 0.992 | [-0.16, 0.16] | 0.000 | 0.999 |
| PLIN3 | -2.162 | 198.660 | 0.032 | [-0.48, -0.02] | -0.032 | 0.808 |
| FCMR | -0.252 | 186.807 | 0.801 | [-0.24, 0.18] | -0.003 | 0.960 |
| H2BC12 | 0.609 | 198.119 | 0.543 | [-0.17, 0.31] | 0.009 | 0.924 |
| ZG16 | -1.031 | 200.311 | 0.304 | [-0.38, 0.12] | -0.016 | 0.892 |
| CUTA | -1.761 | 195.463 | 0.080 | [-0.41, 0.02] | -0.020 | 0.818 |
| FCGR3B | -1.855 | 168.882 | 0.065 | [-0.47, 0.01] | -0.023 | 0.818 |
| LILRB5 | 0.045 | 205.289 | 0.964 | [-0.13, 0.13] | 0.000 | 0.997 |
| WDR1 | -0.726 | 204.148 | 0.469 | [-0.74, 0.34] | -0.022 | 0.918 |
| CPNE3 | -0.880 | 193.585 | 0.380 | [-0.24, 0.09] | -0.010 | 0.917 |
| ICOSLG | -1.675 | 182.651 | 0.096 | [-0.19, 0.02] | -0.008 | 0.830 |
| GGCT | 0.882 | 203.841 | 0.379 | [-0.16, 0.41] | 0.016 | 0.917 |
| SEMA7A | -1.463 | 205.099 | 0.145 | [-0.31, 0.05] | -0.017 | 0.833 |
| CILP | -0.801 | 204.224 | 0.424 | [-0.32, 0.13] | -0.010 | 0.918 |
| PDCD6 | -2.422 | 182.931 | 0.016 | [-0.49, -0.05] | -0.035 | 0.740 |
| TBCA | 0.032 | 200.619 | 0.975 | [-0.3, 0.31] | 0.001 | 0.997 |
| ENTPD5 | 0.560 | 196.730 | 0.576 | [-0.1, 0.18] | 0.005 | 0.928 |
| SH3BGRL | -0.398 | 204.729 | 0.691 | [-0.5, 0.33] | -0.009 | 0.946 |
| TNFRSF21 | -1.139 | 150.602 | 0.256 | [-0.46, 0.12] | -0.022 | 0.885 |
| TAF5L | -1.320 | 173.458 | 0.188 | [-0.26, 0.05] | -0.012 | 0.867 |
| PGLYRP1 | 0.843 | 193.513 | 0.400 | [-0.11, 0.27] | 0.009 | 0.917 |
| LYPLA1 | -2.005 | 186.519 | 0.046 | [-0.44, 0] | -0.026 | 0.808 |
| FCN3 | 1.232 | 199.513 | 0.219 | [-0.12, 0.54] | 0.016 | 0.885 |
| GRAP2 | -0.177 | 162.102 | 0.860 | [-0.19, 0.16] | -0.002 | 0.971 |
| IDH1 | -1.650 | 198.939 | 0.101 | [-0.49, 0.04] | -0.024 | 0.830 |
| ATRN | 1.137 | 204.609 | 0.257 | [-0.03, 0.09] | 0.003 | 0.885 |
| CPD | -0.433 | 169.403 | 0.666 | [-0.16, 0.11] | -0.004 | 0.940 |
| STC2 | -1.107 | 202.657 | 0.269 | [-0.23, 0.07] | -0.010 | 0.888 |
| ECM2 | 0.156 | 205.899 | 0.876 | [-0.1, 0.11] | 0.001 | 0.973 |
| CLSTN1 | 1.166 | 205.738 | 0.245 | [-0.04, 0.14] | 0.005 | 0.885 |
| APOL3 | -0.697 | 205.836 | 0.487 | [-0.26, 0.12] | -0.007 | 0.918 |
| ABCC6 | -1.363 | 205.563 | 0.175 | [-0.25, 0.05] | -0.011 | 0.857 |
| PGLS | -0.752 | 199.779 | 0.453 | [-0.41, 0.18] | -0.014 | 0.918 |
| PAPLN | 0.313 | 201.738 | 0.755 | [-0.16, 0.22] | 0.004 | 0.952 |
| APOM | -0.362 | 205.709 | 0.717 | [-0.2, 0.13] | -0.002 | 0.946 |
| H6PD | -2.006 | 204.595 | 0.046 | [-0.27, 0] | -0.013 | 0.808 |
| VNN1 | -0.778 | 199.265 | 0.437 | [-0.44, 0.19] | -0.011 | 0.918 |
| FSTL3 | 0.497 | 203.888 | 0.620 | [-0.11, 0.19] | 0.005 | 0.935 |
| CRTAM | -1.168 | 179.924 | 0.245 | [-0.24, 0.06] | -0.011 | 0.885 |
| CAVIN2 | 1.665 | 188.562 | 0.098 | [-0.05, 0.59] | 0.028 | 0.830 |
| EML2 | -0.990 | 177.240 | 0.323 | [-0.26, 0.09] | -0.012 | 0.892 |
| OLFM2 | -0.347 | 203.302 | 0.729 | [-0.21, 0.14] | -0.003 | 0.946 |
| RECK | 1.790 | 202.530 | 0.075 | [-0.01, 0.2] | 0.011 | 0.818 |
| ADH1B | -1.117 | 204.059 | 0.265 | [-0.44, 0.12] | -0.018 | 0.886 |
| ADH1C | -0.467 | 194.458 | 0.641 | [-0.46, 0.29] | -0.011 | 0.935 |
| LDHA | -1.905 | 203.373 | 0.058 | [-0.62, 0.01] | -0.027 | 0.811 |
| ALDH1A1 | -1.922 | 187.918 | 0.056 | [-0.56, 0.01] | -0.030 | 0.810 |
| GLUD1 | -1.133 | 205.873 | 0.258 | [-0.33, 0.09] | -0.017 | 0.885 |
| GSR | -1.274 | 175.241 | 0.204 | [-0.22, 0.05] | -0.009 | 0.885 |
| SOD1 | 0.272 | 194.260 | 0.786 | [-0.21, 0.28] | 0.003 | 0.954 |
| CP | 1.452 | 199.422 | 0.148 | [-0.02, 0.12] | 0.003 | 0.833 |
| F8 | 0.123 | 204.110 | 0.902 | [-0.11, 0.13] | 0.001 | 0.976 |
| OTC | -1.223 | 205.121 | 0.223 | [-0.39, 0.09] | -0.018 | 0.885 |
| F13A1 | -0.684 | 168.536 | 0.495 | [-0.21, 0.1] | -0.004 | 0.918 |
| PNP | -0.407 | 202.286 | 0.685 | [-0.46, 0.3] | -0.008 | 0.946 |
| HPRT1 | -0.488 | 205.364 | 0.626 | [-0.3, 0.18] | -0.007 | 0.935 |
| EGFR | 0.751 | 202.073 | 0.453 | [-0.04, 0.09] | 0.002 | 0.918 |
| PGK1 | -0.552 | 200.724 | 0.581 | [-0.45, 0.25] | -0.010 | 0.930 |
| AK1 | -1.459 | 182.179 | 0.146 | [-0.55, 0.08] | -0.026 | 0.833 |
| LALBA | -0.752 | 202.992 | 0.453 | [-0.44, 0.2] | -0.011 | 0.918 |
| F2 | 2.038 | 183.873 | 0.043 | [0., 0.09] | 0.003 | 0.808 |
| C1R | -0.969 | 205.349 | 0.333 | [-0.08, 0.03] | -0.002 | 0.892 |
| HP | -0.523 | 195.646 | 0.602 | [-0.48, 0.28] | -0.006 | 0.932 |
| HPR | -0.535 | 205.900 | 0.593 | [-0.26, 0.15] | -0.005 | 0.930 |
| F9 | -1.446 | 187.201 | 0.150 | [-0.1, 0.02] | -0.003 | 0.833 |
| F10 | 0.538 | 191.649 | 0.591 | [-0.05, 0.09] | 0.002 | 0.930 |
| CFD | -0.698 | 205.844 | 0.486 | [-0.13, 0.06] | -0.003 | 0.918 |
| PLG | 0.955 | 205.892 | 0.341 | [-0.02, 0.07] | 0.001 | 0.897 |
| F12 | -0.406 | 205.548 | 0.685 | [-0.19, 0.13] | -0.002 | 0.946 |
| CFB | -0.007 | 200.268 | 0.994 | [-0.08, 0.08] | 0.000 | 0.999 |
| CA1 | -0.247 | 177.205 | 0.806 | [-0.33, 0.26] | -0.003 | 0.962 |
| CA2 | 0.185 | 184.774 | 0.853 | [-0.26, 0.31] | 0.002 | 0.971 |
| ASS1 | -1.897 | 205.926 | 0.059 | [-0.44, 0.01] | -0.026 | 0.811 |
| SPINK1 | -0.177 | 199.279 | 0.859 | [-0.22, 0.18] | -0.002 | 0.971 |
| SERPINC1 | 2.170 | 199.017 | 0.031 | [0.01, 0.11] | 0.003 | 0.808 |
| SERPINA1 | -1.255 | 203.623 | 0.211 | [-0.48, 0.11] | -0.012 | 0.885 |
| SERPINA3 | 0.585 | 205.665 | 0.559 | [-0.07, 0.13] | 0.002 | 0.925 |
| AGT | 0.008 | 197.678 | 0.994 | [-0.09, 0.09] | 0.000 | 0.999 |
| A2M | -0.489 | 205.875 | 0.625 | [-0.33, 0.2] | -0.004 | 0.935 |
| C3 | 0.137 | 204.531 | 0.891 | [-0.16, 0.18] | 0.001 | 0.976 |
| C5 | -2.717 | 161.351 | 0.007 | [-0.16, -0.03] | -0.006 | 0.705 |
| TIMP1 | 0.544 | 199.216 | 0.587 | [-0.08, 0.14] | 0.003 | 0.930 |
| CST3 | -0.875 | 205.250 | 0.382 | [-0.11, 0.04] | -0.003 | 0.917 |
| CSTA | 0.166 | 203.499 | 0.868 | [-0.25, 0.3] | 0.003 | 0.972 |
| KNG1 | -0.174 | 190.772 | 0.862 | [-0.06, 0.05] | 0.000 | 0.971 |
| LDLR | -0.966 | 188.103 | 0.335 | [-0.33, 0.11] | -0.012 | 0.892 |
| TGFB1 | 1.028 | 199.565 | 0.305 | [-0.13, 0.43] | 0.018 | 0.892 |
| PRL | 1.359 | 191.218 | 0.176 | [-0.11, 0.6] | 0.028 | 0.857 |
| IGF2 | 0.054 | 195.166 | 0.957 | [-0.07, 0.07] | 0.000 | 0.995 |
| JCHAIN | 0.461 | 198.368 | 0.646 | [-0.22, 0.35] | 0.005 | 0.935 |
| IGKV1D | -0.392 | 204.763 | 0.695 | [-0.34, 0.23] | -0.005 | 0.946 |
| IGKV1 | -0.639 | 204.727 | 0.524 | [-0.3, 0.15] | -0.007 | 0.924 |
| IGKV1 | -0.638 | 205.509 | 0.524 | [-0.44, 0.22] | -0.010 | 0.924 |
| IGKV1D | 0.276 | 205.905 | 0.783 | [-0.2, 0.26] | 0.003 | 0.954 |
| IGKV1 | 0.572 | 199.065 | 0.568 | [-0.21, 0.38] | 0.007 | 0.926 |
| IGKV3 | -0.427 | 205.011 | 0.670 | [-0.33, 0.21] | -0.004 | 0.941 |
| IGLV1 | -1.017 | 189.459 | 0.311 | [-0.36, 0.12] | -0.012 | 0.892 |
| IGLV1 | 0.171 | 205.976 | 0.864 | [-0.23, 0.28] | 0.002 | 0.971 |
| IGLV1 | -0.116 | 205.956 | 0.908 | [-0.3, 0.27] | -0.001 | 0.976 |
| IGLV1 | -1.572 | 192.382 | 0.118 | [-0.56, 0.06] | -0.023 | 0.830 |
| IGLV2 | -0.892 | 193.899 | 0.374 | [-0.51, 0.19] | -0.015 | 0.917 |
| IGLV2 | -1.653 | 186.762 | 0.100 | [-0.53, 0.05] | -0.024 | 0.830 |
| IGLV2 | 0.334 | 200.928 | 0.739 | [-0.23, 0.33] | 0.004 | 0.946 |
| IGLV2 | -2.141 | 199.071 | 0.033 | [-0.64, -0.03] | -0.031 | 0.808 |
| IGLV3 | -0.435 | 194.174 | 0.664 | [-0.35, 0.22] | -0.006 | 0.939 |
| IGLV3 | -1.495 | 202.531 | 0.137 | [-0.34, 0.05] | -0.018 | 0.833 |
| IGLV3 | 0.419 | 201.673 | 0.676 | [-0.19, 0.3] | 0.005 | 0.946 |
| IGLV3 | -0.009 | 196.883 | 0.993 | [-0.27, 0.27] | 0.000 | 0.999 |
| IGLV6 | 1.367 | 205.973 | 0.173 | [-0.09, 0.48] | 0.019 | 0.857 |
| IGHV1 | -0.233 | 205.999 | 0.816 | [-0.3, 0.24] | -0.003 | 0.965 |
| IGHV1 | -0.125 | 195.192 | 0.901 | [-0.3, 0.27] | -0.002 | 0.976 |
| IGHV3 | -0.617 | 198.864 | 0.538 | [-0.32, 0.17] | -0.006 | 0.924 |
| IGHV3 | -0.616 | 148.489 | 0.539 | [-0.17, 0.09] | -0.004 | 0.924 |
| IGHV3 | -0.148 | 182.796 | 0.883 | [-0.16, 0.14] | -0.001 | 0.976 |
| IGHV3 | 1.044 | 205.681 | 0.298 | [-0.17, 0.55] | 0.018 | 0.888 |
| IGHV3 | 0.022 | 201.487 | 0.983 | [-0.28, 0.29] | 0.000 | 0.998 |
| IGHV3 | -0.375 | 199.122 | 0.708 | [-0.36, 0.25] | -0.005 | 0.946 |
| IGHV3 | -0.825 | 206.000 | 0.410 | [-0.39, 0.16] | -0.010 | 0.918 |
| IGHV2 | -0.104 | 205.997 | 0.917 | [-0.22, 0.2] | -0.001 | 0.979 |
| IGHV2 | -0.519 | 200.258 | 0.604 | [-0.35, 0.2] | -0.007 | 0.934 |
| PIGR | -0.070 | 195.568 | 0.944 | [-0.22, 0.21] | -0.001 | 0.988 |
| IGKC | 0.331 | 205.631 | 0.741 | [-0.24, 0.33] | 0.003 | 0.946 |
| IGHE | -0.360 | 203.698 | 0.719 | [-0.31, 0.22] | -0.006 | 0.946 |
| IGHG1 | 0.311 | 192.361 | 0.756 | [-0.3, 0.41] | 0.004 | 0.952 |
| IGHG2 | -0.113 | 203.455 | 0.910 | [-0.36, 0.32] | -0.001 | 0.977 |
| IGHG3 | 0.993 | 205.769 | 0.322 | [-0.15, 0.46] | 0.009 | 0.892 |
| IGHG4 | 0.975 | 196.246 | 0.331 | [-0.2, 0.61] | 0.016 | 0.892 |
| IGHM | 0.610 | 197.950 | 0.543 | [-0.21, 0.39] | 0.006 | 0.924 |
| IGHA1 | 1.074 | 205.550 | 0.284 | [-0.12, 0.42] | 0.010 | 0.888 |
| IGHA2 | 1.580 | 205.601 | 0.116 | [-0.06, 0.51] | 0.015 | 0.830 |
| IGHD | 1.150 | 204.998 | 0.252 | [-0.25, 0.96] | 0.033 | 0.885 |
| HLA | -1.806 | 191.104 | 0.072 | [-0.59, 0.03] | -0.033 | 0.818 |
| HBD | -0.286 | 178.777 | 0.775 | [-0.5, 0.38] | -0.006 | 0.954 |
| MB | -0.947 | 165.973 | 0.345 | [-0.35, 0.12] | -0.013 | 0.897 |
| COL1A1 | 0.597 | 202.898 | 0.551 | [-0.12, 0.23] | 0.005 | 0.925 |
| COL3A1 | 1.222 | 205.974 | 0.223 | [-0.07, 0.3] | 0.014 | 0.885 |
| KRT14 | 1.131 | 201.881 | 0.259 | [-0.16, 0.61] | 0.019 | 0.885 |
| KRT6A | -0.331 | 205.481 | 0.741 | [-0.4, 0.28] | -0.006 | 0.946 |
| SPTA1 | -0.012 | 205.066 | 0.990 | [-0.25, 0.25] | 0.000 | 0.999 |
| APOA1 | 0.413 | 203.345 | 0.680 | [-0.16, 0.25] | 0.003 | 0.946 |
| APOE | 0.052 | 195.649 | 0.959 | [-0.15, 0.16] | 0.000 | 0.995 |
| APOA2 | -1.381 | 205.968 | 0.169 | [-0.44, 0.08] | -0.012 | 0.857 |
| APOC1 | -0.650 | 201.144 | 0.517 | [-0.24, 0.12] | -0.004 | 0.924 |
| APOC2 | -0.267 | 196.121 | 0.790 | [-0.33, 0.25] | -0.003 | 0.954 |
| APOC3 | -0.771 | 194.909 | 0.441 | [-0.32, 0.14] | -0.006 | 0.918 |
| FGA | -0.485 | 169.440 | 0.628 | [-0.19, 0.11] | -0.003 | 0.935 |
| FGB | -0.533 | 149.823 | 0.595 | [-0.22, 0.12] | -0.003 | 0.930 |
| FGG | -1.074 | 151.303 | 0.285 | [-0.28, 0.08] | -0.006 | 0.888 |
| CRP | -1.857 | 188.059 | 0.065 | [-1.23, 0.04] | -0.049 | 0.818 |
| APCS | -2.351 | 161.767 | 0.020 | [-0.32, -0.03] | -0.012 | 0.740 |
| C1QA | 0.858 | 203.653 | 0.392 | [-0.04, 0.1] | 0.002 | 0.917 |
| C1QB | -0.944 | 205.790 | 0.346 | [-0.09, 0.03] | -0.002 | 0.897 |
| C1QC | 0.436 | 190.496 | 0.663 | [-0.05, 0.07] | 0.001 | 0.938 |
| C9 | -1.188 | 200.541 | 0.236 | [-0.18, 0.04] | -0.004 | 0.885 |
| APOH | -0.538 | 205.943 | 0.591 | [-0.08, 0.05] | -0.001 | 0.930 |
| LRG1 | -1.095 | 205.269 | 0.275 | [-0.21, 0.06] | -0.005 | 0.888 |
| FN1 | 0.667 | 200.819 | 0.506 | [-0.17, 0.33] | 0.006 | 0.924 |
| RBP4 | -1.722 | 202.647 | 0.087 | [-0.2, 0.01] | -0.006 | 0.830 |
| AMBP | 0.654 | 199.232 | 0.514 | [-0.04, 0.08] | 0.001 | 0.924 |
| ORM1 | 1.527 | 196.093 | 0.128 | [-0.07, 0.53] | 0.016 | 0.833 |
| AHSG | 0.386 | 205.980 | 0.700 | [-0.06, 0.09] | 0.001 | 0.946 |
| TTR | 0.216 | 191.922 | 0.829 | [-0.14, 0.18] | 0.001 | 0.970 |
| ALB | 1.190 | 201.953 | 0.235 | [-0.15, 0.61] | 0.013 | 0.885 |
| GC | -0.067 | 191.768 | 0.947 | [-0.05, 0.05] | 0.000 | 0.989 |
| PPBP | 1.345 | 203.723 | 0.180 | [-0.12, 0.65] | 0.020 | 0.861 |
| PF4 | 2.212 | 197.677 | 0.028 | [0.05, 0.9] | 0.041 | 0.792 |
| TFRC | -1.968 | 205.382 | 0.050 | [-0.73, 0] | -0.035 | 0.808 |
| TF | -0.087 | 204.669 | 0.931 | [-0.25, 0.22] | -0.001 | 0.981 |
| LTF | 0.444 | 168.483 | 0.658 | [-0.16, 0.26] | 0.005 | 0.937 |
| HPX | 0.884 | 205.138 | 0.378 | [-0.04, 0.1] | 0.002 | 0.917 |
| FTL | -0.567 | 198.671 | 0.572 | [-0.59, 0.32] | -0.014 | 0.926 |
| FTH1 | 1.559 | 196.356 | 0.121 | [-0.05, 0.43] | 0.025 | 0.830 |
| PRH1; | -3.120 | 200.551 | 0.002 | [-0.6, -0.14] | -0.044 | 0.578 |
| ANG | -0.289 | 199.818 | 0.773 | [-0.11, 0.08] | -0.001 | 0.954 |
| F11 | -0.033 | 184.359 | 0.974 | [-0.08, 0.08] | 0.000 | 0.997 |
| KLKB1 | 0.649 | 187.824 | 0.517 | [-0.04, 0.08] | 0.001 | 0.924 |
| SLPI | 0.177 | 205.941 | 0.860 | [-0.09, 0.11] | 0.001 | 0.971 |
| C4BPA | -1.496 | 195.470 | 0.136 | [-0.31, 0.04] | -0.008 | 0.833 |
| VTN | 0.760 | 204.179 | 0.448 | [-0.07, 0.15] | 0.002 | 0.918 |
| CAT | -0.876 | 185.347 | 0.382 | [-0.35, 0.13] | -0.010 | 0.917 |
| PLA2G1B | 1.075 | 181.846 | 0.284 | [-0.08, 0.29] | 0.014 | 0.888 |
| FUCA1 | -1.856 | 181.271 | 0.065 | [-0.25, 0.01] | -0.012 | 0.818 |
| PROC | 1.552 | 197.402 | 0.122 | [-0.02, 0.15] | 0.006 | 0.830 |
| ALDOA | -0.354 | 203.728 | 0.723 | [-0.35, 0.24] | -0.005 | 0.946 |
| CSTB | 0.141 | 200.003 | 0.888 | [-0.17, 0.2] | 0.002 | 0.976 |
| ANXA1 | 1.726 | 205.422 | 0.086 | [-0.06, 0.89] | 0.061 | 0.829 |
| APOB | 0.306 | 200.103 | 0.760 | [-0.09, 0.12] | 0.001 | 0.952 |
| PRNP | -0.754 | 200.714 | 0.452 | [-0.29, 0.13] | -0.010 | 0.918 |
| SOD2 | -2.795 | 189.513 | 0.006 | [-0.44, -0.08] | -0.027 | 0.705 |
| LCAT | 0.458 | 204.572 | 0.647 | [-0.08, 0.13] | 0.002 | 0.935 |
| HRG | 1.974 | 205.675 | 0.050 | [0., 0.25] | 0.008 | 0.808 |
| IGLV7 | -0.171 | 199.713 | 0.864 | [-0.32, 0.26] | -0.002 | 0.971 |
| A1BG | -0.801 | 158.176 | 0.424 | [-0.08, 0.04] | -0.001 | 0.918 |
| HLA | 0.404 | 202.272 | 0.687 | [-0.22, 0.34] | 0.007 | 0.946 |
| KRT6B | 1.394 | 204.301 | 0.165 | [-0.13, 0.75] | 0.025 | 0.853 |
| KRT1 | 1.760 | 205.632 | 0.080 | [-0.04, 0.78] | 0.031 | 0.818 |
| VWF | 1.229 | 190.124 | 0.221 | [-0.06, 0.25] | 0.008 | 0.885 |
| SHBG | 0.119 | 202.308 | 0.906 | [-0.28, 0.32] | 0.001 | 0.976 |
| GAPDH | -0.808 | 198.463 | 0.420 | [-0.54, 0.23] | -0.015 | 0.918 |
| ASL | -0.107 | 189.301 | 0.915 | [-0.34, 0.3] | -0.002 | 0.979 |
| IGKV1 | -1.127 | 193.213 | 0.261 | [-0.38, 0.1] | -0.014 | 0.885 |
| IGKV3 | 0.100 | 205.884 | 0.921 | [-0.3, 0.33] | 0.001 | 0.979 |
| CAPNS1 | -0.350 | 197.958 | 0.726 | [-0.43, 0.3] | -0.008 | 0.946 |
| AMY1A | -0.883 | 205.484 | 0.378 | [-0.36, 0.14] | -0.013 | 0.917 |
| AMY2A | 1.067 | 198.726 | 0.287 | [-0.11, 0.36] | 0.014 | 0.888 |
| HSPB1 | 0.687 | 201.440 | 0.493 | [-0.19, 0.39] | 0.013 | 0.918 |
| IGF1 | 1.745 | 195.964 | 0.083 | [-0.02, 0.33] | 0.013 | 0.818 |
| ALDOB | -0.504 | 203.317 | 0.615 | [-0.43, 0.25] | -0.008 | 0.935 |
| APP | 0.781 | 196.816 | 0.436 | [-0.21, 0.49] | 0.015 | 0.918 |
| ARG1 | -0.300 | 205.736 | 0.765 | [-0.21, 0.15] | -0.003 | 0.954 |
| APOD | 0.342 | 201.712 | 0.733 | [-0.12, 0.17] | 0.002 | 0.946 |
| ITGB3 | -0.628 | 200.246 | 0.531 | [-0.28, 0.15] | -0.009 | 0.924 |
| ITGB2 | -0.315 | 203.070 | 0.753 | [-0.25, 0.18] | -0.004 | 0.951 |
| S100A8 | -0.364 | 192.334 | 0.716 | [-0.41, 0.28] | -0.006 | 0.946 |
| SERPINE1 | 0.270 | 194.610 | 0.788 | [-0.24, 0.32] | 0.004 | 0.954 |
| SERPINA5 | -1.790 | 190.844 | 0.075 | [-0.16, 0.01] | -0.006 | 0.818 |
| SERPING1 | -0.737 | 203.295 | 0.462 | [-0.07, 0.03] | -0.001 | 0.918 |
| CFI | -0.364 | 196.719 | 0.716 | [-0.08, 0.05] | -0.001 | 0.946 |
| F13B | 1.871 | 200.821 | 0.063 | [-0., 0.15] | 0.005 | 0.818 |
| MPO | -0.142 | 173.601 | 0.887 | [-0.2, 0.17] | -0.001 | 0.976 |
| ALPL | -1.439 | 205.841 | 0.152 | [-0.33, 0.05] | -0.017 | 0.833 |
| ICAM1 | -1.974 | 204.328 | 0.050 | [-0.19, 0] | -0.009 | 0.808 |
| REG1A | -1.742 | 203.721 | 0.083 | [-0.45, 0.03] | -0.020 | 0.818 |
| CLEC3B | 1.817 | 202.254 | 0.071 | [-0.01, 0.13] | 0.004 | 0.818 |
| HLA | -0.484 | 203.168 | 0.629 | [-0.32, 0.19] | -0.006 | 0.935 |
| SERPINA7 | -0.863 | 201.847 | 0.389 | [-0.12, 0.05] | -0.002 | 0.917 |
| SERPIND1 | -1.401 | 197.796 | 0.163 | [-0.13, 0.02] | -0.003 | 0.849 |
| ITGB1 | 0.032 | 205.825 | 0.975 | [-0.09, 0.09] | 0.000 | 0.997 |
| MYL1 | -0.588 | 190.250 | 0.557 | [-0.4, 0.22] | -0.013 | 0.925 |
| HPN | -0.715 | 155.851 | 0.475 | [-0.18, 0.08] | -0.007 | 0.918 |
| UROD | -0.353 | 170.254 | 0.725 | [-0.31, 0.22] | -0.006 | 0.946 |
| BCHE | 1.544 | 204.753 | 0.124 | [-0.02, 0.18] | 0.005 | 0.830 |
| GLA | -0.759 | 202.308 | 0.449 | [-0.13, 0.06] | -0.005 | 0.918 |
| IGKV4 | 0.357 | 205.967 | 0.721 | [-0.23, 0.33] | 0.004 | 0.946 |
| IGHV4 | -0.402 | 205.479 | 0.688 | [-0.33, 0.22] | -0.005 | 0.946 |
| GSN | -0.967 | 202.745 | 0.335 | [-0.12, 0.04] | -0.003 | 0.892 |
| C2 | 0.321 | 198.904 | 0.749 | [-0.05, 0.07] | 0.001 | 0.951 |
| S100A9 | -0.045 | 193.533 | 0.964 | [-0.33, 0.31] | -0.001 | 0.997 |
| S100A6 | 0.946 | 197.543 | 0.345 | [-0.15, 0.44] | 0.014 | 0.897 |
| APOA4 | -1.808 | 205.917 | 0.072 | [-0.3, 0.01] | -0.009 | 0.818 |
| CKM | -0.739 | 185.990 | 0.461 | [-0.52, 0.23] | -0.016 | 0.918 |
| ENO1 | -0.901 | 199.368 | 0.369 | [-0.54, 0.2] | -0.017 | 0.913 |
| PYGL | -0.307 | 199.495 | 0.759 | [-0.25, 0.19] | -0.004 | 0.952 |
| GPI | -0.175 | 198.910 | 0.861 | [-0.37, 0.31] | -0.003 | 0.971 |
| TPM3 | -0.238 | 200.758 | 0.812 | [-0.32, 0.25] | -0.004 | 0.964 |
| ITGAV | -0.209 | 199.049 | 0.835 | [-0.18, 0.15] | -0.002 | 0.971 |
| LPL | 0.624 | 175.606 | 0.533 | [-0.11, 0.22] | 0.007 | 0.924 |
| HEXA | -0.230 | 201.430 | 0.819 | [-0.12, 0.1] | -0.001 | 0.966 |
| SERPINE2 | -0.736 | 186.278 | 0.463 | [-0.38, 0.17] | -0.013 | 0.918 |
| DBI | 0.249 | 199.419 | 0.803 | [-0.23, 0.3] | 0.004 | 0.961 |
| FABP1 | -1.256 | 204.368 | 0.210 | [-0.43, 0.1] | -0.019 | 0.885 |
| LDHB | -1.181 | 204.525 | 0.239 | [-0.31, 0.08] | -0.011 | 0.885 |
| GPX1 | -1.402 | 197.913 | 0.163 | [-0.45, 0.08] | -0.022 | 0.849 |
| PROS1 | -1.084 | 197.142 | 0.279 | [-0.18, 0.05] | -0.004 | 0.888 |
| P4HB | -1.268 | 199.522 | 0.206 | [-0.29, 0.06] | -0.013 | 0.885 |
| ASGR2 | -0.474 | 204.349 | 0.636 | [-0.2, 0.12] | -0.004 | 0.935 |
| ADH1A | -1.484 | 200.950 | 0.139 | [-0.51, 0.07] | -0.027 | 0.833 |
| CSF1R | -0.790 | 202.822 | 0.430 | [-0.15, 0.06] | -0.004 | 0.918 |
| CTSD | 0.321 | 172.628 | 0.749 | [-0.12, 0.17] | 0.002 | 0.951 |
| ANXA2 | 0.475 | 205.955 | 0.635 | [-0.2, 0.32] | 0.009 | 0.935 |
| C8A | -0.535 | 193.989 | 0.594 | [-0.08, 0.05] | -0.001 | 0.930 |
| C8B | 0.102 | 194.460 | 0.919 | [-0.07, 0.07] | 0.000 | 0.979 |
| GP1BA | -0.014 | 205.549 | 0.989 | [-0.12, 0.12] | 0.000 | 0.999 |
| C8G | -0.644 | 171.364 | 0.520 | [-0.11, 0.05] | -0.002 | 0.924 |
| CAPN1 | -0.707 | 200.627 | 0.481 | [-0.48, 0.22] | -0.013 | 0.918 |
| TUBB | 0.556 | 199.854 | 0.579 | [-0.26, 0.47] | 0.012 | 0.930 |
| CA3 | 0.189 | 187.747 | 0.851 | [-0.24, 0.29] | 0.003 | 0.971 |
| PRSS1 | 1.945 | 203.898 | 0.053 | [-0.01, 1.15] | 0.056 | 0.808 |
| PRSS2 | 0.981 | 155.014 | 0.328 | [-0.27, 0.79] | 0.027 | 0.892 |
| DCN | -0.145 | 165.984 | 0.885 | [-0.12, 0.1] | -0.001 | 0.976 |
| PSAP | 1.871 | 205.395 | 0.063 | [-0.01, 0.3] | 0.016 | 0.818 |
| HEXB | -0.005 | 192.775 | 0.996 | [-0.14, 0.14] | 0.000 | 0.999 |
| CTSL | 0.165 | 196.964 | 0.869 | [-0.14, 0.17] | 0.001 | 0.972 |
| PFN1 | -0.158 | 202.928 | 0.875 | [-0.67, 0.57] | -0.004 | 0.973 |
| BPGM | -0.347 | 184.134 | 0.729 | [-0.29, 0.21] | -0.004 | 0.946 |
| CTSB | 0.201 | 181.358 | 0.841 | [-0.14, 0.17] | 0.002 | 0.971 |
| HSP90AA1 | -0.332 | 204.185 | 0.740 | [-0.43, 0.31] | -0.007 | 0.946 |
| UMOD | 2.377 | 206.000 | 0.018 | [0.03, 0.36] | 0.019 | 0.740 |
| LAMB1 | 1.403 | 204.140 | 0.162 | [-0.03, 0.16] | 0.007 | 0.849 |
| TPM2 | -2.290 | 201.279 | 0.023 | [-0.52, -0.04] | -0.031 | 0.740 |
| THBS1 | 1.812 | 200.290 | 0.072 | [-0.03, 0.73] | 0.028 | 0.818 |
| RNASE1 | 0.330 | 202.901 | 0.741 | [-0.1, 0.14] | 0.002 | 0.946 |
| COL1A2 | 0.040 | 198.429 | 0.968 | [-0.15, 0.16] | 0.000 | 0.997 |
| ANXA6 | -0.458 | 200.783 | 0.647 | [-0.34, 0.21] | -0.009 | 0.935 |
| CD55 | -0.755 | 203.823 | 0.451 | [-0.19, 0.08] | -0.006 | 0.918 |
| SERPINA6 | 1.763 | 203.645 | 0.079 | [-0.01, 0.13] | 0.004 | 0.818 |
| SLC3A2 | -0.639 | 197.550 | 0.523 | [-0.11, 0.06] | -0.003 | 0.924 |
| GUSB | 0.182 | 183.865 | 0.856 | [-0.2, 0.24] | 0.003 | 0.971 |
| HSP90AB1 | -0.793 | 200.919 | 0.429 | [-0.47, 0.2] | -0.014 | 0.918 |
| ELANE | 0.320 | 175.403 | 0.750 | [-0.13, 0.18] | 0.003 | 0.951 |
| MMP2 | -0.161 | 198.441 | 0.872 | [-0.13, 0.11] | -0.001 | 0.972 |
| MMP3 | 1.141 | 205.684 | 0.255 | [-0.05, 0.19] | 0.010 | 0.885 |
| GSTA1 | 0.428 | 203.289 | 0.669 | [-0.18, 0.27] | 0.006 | 0.941 |
| SOD3 | 0.732 | 170.478 | 0.465 | [-0.14, 0.31] | 0.008 | 0.918 |
| CTSG | 0.383 | 191.718 | 0.702 | [-0.17, 0.25] | 0.005 | 0.946 |
| ADH4 | -0.518 | 193.447 | 0.605 | [-0.41, 0.24] | -0.009 | 0.934 |
| HMBS | -0.478 | 166.287 | 0.633 | [-0.2, 0.12] | -0.005 | 0.935 |
| MGP | 1.086 | 179.379 | 0.279 | [-0.08, 0.28] | 0.009 | 0.888 |
| ITGA2B | 0.475 | 174.883 | 0.636 | [-0.22, 0.36] | 0.009 | 0.935 |
| LPA | -1.460 | 202.246 | 0.146 | [-0.66, 0.1] | -0.022 | 0.833 |
| PLEK | -0.008 | 204.495 | 0.993 | [-0.68, 0.67] | 0.000 | 0.999 |
| CD14 | -1.832 | 199.950 | 0.068 | [-0.17, 0.01] | -0.006 | 0.818 |
| COL4A2 | 0.237 | 200.638 | 0.813 | [-0.18, 0.22] | 0.003 | 0.964 |
| MET | -0.455 | 195.459 | 0.650 | [-0.12, 0.08] | -0.002 | 0.935 |
| CFH | 0.595 | 196.465 | 0.552 | [-0.05, 0.09] | 0.001 | 0.925 |
| FCGR3A | -1.427 | 200.285 | 0.155 | [-0.28, 0.04] | -0.010 | 0.839 |
| ITGA5 | 0.093 | 190.781 | 0.926 | [-0.14, 0.15] | 0.001 | 0.979 |
| VIM | -0.328 | 205.998 | 0.743 | [-0.24, 0.17] | -0.004 | 0.946 |
| SERPINF2 | 1.503 | 194.865 | 0.134 | [-0.01, 0.09] | 0.002 | 0.833 |
| F7 | -1.247 | 205.940 | 0.214 | [-0.15, 0.03] | -0.005 | 0.885 |
| ANXA5 | -2.250 | 195.866 | 0.026 | [-0.55, -0.04] | -0.034 | 0.740 |
| KRT16 | 0.237 | 203.277 | 0.813 | [-0.38, 0.48] | 0.006 | 0.964 |
| IGFBP1 | 2.570 | 191.781 | 0.011 | [0.12, 0.91] | 0.057 | 0.740 |
| CELA3B | 0.222 | 196.882 | 0.824 | [-0.14, 0.18] | 0.002 | 0.970 |
| IL6R | 1.968 | 183.958 | 0.051 | [-0., 0.35] | 0.020 | 0.808 |
| ENO2 | -0.932 | 205.786 | 0.352 | [-0.54, 0.19] | -0.019 | 0.900 |
| DBH | 0.648 | 194.271 | 0.518 | [-0.23, 0.45] | 0.010 | 0.924 |
| GSTP1 | 0.048 | 205.659 | 0.962 | [-0.44, 0.46] | 0.001 | 0.997 |
| CD48 | -1.239 | 203.070 | 0.217 | [-0.28, 0.06] | -0.014 | 0.885 |
| LGALS1 | -0.983 | 205.652 | 0.327 | [-0.17, 0.06] | -0.006 | 0.892 |
| FBP1 | 0.133 | 200.667 | 0.894 | [-0.27, 0.31] | 0.002 | 0.976 |
| SPARC | 1.450 | 197.580 | 0.149 | [-0.08, 0.53] | 0.023 | 0.833 |
| TPM1 | -0.944 | 170.116 | 0.346 | [-0.3, 0.1] | -0.011 | 0.897 |
| ANXA4 | 0.724 | 205.964 | 0.470 | [-0.14, 0.3] | 0.010 | 0.918 |
| CSF1 | -2.508 | 204.336 | 0.013 | [-0.33, -0.04] | -0.024 | 0.740 |
| PDGFRB | 1.032 | 197.368 | 0.303 | [-0.11, 0.34] | 0.013 | 0.892 |
| DLD | -1.537 | 188.959 | 0.126 | [-0.3, 0.04] | -0.017 | 0.830 |
| CTSH | -0.732 | 204.910 | 0.465 | [-0.22, 0.1] | -0.006 | 0.918 |
| C1S | 0.538 | 205.270 | 0.591 | [-0.03, 0.06] | 0.001 | 0.930 |
| LTA4H | -0.840 | 192.291 | 0.402 | [-0.22, 0.09] | -0.007 | 0.917 |
| ALDOC | -0.965 | 200.757 | 0.336 | [-0.34, 0.12] | -0.010 | 0.892 |
| C4A | -0.981 | 194.683 | 0.328 | [-0.17, 0.06] | -0.003 | 0.892 |
| C4B | -0.665 | 191.148 | 0.507 | [-0.21, 0.1] | -0.003 | 0.924 |
| H2AZ1 | 0.536 | 189.455 | 0.593 | [-0.36, 0.62] | 0.012 | 0.930 |
| POTEI | -0.209 | 204.003 | 0.835 | [-0.29, 0.24] | -0.003 | 0.971 |
| UBB | 0.202 | 196.280 | 0.840 | [-0.2, 0.24] | 0.002 | 0.971 |
| PGA4 | -1.778 | 194.458 | 0.077 | [-0.42, 0.02] | -0.022 | 0.818 |
| SAA1 | -1.067 | 187.592 | 0.287 | [-0.6, 0.18] | -0.021 | 0.888 |
| SULT1A3 | -0.387 | 204.931 | 0.699 | [-0.14, 0.1] | -0.003 | 0.946 |
| HSPA1A | -0.508 | 203.100 | 0.612 | [-0.38, 0.22] | -0.009 | 0.935 |
| IGLC2 | 1.889 | 205.338 | 0.060 | [-0.01, 0.67] | 0.023 | 0.813 |
| IGHV1 | -0.038 | 205.821 | 0.970 | [-0.36, 0.35] | -0.001 | 0.997 |
| IGHV3 | -0.006 | 205.450 | 0.995 | [-0.27, 0.27] | 0.000 | 0.999 |
| IGHV4 | 0.720 | 199.700 | 0.472 | [-0.14, 0.29] | 0.009 | 0.918 |
| CALM1 | -0.808 | 199.118 | 0.420 | [-0.55, 0.23] | -0.016 | 0.918 |
| SRGN | 1.237 | 146.965 | 0.218 | [-0.1, 0.43] | 0.023 | 0.885 |
| RNASE2 | -1.183 | 193.635 | 0.238 | [-0.3, 0.08] | -0.012 | 0.885 |
| GAA | -1.124 | 200.944 | 0.262 | [-0.21, 0.06] | -0.011 | 0.885 |
| SPP1 | 0.110 | 205.139 | 0.913 | [-0.17, 0.19] | 0.001 | 0.979 |
| PTPRF | 0.123 | 205.474 | 0.902 | [-0.09, 0.1] | 0.001 | 0.976 |
| TXN | 0.601 | 197.273 | 0.549 | [-0.19, 0.37] | 0.009 | 0.925 |
| CTSA | 0.077 | 200.143 | 0.939 | [-0.22, 0.24] | 0.001 | 0.984 |
| C7 | 1.671 | 205.756 | 0.096 | [-0.02, 0.22] | 0.007 | 0.830 |
| PRKAR1A | -2.988 | 205.689 | 0.003 | [-0.68, -0.14] | -0.049 | 0.578 |
| CHGA | -0.460 | 203.367 | 0.646 | [-0.26, 0.16] | -0.006 | 0.935 |
| TFPI | 0.990 | 201.870 | 0.323 | [-0.06, 0.17] | 0.006 | 0.892 |
| PF4V1 | 1.746 | 201.165 | 0.082 | [-0.05, 0.8] | 0.033 | 0.818 |
| KIT | 0.358 | 187.178 | 0.721 | [-0.1, 0.15] | 0.002 | 0.946 |
| ESD | -0.661 | 204.974 | 0.509 | [-0.32, 0.16] | -0.009 | 0.924 |
| CLU | -0.442 | 184.361 | 0.659 | [-0.11, 0.07] | -0.001 | 0.937 |
| GHR | 0.553 | 199.722 | 0.581 | [-0.19, 0.34] | 0.008 | 0.930 |
| HSPA5 | -0.551 | 190.927 | 0.582 | [-0.11, 0.06] | -0.002 | 0.930 |
| LAMC1 | -1.518 | 204.600 | 0.131 | [-0.19, 0.03] | -0.010 | 0.833 |
| HSPA8 | -0.608 | 200.637 | 0.544 | [-0.45, 0.24] | -0.011 | 0.924 |
| LIPC | -1.176 | 179.519 | 0.241 | [-0.34, 0.09] | -0.014 | 0.885 |
| PYGB | -0.874 | 170.250 | 0.383 | [-0.34, 0.13] | -0.013 | 0.917 |
| PYGM | -1.512 | 166.163 | 0.132 | [-0.58, 0.08] | -0.029 | 0.833 |
| MBL2 | -1.047 | 199.123 | 0.296 | [-0.52, 0.16] | -0.016 | 0.888 |
| LAMP1 | -2.248 | 185.184 | 0.026 | [-0.33, -0.02] | -0.019 | 0.740 |
| FGFR1 | -0.925 | 196.669 | 0.356 | [-0.2, 0.07] | -0.007 | 0.907 |
| G6PD | -0.370 | 196.237 | 0.712 | [-0.34, 0.23] | -0.007 | 0.946 |
| CETP | 0.339 | 204.776 | 0.735 | [-0.19, 0.27] | 0.004 | 0.946 |
| SCGB1A1 | 1.275 | 201.376 | 0.204 | [-0.06, 0.29] | 0.011 | 0.885 |
| IGF2R | -0.230 | 195.604 | 0.818 | [-0.09, 0.07] | -0.001 | 0.966 |
| ADH5 | -0.967 | 188.999 | 0.335 | [-0.34, 0.12] | -0.013 | 0.892 |
| COL6A1 | -2.423 | 188.584 | 0.016 | [-0.22, -0.02] | -0.012 | 0.740 |
| COL6A2 | -1.030 | 197.073 | 0.304 | [-0.19, 0.06] | -0.007 | 0.892 |
| COL6A3 | -1.807 | 201.396 | 0.072 | [-0.18, 0.01] | -0.008 | 0.818 |
| F5 | 0.291 | 204.269 | 0.772 | [-0.09, 0.12] | 0.001 | 0.954 |
| PIP | -1.804 | 149.850 | 0.073 | [-0.4, 0.02] | -0.023 | 0.818 |
| FCGR2A | -2.068 | 200.797 | 0.040 | [-0.28, -0.01] | -0.013 | 0.808 |
| ANXA3 | 0.571 | 190.611 | 0.569 | [-0.2, 0.35] | 0.010 | 0.926 |
| ACTN1 | -0.307 | 203.013 | 0.759 | [-0.66, 0.48] | -0.009 | 0.952 |
| ACE | -0.023 | 192.525 | 0.982 | [-0.17, 0.17] | 0.000 | 0.998 |
| CDH1 | -0.559 | 205.527 | 0.577 | [-0.15, 0.08] | -0.003 | 0.928 |
| SRC | 0.116 | 205.305 | 0.908 | [-0.18, 0.2] | 0.001 | 0.976 |
| PEPD | 0.192 | 201.411 | 0.848 | [-0.1, 0.12] | 0.001 | 0.971 |
| LAMP2 | 0.592 | 199.417 | 0.554 | [-0.09, 0.17] | 0.003 | 0.925 |
| RNH1 | -1.543 | 181.342 | 0.125 | [-0.38, 0.05] | -0.019 | 0.830 |
| BMP1 | 0.188 | 196.147 | 0.851 | [-0.14, 0.17] | 0.002 | 0.971 |
| NCAM1 | -0.208 | 195.628 | 0.836 | [-0.11, 0.09] | -0.001 | 0.971 |
| ICAM2 | -1.961 | 179.614 | 0.051 | [-0.33, 0] | -0.015 | 0.808 |
| VCAN | 0.835 | 193.594 | 0.405 | [-0.09, 0.21] | 0.007 | 0.918 |
| EEF2 | 1.098 | 205.033 | 0.273 | [-0.1, 0.35] | 0.015 | 0.888 |
| KRT10 | 1.432 | 205.757 | 0.154 | [-0.11, 0.72] | 0.026 | 0.834 |
| KRT5 | 1.519 | 205.026 | 0.130 | [-0.09, 0.73] | 0.030 | 0.833 |
| PDIA4 | -0.830 | 204.388 | 0.407 | [-0.36, 0.15] | -0.010 | 0.918 |
| C6 | 1.631 | 204.279 | 0.104 | [-0.01, 0.11] | 0.003 | 0.830 |
| ACP5 | -0.342 | 195.921 | 0.733 | [-0.23, 0.16] | -0.004 | 0.946 |
| CEACAM1 | -0.624 | 205.804 | 0.533 | [-0.33, 0.17] | -0.009 | 0.924 |
| TPT1 | -0.755 | 201.508 | 0.451 | [-0.33, 0.15] | -0.009 | 0.918 |
| ALAD | 0.266 | 191.237 | 0.790 | [-0.28, 0.37] | 0.005 | 0.954 |
| PRG2 | -0.823 | 187.445 | 0.412 | [-0.34, 0.14] | -0.009 | 0.918 |
| LCP1 | 0.073 | 202.950 | 0.942 | [-0.09, 0.09] | 0.000 | 0.987 |
| PLS3 | -1.478 | 142.834 | 0.142 | [-0.43, 0.06] | -0.024 | 0.833 |
| APEH | -0.717 | 189.586 | 0.474 | [-0.33, 0.16] | -0.011 | 0.918 |
| ENO3 | 0.847 | 160.326 | 0.398 | [-0.11, 0.28] | 0.011 | 0.917 |
| COL11A2 | -0.142 | 191.676 | 0.887 | [-0.22, 0.19] | -0.002 | 0.976 |
| CD59 | -0.729 | 190.620 | 0.467 | [-0.26, 0.12] | -0.008 | 0.918 |
| SELL | -0.391 | 184.982 | 0.697 | [-0.16, 0.11] | -0.002 | 0.946 |
| MIF | 1.064 | 200.755 | 0.289 | [-0.18, 0.61] | 0.020 | 0.888 |
| FOLR2 | 1.379 | 202.665 | 0.169 | [-0.05, 0.29] | 0.013 | 0.857 |
| PRF1 | 0.163 | 197.106 | 0.870 | [-0.11, 0.13] | 0.001 | 0.972 |
| PRKCSH | -1.154 | 204.148 | 0.250 | [-0.24, 0.06] | -0.010 | 0.885 |
| FDPS | -0.682 | 204.917 | 0.496 | [-0.32, 0.16] | -0.010 | 0.918 |
| CPM | -1.335 | 197.850 | 0.183 | [-0.3, 0.06] | -0.016 | 0.863 |
| NID1 | -0.148 | 205.995 | 0.882 | [-0.11, 0.1] | -0.001 | 0.976 |
| PKM | -1.003 | 195.781 | 0.317 | [-0.48, 0.16] | -0.015 | 0.892 |
| HSP90B1 | -0.733 | 194.736 | 0.464 | [-0.19, 0.09] | -0.004 | 0.918 |
| IDE | 0.133 | 185.903 | 0.894 | [-0.22, 0.25] | 0.002 | 0.976 |
| MMP9 | 1.044 | 190.940 | 0.298 | [-0.14, 0.45] | 0.016 | 0.888 |
| JUP | 0.635 | 200.800 | 0.526 | [-0.2, 0.38] | 0.011 | 0.924 |
| CPA1 | 0.661 | 200.366 | 0.510 | [-0.14, 0.28] | 0.008 | 0.924 |
| CPB1 | -0.335 | 201.112 | 0.738 | [-0.26, 0.19] | -0.005 | 0.946 |
| FABP4 | -2.848 | 194.489 | 0.005 | [-0.68, -0.12] | -0.042 | 0.705 |
| ANPEP | 0.004 | 204.186 | 0.997 | [-0.08, 0.08] | 0.000 | 0.999 |
| PVR | 2.146 | 205.552 | 0.033 | [0.01, 0.2] | 0.009 | 0.808 |
| RAC2 | -1.061 | 183.449 | 0.290 | [-0.42, 0.13] | -0.018 | 0.888 |
| CPN1 | 0.990 | 202.029 | 0.323 | [-0.04, 0.11] | 0.003 | 0.892 |
| ARSA | 1.571 | 205.287 | 0.118 | [-0.04, 0.32] | 0.017 | 0.830 |
| B4GALT1 | -1.372 | 198.341 | 0.171 | [-0.23, 0.04] | -0.010 | 0.857 |
| EZR | 0.193 | 205.253 | 0.847 | [-0.36, 0.44] | 0.005 | 0.971 |
| NME1 | 0.693 | 205.293 | 0.489 | [-0.17, 0.36] | 0.011 | 0.918 |
| IGLL1 | -0.529 | 205.164 | 0.597 | [-0.38, 0.22] | -0.005 | 0.930 |
| ST6GAL1 | 0.805 | 198.270 | 0.422 | [-0.09, 0.22] | 0.008 | 0.918 |
| DSP | 0.040 | 203.194 | 0.968 | [-0.26, 0.27] | 0.001 | 0.997 |
| TIMP2 | -0.443 | 204.790 | 0.658 | [-0.15, 0.09] | -0.003 | 0.937 |
| CD44 | -2.256 | 191.072 | 0.025 | [-0.33, -0.02] | -0.016 | 0.740 |
| SELP | 1.350 | 196.882 | 0.178 | [-0.08, 0.4] | 0.019 | 0.860 |
| ACAN | -0.123 | 191.932 | 0.903 | [-0.17, 0.15] | -0.001 | 0.976 |
| CBR1 | -1.294 | 190.066 | 0.197 | [-0.45, 0.09] | -0.022 | 0.885 |
| HLA | -1.609 | 205.673 | 0.109 | [-0.45, 0.05] | -0.021 | 0.830 |
| PNLIP | 1.373 | 189.094 | 0.172 | [-0.08, 0.47] | 0.025 | 0.857 |
| PECAM1 | 0.465 | 180.237 | 0.643 | [-0.13, 0.21] | 0.005 | 0.935 |
| SELE | -0.899 | 197.268 | 0.370 | [-0.41, 0.15] | -0.015 | 0.913 |
| FAH | -1.537 | 205.181 | 0.126 | [-0.34, 0.04] | -0.013 | 0.830 |
| NAGA | -0.629 | 199.003 | 0.530 | [-0.27, 0.14] | -0.008 | 0.924 |
| GOT1 | -1.369 | 205.105 | 0.172 | [-0.32, 0.06] | -0.014 | 0.857 |
| ITGA2 | -1.023 | 179.032 | 0.308 | [-0.26, 0.08] | -0.011 | 0.892 |
| ENG | 0.281 | 192.549 | 0.779 | [-0.1, 0.14] | 0.002 | 0.954 |
| GM2A | 0.056 | 205.516 | 0.956 | [-0.15, 0.16] | 0.001 | 0.994 |
| CR1 | -0.255 | 191.662 | 0.799 | [-0.18, 0.14] | -0.003 | 0.960 |
| IGFBP3 | 0.768 | 199.766 | 0.443 | [-0.05, 0.11] | 0.002 | 0.918 |
| TCP1 | -0.767 | 195.886 | 0.444 | [-0.28, 0.12] | -0.010 | 0.918 |
| IGFBP2 | 0.057 | 201.412 | 0.955 | [-0.26, 0.28] | 0.001 | 0.994 |
| ARF4 | -1.230 | 198.611 | 0.220 | [-0.4, 0.09] | -0.019 | 0.885 |
| VCL | -0.460 | 203.976 | 0.646 | [-0.59, 0.37] | -0.010 | 0.935 |
| LBP | -2.717 | 189.159 | 0.007 | [-0.37, -0.06] | -0.017 | 0.705 |
| PGAM1 | -0.676 | 199.143 | 0.500 | [-0.39, 0.19] | -0.011 | 0.918 |
| ATF6 | 0.984 | 202.859 | 0.326 | [-0.11, 0.31] | 0.012 | 0.892 |
| PAM | -1.261 | 205.019 | 0.209 | [-0.14, 0.03] | -0.006 | 0.885 |
| CDH2 | -1.556 | 198.612 | 0.121 | [-0.25, 0.03] | -0.012 | 0.830 |
| VCAM1 | -0.674 | 204.482 | 0.501 | [-0.14, 0.07] | -0.003 | 0.919 |
| HK1 | -1.358 | 203.895 | 0.176 | [-0.44, 0.08] | -0.021 | 0.857 |
| ORM2 | 1.463 | 198.468 | 0.145 | [-0.06, 0.41] | 0.012 | 0.833 |
| ITIH2 | 2.132 | 205.055 | 0.034 | [0.01, 0.13] | 0.004 | 0.808 |
| ITIH1 | 0.265 | 184.122 | 0.791 | [-0.06, 0.08] | 0.001 | 0.955 |
| PI3 | -1.048 | 188.532 | 0.296 | [-0.39, 0.12] | -0.015 | 0.888 |
| AMY2B | -0.364 | 202.643 | 0.716 | [-0.2, 0.13] | -0.003 | 0.946 |
| TYMP | -1.153 | 200.860 | 0.250 | [-0.43, 0.11] | -0.019 | 0.885 |
| CR2 | -0.480 | 200.617 | 0.632 | [-0.23, 0.14] | -0.005 | 0.935 |
| TCN1 | 0.472 | 202.658 | 0.637 | [-0.17, 0.28] | 0.007 | 0.935 |
| TCN2 | -0.991 | 197.723 | 0.323 | [-0.19, 0.06] | -0.007 | 0.892 |
| ANXA7 | -1.597 | 205.610 | 0.112 | [-0.31, 0.03] | -0.017 | 0.830 |
| AZU1 | 1.250 | 129.400 | 0.214 | [-0.09, 0.38] | 0.020 | 0.885 |
| PSMB1 | -1.299 | 198.940 | 0.195 | [-0.39, 0.08] | -0.019 | 0.885 |
| PZP | 1.217 | 205.000 | 0.225 | [-0.25, 1.05] | 0.036 | 0.885 |
| OGN | -1.882 | 205.821 | 0.061 | [-0.25, 0.01] | -0.012 | 0.815 |
| CAST | 0.532 | 189.576 | 0.595 | [-0.19, 0.32] | 0.008 | 0.930 |
| C4BPB | -1.577 | 198.605 | 0.116 | [-0.34, 0.04] | -0.011 | 0.830 |
| COL5A1 | -0.475 | 205.713 | 0.635 | [-0.29, 0.18] | -0.007 | 0.935 |
| FLG | 0.068 | 201.425 | 0.946 | [-0.27, 0.29] | 0.001 | 0.989 |
| AGA | 0.990 | 192.687 | 0.323 | [-0.07, 0.21] | 0.008 | 0.892 |
| GSTM3 | -0.632 | 188.851 | 0.528 | [-0.21, 0.11] | -0.007 | 0.924 |
| CSRP1 | 0.689 | 203.452 | 0.492 | [-0.15, 0.31] | 0.009 | 0.918 |
| FLNA | 0.900 | 194.705 | 0.369 | [-0.29, 0.77] | 0.025 | 0.913 |
| ACO1 | 0.350 | 200.448 | 0.727 | [-0.18, 0.26] | 0.005 | 0.946 |
| NT5E | 0.865 | 191.342 | 0.388 | [-0.09, 0.24] | 0.010 | 0.917 |
| GPD1 | -2.290 | 200.799 | 0.023 | [-0.52, -0.04] | -0.034 | 0.740 |
| EPHA1 | -2.043 | 205.288 | 0.042 | [-0.34, -0.01] | -0.019 | 0.808 |
| BGN | -1.415 | 202.181 | 0.159 | [-0.2, 0.03] | -0.011 | 0.841 |
| CD9 | 1.590 | 195.104 | 0.113 | [-0.08, 0.73] | 0.038 | 0.830 |
| PCMT1 | -0.057 | 203.962 | 0.955 | [-0.26, 0.25] | -0.001 | 0.994 |
| TNXB | 1.209 | 188.184 | 0.228 | [-0.04, 0.17] | 0.005 | 0.885 |
| IDS | 2.752 | 202.559 | 0.006 | [0.06, 0.35] | 0.024 | 0.705 |
| UBA1 | -0.469 | 186.490 | 0.640 | [-0.29, 0.18] | -0.007 | 0.935 |
| GPX3 | 0.954 | 204.553 | 0.341 | [-0.05, 0.15] | 0.003 | 0.897 |
| NME2 | -0.467 | 196.757 | 0.641 | [-0.48, 0.3] | -0.010 | 0.935 |
| ENPP1 | -1.500 | 184.273 | 0.135 | [-0.25, 0.03] | -0.015 | 0.833 |
| FGFR4 | -2.784 | 198.811 | 0.006 | [-0.45, -0.08] | -0.034 | 0.705 |
| SPRR2D | -1.770 | 190.882 | 0.078 | [-0.49, 0.03] | -0.028 | 0.818 |
| IGFBP4 | -1.637 | 204.910 | 0.103 | [-0.13, 0.01] | -0.005 | 0.830 |
| CPN2 | 0.863 | 205.998 | 0.389 | [-0.04, 0.1] | 0.002 | 0.917 |
| PROZ | 0.332 | 190.113 | 0.740 | [-0.14, 0.2] | 0.002 | 0.946 |
| MRC1 | -0.897 | 205.743 | 0.371 | [-0.13, 0.05] | -0.004 | 0.914 |
| IGHV1 | -0.962 | 205.940 | 0.337 | [-0.45, 0.15] | -0.012 | 0.893 |
| CES1 | -0.778 | 201.743 | 0.438 | [-0.35, 0.15] | -0.011 | 0.918 |
| FBLN1 | -0.841 | 191.084 | 0.401 | [-0.16, 0.06] | -0.004 | 0.917 |
| ITGA6 | -0.491 | 205.863 | 0.624 | [-0.36, 0.22] | -0.009 | 0.935 |
| PPIB | -0.024 | 203.970 | 0.981 | [-0.33, 0.32] | 0.000 | 0.998 |
| WARS1 | -2.670 | 199.863 | 0.008 | [-0.4, -0.06] | -0.027 | 0.705 |
| CBLN1 | 1.493 | 189.684 | 0.137 | [-0.06, 0.46] | 0.021 | 0.833 |
| PTPRB | 2.079 | 205.424 | 0.039 | [0.01, 0.3] | 0.019 | 0.808 |
| PTPRD | -0.800 | 203.259 | 0.424 | [-0.2, 0.09] | -0.008 | 0.918 |
| PTPRG | -0.501 | 195.070 | 0.617 | [-0.12, 0.07] | -0.002 | 0.935 |
| PTPRZ1 | -0.171 | 192.484 | 0.864 | [-0.17, 0.14] | -0.002 | 0.971 |
| LORICRIN | 0.566 | 174.325 | 0.572 | [-0.18, 0.32] | 0.009 | 0.926 |
| AHCY | -1.329 | 195.970 | 0.185 | [-0.47, 0.09] | -0.022 | 0.863 |
| CFL1 | -0.445 | 201.007 | 0.657 | [-0.66, 0.42] | -0.011 | 0.937 |
| LAMA2 | 0.477 | 203.645 | 0.634 | [-0.08, 0.13] | 0.003 | 0.935 |
| GPT | -1.348 | 203.840 | 0.179 | [-0.46, 0.09] | -0.022 | 0.860 |
| CRHBP | -0.287 | 205.491 | 0.774 | [-0.12, 0.09] | -0.002 | 0.954 |
| IGFBP6 | -1.057 | 204.925 | 0.292 | [-0.16, 0.05] | -0.005 | 0.888 |
| IGFBP5 | 1.200 | 191.418 | 0.232 | [-0.04, 0.17] | 0.005 | 0.885 |
| ACP1 | -1.102 | 192.078 | 0.272 | [-0.39, 0.11] | -0.015 | 0.888 |
| TNC | -1.170 | 202.021 | 0.244 | [-0.19, 0.05] | -0.007 | 0.885 |
| MYL9 | -0.744 | 193.819 | 0.458 | [-0.25, 0.11] | -0.008 | 0.918 |
| AZGP1 | 0.448 | 205.249 | 0.655 | [-0.07, 0.11] | 0.001 | 0.936 |
| CTSS | -0.837 | 193.340 | 0.403 | [-0.18, 0.07] | -0.005 | 0.917 |
| PSMA1 | -1.554 | 180.286 | 0.122 | [-0.43, 0.05] | -0.020 | 0.830 |
| PSMA2 | -1.287 | 198.754 | 0.200 | [-0.49, 0.1] | -0.023 | 0.885 |
| PSMA3 | -1.907 | 191.720 | 0.058 | [-0.5, 0.01] | -0.028 | 0.811 |
| PSMA4 | -0.845 | 205.024 | 0.399 | [-0.3, 0.12] | -0.011 | 0.917 |
| S100P | 1.300 | 195.084 | 0.195 | [-0.11, 0.53] | 0.028 | 0.885 |
| PTX3 | -0.314 | 194.217 | 0.754 | [-0.15, 0.11] | -0.003 | 0.951 |
| MSN | -0.006 | 203.508 | 0.995 | [-0.28, 0.28] | 0.000 | 0.999 |
| S100A4 | 0.394 | 193.651 | 0.694 | [-0.25, 0.37] | 0.006 | 0.946 |
| MGAT1 | -2.005 | 205.765 | 0.046 | [-0.27, 0] | -0.014 | 0.808 |
| PTBP1 | -0.185 | 201.636 | 0.854 | [-0.19, 0.16] | -0.002 | 0.971 |
| TARS1 | -2.479 | 159.864 | 0.014 | [-0.29, -0.03] | -0.021 | 0.740 |
| EEF1G | -0.574 | 205.840 | 0.567 | [-0.4, 0.22] | -0.010 | 0.926 |
| MST1 | -0.638 | 198.702 | 0.524 | [-0.1, 0.05] | -0.002 | 0.924 |
| CNTFR | 1.104 | 203.758 | 0.271 | [-0.08, 0.27] | 0.011 | 0.888 |
| PON1 | 1.004 | 175.498 | 0.317 | [-0.11, 0.33] | 0.008 | 0.892 |
| YWHAQ | -1.666 | 185.085 | 0.097 | [-0.68, 0.06] | -0.038 | 0.830 |
| DPP4 | 0.858 | 201.476 | 0.392 | [-0.06, 0.16] | 0.004 | 0.917 |
| CALR | -0.711 | 200.738 | 0.478 | [-0.28, 0.13] | -0.007 | 0.918 |
| CANX | 0.353 | 176.360 | 0.725 | [-0.09, 0.13] | 0.003 | 0.946 |
| CFP | 0.209 | 203.514 | 0.835 | [-0.06, 0.07] | 0.000 | 0.971 |
| IL1R2 | -1.509 | 202.849 | 0.133 | [-0.45, 0.06] | -0.021 | 0.833 |
| PSMB8 | -1.578 | 205.559 | 0.116 | [-0.43, 0.05] | -0.022 | 0.830 |
| PSMA5 | -1.588 | 190.163 | 0.114 | [-0.48, 0.05] | -0.025 | 0.830 |
| PSMB4 | -0.979 | 205.585 | 0.329 | [-0.43, 0.14] | -0.018 | 0.892 |
| PSMB6 | -1.631 | 191.460 | 0.105 | [-0.45, 0.04] | -0.024 | 0.830 |
| PSMB5 | -1.343 | 191.187 | 0.181 | [-0.39, 0.07] | -0.019 | 0.861 |
| GRN | -0.096 | 174.685 | 0.924 | [-0.15, 0.14] | -0.001 | 0.979 |
| PTPRM | 0.773 | 200.773 | 0.440 | [-0.08, 0.18] | 0.005 | 0.918 |
| LAP3 | -1.153 | 200.791 | 0.250 | [-0.31, 0.08] | -0.015 | 0.885 |
| CD1C | -0.987 | 192.243 | 0.325 | [-0.18, 0.06] | -0.008 | 0.892 |
| IMPA1 | -0.093 | 204.482 | 0.926 | [-0.25, 0.22] | -0.001 | 0.979 |
| CCN2 | -0.336 | 199.773 | 0.737 | [-0.26, 0.18] | -0.004 | 0.946 |
| PTPN6 | -0.134 | 205.035 | 0.894 | [-0.24, 0.21] | -0.002 | 0.976 |
| TKT | -1.535 | 197.304 | 0.126 | [-0.35, 0.04] | -0.017 | 0.830 |
| SERPINA4 | -1.192 | 185.621 | 0.235 | [-0.13, 0.03] | -0.003 | 0.885 |
| MARCKS | 0.676 | 204.167 | 0.500 | [-0.07, 0.13] | 0.005 | 0.918 |
| ERP29 | -1.458 | 180.798 | 0.147 | [-0.54, 0.08] | -0.028 | 0.833 |
| PRDX6 | -0.630 | 191.728 | 0.529 | [-0.43, 0.22] | -0.010 | 0.924 |
| GATD3 | -0.707 | 191.106 | 0.480 | [-0.46, 0.21] | -0.011 | 0.918 |
| BLVRB | 0.176 | 180.511 | 0.861 | [-0.26, 0.31] | 0.002 | 0.971 |
| PRDX5 | 0.389 | 203.589 | 0.698 | [-0.25, 0.37] | 0.007 | 0.946 |
| DDT | -1.289 | 183.942 | 0.199 | [-0.42, 0.09] | -0.017 | 0.885 |
| CMPK1 | 0.364 | 177.524 | 0.716 | [-0.21, 0.31] | 0.006 | 0.946 |
| PEBP1 | 0.115 | 201.765 | 0.909 | [-0.24, 0.27] | 0.002 | 0.976 |
| PDIA3 | -0.885 | 203.416 | 0.377 | [-0.41, 0.16] | -0.013 | 0.917 |
| AXL | -0.241 | 197.779 | 0.810 | [-0.15, 0.12] | -0.002 | 0.964 |
| PKLR | 0.529 | 190.595 | 0.597 | [-0.1, 0.17] | 0.005 | 0.930 |
| CLIP1 | 1.519 | 183.892 | 0.130 | [-0.07, 0.52] | 0.028 | 0.833 |
| SRI | -1.042 | 200.241 | 0.298 | [-0.32, 0.1] | -0.013 | 0.888 |
| SERPINB1 | -0.424 | 201.078 | 0.672 | [-0.49, 0.32] | -0.010 | 0.943 |
| LCN1 | -1.972 | 205.991 | 0.050 | [-0.42, 0] | -0.028 | 0.808 |
| CORO1A | -0.644 | 204.736 | 0.521 | [-0.58, 0.29] | -0.015 | 0.924 |
| GDI1 | -0.942 | 201.298 | 0.347 | [-0.53, 0.19] | -0.019 | 0.897 |
| S100A7 | -0.329 | 203.338 | 0.743 | [-0.4, 0.29] | -0.008 | 0.946 |
| ATIC | -0.268 | 202.135 | 0.789 | [-0.3, 0.23] | -0.005 | 0.954 |
| CASP14 | -1.463 | 203.325 | 0.145 | [-0.48, 0.07] | -0.026 | 0.833 |
| YWHAB | -0.351 | 204.082 | 0.726 | [-0.52, 0.36] | -0.008 | 0.946 |
| STIP1 | -0.848 | 205.869 | 0.398 | [-0.54, 0.21] | -0.019 | 0.917 |
| S100A11 | -0.972 | 176.003 | 0.332 | [-0.41, 0.14] | -0.016 | 0.892 |
| L1CAM | 0.283 | 196.798 | 0.778 | [-0.13, 0.17] | 0.002 | 0.954 |
| PRDX2 | -0.219 | 180.878 | 0.827 | [-0.31, 0.25] | -0.003 | 0.970 |
| HPD | -1.541 | 204.730 | 0.125 | [-0.56, 0.07] | -0.030 | 0.830 |
| ICAM3 | 0.245 | 193.528 | 0.807 | [-0.16, 0.2] | 0.002 | 0.962 |
| CDH5 | 0.269 | 205.684 | 0.788 | [-0.09, 0.12] | 0.001 | 0.954 |
| MAN1A1 | -2.355 | 200.828 | 0.019 | [-0.16, -0.01] | -0.008 | 0.740 |
| MCM4 | -1.168 | 164.163 | 0.244 | [-0.31, 0.08] | -0.012 | 0.885 |
| GALNS | 0.872 | 196.402 | 0.384 | [-0.11, 0.29] | 0.011 | 0.917 |
| RNASE4 | -0.561 | 197.793 | 0.575 | [-0.1, 0.06] | -0.002 | 0.928 |
| HSPA4 | -2.053 | 200.866 | 0.041 | [-0.38, -0.01] | -0.026 | 0.808 |
| PRSS3 | -2.546 | 204.267 | 0.012 | [-0.52, -0.07] | -0.033 | 0.740 |
| GPC1 | 1.434 | 166.928 | 0.153 | [-0.05, 0.29] | 0.017 | 0.834 |
| SERPINB6 | -0.279 | 201.329 | 0.781 | [-0.37, 0.28] | -0.005 | 0.954 |
| SFTPD | -1.145 | 172.821 | 0.254 | [-0.38, 0.1] | -0.017 | 0.885 |
| THBS2 | 1.569 | 197.892 | 0.118 | [-0.07, 0.62] | 0.026 | 0.830 |
| THBS4 | -0.507 | 194.591 | 0.613 | [-0.2, 0.12] | -0.004 | 0.935 |
| KRT9 | 1.925 | 205.048 | 0.056 | [-0.01, 0.88] | 0.037 | 0.810 |
| SAA4 | 0.036 | 205.487 | 0.972 | [-0.16, 0.16] | 0.000 | 0.997 |
| FBN1 | -0.531 | 199.637 | 0.596 | [-0.14, 0.08] | -0.003 | 0.930 |
| FBN2 | -1.664 | 201.984 | 0.098 | [-0.29, 0.02] | -0.015 | 0.830 |
| MYH9 | 0.997 | 197.551 | 0.320 | [-0.13, 0.39] | 0.017 | 0.892 |
| TIE1 | 0.046 | 204.973 | 0.964 | [-0.09, 0.1] | 0.000 | 0.997 |
| GLRX | 0.358 | 199.123 | 0.721 | [-0.21, 0.31] | 0.005 | 0.946 |
| PPM1A | 0.794 | 205.438 | 0.428 | [-0.3, 0.7] | 0.018 | 0.918 |
| IGFALS | 1.134 | 203.176 | 0.258 | [-0.03, 0.13] | 0.003 | 0.885 |
| KRT2 | 1.151 | 205.684 | 0.251 | [-0.16, 0.62] | 0.021 | 0.885 |
| FLT4 | -1.246 | 205.536 | 0.214 | [-0.18, 0.04] | -0.007 | 0.885 |
| KDR | 0.773 | 191.671 | 0.440 | [-0.09, 0.2] | 0.007 | 0.918 |
| CHI3L1 | 0.987 | 197.796 | 0.325 | [-0.14, 0.41] | 0.016 | 0.892 |
| GGT5 | 0.540 | 190.560 | 0.590 | [-0.14, 0.25] | 0.007 | 0.930 |
| PGM1 | -1.467 | 195.001 | 0.144 | [-0.51, 0.08] | -0.026 | 0.833 |
| SERPINF1 | -3.002 | 196.728 | 0.003 | [-0.2, -0.04] | -0.008 | 0.578 |
| GMPR | 0.410 | 205.933 | 0.682 | [-0.2, 0.3] | 0.006 | 0.946 |
| CFHR2 | 1.372 | 186.885 | 0.172 | [-0.07, 0.41] | 0.013 | 0.857 |
| HPCAL1 | -0.765 | 192.722 | 0.445 | [-0.48, 0.21] | -0.016 | 0.918 |
| TAGLN2 | 0.033 | 202.817 | 0.974 | [-0.66, 0.68] | 0.001 | 0.997 |
| TALDO1 | -1.226 | 197.676 | 0.222 | [-0.36, 0.08] | -0.014 | 0.885 |
| SNCA | -0.779 | 200.560 | 0.437 | [-0.64, 0.28] | -0.020 | 0.918 |
| ATP6V1A | -0.577 | 189.829 | 0.564 | [-0.28, 0.15] | -0.008 | 0.926 |
| COL15A1 | -0.694 | 203.882 | 0.489 | [-0.17, 0.08] | -0.005 | 0.918 |
| COL18A1 | -1.321 | 205.891 | 0.188 | [-0.13, 0.03] | -0.005 | 0.867 |
| CAPG | -0.837 | 203.996 | 0.404 | [-0.34, 0.14] | -0.014 | 0.917 |
| IL6ST | 0.309 | 191.610 | 0.758 | [-0.06, 0.08] | 0.001 | 0.952 |
| GP5 | 0.422 | 194.262 | 0.673 | [-0.25, 0.38] | 0.007 | 0.944 |
| MDH1 | -0.872 | 197.892 | 0.385 | [-0.37, 0.14] | -0.012 | 0.917 |
| PMEL | 0.403 | 189.760 | 0.687 | [-0.17, 0.26] | 0.005 | 0.946 |
| LEP | -0.344 | 196.599 | 0.731 | [-0.35, 0.25] | -0.006 | 0.946 |
| PTGDS | -0.792 | 203.543 | 0.430 | [-0.15, 0.07] | -0.004 | 0.918 |
| UBA7 | -0.826 | 201.646 | 0.410 | [-0.39, 0.16] | -0.015 | 0.918 |
| CSK | 0.522 | 205.880 | 0.602 | [-0.18, 0.3] | 0.007 | 0.932 |
| NBL1 | -1.085 | 201.414 | 0.279 | [-0.2, 0.06] | -0.007 | 0.888 |
| FOLR3 | -0.219 | 205.858 | 0.827 | [-0.29, 0.23] | -0.004 | 0.970 |
| ECI1 | 0.103 | 205.996 | 0.918 | [-0.19, 0.21] | 0.001 | 0.979 |
| PRCP | -0.077 | 190.069 | 0.938 | [-0.12, 0.11] | 0.000 | 0.984 |
| PAFAH1B1 | -1.889 | 181.263 | 0.060 | [-0.47, 0.01] | -0.030 | 0.813 |
| MCAM | -0.147 | 202.395 | 0.883 | [-0.1, 0.08] | -0.001 | 0.976 |
| BTD | -0.134 | 205.305 | 0.893 | [-0.07, 0.06] | 0.000 | 0.976 |
| SYK | -0.593 | 181.190 | 0.554 | [-0.22, 0.12] | -0.006 | 0.925 |
| RANBP1 | 0.541 | 192.684 | 0.589 | [-0.14, 0.25] | 0.007 | 0.930 |
| AFM | 0.349 | 198.259 | 0.728 | [-0.06, 0.09] | 0.001 | 0.946 |
| TSFM | -0.577 | 206.000 | 0.565 | [-0.24, 0.13] | -0.006 | 0.926 |
| PPIC | 0.680 | 205.062 | 0.497 | [-0.11, 0.22] | 0.006 | 0.918 |
| CRKL | 0.198 | 186.567 | 0.843 | [-0.18, 0.22] | 0.003 | 0.971 |
| NOTCH1 | 1.200 | 202.786 | 0.232 | [-0.03, 0.14] | 0.006 | 0.885 |
| CAPZA2 | -1.016 | 205.959 | 0.311 | [-0.32, 0.1] | -0.013 | 0.892 |
| CAPZB | -0.036 | 200.699 | 0.971 | [-0.32, 0.31] | -0.001 | 0.997 |
| LGALS7 | 0.286 | 190.664 | 0.775 | [-0.26, 0.35] | 0.006 | 0.954 |
| LIMS1 | -0.472 | 191.733 | 0.638 | [-0.4, 0.25] | -0.009 | 0.935 |
| LEPR | -1.058 | 192.637 | 0.291 | [-0.26, 0.08] | -0.011 | 0.888 |
| PIP4K2A | -0.561 | 199.311 | 0.575 | [-0.29, 0.16] | -0.008 | 0.928 |
| ARCN1 | 0.900 | 205.678 | 0.369 | [-0.31, 0.82] | 0.030 | 0.913 |
| GCLC | -0.585 | 184.770 | 0.560 | [-0.35, 0.19] | -0.010 | 0.925 |
| GCLM | -0.994 | 197.983 | 0.321 | [-0.27, 0.09] | -0.012 | 0.892 |
| SERPINB4 | 0.568 | 168.259 | 0.571 | [-0.58, 1.04] | 0.020 | 0.926 |
| GSS | -2.270 | 201.669 | 0.024 | [-0.27, -0.02] | -0.015 | 0.740 |
| KRT6C | 1.606 | 205.991 | 0.110 | [-0.07, 0.71] | 0.031 | 0.830 |
| HSPA13 | 0.579 | 189.576 | 0.563 | [-0.12, 0.22] | 0.006 | 0.926 |
| MASP1 | -1.503 | 205.971 | 0.134 | [-0.12, 0.02] | -0.004 | 0.833 |
| ADGRE5 | -0.820 | 195.110 | 0.413 | [-0.19, 0.08] | -0.007 | 0.918 |
| ALDH9A1 | -0.747 | 185.674 | 0.456 | [-0.26, 0.12] | -0.008 | 0.918 |
| RPIA | 0.952 | 197.264 | 0.342 | [-0.12, 0.36] | 0.015 | 0.897 |
| LMAN1 | -1.656 | 195.048 | 0.099 | [-0.39, 0.03] | -0.020 | 0.830 |
| CCT3 | 0.571 | 187.707 | 0.569 | [-0.25, 0.46] | 0.014 | 0.926 |
| ARRB1 | -0.996 | 192.011 | 0.321 | [-0.45, 0.15] | -0.018 | 0.892 |
| AARS1 | -0.910 | 199.822 | 0.364 | [-0.22, 0.08] | -0.009 | 0.913 |
| SARS1 | -0.004 | 180.583 | 0.997 | [-0.19, 0.19] | 0.000 | 0.999 |
| MAN2A2 | -3.472 | 205.545 | 0.001 | [-0.23, -0.06] | -0.016 | 0.461 |
| PSMB3 | 0.949 | 205.980 | 0.344 | [-0.1, 0.27] | 0.011 | 0.897 |
| PSMB2 | -0.035 | 205.998 | 0.972 | [-0.29, 0.28] | -0.001 | 0.997 |
| THBS3 | -1.355 | 177.400 | 0.177 | [-0.25, 0.05] | -0.013 | 0.860 |
| COMP | -0.602 | 197.392 | 0.548 | [-0.17, 0.09] | -0.004 | 0.925 |
| HINT1 | -0.192 | 201.829 | 0.848 | [-0.23, 0.19] | -0.003 | 0.971 |
| SELENOP | 4.436 | 204.924 | 0.000 | [0.1, 0.25] | 0.014 | 0.022 |
| CAMP | -0.530 | 199.745 | 0.597 | [-0.31, 0.18] | -0.006 | 0.930 |
| MMP14 | 0.483 | 203.551 | 0.629 | [-0.18, 0.29] | 0.007 | 0.935 |
| GDI2 | -0.182 | 194.683 | 0.855 | [-0.24, 0.2] | -0.002 | 0.971 |
| SERPINB8 | -0.289 | 188.230 | 0.773 | [-0.33, 0.25] | -0.005 | 0.954 |
| ST13 | 0.506 | 202.629 | 0.613 | [-0.19, 0.32] | 0.007 | 0.935 |
| VASP | 1.212 | 188.409 | 0.227 | [-0.15, 0.63] | 0.027 | 0.885 |
| DNM2 | 0.091 | 205.930 | 0.928 | [-0.17, 0.18] | 0.001 | 0.979 |
| KNTC1 | -0.380 | 186.963 | 0.704 | [-0.4, 0.27] | -0.007 | 0.946 |
| CDK9 | -1.004 | 191.735 | 0.317 | [-0.43, 0.14] | -0.015 | 0.892 |
| BCAM | 0.233 | 203.844 | 0.816 | [-0.13, 0.17] | 0.002 | 0.965 |
| CCT8 | -0.013 | 195.811 | 0.989 | [-0.24, 0.24] | 0.000 | 0.999 |
| RAB7A | -0.584 | 200.348 | 0.560 | [-0.32, 0.17] | -0.008 | 0.925 |
| FABP6 | 0.439 | 151.906 | 0.661 | [-0.14, 0.22] | 0.005 | 0.938 |
| DUSP3 | 1.101 | 193.945 | 0.272 | [-0.16, 0.56] | 0.024 | 0.888 |
| HSD17B4 | 0.034 | 182.285 | 0.973 | [-0.19, 0.2] | 0.000 | 0.997 |
| PSMD7 | -0.976 | 196.478 | 0.330 | [-0.2, 0.07] | -0.010 | 0.892 |
| APLP1 | -2.288 | 157.999 | 0.023 | [-0.3, -0.02] | -0.020 | 0.740 |
| CLCNKA | 1.017 | 191.463 | 0.311 | [-0.09, 0.27] | 0.009 | 0.892 |
| LUM | -0.574 | 204.263 | 0.567 | [-0.12, 0.07] | -0.002 | 0.926 |
| PGD | -1.293 | 180.699 | 0.198 | [-0.49, 0.1] | -0.022 | 0.885 |
| ARHGDIA | -0.705 | 205.757 | 0.482 | [-0.43, 0.2] | -0.013 | 0.918 |
| ARHGDIB | -0.615 | 205.923 | 0.539 | [-0.66, 0.35] | -0.017 | 0.924 |
| NDST1 | -0.176 | 203.121 | 0.860 | [-0.31, 0.26] | -0.003 | 0.971 |
| CAPZA1 | 0.092 | 199.297 | 0.927 | [-0.4, 0.44] | 0.002 | 0.979 |
| BLVRA | -0.467 | 196.920 | 0.641 | [-0.39, 0.24] | -0.009 | 0.935 |
| ACLY | 0.710 | 205.883 | 0.479 | [-0.19, 0.4] | 0.014 | 0.918 |
| CTSC | 0.933 | 200.892 | 0.352 | [-0.1, 0.28] | 0.010 | 0.900 |
| CRISP3 | 0.978 | 195.682 | 0.329 | [-0.06, 0.18] | 0.005 | 0.892 |
| CACNA2D1 | 0.191 | 205.734 | 0.849 | [-0.12, 0.15] | 0.001 | 0.971 |
| YARS1 | 0.009 | 192.334 | 0.993 | [-0.32, 0.32] | 0.000 | 0.999 |
| USP14 | -1.166 | 197.235 | 0.245 | [-0.47, 0.12] | -0.020 | 0.885 |
| HSPA2 | -1.180 | 202.988 | 0.240 | [-0.42, 0.11] | -0.016 | 0.885 |
| RAD23A | 1.935 | 204.973 | 0.054 | [-0., 0.42] | 0.026 | 0.810 |
| RAD23B | 1.564 | 197.056 | 0.119 | [-0.06, 0.51] | 0.027 | 0.830 |
| EPHB4 | 0.115 | 203.909 | 0.909 | [-0.14, 0.16] | 0.001 | 0.976 |
| EPHA4 | 1.249 | 204.313 | 0.213 | [-0.05, 0.24] | 0.011 | 0.885 |
| NAGLU | -1.589 | 199.215 | 0.114 | [-0.3, 0.03] | -0.013 | 0.830 |
| AK2 | -0.677 | 205.790 | 0.499 | [-0.26, 0.13] | -0.007 | 0.918 |
| NAPA | -1.507 | 193.221 | 0.133 | [-0.44, 0.06] | -0.022 | 0.833 |
| SLURP1 | -0.550 | 185.641 | 0.583 | [-0.38, 0.21] | -0.009 | 0.930 |
| APOC4 | -0.709 | 194.745 | 0.479 | [-0.39, 0.18] | -0.009 | 0.918 |
| PLTP | -1.451 | 205.471 | 0.148 | [-0.23, 0.03] | -0.009 | 0.833 |
| VCP | -1.350 | 195.784 | 0.179 | [-0.59, 0.11] | -0.027 | 0.860 |
| MFAP4 | -0.730 | 194.685 | 0.466 | [-0.38, 0.17] | -0.012 | 0.918 |
| INHBC | -1.185 | 203.967 | 0.237 | [-0.17, 0.04] | -0.006 | 0.885 |
| MANF | -1.036 | 168.476 | 0.302 | [-0.42, 0.13] | -0.018 | 0.892 |
| MTTP | -0.536 | 186.275 | 0.592 | [-0.2, 0.11] | -0.006 | 0.930 |
| NAP1L1 | -0.227 | 195.214 | 0.821 | [-0.3, 0.24] | -0.004 | 0.967 |
| LAMB2 | -0.515 | 205.926 | 0.607 | [-0.14, 0.08] | -0.003 | 0.935 |
| CDH6 | -0.387 | 200.639 | 0.699 | [-0.14, 0.1] | -0.002 | 0.946 |
| CDH11 | -2.655 | 198.028 | 0.009 | [-0.33, -0.05] | -0.023 | 0.705 |
| CDH13 | 1.131 | 205.824 | 0.259 | [-0.05, 0.17] | 0.006 | 0.885 |
| NPEPPS | -1.437 | 186.766 | 0.152 | [-0.33, 0.05] | -0.018 | 0.833 |
| SUMO3 | 1.118 | 205.435 | 0.265 | [-0.14, 0.49] | 0.020 | 0.886 |
| FCGRT | -2.145 | 204.680 | 0.033 | [-0.48, -0.02] | -0.028 | 0.808 |
| ITGA1 | 1.230 | 205.163 | 0.220 | [-0.1, 0.44] | 0.019 | 0.885 |
| INHBE | -0.719 | 201.906 | 0.473 | [-0.29, 0.14] | -0.009 | 0.918 |
| ANTXR2 | 0.267 | 176.193 | 0.790 | [-0.14, 0.19] | 0.003 | 0.954 |
| MTPN | -0.096 | 203.708 | 0.924 | [-0.46, 0.41] | -0.002 | 0.979 |
| DEFA1 | -0.317 | 184.591 | 0.752 | [-0.22, 0.16] | -0.002 | 0.951 |
| ARPC4 | -1.899 | 189.082 | 0.059 | [-0.77, 0.01] | -0.040 | 0.811 |
| DEFB1 | 0.535 | 201.668 | 0.593 | [-0.2, 0.36] | 0.011 | 0.930 |
| TPI1 | -0.607 | 203.061 | 0.545 | [-0.5, 0.27] | -0.012 | 0.924 |
| MYL6 | 0.758 | 187.762 | 0.449 | [-0.17, 0.38] | 0.013 | 0.918 |
| ACTB | -0.969 | 203.616 | 0.334 | [-0.88, 0.3] | -0.028 | 0.892 |
| EIF4A1 | -1.098 | 201.239 | 0.274 | [-0.58, 0.16] | -0.023 | 0.888 |
| PRPS1 | -0.300 | 205.481 | 0.764 | [-0.43, 0.32] | -0.006 | 0.954 |
| PSMA6 | -1.186 | 198.384 | 0.237 | [-0.4, 0.1] | -0.018 | 0.885 |
| DSTN | -1.374 | 190.196 | 0.171 | [-0.46, 0.08] | -0.022 | 0.857 |
| UBE2D3 | -0.775 | 197.490 | 0.439 | [-0.4, 0.17] | -0.013 | 0.918 |
| UBE2N | -0.779 | 200.486 | 0.437 | [-0.39, 0.17] | -0.012 | 0.918 |
| RAB14 | -2.397 | 166.223 | 0.018 | [-0.42, -0.04] | -0.027 | 0.740 |
| ACTR3 | -1.168 | 201.214 | 0.244 | [-0.69, 0.18] | -0.030 | 0.885 |
| ACTR2 | -0.688 | 197.216 | 0.492 | [-0.45, 0.22] | -0.013 | 0.918 |
| ARF3 | 0.001 | 198.739 | 1.000 | [-0.37, 0.37] | 0.000 | 1.000 |
| RAP1B | -0.920 | 203.461 | 0.358 | [-0.42, 0.15] | -0.015 | 0.908 |
| RHOA | -1.463 | 188.021 | 0.145 | [-0.44, 0.06] | -0.023 | 0.833 |
| LYZ | -0.460 | 200.286 | 0.646 | [-0.15, 0.09] | -0.002 | 0.935 |
| B2M | -1.963 | 199.879 | 0.051 | [-0.24, 0] | -0.009 | 0.808 |
| NPC2 | 0.153 | 199.749 | 0.878 | [-0.09, 0.11] | 0.001 | 0.973 |
| NUTF2 | -1.122 | 202.655 | 0.263 | [-0.46, 0.13] | -0.018 | 0.885 |
| HNRNPK | -2.333 | 190.887 | 0.021 | [-0.4, -0.03] | -0.028 | 0.740 |
| YWHAG | -2.074 | 196.112 | 0.039 | [-0.79, -0.02] | -0.048 | 0.808 |
| PPP1CA | 0.658 | 198.033 | 0.512 | [-0.13, 0.25] | 0.009 | 0.924 |
| YWHAE | -0.620 | 203.775 | 0.536 | [-0.45, 0.24] | -0.012 | 0.924 |
| SNRPD3 | 2.267 | 153.892 | 0.025 | [0.06, 0.83] | 0.063 | 0.740 |
| TMSB4X | 0.947 | 203.872 | 0.345 | [-0.13, 0.36] | 0.013 | 0.897 |
| RAB11A | 0.713 | 204.934 | 0.477 | [-0.3, 0.63] | 0.020 | 0.918 |
| H4C1 | 0.577 | 196.921 | 0.565 | [-0.27, 0.49] | 0.013 | 0.926 |
| RAB1A | 0.935 | 205.998 | 0.351 | [-0.13, 0.36] | 0.014 | 0.900 |
| RAN | -0.353 | 202.460 | 0.725 | [-0.37, 0.26] | -0.006 | 0.946 |
| PPIA | 0.178 | 205.804 | 0.859 | [-0.36, 0.43] | 0.003 | 0.971 |
| FKBP1A | 0.540 | 198.444 | 0.590 | [-0.29, 0.5] | 0.012 | 0.930 |
| GRB2 | -1.691 | 127.676 | 0.093 | [-0.58, 0.05] | -0.032 | 0.830 |
| YWHAZ | -0.117 | 203.333 | 0.907 | [-0.62, 0.55] | -0.003 | 0.976 |
| EIF5A | -0.293 | 200.091 | 0.770 | [-0.45, 0.33] | -0.007 | 0.954 |
| ACTG1 | -0.722 | 204.670 | 0.471 | [-0.67, 0.31] | -0.015 | 0.918 |
| TPM4 | 0.585 | 205.804 | 0.559 | [-0.41, 0.76] | 0.016 | 0.925 |
| ACTC1 | -0.290 | 203.936 | 0.772 | [-0.63, 0.47] | -0.008 | 0.954 |
| UBE2L3 | 1.050 | 205.742 | 0.295 | [-0.16, 0.52] | 0.020 | 0.888 |
| EEF1A1 | -1.083 | 201.921 | 0.280 | [-0.5, 0.15] | -0.020 | 0.888 |
| ACTA1 | -0.189 | 205.957 | 0.851 | [-0.46, 0.38] | -0.004 | 0.971 |
| TUBA1B | 0.812 | 202.204 | 0.418 | [-0.28, 0.67] | 0.022 | 0.918 |
| TUBA4A | -0.012 | 178.060 | 0.990 | [-0.26, 0.26] | 0.000 | 0.999 |
| TUBB4B | -0.507 | 176.694 | 0.613 | [-0.33, 0.2] | -0.009 | 0.935 |
| PAFAH1B2 | -0.642 | 204.771 | 0.522 | [-0.44, 0.22] | -0.012 | 0.924 |
| HBB | -0.442 | 179.042 | 0.659 | [-0.52, 0.33] | -0.007 | 0.937 |
| NOMO3 | -1.921 | 191.297 | 0.056 | [-0.21, 0] | -0.012 | 0.810 |
| HBG1 | 0.646 | 189.557 | 0.519 | [-0.32, 0.64] | 0.017 | 0.924 |
| HBG2 | 0.600 | 179.712 | 0.550 | [-0.32, 0.6] | 0.009 | 0.925 |
| HBA1; | -0.456 | 178.804 | 0.649 | [-0.53, 0.33] | -0.007 | 0.935 |
| SIRPA | -0.609 | 163.650 | 0.544 | [-0.22, 0.12] | -0.006 | 0.924 |
| CCT2 | 0.027 | 204.740 | 0.978 | [-0.19, 0.2] | 0.000 | 0.998 |
| GSTO1 | -1.135 | 199.099 | 0.258 | [-0.45, 0.12] | -0.015 | 0.885 |
| JAG1 | 0.310 | 171.158 | 0.757 | [-0.14, 0.19] | 0.003 | 0.952 |
| RELN | -0.818 | 199.314 | 0.414 | [-0.14, 0.06] | -0.004 | 0.918 |
| IL13RA1 | 0.292 | 205.905 | 0.771 | [-0.15, 0.21] | 0.003 | 0.954 |
| GPLD1 | -1.364 | 191.151 | 0.174 | [-0.2, 0.04] | -0.006 | 0.857 |
| LCN2 | -1.002 | 198.167 | 0.318 | [-0.21, 0.07] | -0.007 | 0.892 |
| S100A12 | -0.588 | 193.824 | 0.558 | [-0.62, 0.34] | -0.016 | 0.925 |
| BASP1 | 0.864 | 196.587 | 0.389 | [-0.13, 0.34] | 0.014 | 0.917 |
| IGLV3 | -0.208 | 197.331 | 0.835 | [-0.35, 0.28] | -0.003 | 0.971 |
| DCD | -0.201 | 203.721 | 0.841 | [-0.22, 0.18] | -0.003 | 0.971 |
| FBLN2 | -0.491 | 187.560 | 0.624 | [-0.23, 0.14] | -0.006 | 0.935 |
| HSPG2 | -0.400 | 204.828 | 0.690 | [-0.1, 0.07] | -0.002 | 0.946 |
| EFNB1 | -2.024 | 173.316 | 0.044 | [-0.59, -0.01] | -0.034 | 0.808 |
| CYCS | -1.251 | 202.106 | 0.213 | [-0.36, 0.08] | -0.016 | 0.885 |
| MPP1 | -1.555 | 203.982 | 0.122 | [-0.28, 0.03] | -0.015 | 0.830 |
| MAT1A | -0.402 | 199.365 | 0.688 | [-0.16, 0.11] | -0.004 | 0.946 |
| SORD | -0.863 | 205.913 | 0.389 | [-0.27, 0.1] | -0.010 | 0.917 |
| HNRNPU | -0.115 | 202.771 | 0.909 | [-0.27, 0.24] | -0.001 | 0.976 |
| MYL7 | 0.599 | 205.613 | 0.550 | [-0.12, 0.23] | 0.006 | 0.925 |
| CTBS | 0.887 | 205.984 | 0.376 | [-0.05, 0.13] | 0.004 | 0.917 |
| FABP5 | 0.103 | 204.658 | 0.918 | [-0.22, 0.24] | 0.001 | 0.979 |
| CAP1 | -1.022 | 198.712 | 0.308 | [-0.92, 0.29] | -0.033 | 0.892 |
| IL1RL1 | 1.180 | 203.752 | 0.239 | [-0.07, 0.29] | 0.012 | 0.885 |
| PFKP | -0.476 | 205.800 | 0.634 | [-0.21, 0.13] | -0.005 | 0.935 |
| TAGLN | 2.419 | 192.954 | 0.016 | [0.04, 0.4] | 0.029 | 0.740 |
| TNFRSF17 | 1.225 | 192.240 | 0.222 | [-0.06, 0.27] | 0.015 | 0.885 |
| DSG1 | 0.333 | 201.173 | 0.740 | [-0.24, 0.33] | 0.006 | 0.946 |
| DSC2 | -0.711 | 195.432 | 0.478 | [-0.23, 0.11] | -0.007 | 0.918 |
| GUCA2A | -0.451 | 194.775 | 0.653 | [-0.25, 0.16] | -0.006 | 0.935 |
| TEK | 0.470 | 185.337 | 0.639 | [-0.14, 0.23] | 0.006 | 0.935 |
| NUCB1 | 0.758 | 204.490 | 0.449 | [-0.12, 0.28] | 0.009 | 0.918 |
| CFHR3 | -1.386 | 205.933 | 0.167 | [-0.13, 0.02] | -0.004 | 0.857 |
| ACY1 | -0.497 | 198.298 | 0.620 | [-0.34, 0.21] | -0.008 | 0.935 |
| TGFBR3 | -0.842 | 205.995 | 0.401 | [-0.17, 0.07] | -0.006 | 0.917 |
| CFHR1 | 0.747 | 202.120 | 0.456 | [-0.05, 0.1] | 0.002 | 0.918 |
| GBE1 | -1.329 | 201.497 | 0.185 | [-0.38, 0.07] | -0.019 | 0.863 |
| KRT17 | 0.623 | 202.965 | 0.534 | [-0.24, 0.46] | 0.012 | 0.924 |
| NOTCH2 | 0.405 | 200.806 | 0.686 | [-0.05, 0.08] | 0.001 | 0.946 |
| HGFAC | -0.338 | 183.613 | 0.736 | [-0.12, 0.09] | -0.001 | 0.946 |
| GLO1 | -1.206 | 193.055 | 0.229 | [-0.44, 0.11] | -0.020 | 0.885 |
| YWHAH | -2.026 | 186.629 | 0.044 | [-0.57, -0.01] | -0.033 | 0.808 |
| CALD1 | 0.741 | 204.379 | 0.459 | [-0.17, 0.38] | 0.012 | 0.918 |
| ITIH3 | -1.138 | 201.886 | 0.257 | [-0.21, 0.06] | -0.005 | 0.885 |
| REG3A | -1.259 | 204.487 | 0.209 | [-0.43, 0.09] | -0.020 | 0.885 |
| BTK | 0.577 | 198.636 | 0.564 | [-0.28, 0.51] | 0.016 | 0.926 |
| PSME1 | -0.184 | 204.491 | 0.854 | [-0.25, 0.2] | -0.002 | 0.971 |
| FMOD | -0.564 | 195.878 | 0.573 | [-0.27, 0.15] | -0.007 | 0.928 |
| PRDX1 | -0.283 | 191.167 | 0.777 | [-0.29, 0.21] | -0.004 | 0.954 |
| ENPEP | 0.131 | 203.397 | 0.896 | [-0.13, 0.14] | 0.001 | 0.976 |
| LRP1 | -1.238 | 197.676 | 0.217 | [-0.14, 0.03] | -0.006 | 0.885 |
| ARHGAP1 | -2.078 | 201.505 | 0.039 | [-0.72, -0.02] | -0.045 | 0.808 |
| ACTN3 | 0.028 | 205.820 | 0.977 | [-0.24, 0.25] | 0.000 | 0.998 |
| PCDH1 | 0.567 | 205.162 | 0.572 | [-0.13, 0.23] | 0.006 | 0.926 |
| TGM3 | -1.646 | 178.541 | 0.102 | [-0.38, 0.03] | -0.022 | 0.830 |
| CRYZ | -1.543 | 205.202 | 0.124 | [-0.48, 0.06] | -0.025 | 0.830 |
| DDR1 | -0.679 | 205.396 | 0.498 | [-0.24, 0.12] | -0.008 | 0.918 |
| LGALS3BP | -1.668 | 200.686 | 0.097 | [-0.3, 0.03] | -0.012 | 0.830 |
| DMTN | -1.837 | 200.768 | 0.068 | [-0.41, 0.01] | -0.023 | 0.818 |
| DSC1 | -0.131 | 198.532 | 0.896 | [-0.14, 0.13] | -0.001 | 0.976 |
| FGL1 | 0.739 | 205.603 | 0.461 | [-0.17, 0.37] | 0.010 | 0.918 |
| SIGLEC14 | -2.789 | 205.599 | 0.006 | [-0.44, -0.08] | -0.031 | 0.705 |
| AHNAK | 0.404 | 204.058 | 0.687 | [-0.35, 0.53] | 0.013 | 0.946 |
| MGAT2 | -0.119 | 193.323 | 0.905 | [-0.18, 0.16] | -0.001 | 0.976 |
| GALNT2 | -0.634 | 202.744 | 0.527 | [-0.13, 0.07] | -0.003 | 0.924 |
| GALNT1 | 1.128 | 204.661 | 0.261 | [-0.08, 0.3] | 0.014 | 0.885 |
| BST1 | 0.268 | 201.797 | 0.789 | [-0.12, 0.15] | 0.002 | 0.954 |
| ARHGEF5 | 0.950 | 205.902 | 0.343 | [-0.16, 0.45] | 0.013 | 0.897 |
| EFEMP1 | 0.601 | 201.773 | 0.549 | [-0.07, 0.13] | 0.002 | 0.925 |
| FSTL1 | -1.586 | 205.499 | 0.114 | [-0.18, 0.02] | -0.008 | 0.830 |
| CNTN1 | 1.467 | 202.760 | 0.144 | [-0.02, 0.17] | 0.006 | 0.833 |
| CDH17 | 0.347 | 190.101 | 0.729 | [-0.17, 0.25] | 0.005 | 0.946 |
| MERTK | -0.680 | 182.711 | 0.498 | [-0.19, 0.09] | -0.005 | 0.918 |
| GRIN2A | -1.595 | 183.133 | 0.112 | [-0.32, 0.03] | -0.015 | 0.830 |
| FAP | 0.476 | 203.801 | 0.635 | [-0.12, 0.2] | 0.004 | 0.935 |
| LMAN2 | -0.872 | 204.658 | 0.384 | [-0.15, 0.06] | -0.005 | 0.917 |
| PTPRJ | 0.310 | 202.830 | 0.757 | [-0.08, 0.11] | 0.002 | 0.952 |
| PLA2G7 | 0.613 | 202.533 | 0.541 | [-0.12, 0.22] | 0.005 | 0.924 |
| LCP2 | -0.361 | 201.169 | 0.718 | [-0.36, 0.25] | -0.006 | 0.946 |
| SPP2 | -0.130 | 194.626 | 0.897 | [-0.31, 0.27] | -0.002 | 0.976 |
| MMRN1 | 1.404 | 197.040 | 0.162 | [-0.09, 0.51] | 0.023 | 0.849 |
| PAPPA | -1.067 | 200.649 | 0.287 | [-0.67, 0.2] | -0.026 | 0.888 |
| SELENBP1 | -0.933 | 184.320 | 0.352 | [-0.29, 0.1] | -0.009 | 0.900 |
| CHIT1 | 0.128 | 198.821 | 0.899 | [-0.21, 0.24] | 0.002 | 0.976 |
| NME3 | -0.808 | 199.994 | 0.420 | [-0.33, 0.14] | -0.011 | 0.918 |
| SEMA3F | -1.330 | 195.439 | 0.185 | [-0.19, 0.04] | -0.009 | 0.863 |
| PTK7 | 0.429 | 189.284 | 0.668 | [-0.11, 0.17] | 0.004 | 0.941 |
| PTPRS | -0.190 | 204.436 | 0.849 | [-0.12, 0.1] | -0.001 | 0.971 |
| MFAP5 | 0.985 | 197.760 | 0.326 | [-0.13, 0.38] | 0.016 | 0.892 |
| UBE2V1 | -0.681 | 205.779 | 0.497 | [-0.3, 0.14] | -0.009 | 0.918 |
| ILK | 0.801 | 205.566 | 0.424 | [-0.19, 0.44] | 0.014 | 0.918 |
| MSLN | -0.854 | 191.372 | 0.394 | [-0.32, 0.13] | -0.009 | 0.917 |
| LSAMP | 0.637 | 203.213 | 0.525 | [-0.11, 0.21] | 0.005 | 0.924 |
| ART3 | -0.968 | 204.213 | 0.334 | [-0.21, 0.07] | -0.007 | 0.892 |
| IQGAP2 | -0.205 | 201.758 | 0.838 | [-0.2, 0.16] | -0.002 | 0.971 |
| GFUS | 0.660 | 205.002 | 0.510 | [-0.13, 0.27] | 0.008 | 0.924 |
| FHL1 | 1.999 | 195.905 | 0.047 | [0., 0.52] | 0.030 | 0.808 |
| ALCAM | -0.982 | 196.464 | 0.327 | [-0.11, 0.04] | -0.003 | 0.892 |
| APOF | 0.065 | 185.722 | 0.948 | [-0.19, 0.2] | 0.001 | 0.989 |
| ENPP2 | -0.105 | 204.307 | 0.916 | [-0.12, 0.1] | -0.001 | 0.979 |
| PKP1 | -0.990 | 203.904 | 0.323 | [-0.25, 0.08] | -0.011 | 0.892 |
| BLMH | -0.633 | 204.361 | 0.527 | [-0.24, 0.12] | -0.006 | 0.924 |
| RAPGEF1 | -0.410 | 190.357 | 0.682 | [-0.25, 0.16] | -0.006 | 0.946 |
| CCIN | -0.780 | 200.497 | 0.436 | [-0.24, 0.1] | -0.007 | 0.918 |
| COTL1 | -0.512 | 201.834 | 0.609 | [-0.64, 0.38] | -0.014 | 0.935 |
| NID2 | 1.438 | 182.246 | 0.152 | [-0.06, 0.4] | 0.021 | 0.833 |
| DAG1 | -3.154 | 205.572 | 0.002 | [-0.31, -0.07] | -0.020 | 0.578 |
| DSG2 | 0.082 | 205.854 | 0.935 | [-0.09, 0.1] | 0.000 | 0.983 |
| SCARF1 | 1.995 | 178.631 | 0.048 | [0., 0.29] | 0.023 | 0.808 |
| MLEC | 0.583 | 176.892 | 0.560 | [-0.08, 0.14] | 0.004 | 0.925 |
| EBI3 | 0.194 | 187.898 | 0.846 | [-0.14, 0.17] | 0.002 | 0.971 |
| EIF4A2 | -0.387 | 170.146 | 0.699 | [-0.19, 0.13] | -0.004 | 0.946 |
| CTTN | 0.332 | 187.668 | 0.741 | [-0.25, 0.35] | 0.006 | 0.946 |
| FGL2 | -2.261 | 204.189 | 0.025 | [-0.42, -0.03] | -0.024 | 0.740 |
| FLNC | 0.832 | 197.110 | 0.406 | [-0.11, 0.28] | 0.010 | 0.918 |
| GAS6 | -1.453 | 194.263 | 0.148 | [-0.23, 0.04] | -0.013 | 0.833 |
| NEDD9 | -0.471 | 197.404 | 0.638 | [-0.4, 0.25] | -0.006 | 0.935 |
| SPARCL1 | -0.040 | 205.863 | 0.968 | [-0.09, 0.08] | 0.000 | 0.997 |
| FAT1 | -1.334 | 161.725 | 0.184 | [-0.77, 0.15] | -0.037 | 0.863 |
| HABP2 | -0.769 | 206.000 | 0.443 | [-0.1, 0.04] | -0.002 | 0.918 |
| KRT33B | -0.097 | 193.417 | 0.923 | [-0.23, 0.21] | -0.001 | 0.979 |
| PDIA5 | -0.697 | 202.217 | 0.487 | [-0.42, 0.2] | -0.014 | 0.918 |
| SEMA3A | -0.780 | 205.635 | 0.436 | [-0.19, 0.08] | -0.007 | 0.918 |
| DSC3 | -0.083 | 205.992 | 0.934 | [-0.12, 0.11] | 0.000 | 0.983 |
| IHH | -0.262 | 198.438 | 0.793 | [-0.22, 0.17] | -0.003 | 0.956 |
| ITIH4 | 0.138 | 199.833 | 0.890 | [-0.05, 0.05] | 0.000 | 0.976 |
| GANAB | -1.838 | 192.730 | 0.068 | [-0.36, 0.01] | -0.021 | 0.818 |
| LTBP1 | 1.656 | 204.803 | 0.099 | [-0.05, 0.61] | 0.030 | 0.830 |
| LTBP2 | 1.235 | 201.310 | 0.218 | [-0.05, 0.24] | 0.012 | 0.885 |
| LASP1 | -1.242 | 179.347 | 0.216 | [-0.47, 0.11] | -0.021 | 0.885 |
| GPNMB | -0.839 | 202.194 | 0.402 | [-0.17, 0.07] | -0.005 | 0.917 |
| KPNB1 | -0.102 | 204.060 | 0.919 | [-0.3, 0.27] | -0.002 | 0.979 |
| SEPTIN2 | -1.057 | 186.692 | 0.292 | [-0.27, 0.08] | -0.011 | 0.888 |
| POSTN | 0.288 | 198.184 | 0.774 | [-0.13, 0.18] | 0.002 | 0.954 |
| PDIA6 | -1.319 | 195.165 | 0.189 | [-0.48, 0.1] | -0.024 | 0.867 |
| PCOLCE | -1.812 | 204.911 | 0.071 | [-0.22, 0.01] | -0.009 | 0.818 |
| PON3 | 1.236 | 200.846 | 0.218 | [-0.06, 0.25] | 0.009 | 0.885 |
| PTGES3 | 0.378 | 189.322 | 0.706 | [-0.23, 0.34] | 0.006 | 0.946 |
| NECTIN1 | -0.020 | 203.937 | 0.984 | [-0.09, 0.09] | 0.000 | 0.998 |
| PTPA | -0.609 | 203.972 | 0.543 | [-0.38, 0.2] | -0.011 | 0.924 |
| PTPRK | -0.507 | 200.733 | 0.613 | [-0.17, 0.1] | -0.004 | 0.935 |
| KRT31 | 1.084 | 196.634 | 0.280 | [-0.15, 0.52] | 0.022 | 0.888 |
| PCBP1 | -1.395 | 189.514 | 0.165 | [-0.66, 0.11] | -0.032 | 0.853 |
| PCBP2 | 1.071 | 205.474 | 0.286 | [-0.17, 0.56] | 0.024 | 0.888 |
| RSU1 | -1.007 | 194.794 | 0.315 | [-0.64, 0.21] | -0.022 | 0.892 |
| FCN2 | 1.489 | 153.777 | 0.138 | [-0.05, 0.35] | 0.018 | 0.833 |
| CDSN | 0.234 | 193.933 | 0.815 | [-0.27, 0.34] | 0.004 | 0.965 |
| MAPRE2 | -1.538 | 202.633 | 0.126 | [-0.44, 0.05] | -0.024 | 0.830 |
| TGFBI | -0.853 | 205.577 | 0.394 | [-0.13, 0.05] | -0.003 | 0.917 |
| MAPRE1 | 0.303 | 204.236 | 0.763 | [-0.31, 0.42] | 0.007 | 0.953 |
| MYLK | -1.335 | 189.903 | 0.183 | [-0.33, 0.06] | -0.017 | 0.863 |
| CD226 | -0.140 | 180.112 | 0.889 | [-0.16, 0.14] | -0.002 | 0.976 |
| CST6 | 0.408 | 200.180 | 0.684 | [-0.11, 0.17] | 0.003 | 0.946 |
| NEDD8 | -1.469 | 192.670 | 0.143 | [-0.48, 0.07] | -0.028 | 0.833 |
| ADIPOQ | 1.039 | 203.076 | 0.300 | [-0.11, 0.35] | 0.010 | 0.891 |
| ATP6AP1 | 1.285 | 156.340 | 0.201 | [-0.12, 0.55] | 0.027 | 0.885 |
| ZYX | 0.263 | 202.489 | 0.793 | [-0.58, 0.76] | 0.010 | 0.955 |
| SEPTIN7 | 0.906 | 205.995 | 0.366 | [-0.1, 0.26] | 0.009 | 0.913 |
| IGFBP7 | 0.752 | 205.575 | 0.453 | [-0.06, 0.14] | 0.004 | 0.918 |
| PRR4 | -0.041 | 195.669 | 0.967 | [-0.21, 0.2] | 0.000 | 0.997 |
| EXT1 | 0.454 | 198.093 | 0.650 | [-0.08, 0.13] | 0.002 | 0.935 |
| TNXA | 0.297 | 189.582 | 0.767 | [-0.1, 0.14] | 0.002 | 0.954 |
| DDB1 | -0.129 | 201.826 | 0.897 | [-0.19, 0.16] | -0.002 | 0.976 |
| MAPK14 | -0.194 | 205.533 | 0.846 | [-0.21, 0.17] | -0.003 | 0.971 |
| DPYSL2 | -1.586 | 205.947 | 0.114 | [-0.39, 0.04] | -0.022 | 0.830 |
| ECM1 | 1.490 | 202.744 | 0.138 | [-0.02, 0.16] | 0.005 | 0.833 |
| NTRK2 | -0.390 | 172.768 | 0.697 | [-0.28, 0.19] | -0.005 | 0.946 |
| CCL14 | 0.125 | 205.999 | 0.901 | [-0.13, 0.15] | 0.001 | 0.976 |
| FSCN1 | -1.070 | 180.114 | 0.286 | [-0.29, 0.09] | -0.013 | 0.888 |
| CCL15 | -0.096 | 199.880 | 0.924 | [-0.23, 0.21] | -0.001 | 0.979 |
| MAN2A1 | -0.676 | 191.874 | 0.500 | [-0.12, 0.06] | -0.003 | 0.918 |
| HAGH | -1.417 | 202.315 | 0.158 | [-0.7, 0.11] | -0.034 | 0.841 |
| H2AC20 | 1.077 | 204.062 | 0.283 | [-0.1, 0.35] | 0.014 | 0.888 |
| MEP1A | -1.095 | 204.021 | 0.275 | [-0.18, 0.05] | -0.009 | 0.888 |
| UGP2 | -0.691 | 193.274 | 0.490 | [-0.53, 0.25] | -0.016 | 0.918 |
| AOC3 | -0.900 | 194.767 | 0.369 | [-0.17, 0.06] | -0.005 | 0.913 |
| TATDN3 | 1.490 | 187.108 | 0.138 | [-0.07, 0.49] | 0.019 | 0.833 |
| TKFC | -0.155 | 203.651 | 0.877 | [-0.24, 0.21] | -0.002 | 0.973 |
| ZSCAN23 | -1.759 | 172.281 | 0.080 | [-0.23, 0.01] | -0.015 | 0.818 |
| MOGAT2 | 1.422 | 205.228 | 0.156 | [-0.08, 0.47] | 0.021 | 0.841 |
| LGALSL | -0.269 | 187.605 | 0.788 | [-0.51, 0.38] | -0.007 | 0.954 |
| LONRF3 | 0.390 | 196.653 | 0.697 | [-0.22, 0.34] | 0.005 | 0.946 |
| GXYLT1 | -0.671 | 198.480 | 0.503 | [-0.25, 0.12] | -0.008 | 0.921 |
| CDON | -0.938 | 168.201 | 0.349 | [-0.27, 0.09] | -0.010 | 0.900 |
| SVEP1 | -0.340 | 205.234 | 0.734 | [-0.2, 0.14] | -0.003 | 0.946 |
| PABPC1L | 0.256 | 195.769 | 0.798 | [-0.27, 0.36] | 0.005 | 0.960 |
| FBLN7 | 1.569 | 183.054 | 0.118 | [-0.03, 0.29] | 0.016 | 0.830 |
| ACTBL2 | -1.420 | 200.106 | 0.157 | [-0.72, 0.12] | -0.025 | 0.841 |
| SDK2 | 1.153 | 205.894 | 0.250 | [-0.09, 0.34] | 0.013 | 0.885 |
| RUNDC3A | -1.279 | 205.041 | 0.202 | [-0.33, 0.07] | -0.010 | 0.885 |
| FLG2 | -0.397 | 198.625 | 0.692 | [-0.34, 0.23] | -0.007 | 0.946 |
| WDR44 | -1.348 | 168.153 | 0.179 | [-0.36, 0.07] | -0.019 | 0.860 |
| FRMPD1 | -1.142 | 202.124 | 0.255 | [-0.18, 0.05] | -0.005 | 0.885 |
| KPRP | 0.228 | 204.865 | 0.820 | [-0.29, 0.37] | 0.004 | 0.966 |
| KPLCE | -1.112 | 204.433 | 0.268 | [-0.49, 0.14] | -0.018 | 0.888 |
| MYOM3 | -0.691 | 204.456 | 0.490 | [-0.32, 0.15] | -0.010 | 0.918 |
| YOD1 | -1.581 | 177.016 | 0.116 | [-0.19, 0.02] | -0.011 | 0.830 |
| PEAR1 | 1.272 | 204.495 | 0.205 | [-0.04, 0.19] | 0.008 | 0.885 |
| CD276 | -0.378 | 190.889 | 0.706 | [-0.21, 0.14] | -0.004 | 0.946 |
| DMKN | -0.370 | 203.902 | 0.712 | [-0.27, 0.18] | -0.006 | 0.946 |
| VASN | -0.397 | 205.830 | 0.692 | [-0.1, 0.07] | -0.001 | 0.946 |
| PM20D1 | -0.788 | 189.135 | 0.432 | [-0.35, 0.15] | -0.010 | 0.918 |
| TWF2 | -1.770 | 202.803 | 0.078 | [-0.46, 0.02] | -0.026 | 0.818 |
| KRT80 | -0.285 | 206.000 | 0.776 | [-0.18, 0.14] | -0.003 | 0.954 |
| NIPBL | 0.523 | 200.463 | 0.601 | [-0.37, 0.64] | 0.018 | 0.932 |
| ERAP2 | 0.860 | 199.288 | 0.391 | [-0.18, 0.46] | 0.014 | 0.917 |
| NOTUM | -1.761 | 195.912 | 0.080 | [-0.5, 0.03] | -0.031 | 0.818 |
| LILRA6 | -0.137 | 202.996 | 0.891 | [-0.14, 0.12] | -0.001 | 0.976 |
| DNM1P34 | 0.784 | 189.597 | 0.434 | [-0.13, 0.31] | 0.009 | 0.918 |
| APOA5 | -0.993 | 190.259 | 0.322 | [-0.28, 0.09] | -0.011 | 0.892 |
| ADGRD1 | -1.686 | 137.331 | 0.094 | [-0.33, 0.03] | -0.015 | 0.830 |
| CSPG4 | -0.172 | 202.613 | 0.863 | [-0.15, 0.13] | -0.001 | 0.971 |
| SBSN | -0.856 | 205.836 | 0.393 | [-0.22, 0.09] | -0.008 | 0.917 |
| PLXDC2 | 1.266 | 202.530 | 0.207 | [-0.02, 0.11] | 0.004 | 0.885 |
| PI16 | 0.686 | 205.462 | 0.494 | [-0.1, 0.2] | 0.005 | 0.918 |
| SEZ6L2 | 0.065 | 192.889 | 0.948 | [-0.12, 0.12] | 0.000 | 0.989 |
| ANGPTL8 | -0.390 | 203.376 | 0.697 | [-0.27, 0.18] | -0.005 | 0.946 |
| PAMR1 | 1.178 | 174.280 | 0.241 | [-0.07, 0.28] | 0.012 | 0.885 |
| NPNT | -0.033 | 197.856 | 0.974 | [-0.15, 0.15] | 0.000 | 0.997 |
| LRRN1 | -0.757 | 199.329 | 0.450 | [-0.2, 0.09] | -0.008 | 0.918 |
| ADAMTSL4 | 1.045 | 202.560 | 0.297 | [-0.06, 0.2] | 0.007 | 0.888 |
| CHRDL2 | -0.438 | 204.704 | 0.662 | [-0.25, 0.16] | -0.006 | 0.938 |
| NAPRT | -0.287 | 187.199 | 0.774 | [-0.19, 0.14] | -0.003 | 0.954 |
| CD109 | -0.398 | 201.619 | 0.691 | [-0.13, 0.09] | -0.002 | 0.946 |
| BNC2 | 1.840 | 203.204 | 0.067 | [-0.01, 0.43] | 0.016 | 0.818 |
| DNAH12 | -0.699 | 205.827 | 0.486 | [-0.29, 0.14] | -0.008 | 0.918 |
| QSOX2 | -0.642 | 165.399 | 0.522 | [-0.29, 0.15] | -0.009 | 0.924 |
| TMTC3 | 0.650 | 205.627 | 0.517 | [-0.11, 0.22] | 0.006 | 0.924 |
| UNC13D | 0.762 | 169.261 | 0.447 | [-0.22, 0.49] | 0.018 | 0.918 |
| ADAMTS13 | 2.266 | 192.872 | 0.025 | [0.01, 0.19] | 0.009 | 0.740 |
| CCDC80 | -0.448 | 205.920 | 0.655 | [-0.19, 0.12] | -0.004 | 0.936 |
| SND1 | -0.244 | 186.375 | 0.807 | [-0.31, 0.24] | -0.004 | 0.962 |
| CYFIP1 | -0.274 | 191.254 | 0.784 | [-0.12, 0.09] | -0.002 | 0.954 |
| CHST3 | 0.121 | 205.994 | 0.904 | [-0.17, 0.19] | 0.001 | 0.976 |
| KRT74 | -0.024 | 200.699 | 0.981 | [-0.33, 0.32] | 0.000 | 0.998 |
| NEGR1 | -1.063 | 205.929 | 0.289 | [-0.19, 0.06] | -0.007 | 0.888 |
| KRT28 | -0.922 | 203.586 | 0.358 | [-0.23, 0.09] | -0.007 | 0.907 |
| KRT26 | 2.186 | 197.174 | 0.030 | [0.08, 1.58] | 0.116 | 0.808 |
| DCXR | -0.967 | 205.999 | 0.335 | [-0.43, 0.15] | -0.017 | 0.892 |
| ARHGAP22 | 0.096 | 205.138 | 0.924 | [-0.42, 0.46] | 0.002 | 0.979 |
| SDK1 | -0.543 | 205.719 | 0.588 | [-0.25, 0.14] | -0.006 | 0.930 |
| KRT77 | 1.053 | 201.545 | 0.294 | [-0.24, 0.8] | 0.023 | 0.888 |
| ABI3BP | 0.605 | 203.262 | 0.546 | [-0.08, 0.15] | 0.003 | 0.925 |
| MEGF8 | 0.882 | 205.094 | 0.379 | [-0.04, 0.11] | 0.003 | 0.917 |
| B3GNT8 | -0.289 | 199.309 | 0.773 | [-0.15, 0.11] | -0.002 | 0.954 |
| GALNT5 | 0.761 | 182.926 | 0.448 | [-0.14, 0.31] | 0.011 | 0.918 |
| GALNT7 | -0.757 | 205.631 | 0.450 | [-0.24, 0.11] | -0.008 | 0.918 |
| ADGRG6 | -1.649 | 204.365 | 0.101 | [-0.18, 0.02] | -0.008 | 0.830 |
| GALNT10 | 1.104 | 171.662 | 0.271 | [-0.11, 0.4] | 0.017 | 0.888 |
| ADAMTSL2 | 0.049 | 192.848 | 0.961 | [-0.12, 0.12] | 0.000 | 0.996 |
| SERPINA11 | 0.677 | 192.786 | 0.499 | [-0.1, 0.2] | 0.005 | 0.918 |
| OAF | -1.765 | 200.753 | 0.079 | [-0.19, 0.01] | -0.010 | 0.818 |
| RTN4RL2 | -0.542 | 205.887 | 0.588 | [-0.17, 0.1] | -0.004 | 0.930 |
| ITIH5 | -0.848 | 184.169 | 0.397 | [-0.44, 0.17] | -0.012 | 0.917 |
| FERMT3 | -0.727 | 200.719 | 0.468 | [-1.07, 0.49] | -0.028 | 0.918 |
| CD163 | -0.614 | 205.918 | 0.540 | [-0.19, 0.1] | -0.004 | 0.924 |
| TAX1BP1 | 2.065 | 200.656 | 0.040 | [0.01, 0.63] | 0.027 | 0.808 |
| CAND1 | -1.391 | 204.551 | 0.166 | [-0.36, 0.06] | -0.018 | 0.855 |
| LRP11 | -0.354 | 205.201 | 0.724 | [-0.2, 0.14] | -0.005 | 0.946 |
| PKHD1L1 | 2.254 | 205.104 | 0.025 | [0.02, 0.33] | 0.021 | 0.740 |
| LSR | -1.705 | 203.213 | 0.090 | [-0.38, 0.03] | -0.023 | 0.830 |
| TMEM25 | -1.782 | 176.774 | 0.076 | [-0.39, 0.02] | -0.026 | 0.818 |
| PRAG1 | -0.437 | 186.215 | 0.663 | [-0.18, 0.12] | -0.004 | 0.938 |
| TREML1 | 0.237 | 176.569 | 0.813 | [-0.29, 0.37] | 0.004 | 0.964 |
| HRNR | 0.712 | 205.171 | 0.477 | [-0.18, 0.38] | 0.011 | 0.918 |
| PLXDC1 | 1.485 | 205.762 | 0.139 | [-0.03, 0.25] | 0.012 | 0.833 |
| CBLN2 | 0.495 | 205.894 | 0.621 | [-0.21, 0.35] | 0.008 | 0.935 |
| CILP2 | -0.292 | 197.639 | 0.770 | [-0.25, 0.18] | -0.003 | 0.954 |
| AEBP1 | -0.345 | 149.554 | 0.730 | [-0.12, 0.08] | -0.003 | 0.946 |
| CDKL3 | -0.318 | 181.916 | 0.751 | [-0.18, 0.13] | -0.003 | 0.951 |
| SERPINA12 | -0.020 | 191.481 | 0.984 | [-0.27, 0.27] | 0.000 | 0.998 |
| ADGRA3 | 1.361 | 192.174 | 0.175 | [-0.05, 0.28] | 0.016 | 0.857 |
| SFTPA2 | 0.989 | 180.404 | 0.324 | [-0.14, 0.41] | 0.014 | 0.892 |
| CNTN4 | -0.306 | 205.154 | 0.760 | [-0.11, 0.08] | -0.002 | 0.952 |
| FAM20C | -1.045 | 203.796 | 0.297 | [-0.13, 0.04] | -0.005 | 0.888 |
| MSRB3 | -1.123 | 176.828 | 0.263 | [-0.29, 0.08] | -0.013 | 0.885 |
| OSCAR | -0.343 | 201.414 | 0.732 | [-0.18, 0.13] | -0.003 | 0.946 |
| ADGRF5 | -0.176 | 199.153 | 0.860 | [-0.11, 0.09] | -0.001 | 0.971 |
| CPAMD8 | 0.030 | 201.718 | 0.976 | [-0.31, 0.32] | 0.001 | 0.998 |
| LGI3 | 0.059 | 126.752 | 0.953 | [-0.3, 0.32] | 0.001 | 0.993 |
| LILRA2 | 3.060 | 148.713 | 0.003 | [0.24, 1.13] | 0.091 | 0.578 |
| KRT78 | 0.509 | 204.169 | 0.612 | [-0.34, 0.58] | 0.013 | 0.935 |
| ARHGAP18 | -2.034 | 196.330 | 0.043 | [-0.83, -0.01] | -0.054 | 0.808 |
| TMEM132C | 0.017 | 198.749 | 0.987 | [-0.15, 0.15] | 0.000 | 0.999 |
| LILRA3 | 1.145 | 182.497 | 0.254 | [-0.16, 0.6] | 0.025 | 0.885 |
| ABTB2 | 0.495 | 205.707 | 0.621 | [-0.18, 0.3] | 0.004 | 0.935 |
| GOLM1 | 0.740 | 203.112 | 0.460 | [-0.11, 0.25] | 0.008 | 0.918 |
| PCSK9 | -0.948 | 204.954 | 0.344 | [-0.2, 0.07] | -0.006 | 0.897 |
| TXNDC5 | -0.269 | 202.108 | 0.788 | [-0.23, 0.18] | -0.004 | 0.954 |
| PLA2G15 | -0.766 | 167.964 | 0.445 | [-0.22, 0.1] | -0.008 | 0.918 |
| GALNT6 | -0.912 | 199.491 | 0.363 | [-0.25, 0.09] | -0.010 | 0.913 |
| NAXE | 0.118 | 164.466 | 0.906 | [-0.11, 0.13] | 0.001 | 0.976 |
| HMCN2 | -1.698 | 205.456 | 0.091 | [-0.34, 0.03] | -0.019 | 0.830 |
| B3GNT7 | 0.096 | 187.197 | 0.924 | [-0.2, 0.22] | 0.001 | 0.979 |
| DNER | -0.129 | 168.418 | 0.898 | [-0.11, 0.09] | -0.001 | 0.976 |
| LILRB1 | 1.637 | 187.032 | 0.103 | [-0.03, 0.3] | 0.019 | 0.830 |
| ANGPTL6 | 1.259 | 171.074 | 0.210 | [-0.05, 0.24] | 0.010 | 0.885 |
| ACTRT1 | -1.123 | 184.074 | 0.263 | [-0.4, 0.11] | -0.014 | 0.885 |
| BPIFB1 | -1.332 | 203.492 | 0.184 | [-0.38, 0.07] | -0.020 | 0.863 |
| HAVCR2 | 0.492 | 204.359 | 0.623 | [-0.13, 0.22] | 0.005 | 0.935 |
| IGDCC4 | -1.827 | 186.704 | 0.069 | [-0.23, 0.01] | -0.012 | 0.818 |
| SNED1 | 1.118 | 205.066 | 0.265 | [-0.04, 0.14] | 0.005 | 0.886 |
| TSKU | -1.290 | 125.251 | 0.200 | [-0.49, 0.1] | -0.024 | 0.885 |
| CEMIP | -1.042 | 182.736 | 0.299 | [-0.18, 0.05] | -0.008 | 0.888 |
| PDCD6IP | -0.640 | 203.816 | 0.523 | [-0.28, 0.14] | -0.009 | 0.924 |
| LRRN4 | -2.395 | 162.921 | 0.018 | [-0.25, -0.02] | -0.019 | 0.740 |
| SECTM1 | -1.719 | 194.337 | 0.087 | [-0.5, 0.03] | -0.024 | 0.830 |
| ZFPM2 | -1.678 | 201.726 | 0.095 | [-0.59, 0.05] | -0.029 | 0.830 |
| ITLN1 | -1.110 | 205.420 | 0.268 | [-0.46, 0.13] | -0.018 | 0.888 |
| FCAMR | 0.080 | 202.322 | 0.936 | [-0.23, 0.25] | 0.001 | 0.984 |
| OIT3 | -0.587 | 205.070 | 0.558 | [-0.12, 0.07] | -0.003 | 0.925 |
| TTN | 0.505 | 201.028 | 0.614 | [-0.2, 0.33] | 0.007 | 0.935 |
| KLHL6 | 0.910 | 196.756 | 0.364 | [-0.13, 0.35] | 0.012 | 0.913 |
| ROBO4 | 0.132 | 204.985 | 0.895 | [-0.14, 0.16] | 0.001 | 0.976 |
| POMGNT1 | -1.091 | 201.226 | 0.277 | [-0.19, 0.05] | -0.008 | 0.888 |
| SMPDL3A | 1.277 | 195.908 | 0.203 | [-0.08, 0.36] | 0.017 | 0.885 |
| CFHR4 | -1.742 | 204.949 | 0.083 | [-0.31, 0.02] | -0.020 | 0.818 |
| FAM3C | -0.837 | 205.599 | 0.403 | [-0.2, 0.08] | -0.006 | 0.917 |
| PSMF1 | -0.179 | 205.658 | 0.858 | [-0.22, 0.18] | -0.002 | 0.971 |
| PXDN | 0.375 | 173.699 | 0.708 | [-0.16, 0.24] | 0.005 | 0.946 |
| NRGN | 0.426 | 203.193 | 0.670 | [-0.25, 0.39] | 0.007 | 0.941 |
| HTRA1 | -0.860 | 197.811 | 0.391 | [-0.22, 0.09] | -0.007 | 0.917 |
| GGH | -1.276 | 190.650 | 0.204 | [-0.19, 0.04] | -0.006 | 0.885 |
| NRCAM | 0.485 | 205.537 | 0.628 | [-0.08, 0.13] | 0.003 | 0.935 |
| SEMA4D | 0.471 | 192.265 | 0.638 | [-0.14, 0.22] | 0.005 | 0.935 |
| NEO1 | 2.113 | 205.999 | 0.036 | [0.01, 0.21] | 0.011 | 0.808 |
| OSTF1 | 0.725 | 191.136 | 0.470 | [-0.14, 0.3] | 0.011 | 0.918 |
| KHSRP | -0.035 | 202.640 | 0.972 | [-0.21, 0.2] | 0.000 | 0.997 |
| PRG4 | -0.514 | 198.041 | 0.608 | [-0.18, 0.11] | -0.003 | 0.935 |
| EXT2 | 2.348 | 203.537 | 0.020 | [0.02, 0.21] | 0.012 | 0.740 |
| ART4 | 0.109 | 197.981 | 0.914 | [-0.24, 0.26] | 0.002 | 0.979 |
| BHMT | -2.324 | 205.551 | 0.021 | [-0.54, -0.04] | -0.036 | 0.740 |
| HLA | -0.677 | 188.930 | 0.499 | [-0.4, 0.19] | -0.012 | 0.918 |
| HLA | 0.555 | 200.643 | 0.579 | [-0.14, 0.25] | 0.007 | 0.930 |
| LEAP2 | 1.850 | 189.749 | 0.066 | [-0.01, 0.37] | 0.014 | 0.818 |
| MYDGF | 1.199 | 200.953 | 0.232 | [-0.11, 0.44] | 0.021 | 0.885 |
| WBP2 | -1.135 | 194.523 | 0.258 | [-0.37, 0.1] | -0.017 | 0.885 |
| ESAM | -1.508 | 181.552 | 0.133 | [-0.43, 0.06] | -0.024 | 0.833 |
| COA7 | 0.626 | 197.146 | 0.532 | [-0.18, 0.35] | 0.008 | 0.924 |
| PLD4 | -0.705 | 175.446 | 0.482 | [-0.31, 0.15] | -0.009 | 0.918 |
| KCTD12 | -0.464 | 203.958 | 0.643 | [-0.26, 0.16] | -0.006 | 0.935 |
| ZG16B | -0.657 | 205.930 | 0.512 | [-0.33, 0.17] | -0.010 | 0.924 |
| PGM2 | 0.380 | 205.611 | 0.704 | [-0.2, 0.29] | 0.006 | 0.946 |
| SMARCD1 | -1.458 | 191.211 | 0.146 | [-0.28, 0.04] | -0.016 | 0.833 |
| TIMD4 | 0.899 | 205.480 | 0.370 | [-0.08, 0.21] | 0.008 | 0.913 |
| CRELD1 | -2.091 | 204.669 | 0.038 | [-0.29, -0.01] | -0.018 | 0.808 |
| ABHD14B | -0.497 | 205.941 | 0.620 | [-0.33, 0.2] | -0.008 | 0.935 |
| CPB2 | -1.003 | 197.828 | 0.317 | [-0.11, 0.04] | -0.003 | 0.892 |
| TXNDC15 | -1.094 | 196.545 | 0.276 | [-0.25, 0.07] | -0.010 | 0.888 |
| DNAH8 | -0.450 | 197.555 | 0.653 | [-0.5, 0.31] | -0.011 | 0.935 |
| L3MBTL3 | -0.818 | 192.823 | 0.414 | [-0.67, 0.28] | -0.019 | 0.918 |
| DCHS1 | -0.409 | 200.725 | 0.683 | [-0.24, 0.16] | -0.005 | 0.946 |
| MEGF10 | -1.182 | 204.730 | 0.239 | [-0.29, 0.07] | -0.013 | 0.885 |
| CNDP1 | -1.414 | 194.179 | 0.159 | [-0.42, 0.07] | -0.014 | 0.841 |
| CNDP2 | -0.846 | 204.240 | 0.398 | [-0.26, 0.1] | -0.010 | 0.917 |
| FAM20A | -0.278 | 198.661 | 0.781 | [-0.11, 0.08] | -0.002 | 0.954 |
| CFAP54 | -1.171 | 201.082 | 0.243 | [-0.69, 0.18] | -0.031 | 0.885 |
| DOCK7 | -0.245 | 187.752 | 0.807 | [-0.17, 0.13] | -0.002 | 0.962 |
| PRAP1 | -1.942 | 204.666 | 0.053 | [-0.35, 0] | -0.017 | 0.808 |
| SERPINB12 | -0.776 | 182.348 | 0.439 | [-0.39, 0.17] | -0.013 | 0.918 |
| DCBLD2 | -1.921 | 174.419 | 0.056 | [-0.35, 0] | -0.021 | 0.810 |
| PGLYRP2 | 1.266 | 204.029 | 0.207 | [-0.03, 0.13] | 0.003 | 0.885 |
| MTMR9 | -1.133 | 135.542 | 0.259 | [-0.51, 0.14] | -0.015 | 0.885 |
| PANX3 | 0.749 | 205.338 | 0.454 | [-0.32, 0.71] | 0.021 | 0.918 |
| FCRL5 | 0.261 | 200.042 | 0.794 | [-0.12, 0.16] | 0.002 | 0.956 |
| VPS13A | 0.219 | 197.315 | 0.827 | [-0.32, 0.39] | 0.004 | 0.970 |
| SPRR2F | -0.922 | 197.970 | 0.358 | [-0.47, 0.17] | -0.018 | 0.907 |
| HMCN1 | -1.483 | 192.229 | 0.140 | [-0.26, 0.04] | -0.015 | 0.833 |
| PEBP4 | -0.407 | 200.584 | 0.684 | [-0.24, 0.16] | -0.004 | 0.946 |
| NIBAN2 | -0.634 | 187.684 | 0.527 | [-0.12, 0.06] | -0.004 | 0.924 |
| ACOX2 | 1.087 | 199.464 | 0.278 | [-0.29, 1.01] | 0.033 | 0.888 |
| NELL2 | -2.436 | 204.535 | 0.016 | [-0.3, -0.03] | -0.022 | 0.740 |
| CNN2 | -0.453 | 203.996 | 0.651 | [-0.35, 0.22] | -0.007 | 0.935 |
| PARK7 | -0.452 | 193.561 | 0.651 | [-0.35, 0.22] | -0.006 | 0.935 |
| VAT1 | -1.911 | 193.107 | 0.058 | [-0.49, 0.01] | -0.028 | 0.811 |
| OSMR | 0.138 | 205.403 | 0.890 | [-0.11, 0.12] | 0.001 | 0.976 |
| COL12A1 | -0.001 | 190.576 | 1.000 | [-0.17, 0.17] | 0.000 | 1.000 |
| NAP1L4 | -0.698 | 200.930 | 0.486 | [-0.18, 0.09] | -0.006 | 0.918 |
| OLFM1 | 1.214 | 205.768 | 0.226 | [-0.04, 0.16] | 0.006 | 0.885 |
| ATF6B | 0.196 | 198.589 | 0.845 | [-0.13, 0.16] | 0.002 | 0.971 |
| RARRES2 | -1.637 | 189.623 | 0.103 | [-0.23, 0.02] | -0.009 | 0.830 |
| MYOC | -0.690 | 201.208 | 0.491 | [-0.2, 0.1] | -0.005 | 0.918 |
| OMD | -0.366 | 202.392 | 0.715 | [-0.26, 0.18] | -0.004 | 0.946 |
| DPYSL5 | 1.971 | 205.191 | 0.050 | [-0., 0.53] | 0.033 | 0.808 |
| NCAPG | 2.406 | 194.832 | 0.017 | [0.03, 0.28] | 0.019 | 0.740 |
| PDCD1LG2 | -2.297 | 181.270 | 0.023 | [-0.31, -0.02] | -0.019 | 0.740 |
| CORO1B | -0.102 | 185.956 | 0.919 | [-0.26, 0.24] | -0.002 | 0.979 |
| TXNDC17 | 1.043 | 202.092 | 0.298 | [-0.21, 0.69] | 0.030 | 0.888 |
| ERP44 | -0.610 | 194.051 | 0.542 | [-0.24, 0.13] | -0.007 | 0.924 |
| LXN | -0.631 | 201.989 | 0.529 | [-0.31, 0.16] | -0.009 | 0.924 |
| AARSD1 | 1.625 | 203.084 | 0.106 | [-0.03, 0.31] | 0.015 | 0.830 |
| FUCA2 | -1.217 | 170.407 | 0.225 | [-0.2, 0.05] | -0.008 | 0.885 |
| SPON2 | -1.625 | 201.566 | 0.106 | [-0.51, 0.05] | -0.024 | 0.830 |
| PDCD10 | -1.554 | 203.015 | 0.122 | [-0.28, 0.03] | -0.015 | 0.830 |
| MENT | -0.198 | 203.992 | 0.844 | [-0.29, 0.24] | -0.003 | 0.971 |
| APOO | -1.264 | 195.658 | 0.208 | [-0.2, 0.04] | -0.005 | 0.885 |
| ACAT2 | -0.181 | 195.059 | 0.857 | [-0.24, 0.2] | -0.003 | 0.971 |
| COLEC11 | 1.954 | 198.405 | 0.052 | [-0., 0.22] | 0.009 | 0.808 |
| BOC | -1.757 | 203.325 | 0.080 | [-0.24, 0.01] | -0.014 | 0.818 |
| C1QTNF5 | 0.473 | 200.989 | 0.637 | [-0.16, 0.26] | 0.006 | 0.935 |
| C1QTNF3 | 0.581 | 202.438 | 0.562 | [-0.11, 0.2] | 0.005 | 0.926 |
| CFHR5 | -0.678 | 196.402 | 0.498 | [-0.16, 0.08] | -0.003 | 0.918 |
| AP1M1 | -0.005 | 204.194 | 0.996 | [-0.45, 0.45] | 0.000 | 0.999 |
| CADM1 | 0.316 | 203.157 | 0.753 | [-0.09, 0.13] | 0.002 | 0.951 |
| CDHR2 | -0.334 | 187.089 | 0.739 | [-0.46, 0.32] | -0.008 | 0.946 |
| SEZ6L | 1.706 | 199.042 | 0.090 | [-0.03, 0.38] | 0.022 | 0.830 |
| FGFBP2 | -1.541 | 177.845 | 0.125 | [-0.38, 0.05] | -0.020 | 0.830 |
| UBE2O | -1.128 | 187.233 | 0.261 | [-0.21, 0.06] | -0.010 | 0.885 |
| PITHD1 | -0.877 | 186.010 | 0.381 | [-0.27, 0.1] | -0.010 | 0.917 |
| NIF3L1 | -0.817 | 193.250 | 0.415 | [-0.21, 0.09] | -0.007 | 0.918 |
| TWSG1 | -0.830 | 177.880 | 0.408 | [-0.16, 0.07] | -0.006 | 0.918 |
| SIL1 | 0.026 | 184.636 | 0.979 | [-0.17, 0.17] | 0.000 | 0.998 |
| MEGF9 | -1.443 | 190.925 | 0.151 | [-0.28, 0.04] | -0.016 | 0.833 |
| CDH23 | 2.278 | 196.373 | 0.024 | [0.03, 0.45] | 0.032 | 0.740 |
| SH3BGRL3 | 0.506 | 203.005 | 0.614 | [-0.45, 0.76] | 0.015 | 0.935 |
| SEMA4A | -0.618 | 198.495 | 0.537 | [-0.18, 0.09] | -0.006 | 0.924 |
| RNPEP | 0.275 | 167.212 | 0.783 | [-0.24, 0.32] | 0.005 | 0.954 |
| DPEP2 | 0.278 | 202.754 | 0.782 | [-0.09, 0.12] | 0.001 | 0.954 |
| TUBB1 | 1.737 | 204.382 | 0.084 | [-0.04, 0.65] | 0.034 | 0.821 |
| GLIPR2 | 0.643 | 179.042 | 0.521 | [-0.08, 0.16] | 0.004 | 0.924 |
| ALG2 | -1.805 | 205.999 | 0.073 | [-0.32, 0.01] | -0.021 | 0.818 |
| ANTXR1 | -2.053 | 198.709 | 0.041 | [-0.37, -0.01] | -0.022 | 0.808 |
| TUBA4B | -1.701 | 191.799 | 0.091 | [-0.34, 0.03] | -0.020 | 0.830 |
| MMRN2 | -0.090 | 205.678 | 0.929 | [-0.1, 0.09] | 0.000 | 0.980 |
| PSTPIP2 | -0.467 | 184.633 | 0.641 | [-0.23, 0.14] | -0.005 | 0.935 |
| COG4 | 2.651 | 205.791 | 0.009 | [0.09, 0.61] | 0.031 | 0.705 |
| CDHR5 | -2.132 | 184.410 | 0.034 | [-0.31, -0.01] | -0.017 | 0.808 |
| PARVB | -1.067 | 194.314 | 0.287 | [-0.79, 0.24] | -0.031 | 0.888 |
| SLC38A10 | -1.447 | 205.969 | 0.150 | [-0.21, 0.03] | -0.011 | 0.833 |
| ADGRL4 | -0.164 | 195.298 | 0.870 | [-0.2, 0.17] | -0.002 | 0.972 |
| GLOD4 | 0.602 | 204.837 | 0.548 | [-0.19, 0.35] | 0.011 | 0.925 |
| SPON1 | -1.241 | 203.008 | 0.216 | [-0.21, 0.05] | -0.009 | 0.885 |
| PCDH18 | -0.723 | 199.030 | 0.471 | [-0.16, 0.07] | -0.005 | 0.918 |
| GP6 | 0.196 | 201.733 | 0.845 | [-0.37, 0.45] | 0.004 | 0.971 |
| CD248 | -1.140 | 194.829 | 0.256 | [-0.24, 0.06] | -0.009 | 0.885 |
| MYO10 | 1.630 | 205.769 | 0.105 | [-0.07, 0.76] | 0.040 | 0.830 |
| RETN | 0.361 | 189.446 | 0.718 | [-0.13, 0.19] | 0.003 | 0.946 |
| APMAP | 0.876 | 199.815 | 0.382 | [-0.14, 0.38] | 0.012 | 0.917 |
| PCDH12 | 0.253 | 202.099 | 0.800 | [-0.15, 0.2] | 0.002 | 0.960 |
| IL1RAP | 1.109 | 187.516 | 0.269 | [-0.05, 0.18] | 0.006 | 0.888 |
| SEMA4B | -1.953 | 183.869 | 0.052 | [-0.2, 0] | -0.010 | 0.808 |
| CD93 | -0.903 | 205.712 | 0.368 | [-0.17, 0.06] | -0.005 | 0.913 |
| B3GAT2 | 0.957 | 205.789 | 0.340 | [-0.11, 0.3] | 0.009 | 0.897 |
| SPINK5 | -0.501 | 204.687 | 0.617 | [-0.18, 0.11] | -0.004 | 0.935 |
| CRTAC1 | -0.459 | 189.621 | 0.647 | [-0.2, 0.12] | -0.003 | 0.935 |
| RTN4 | 1.097 | 205.876 | 0.274 | [-0.08, 0.3] | 0.012 | 0.888 |
| NIT2 | 0.663 | 205.514 | 0.508 | [-0.12, 0.24] | 0.008 | 0.924 |
| NECTIN3 | -1.618 | 196.171 | 0.107 | [-0.32, 0.03] | -0.016 | 0.830 |
| PDLIM7 | 1.387 | 174.935 | 0.167 | [-0.07, 0.39] | 0.018 | 0.857 |
| MAN1C1 | -0.842 | 205.515 | 0.401 | [-0.22, 0.09] | -0.008 | 0.917 |
| ASAH2 | 1.147 | 175.115 | 0.253 | [-0.1, 0.37] | 0.016 | 0.885 |
| CHST12 | -1.588 | 186.002 | 0.114 | [-0.4, 0.04] | -0.024 | 0.830 |
| OLFML3 | -0.677 | 202.683 | 0.499 | [-0.21, 0.1] | -0.007 | 0.918 |
| GKN1 | -1.586 | 205.481 | 0.114 | [-0.36, 0.04] | -0.021 | 0.830 |
| SEMA3G | 1.020 | 202.778 | 0.309 | [-0.07, 0.23] | 0.010 | 0.892 |
| ST6GALNAC1 | 0.782 | 185.483 | 0.435 | [-0.15, 0.35] | 0.011 | 0.918 |
| OLA1 | 0.216 | 198.741 | 0.829 | [-0.28, 0.35] | 0.004 | 0.970 |
| CBLN4 | 1.687 | 205.959 | 0.093 | [-0.02, 0.25] | 0.012 | 0.830 |
| CYRIB | -0.157 | 201.177 | 0.875 | [-0.23, 0.2] | -0.002 | 0.973 |
| SEPTIN11 | -0.536 | 203.039 | 0.593 | [-0.21, 0.12] | -0.005 | 0.930 |
| CZIB | -0.355 | 174.442 | 0.723 | [-0.26, 0.18] | -0.005 | 0.946 |
| STAB1 | -0.039 | 203.044 | 0.969 | [-0.19, 0.18] | 0.000 | 0.997 |
| DPP3 | -0.560 | 199.764 | 0.576 | [-0.26, 0.14] | -0.007 | 0.928 |
| B3GNT2 | 0.508 | 205.463 | 0.612 | [-0.05, 0.08] | 0.002 | 0.935 |
| UGGT1 | -0.808 | 205.957 | 0.420 | [-0.32, 0.13] | -0.012 | 0.918 |
| RBAK | 0.137 | 197.966 | 0.891 | [-0.19, 0.22] | 0.002 | 0.976 |
| ERAP1 | 0.594 | 203.381 | 0.553 | [-0.13, 0.25] | 0.006 | 0.925 |
| AHSP | 0.937 | 198.072 | 0.350 | [-0.15, 0.43] | 0.016 | 0.900 |
| ADA2 | -0.696 | 205.953 | 0.487 | [-0.2, 0.09] | -0.005 | 0.918 |
| EHD3 | 0.524 | 204.954 | 0.601 | [-0.2, 0.34] | 0.008 | 0.932 |
| EHD2 | 0.866 | 204.723 | 0.388 | [-0.2, 0.51] | 0.016 | 0.917 |
| C1RL | 0.840 | 204.259 | 0.402 | [-0.04, 0.09] | 0.002 | 0.917 |
| CALML5 | 0.254 | 203.843 | 0.800 | [-0.33, 0.43] | 0.006 | 0.960 |
| ABRACL | -1.556 | 204.769 | 0.121 | [-0.74, 0.09] | -0.039 | 0.830 |
| CNTN3 | -0.792 | 198.553 | 0.429 | [-0.21, 0.09] | -0.006 | 0.918 |
| PTGFRN | 2.362 | 205.887 | 0.019 | [0.05, 0.56] | 0.038 | 0.740 |
| WRAP73 | -0.397 | 205.502 | 0.692 | [-0.15, 0.1] | -0.003 | 0.946 |
| DPM3 | -0.818 | 163.957 | 0.414 | [-0.21, 0.09] | -0.009 | 0.918 |
| MRC2 | 0.695 | 200.462 | 0.488 | [-0.1, 0.2] | 0.006 | 0.918 |
| DKK3 | 3.060 | 205.599 | 0.003 | [0.12, 0.57] | 0.041 | 0.578 |
| EXTL2 | 0.907 | 198.600 | 0.366 | [-0.06, 0.15] | 0.005 | 0.913 |
| CTSZ | -1.742 | 196.068 | 0.083 | [-0.22, 0.01] | -0.011 | 0.818 |
| BIN2 | 1.132 | 196.139 | 0.259 | [-0.11, 0.41] | 0.016 | 0.885 |
| CTSF | 0.649 | 194.413 | 0.517 | [-0.14, 0.27] | 0.008 | 0.924 |
| FBLN5 | -1.013 | 204.292 | 0.312 | [-0.26, 0.08] | -0.010 | 0.892 |
| MARCO | -1.444 | 188.550 | 0.150 | [-0.29, 0.04] | -0.012 | 0.833 |
| DNAH17 | -1.944 | 198.304 | 0.053 | [-0.69, 0] | -0.026 | 0.808 |
| GASK1A | 0.511 | 160.704 | 0.610 | [-0.12, 0.21] | 0.006 | 0.935 |
| FETUB | -0.692 | 205.074 | 0.489 | [-0.15, 0.07] | -0.003 | 0.918 |
| SUSD2 | -0.414 | 200.707 | 0.680 | [-0.25, 0.17] | -0.005 | 0.946 |
| PCYOX1 | 0.013 | 197.876 | 0.990 | [-0.14, 0.15] | 0.000 | 0.999 |
| CD84 | 0.169 | 195.348 | 0.866 | [-0.15, 0.18] | 0.002 | 0.971 |
| LNPEP | 0.790 | 196.717 | 0.430 | [-0.32, 0.76] | 0.023 | 0.918 |
| VAX2 | 0.756 | 154.329 | 0.451 | [-0.09, 0.21] | 0.007 | 0.918 |
| PLXNA1 | -0.576 | 205.994 | 0.565 | [-0.54, 0.3] | -0.016 | 0.926 |
| GGT7 | 1.149 | 186.232 | 0.252 | [-0.1, 0.38] | 0.019 | 0.885 |
| SH3BGRL2 | 0.185 | 204.736 | 0.853 | [-0.3, 0.36] | 0.004 | 0.971 |
| GNPTG | -1.025 | 205.890 | 0.306 | [-0.15, 0.05] | -0.005 | 0.892 |
| DBNL | 0.679 | 205.917 | 0.498 | [-0.17, 0.36] | 0.011 | 0.918 |
| GGA1 | 0.724 | 186.646 | 0.470 | [-0.12, 0.26] | 0.009 | 0.918 |
| NAGPA | -1.167 | 205.774 | 0.245 | [-0.27, 0.07] | -0.012 | 0.885 |
| SERPINA10 | -0.591 | 201.421 | 0.555 | [-0.11, 0.06] | -0.002 | 0.925 |
| PILRA | -2.350 | 178.430 | 0.020 | [-0.3, -0.03] | -0.020 | 0.740 |
| TRHDE | -0.473 | 205.200 | 0.637 | [-0.27, 0.17] | -0.006 | 0.935 |
| ANGPTL2 | -1.286 | 186.317 | 0.200 | [-0.37, 0.08] | -0.017 | 0.885 |
| MYH2 | -0.587 | 180.448 | 0.558 | [-0.36, 0.19] | -0.009 | 0.925 |
| PCOLCE2 | -0.885 | 191.968 | 0.377 | [-0.29, 0.11] | -0.010 | 0.917 |
| PSME2 | 0.931 | 194.250 | 0.353 | [-0.15, 0.43] | 0.018 | 0.900 |
| HEG1 | -1.968 | 199.795 | 0.051 | [-0.23, 0] | -0.012 | 0.808 |
| CORO1C | 0.942 | 188.224 | 0.347 | [-0.2, 0.57] | 0.020 | 0.897 |
| NOTCH3 | -0.764 | 204.610 | 0.446 | [-0.19, 0.08] | -0.006 | 0.918 |
| SYNPO2 | 1.086 | 182.865 | 0.279 | [-0.06, 0.21] | 0.006 | 0.888 |
| NENF | 0.463 | 204.259 | 0.644 | [-0.15, 0.25] | 0.006 | 0.935 |
| PCDHGC3 | -1.415 | 205.111 | 0.158 | [-0.27, 0.04] | -0.015 | 0.841 |
| PROCR | 0.093 | 191.237 | 0.926 | [-0.15, 0.17] | 0.001 | 0.979 |
| MINPP1 | -0.688 | 197.774 | 0.492 | [-0.08, 0.04] | -0.002 | 0.918 |
| CEP164 | 0.865 | 189.116 | 0.388 | [-0.15, 0.38] | 0.015 | 0.917 |
| PA2G4 | 0.216 | 189.626 | 0.829 | [-0.23, 0.29] | 0.004 | 0.970 |
| TNN | 1.114 | 197.332 | 0.267 | [-0.07, 0.24] | 0.012 | 0.888 |
| CLEC11A | 0.527 | 193.833 | 0.598 | [-0.09, 0.16] | 0.004 | 0.930 |
| HPSE | 0.450 | 180.469 | 0.653 | [-0.21, 0.33] | 0.007 | 0.935 |
| ST3GAL6 | -0.617 | 202.114 | 0.538 | [-0.17, 0.09] | -0.005 | 0.924 |
| TNFSF13B | -0.666 | 191.345 | 0.506 | [-0.19, 0.1] | -0.006 | 0.924 |
| VSIG4 | -0.811 | 203.113 | 0.418 | [-0.2, 0.08] | -0.007 | 0.918 |
| ITM2B | 0.169 | 172.369 | 0.866 | [-0.17, 0.2] | 0.002 | 0.971 |
| MAN2B2 | -0.371 | 182.804 | 0.711 | [-0.25, 0.17] | -0.005 | 0.946 |
| MYRF | 1.645 | 201.431 | 0.102 | [-0.02, 0.27] | 0.015 | 0.830 |
| KLHL20 | 1.374 | 155.106 | 0.172 | [-0.11, 0.6] | 0.036 | 0.857 |
| UFC1 | 0.417 | 204.270 | 0.677 | [-0.18, 0.28] | 0.007 | 0.946 |
| FBXO7 | -0.849 | 162.608 | 0.397 | [-0.28, 0.11] | -0.011 | 0.917 |
| TLN1 | -0.053 | 202.093 | 0.958 | [-0.68, 0.64] | -0.002 | 0.995 |
| NRXN3 | -0.284 | 184.699 | 0.777 | [-0.17, 0.13] | -0.003 | 0.954 |
| PLXND1 | -0.633 | 192.917 | 0.528 | [-0.14, 0.07] | -0.004 | 0.924 |
| TLN2 | -0.626 | 148.091 | 0.532 | [-0.32, 0.17] | -0.008 | 0.924 |
| HYOU1 | -1.285 | 205.986 | 0.200 | [-0.13, 0.03] | -0.004 | 0.885 |
| LRRC42 | 0.194 | 194.764 | 0.846 | [-0.2, 0.24] | 0.003 | 0.971 |
| PPME1 | -0.366 | 166.498 | 0.715 | [-0.19, 0.13] | -0.004 | 0.946 |
| ANGPTL3 | 1.210 | 187.437 | 0.228 | [-0.05, 0.21] | 0.008 | 0.885 |
| LYVE1 | -1.127 | 191.689 | 0.261 | [-0.2, 0.06] | -0.006 | 0.885 |
| PSAT1 | -1.051 | 201.951 | 0.294 | [-0.34, 0.1] | -0.016 | 0.888 |
| CPQ | -0.283 | 166.844 | 0.778 | [-0.14, 0.11] | -0.002 | 0.954 |
| KPTN | 0.791 | 202.878 | 0.430 | [-0.09, 0.21] | 0.007 | 0.918 |
| CLIC4 | -1.077 | 203.993 | 0.283 | [-0.47, 0.14] | -0.018 | 0.888 |
| ROBO1 | 1.159 | 205.528 | 0.248 | [-0.06, 0.21] | 0.009 | 0.885 |
| FCGBP | 0.621 | 205.649 | 0.535 | [-0.09, 0.18] | 0.004 | 0.924 |
| SCIN | -1.154 | 200.874 | 0.250 | [-0.26, 0.07] | -0.009 | 0.885 |
| LY96 | 0.490 | 201.369 | 0.624 | [-0.11, 0.18] | 0.005 | 0.935 |
| COLEC10 | 0.826 | 194.736 | 0.410 | [-0.05, 0.13] | 0.003 | 0.918 |
| dof, degrees of freedom; 95% CI, 95% confidence interval; Log2FC, log2 fold change; FDR, false discovery rate. T-values are reported with treatment responder group as the reference group. | | | | | | |

| Supplementary Table S4. Mass Spectrometry based metabolites between treatment responders and non-responders in the multi-omics cohort | | | | | | |
| --- | --- | --- | --- | --- | --- | --- |
| **Metabolites** | **T** | **dof** | ***P-*value** | **95% CI** | **Log2FC** | **FDR *P-value*** |
| Glycodeoxycholic acid 3-sulfate | -1.437 | 199.362 | 0.152 | [-1.05, 0.16] | -0.104 | 0.998 |
| Tridecanoylcarnitine (AcCa(13:0)) | -0.427 | 192.091 | 0.670 | [-0.51, 0.33] | -0.008 | 0.998 |
| Eicosatrienoylcarnitine (AcCa(20:3)) | 0.628 | 195.188 | 0.531 | [-0.28, 0.54] | 0.040 | 0.998 |
| Hydroxy-eicosadienoylcarnitine (AcCa(20:2-OH)) | 0.180 | 199.271 | 0.857 | [-0.25, 0.29] | 0.006 | 0.998 |
| Deoxycholic acid 3-sulfate | 0.091 | 195.349 | 0.928 | [-0.28, 0.3] | 0.002 | 0.998 |
| 24-Norursodeoxycholic acid | 0.817 | 187.580 | 0.415 | [-0.09, 0.22] | 0.006 | 0.998 |
| 1-Methylhistidine | -0.961 | 205.643 | 0.338 | [-0.46, 0.16] | -0.048 | 0.998 |
| 2-Ketobutyric acid | -1.137 | 193.519 | 0.257 | [-0.67, 0.18] | -0.033 | 0.998 |
| 2-Hydroxybutyric acid | -0.450 | 186.237 | 0.653 | [-1., 0.63] | -0.035 | 0.998 |
| 3-Hydroxybutyric acid | -0.469 | 185.460 | 0.640 | [-0.58, 0.36] | -0.044 | 0.998 |
| 4-Pyridoxic acid | -0.706 | 203.370 | 0.481 | [-0.16, 0.08] | -0.004 | 0.998 |
| Alpha-ketoisovaleric acid | -0.198 | 168.031 | 0.843 | [-0.6, 0.49] | -0.020 | 0.998 |
| Carnosine | -1.962 | 205.643 | 0.051 | [-0.43, 0] | -0.082 | 0.998 |
| Taurocholic acid | -0.721 | 205.984 | 0.471 | [-0.56, 0.26] | -0.053 | 0.998 |
| Butyric acid | -1.404 | 187.383 | 0.162 | [-0.25, 0.04] | -0.015 | 0.998 |
| Acetic acid | -0.488 | 205.195 | 0.626 | [-0.96, 0.58] | -0.037 | 0.998 |
| Betaine | -0.804 | 204.509 | 0.423 | [-0.39, 0.16] | -0.019 | 0.998 |
| Ascorbic acid | -1.231 | 196.093 | 0.220 | [-0.78, 0.18] | -0.203 | 0.998 |
| Bilirubin | 0.542 | 204.189 | 0.588 | [-0.11, 0.19] | 0.004 | 0.998 |
| Beta-Alanine | 0.256 | 200.308 | 0.798 | [-0.46, 0.6] | 0.011 | 0.998 |
| Acetoacetic acid | -0.450 | 185.134 | 0.653 | [-0.42, 0.26] | -0.015 | 0.998 |
| Carnitine | -0.338 | 184.889 | 0.735 | [-0.2, 0.14] | -0.004 | 0.998 |
| L-Carnitine | -0.172 | 197.460 | 0.864 | [-0.18, 0.15] | -0.002 | 0.998 |
| Creatine | -0.335 | 198.414 | 0.738 | [-0.13, 0.09] | -0.002 | 0.998 |
| Pipecolic acid | -1.206 | 198.608 | 0.229 | [-0.14, 0.03] | -0.005 | 0.998 |
| cis-Aconitic acid | -0.103 | 205.975 | 0.918 | [-0.62, 0.56] | -0.007 | 0.998 |
| Dihydrothymine | -1.428 | 204.684 | 0.155 | [-0.24, 0.04] | -0.011 | 0.998 |
| Cytidine | -1.119 | 199.253 | 0.264 | [-0.45, 0.12] | -0.033 | 0.998 |
| N,N-Dimethylglycine | 0.080 | 197.063 | 0.936 | [-0.5, 0.55] | 0.011 | 0.998 |
| Citric acid | -0.049 | 203.635 | 0.961 | [-0.17, 0.16] | 0.000 | 0.998 |
| Choline | 0.458 | 205.983 | 0.647 | [-0.4, 0.65] | 0.022 | 0.998 |
| Glycolate | -1.565 | 194.077 | 0.119 | [-0.49, 0.06] | -0.073 | 0.998 |
| Homovanillic acid | -1.665 | 202.655 | 0.097 | [-0.99, 0.08] | -0.088 | 0.998 |
| Glyoxylic acid | -1.761 | 184.057 | 0.080 | [-0.67, 0.04] | -0.051 | 0.998 |
| D-Glucose | -1.079 | 202.385 | 0.282 | [-0.57, 0.17] | -0.150 | 0.998 |
| Glycine | -0.583 | 198.322 | 0.561 | [-0.42, 0.23] | -0.015 | 0.998 |
| D-Glucuronic acid | 0.244 | 197.446 | 0.808 | [-0.06, 0.07] | 0.001 | 0.998 |
| Guanidoacetic acid | -0.814 | 190.032 | 0.417 | [-0.54, 0.23] | -0.033 | 0.998 |
| Fumaric acid | -0.628 | 200.181 | 0.531 | [-0.38, 0.2] | -0.017 | 0.998 |
| Glycocholic acid | 0.104 | 205.615 | 0.917 | [-0.15, 0.17] | 0.001 | 0.998 |
| Glyceric acid | -0.240 | 205.437 | 0.811 | [-0.24, 0.19] | -0.007 | 0.998 |
| D-Galactose | -0.339 | 186.010 | 0.735 | [-0.37, 0.26] | -0.005 | 0.998 |
| L-Glutamic acid | 0.312 | 205.972 | 0.755 | [-0.25, 0.34] | 0.005 | 0.998 |
| Ethanolamine | -1.864 | 205.970 | 0.064 | [-0.26, 0.01] | -0.012 | 0.998 |
| L-Malic acid | 0.836 | 193.772 | 0.404 | [-0.28, 0.69] | 0.043 | 0.998 |
| Hypoxanthine | -1.110 | 191.155 | 0.268 | [-0.68, 0.19] | -0.079 | 0.998 |
| L-Tyrosine | -0.399 | 202.066 | 0.690 | [-0.12, 0.08] | -0.002 | 0.998 |
| L-Phenylalanine | -2.023 | 206.000 | 0.044 | [-0.33, 0] | -0.048 | 0.998 |
| Alanine | 0.501 | 182.644 | 0.617 | [-0.24, 0.41] | 0.017 | 0.998 |
| L-Proline | 0.136 | 189.746 | 0.892 | [-0.2, 0.23] | 0.003 | 0.998 |
| L-Threonine | -0.622 | 199.427 | 0.535 | [-0.15, 0.08] | -0.004 | 0.998 |
| L-Asparagine | -0.418 | 204.386 | 0.676 | [-0.14, 0.09] | -0.002 | 0.998 |
| D-Mannose | -0.231 | 199.062 | 0.818 | [-0.61, 0.48] | -0.039 | 0.998 |
| L-Isoleucine | -0.845 | 194.875 | 0.399 | [-0.4, 0.16] | -0.026 | 0.998 |
| L-Histidine | 0.448 | 181.610 | 0.655 | [-0.12, 0.19] | 0.002 | 0.998 |
| L-Lysine | -1.001 | 193.468 | 0.318 | [-0.47, 0.15] | -0.044 | 0.998 |
| L-Serine | -0.204 | 199.839 | 0.838 | [-0.25, 0.21] | -0.004 | 0.998 |
| Lactic acid | -0.058 | 200.679 | 0.954 | [-0.46, 0.43] | -0.003 | 0.998 |
| L-Aspartic acid | -0.417 | 198.892 | 0.677 | [-0.47, 0.31] | -0.012 | 0.998 |
| L-Cystine | -1.200 | 201.965 | 0.232 | [-0.27, 0.07] | -0.028 | 0.998 |
| Indoleacetic Acid | -0.356 | 201.225 | 0.722 | [-0.26, 0.18] | -0.007 | 0.998 |
| Acetylcarnitine (AcCa(2:0)) | 0.294 | 204.305 | 0.769 | [-0.28, 0.38] | 0.053 | 0.998 |
| Methylmalonic acid | -0.526 | 202.888 | 0.599 | [-0.23, 0.13] | -0.005 | 0.998 |
| N6-Acetyl-L-lysine | -1.719 | 205.555 | 0.087 | [-0.54, 0.04] | -0.164 | 0.998 |
| Oleic acid | -0.879 | 205.865 | 0.381 | [-0.36, 0.14] | -0.025 | 0.998 |
| Phenylacetic acid | -1.416 | 203.300 | 0.158 | [-0.3, 0.05] | -0.019 | 0.998 |
| Pantothenic acid | -2.065 | 197.393 | 0.040 | [-0.33, -0.01] | -0.023 | 0.998 |
| Ornithine | -0.710 | 201.340 | 0.479 | [-0.37, 0.17] | -0.009 | 0.998 |
| Palmitic acid | -0.703 | 201.829 | 0.483 | [-0.2, 0.09] | -0.008 | 0.998 |
| Palmitoylcarnitine (AcCa(16:0)) | -0.176 | 205.977 | 0.861 | [-0.39, 0.33] | -0.011 | 0.998 |
| Orotic acid | 0.009 | 193.456 | 0.993 | [-0.28, 0.28] | 0.000 | 0.999 |
| Propionic acid | -2.858 | 204.249 | 0.005 | [-0.19, -0.04] | -0.016 | 0.914 |
| Pyruvic acid | -0.705 | 204.477 | 0.481 | [-0.16, 0.07] | -0.005 | 0.998 |
| Thyroxine | -0.794 | 170.144 | 0.429 | [-0.2, 0.09] | -0.013 | 0.998 |
| Taurine | 0.249 | 205.494 | 0.803 | [-0.28, 0.36] | 0.008 | 0.998 |
| Sphingosine | -0.777 | 191.244 | 0.438 | [-0.52, 0.22] | -0.032 | 0.998 |
| Succinic acid | 0.482 | 199.875 | 0.630 | [-0.65, 1.08] | 0.128 | 0.998 |
| Serotonin | -0.260 | 190.943 | 0.795 | [-0.35, 0.27] | -0.005 | 0.998 |
| Pyroglutamic acid | -0.222 | 202.033 | 0.824 | [-0.13, 0.11] | -0.001 | 0.998 |
| Sarcosine | 1.452 | 195.913 | 0.148 | [-0.07, 0.44] | 0.032 | 0.998 |
| Sphingosine 1-phosphate | -1.827 | 205.013 | 0.069 | [-0.35, 0.01] | -0.030 | 0.998 |
| Uric acid | 0.314 | 194.554 | 0.754 | [-0.17, 0.23] | 0.005 | 0.998 |
| Xanthine | -1.265 | 176.516 | 0.208 | [-0.46, 0.1] | -0.018 | 0.998 |
| Uridine | -1.741 | 189.960 | 0.083 | [-0.97, 0.06] | -0.310 | 0.998 |
| Uracil | 1.123 | 205.855 | 0.263 | [-0.06, 0.21] | 0.030 | 0.998 |
| 3beta-Hydroxy-5-cholenoic acid | 2.169 | 204.199 | 0.031 | [0.08, 1.59] | 0.511 | 0.998 |
| 3-Amino-2-piperidone | -1.720 | 205.155 | 0.087 | [-0.29, 0.02] | -0.020 | 0.998 |
| 12-Ketodeoxycholic acid | -0.802 | 196.985 | 0.424 | [-0.35, 0.15] | -0.033 | 0.998 |
| (R)-3-Hydroxyisobutyric acid | 0.003 | 178.977 | 0.998 | [-0.73, 0.73] | 0.001 | 0.999 |
| (S)-3,4-Dihydroxybutyric acid | -1.689 | 117.495 | 0.094 | [-0.61, 0.05] | -0.022 | 0.998 |
| 3-Hydroxysebacic acid | -1.680 | 198.573 | 0.095 | [-0.61, 0.05] | -0.060 | 0.998 |
| 3-Hydroxymethylglutaric acid | -0.165 | 203.938 | 0.869 | [-0.09, 0.08] | -0.001 | 0.998 |
| Isochenodeoxycholic acid | 0.008 | 185.633 | 0.994 | [-0.23, 0.24] | 0.000 | 0.999 |
| Omega-muricholic acid | -1.518 | 195.531 | 0.131 | [-1.02, 0.13] | -0.171 | 0.998 |
| 3-hydroxydodecanoic acid | -1.471 | 200.900 | 0.143 | [-0.16, 0.02] | -0.014 | 0.998 |
| 7-Ketocholic acid | -0.765 | 205.974 | 0.445 | [-0.15, 0.07] | -0.004 | 0.998 |
| 12-Oxocholic acid | -1.337 | 205.183 | 0.183 | [-0.76, 0.15] | -0.237 | 0.998 |
| 2-Hydroxy-3-methylbutyric acid | 1.432 | 199.163 | 0.154 | [-0.04, 0.24] | 0.021 | 0.998 |
| 3beta-Cholic acid | 1.108 | 205.976 | 0.269 | [-0.18, 0.65] | 0.129 | 0.998 |
| 3-Epideoxycholic acid | 0.689 | 200.115 | 0.491 | [-0.22, 0.45] | 0.015 | 0.998 |
| N-Alpha-acetyllysine | -0.043 | 187.270 | 0.965 | [-0.22, 0.22] | -0.001 | 0.998 |
| Adipic acid | -0.063 | 202.693 | 0.950 | [-0.1, 0.09] | 0.000 | 0.998 |
| alpha-Aminobutyric acid | -0.094 | 203.720 | 0.926 | [-0.8, 0.73] | 0.012 | 0.998 |
| 7-Ketochenodeoxycholic acid | 0.832 | 199.782 | 0.406 | [-0.09, 0.23] | 0.007 | 0.998 |
| 3-Methylhistidine | -0.162 | 202.998 | 0.871 | [-0.14, 0.11] | -0.002 | 0.998 |
| Caprylic acid(FFA(8:0) | 0.719 | 195.687 | 0.473 | [-0.3, 0.64] | 0.038 | 0.998 |
| Vanillic acid | 0.326 | 204.808 | 0.745 | [-0.52, 0.72] | 0.052 | 0.998 |
| 3-Methyl-2-oxovaleric acid | -0.624 | 192.028 | 0.533 | [-0.56, 0.29] | -0.032 | 0.998 |
| 4-Hydroxybenzoic acid | 0.148 | 176.108 | 0.882 | [-0.18, 0.21] | 0.002 | 0.998 |
| 3-Oxochenodeoxycholic acid | -0.948 | 205.191 | 0.344 | [-0.37, 0.13] | -0.016 | 0.998 |
| Aminoadipic acid | 0.597 | 192.588 | 0.551 | [-0.15, 0.27] | 0.011 | 0.998 |
| Capric acid | 1.501 | 178.554 | 0.135 | [-0.06, 0.48] | 0.153 | 0.998 |
| Capric acid | 0.213 | 203.674 | 0.831 | [-0.47, 0.59] | 0.008 | 0.998 |
| N-Acetyl-L-phenylalanine | -0.821 | 204.733 | 0.413 | [-0.16, 0.07] | -0.009 | 0.998 |
| L-Arginine | -0.320 | 186.334 | 0.750 | [-0.23, 0.17] | -0.004 | 0.998 |
| Chenodeoxycholic acid | -0.172 | 197.547 | 0.864 | [-0.18, 0.15] | -0.002 | 0.998 |
| cis-5-Tetradecenoic acid | -0.948 | 205.947 | 0.344 | [-0.33, 0.12] | -0.024 | 0.998 |
| Acetylglycine | -0.327 | 199.314 | 0.744 | [-0.28, 0.2] | -0.039 | 0.998 |
| Caproic acid | -0.321 | 190.485 | 0.749 | [-0.16, 0.12] | -0.005 | 0.998 |
| Creatinine | 1.257 | 205.965 | 0.210 | [-0.1, 0.45] | 0.033 | 0.998 |
| Cholic acid | 0.016 | 204.271 | 0.987 | [-0.1, 0.1] | 0.000 | 0.998 |
| Glutaconic acid | -0.296 | 185.465 | 0.767 | [-0.37, 0.27] | -0.006 | 0.998 |
| Ethylmalonic Acid | -1.862 | 172.429 | 0.064 | [-0.48, 0.01] | -0.036 | 0.998 |
| Dodecanedioic acid | 0.066 | 182.870 | 0.947 | [-0.21, 0.22] | 0.001 | 0.998 |
| D-Gluconic acid | -1.222 | 205.969 | 0.223 | [-0.35, 0.08] | -0.017 | 0.998 |
| Deoxycholic acid | 0.954 | 196.715 | 0.341 | [-0.08, 0.24] | 0.023 | 0.998 |
| Cytosine | -1.807 | 203.250 | 0.072 | [-0.44, 0.02] | -0.052 | 0.998 |
| Glycodeoxycholic acid | -0.231 | 205.771 | 0.818 | [-0.24, 0.19] | -0.004 | 0.998 |
| Glycochenodeoxycholic acid | -0.393 | 201.033 | 0.695 | [-0.18, 0.12] | -0.004 | 0.998 |
| Dodecanoic acid | 0.623 | 182.158 | 0.534 | [-0.09, 0.17] | 0.006 | 0.998 |
| L-Glutamine | -1.680 | 202.464 | 0.095 | [-0.39, 0.03] | -0.032 | 0.998 |
| Decanoylcarnitine (AcCa(10:0)) | -0.786 | 204.913 | 0.433 | [-0.24, 0.1] | -0.015 | 0.998 |
| Glutaric acid | -0.462 | 201.503 | 0.644 | [-0.28, 0.18] | -0.009 | 0.998 |
| Homo-L-Arginine | -0.051 | 192.886 | 0.959 | [-0.36, 0.34] | -0.002 | 0.998 |
| Hexadecanedioic acid | -0.274 | 197.658 | 0.784 | [-0.13, 0.1] | -0.002 | 0.998 |
| Linoleic acid | -1.421 | 203.454 | 0.157 | [-0.21, 0.03] | -0.015 | 0.998 |
| indoxyl sulfate | -1.420 | 204.716 | 0.157 | [-0.35, 0.06] | -0.033 | 0.998 |
| Kynurenine | -1.313 | 195.922 | 0.191 | [-0.61, 0.12] | -0.091 | 0.998 |
| Isoursodeoxycholic acid | 0.091 | 201.480 | 0.928 | [-0.25, 0.27] | 0.003 | 0.998 |
| L-Leucine | 0.270 | 202.631 | 0.788 | [-0.11, 0.14] | 0.006 | 0.998 |
| Isovalerylcarnitine (AcCa(iso5:0)) | -0.295 | 199.552 | 0.768 | [-0.46, 0.34] | -0.024 | 0.998 |
| Isocaproic acid | 0.366 | 200.550 | 0.715 | [-0.17, 0.25] | 0.014 | 0.998 |
| L-2-Hydroxyglutaric acid | -0.120 | 200.412 | 0.904 | [-0.18, 0.16] | -0.003 | 0.998 |
| Ketoleucine | 1.170 | 205.176 | 0.243 | [-0.18, 0.69] | 0.038 | 0.998 |
| L-Methionine | -0.243 | 182.081 | 0.808 | [-0.16, 0.12] | -0.003 | 0.998 |
| Glycolithocholic acid | -0.197 | 197.970 | 0.844 | [-0.13, 0.11] | -0.001 | 0.998 |
| 1-Methylnicotinamide | 0.965 | 196.047 | 0.336 | [-0.1, 0.3] | 0.013 | 0.998 |
| Hydroxypropionic acid | -1.038 | 198.067 | 0.301 | [-0.87, 0.27] | -0.089 | 0.998 |
| Hexanoylcarnitine (AcCa(6:0)) | 0.694 | 197.224 | 0.489 | [-0.22, 0.47] | 0.024 | 0.998 |
| Glycoursodeoxycholic acid | -1.769 | 137.219 | 0.079 | [-0.42, 0.02] | -0.041 | 0.998 |
| Hippuric acid | -0.678 | 193.989 | 0.499 | [-0.31, 0.15] | -0.011 | 0.998 |
| kynurenic acid | -0.442 | 190.563 | 0.659 | [-0.39, 0.25] | -0.013 | 0.998 |
| Isovaleric acid | -1.501 | 189.181 | 0.135 | [-0.61, 0.08] | -0.082 | 0.998 |
| Taurolithocholic acid | -0.469 | 190.346 | 0.640 | [-0.27, 0.17] | -0.007 | 0.998 |
| 4-Hydroxyproline | -0.608 | 192.751 | 0.544 | [-0.58, 0.3] | -0.029 | 0.998 |
| Indole-3-acrylic acid | -1.493 | 185.632 | 0.137 | [-0.48, 0.07] | -0.075 | 0.998 |
| Isobutyrylcarnitine (AcCa(iso4:0)) | 0.945 | 186.520 | 0.346 | [-0.1, 0.27] | 0.008 | 0.998 |
| Hydroxyisocaproic acid | -1.080 | 205.511 | 0.282 | [-0.9, 0.26] | -0.047 | 0.998 |
| Hydroxyisovaleric acid | 0.281 | 193.338 | 0.779 | [-0.21, 0.28] | 0.003 | 0.998 |
| 4-hydroxyphenyllactate | 1.150 | 202.606 | 0.252 | [-0.04, 0.16] | 0.010 | 0.998 |
| Hyocholic acid | 0.188 | 191.471 | 0.851 | [-0.11, 0.14] | 0.002 | 0.998 |
| Lithocholic acid | -0.627 | 204.004 | 0.531 | [-0.81, 0.42] | -0.119 | 0.998 |
| N-Acetyl-L-alanine | -2.033 | 174.487 | 0.044 | [-0.62, -0.01] | -0.066 | 0.998 |
| Pseudouridine | -1.241 | 195.144 | 0.216 | [-0.84, 0.19] | -0.119 | 0.998 |
| Nonadecanoic acid | -1.506 | 178.701 | 0.134 | [-0.34, 0.05] | -0.020 | 0.998 |
| Octadecanedioic acid | -0.906 | 201.465 | 0.366 | [-0.88, 0.33] | -0.059 | 0.998 |
| Octanoylcarnitine (AcCa(8:0)) | -0.468 | 198.493 | 0.640 | [-0.87, 0.53] | -0.075 | 0.998 |
| Sebacic acid | -1.655 | 202.515 | 0.099 | [-0.32, 0.03] | -0.021 | 0.998 |
| Myristic acid | 0.176 | 183.709 | 0.860 | [-0.12, 0.14] | 0.002 | 0.998 |
| N-Acetyaspartic acid | -1.578 | 201.134 | 0.116 | [-0.22, 0.02] | -0.023 | 0.998 |
| Propionylcarnitine (AcCa(3:0)) | -1.083 | 203.135 | 0.280 | [-0.34, 0.1] | -0.020 | 0.998 |
| Pentadecanoic acid | 0.470 | 200.466 | 0.639 | [-0.18, 0.29] | 0.007 | 0.998 |
| Stearic acid | -2.527 | 193.647 | 0.012 | [-0.36, -0.04] | -0.030 | 0.998 |
| 2-Hydroxy Hippuric Acid | 2.059 | 187.384 | 0.041 | [0.01, 0.58] | 0.040 | 0.998 |
| Stearoylcarnitine (AcCa(18:0)) | -0.099 | 189.309 | 0.921 | [-0.4, 0.36] | -0.002 | 0.998 |
| Pimelic acid | -2.266 | 196.123 | 0.025 | [-0.81, -0.06] | -0.052 | 0.998 |
| Ribonic acid | -0.805 | 205.999 | 0.422 | [-0.08, 0.03] | -0.004 | 0.998 |
| Histamine | -1.341 | 197.344 | 0.181 | [-0.14, 0.03] | -0.009 | 0.998 |
| Tauroursodeoxycholic acid | -0.328 | 168.983 | 0.744 | [-0.19, 0.14] | -0.003 | 0.998 |
| Trigonelline | -0.696 | 196.576 | 0.487 | [-0.23, 0.11] | -0.008 | 0.998 |
| L-Valine | -0.202 | 205.577 | 0.840 | [-0.15, 0.12] | -0.003 | 0.998 |
| Valeric acid | -0.867 | 187.572 | 0.387 | [-0.45, 0.17] | -0.017 | 0.998 |
| Taurodeoxycholic acid | -2.433 | 168.313 | 0.016 | [-0.32, -0.03] | -0.110 | 0.998 |
| 7-Methylguanine | 0.141 | 198.414 | 0.888 | [-0.22, 0.26] | 0.005 | 0.998 |
| Citrulline | -1.469 | 199.019 | 0.144 | [-0.65, 0.1] | -0.068 | 0.998 |
| Lithocholic acid 3-sulfate | -1.382 | 201.689 | 0.168 | [-0.39, 0.07] | -0.023 | 0.998 |
| Tridecanoic acid | -0.106 | 204.700 | 0.915 | [-0.19, 0.17] | -0.003 | 0.998 |
| Ursocholic acid | -1.862 | 205.204 | 0.064 | [-0.19, 0.01] | -0.027 | 0.998 |
| Trimethylamine N-oxide | -0.680 | 193.675 | 0.497 | [-0.38, 0.19] | -0.013 | 0.998 |
| L-Tryptophan | 0.783 | 198.664 | 0.434 | [-0.04, 0.1] | 0.002 | 0.998 |
| Tauro beta-muricholic acid | -0.185 | 199.507 | 0.854 | [-0.25, 0.2] | -0.003 | 0.998 |
| Threonic acid | -1.078 | 205.228 | 0.282 | [-0.25, 0.07] | -0.043 | 0.998 |
| Behenic acid | -0.877 | 197.859 | 0.382 | [-0.17, 0.06] | -0.013 | 0.998 |
| Ursodeoxycholic acid | -0.289 | 205.994 | 0.773 | [-0.15, 0.11] | -0.005 | 0.998 |
| Undecanoic acid | -0.603 | 194.761 | 0.547 | [-0.32, 0.17] | -0.019 | 0.998 |
| Taurochenodeoxycholic acid | -0.723 | 200.225 | 0.470 | [-0.26, 0.12] | -0.013 | 0.998 |
| Tartaric acid | -0.735 | 204.105 | 0.463 | [-0.44, 0.2] | -0.017 | 0.998 |
| Hypotaurine | 0.924 | 204.516 | 0.356 | [-0.11, 0.31] | 0.026 | 0.998 |
| Biliverdin | -0.849 | 202.963 | 0.397 | [-0.25, 0.1] | -0.012 | 0.998 |
| N-Formyl-L-methionine | -0.653 | 196.105 | 0.514 | [-0.28, 0.14] | -0.031 | 0.998 |
| Arachidonic acid | -0.690 | 205.980 | 0.491 | [-0.27, 0.13] | -0.016 | 0.998 |
| Glyceraldehyde | -0.676 | 185.286 | 0.500 | [-0.29, 0.14] | -0.014 | 0.998 |
| N-Acetyglutamic acid | -0.452 | 187.304 | 0.651 | [-0.43, 0.27] | -0.015 | 0.998 |
| 5'-Methylthioadenosine | -0.270 | 198.274 | 0.788 | [-0.94, 0.71] | -0.056 | 0.998 |
| spermine | 0.493 | 205.387 | 0.623 | [-0.42, 0.7] | 0.015 | 0.998 |
| Hydroxypyruvic acid | -0.286 | 202.495 | 0.775 | [-0.71, 0.53] | -0.027 | 0.998 |
| Sphinganine 1-phosphate | 0.162 | 193.465 | 0.871 | [-0.27, 0.32] | 0.008 | 0.998 |
| alpha-Linolenic acid | -0.483 | 188.894 | 0.630 | [-0.26, 0.16] | -0.013 | 0.998 |
| Niacinamide | -0.842 | 199.219 | 0.401 | [-1.06, 0.42] | -0.112 | 0.998 |
| 3-Hydroxyanthranilic acid | 0.836 | 196.292 | 0.404 | [-0.06, 0.14] | 0.008 | 0.998 |
| 1-Methylguanosine | -0.825 | 204.818 | 0.410 | [-0.26, 0.1] | -0.019 | 0.998 |
| 2-Hydroxycaproic acid | -0.580 | 200.265 | 0.563 | [-0.28, 0.15] | -0.010 | 0.998 |
| D-Xylulose | -0.327 | 195.145 | 0.744 | [-0.22, 0.16] | -0.011 | 0.998 |
| Benzoic acid | -0.181 | 201.198 | 0.856 | [-0.21, 0.17] | -0.007 | 0.998 |
| Isobutyric acid | -0.648 | 200.480 | 0.518 | [-0.18, 0.09] | -0.026 | 0.998 |
| Salicylic acid | -0.267 | 205.300 | 0.790 | [-0.29, 0.22] | -0.013 | 0.998 |
| 2-Hydroxy-2-methylbutyric acid | -1.435 | 195.757 | 0.153 | [-0.38, 0.06] | -0.030 | 0.998 |
| cis-5,8,11,14,17-Eicosapentaenoic acid | -1.333 | 204.854 | 0.184 | [-0.39, 0.08] | -0.059 | 0.998 |
| Myristoleic acid | -1.350 | 197.061 | 0.179 | [-0.7, 0.13] | -0.101 | 0.998 |
| Methionine sulfoxide | -0.128 | 201.353 | 0.898 | [-0.33, 0.29] | -0.027 | 0.998 |
| Butyrylcarnitine (AcCa(4:0)) | -1.781 | 185.859 | 0.077 | [-1.25, 0.06] | 0.926 | 0.998 |
| Tetradecenoylcarnitine 1 (AcCa(14:1) 1) | -1.511 | 196.212 | 0.132 | [-0.64, 0.08] | -0.120 | 0.998 |
| Erucic acid | -0.153 | 205.991 | 0.879 | [-0.26, 0.22] | -0.005 | 0.998 |
| Oleoylethanolamide | -0.522 | 151.689 | 0.602 | [-0.24, 0.14] | -0.004 | 0.998 |
| Malonylcarnitine (AcCa(3:0-DC)) | -1.863 | 188.857 | 0.064 | [-1.73, 0.05] | 0.483 | 0.998 |
| 3-Indolebutyric acid | -1.056 | 198.413 | 0.292 | [-0.46, 0.14] | 0.356 | 0.998 |
| Palmitoylethanolamide | -1.361 | 194.339 | 0.175 | [-0.53, 0.1] | -0.050 | 0.998 |
| Oleamide | -0.978 | 196.726 | 0.329 | [-0.45, 0.15] | -0.064 | 0.998 |
| Ethylmethylacetic acid | -0.681 | 200.795 | 0.497 | [-0.32, 0.16] | 0.123 | 0.998 |
| cis-4,7,10,13,16,19-Docosahexanoic acid | -0.398 | 202.769 | 0.691 | [-0.32, 0.21] | -0.279 | 0.998 |
| 4-hydroxyphenylpropionoic acid | -0.421 | 193.459 | 0.674 | [-0.39, 0.25] | -0.012 | 0.998 |
| 3R-hydroxydecanoic acid | 0.896 | 176.611 | 0.372 | [-0.1, 0.26] | 0.020 | 0.998 |
| Arachidic acid | -0.376 | 205.883 | 0.708 | [-0.19, 0.13] | -0.011 | 0.998 |
| cis-7,10,13,16-Docosatetraenoic acid | -2.039 | 203.246 | 0.043 | [-0.35, -0.01] | -0.053 | 0.998 |
| 11-Eicosenoic acid | -0.727 | 198.737 | 0.468 | [-0.14, 0.07] | -0.004 | 0.998 |
| Dodecanoylcarnitine (AcCa(12:0)) | 0.296 | 203.018 | 0.768 | [-0.3, 0.41] | 0.009 | 0.998 |
| Heptadecanoic acid | -0.287 | 198.801 | 0.775 | [-0.49, 0.37] | -0.024 | 0.998 |
| Imidazolepropionic acid | -2.402 | 192.144 | 0.017 | [-0.85, -0.08] | -0.075 | 0.998 |
| N-Acetylcadaverine | -0.938 | 183.553 | 0.349 | [-0.21, 0.08] | -0.019 | 0.998 |
| Indole-3-propionic acid | -0.539 | 204.160 | 0.591 | [-0.77, 0.44] | -0.070 | 0.998 |
| Oxalic acid | -0.412 | 204.573 | 0.681 | [-0.16, 0.1] | -0.005 | 0.998 |
| Heneicosanoic acid | 0.667 | 203.929 | 0.506 | [-0.26, 0.53] | 0.016 | 0.998 |
| Nervonic acid | -1.770 | 202.060 | 0.078 | [-0.36, 0.02] | -0.039 | 0.998 |
| Taurochenodeoxycholic acid 3-sulfate | -1.611 | 194.081 | 0.109 | [-0.57, 0.06] | -0.253 | 0.998 |
| Glycochenodeoxycholic acid 3-sulfate | -0.234 | 192.760 | 0.816 | [-0.52, 0.41] | -0.017 | 0.998 |
| Taurolithocholic acid 3-sulfate | -0.619 | 196.648 | 0.536 | [-0.17, 0.09] | -0.004 | 0.998 |
| A,b-Dihydroxyisobutyric acid | -0.381 | 195.451 | 0.704 | [-0.11, 0.08] | -0.002 | 0.998 |
| Glycolithocholic acid 3-sulfate | -1.095 | 196.705 | 0.275 | [-0.33, 0.09] | -0.013 | 0.998 |
| 3-(3-Hydroxyphenyl)-3-hydroxypropanoic acid | -1.031 | 198.094 | 0.304 | [-0.34, 0.11] | -0.020 | 0.998 |
| Erythrose | -0.121 | 197.110 | 0.904 | [-0.7, 0.62] | 0.141 | 0.998 |
| 1-Methylinosine | -2.109 | 191.992 | 0.036 | [-0.23, -0.01] | -0.034 | 0.998 |
| Docosatrienoic acid | -1.309 | 205.358 | 0.192 | [-0.18, 0.04] | -0.011 | 0.998 |
| cis-8,11,14-Eicosatrienoic acid | -0.119 | 205.990 | 0.905 | [-0.42, 0.37] | -0.011 | 0.998 |
| O-Acetylserine | 0.853 | 205.214 | 0.394 | [-0.31, 0.78] | 0.039 | 0.998 |
| Ergothioneine | -1.488 | 202.456 | 0.138 | [-0.5, 0.07] | -0.073 | 0.998 |
| Palmitoleic acid | 1.059 | 205.932 | 0.291 | [-0.12, 0.4] | 0.100 | 0.998 |
| Vaccenic acid | 0.967 | 201.665 | 0.335 | [-0.09, 0.26] | 0.019 | 0.998 |
| 1-Methyladenosine | -0.566 | 205.962 | 0.572 | [-0.28, 0.16] | -0.016 | 0.998 |
| Symmetric dimethylarginine | 0.277 | 196.837 | 0.782 | [-0.16, 0.21] | -0.035 | 0.998 |
| 4-Guanidinobutanoic acid | -0.361 | 205.192 | 0.718 | [-0.51, 0.35] | -0.032 | 0.998 |
| 4-Acetamidobutanoic acid | -1.362 | 196.937 | 0.175 | [-1.15, 0.21] | 1.084 | 0.998 |
| Bovinic acid | -0.547 | 203.380 | 0.585 | [-0.08, 0.04] | -0.005 | 0.998 |
| N6-Methyladenosine | -1.402 | 186.831 | 0.162 | [-0.56, 0.09] | -0.076 | 0.998 |
| 5-Methoxyindoleacetate | -0.975 | 189.337 | 0.331 | [-0.44, 0.15] | -0.049 | 0.998 |
| dopamine-4-sulfate | -0.972 | 188.337 | 0.333 | [-0.51, 0.17] | -0.077 | 0.998 |
| N1-Methyl-2-pyridone-5-carboxamide | -1.092 | 205.997 | 0.276 | [-0.48, 0.14] | -0.076 | 0.998 |
| N-a-Acetyl-L-arginine | -0.890 | 190.123 | 0.375 | [-0.52, 0.2] | -0.030 | 0.998 |
| N2,N2-Dimethylguanosine | -1.533 | 125.694 | 0.128 | [-0.32, 0.04] | -0.012 | 0.998 |
| Eicosadienoic acid | -1.085 | 195.392 | 0.279 | [-0.62, 0.18] | -0.080 | 0.998 |
| Oleoylcarnitine (AcCa(18:1)) | -1.098 | 192.730 | 0.274 | [-0.77, 0.22] | -0.159 | 0.998 |
| Tetradecanoylcarnitine (AcCa(14:0)) | -0.708 | 197.773 | 0.480 | [-0.75, 0.35] | -0.115 | 0.998 |
| N-Acetyglutamine | -0.322 | 189.056 | 0.748 | [-0.19, 0.14] | -0.035 | 0.998 |
| N2-Methylguanine | -0.226 | 193.672 | 0.821 | [-0.22, 0.17] | -0.024 | 0.998 |
| 3-Hydroxyhippuric acid | -0.775 | 199.484 | 0.439 | [-0.74, 0.32] | -0.126 | 0.998 |
| Linolenylcarnitine (AcCa(18:3)) | 0.362 | 190.276 | 0.718 | [-0.22, 0.32] | 0.015 | 0.998 |
| Phenylacetylglutamine | 1.412 | 200.537 | 0.159 | [-0.06, 0.36] | 0.032 | 0.998 |
| Arachidonoylcarnitine (AcCa(20:4)) | -0.043 | 196.063 | 0.966 | [-0.28, 0.27] | -0.003 | 0.998 |
| Linoleylcarnitine (AcCa(18:2)) | 0.660 | 203.668 | 0.510 | [-0.13, 0.26] | 0.011 | 0.998 |
| Docosapentaenoic acid(22:5n-3) | 0.104 | 192.646 | 0.917 | [-0.11, 0.12] | 0.000 | 0.998 |
| 5,8,11-Eicosatrienoic acid | 1.463 | 197.924 | 0.145 | [-0.04, 0.28] | 0.013 | 0.998 |
| 2-Decenoic acid | 0.884 | 192.666 | 0.378 | [-0.1, 0.25] | 0.011 | 0.998 |
| Cinnamoylglycine | 0.720 | 205.827 | 0.472 | [-0.3, 0.64] | 0.150 | 0.998 |
| p-Cresol sulfate | 1.273 | 200.703 | 0.205 | [-0.07, 0.34] | 0.023 | 0.998 |
| p-Cresol glucuronide | 1.353 | 198.753 | 0.178 | [-0.05, 0.25] | 0.012 | 0.998 |
| Homovanillic acid sulfate | 1.009 | 197.812 | 0.314 | [-0.08, 0.25] | 0.016 | 0.998 |
| N-Acetymethionine | 0.930 | 196.722 | 0.353 | [-0.08, 0.22] | 0.008 | 0.998 |
| N-Acetylvaline | 1.747 | 198.882 | 0.082 | [-0.01, 0.2] | 0.008 | 0.998 |
| Iodide | 1.348 | 198.892 | 0.179 | [-0.04, 0.23] | 0.009 | 0.998 |
| Valerylcarnitine (AcCa(5:0)) | 1.461 | 204.483 | 0.145 | [-0.04, 0.27] | 0.023 | 0.998 |
| Glutarylcarnitine (AcCa(5:0-DC)) | 0.647 | 202.516 | 0.519 | [-0.26, 0.52] | 0.016 | 0.998 |
| Methylmalonylcarnitine/Succinylcarnitine (AcCa(4:0-DC)) | 1.271 | 198.985 | 0.205 | [-0.05, 0.24] | 0.014 | 0.998 |
| Hexenoylcarnitine (AcCa(6:1)) | 1.060 | 194.985 | 0.291 | [-0.07, 0.22] | 0.009 | 0.998 |
| Hexadecenoylcarnitine (AcCa(16:1)) | 1.034 | 192.214 | 0.302 | [-0.07, 0.22] | 0.012 | 0.998 |
| Octenoylcarnitine (AcCa(8:1)) | 0.313 | 202.984 | 0.755 | [-0.25, 0.34] | 0.006 | 0.998 |
| Decadienoylcarnitine 1 (AcCa(10:2) 1) | 1.176 | 196.242 | 0.241 | [-0.06, 0.24] | 0.009 | 0.998 |
| Dodecenoylcarnitine 1 (AcCa(12:1) 1) | 0.929 | 202.412 | 0.354 | [-0.08, 0.22] | 0.007 | 0.998 |
| Tetradecenoylcarnitine 2 (AcCa(14:1) 2) | 1.179 | 195.179 | 0.240 | [-0.06, 0.25] | 0.013 | 0.998 |
| Hydroxy-tetradecenoylcarnitine (AcCa(14:1-OH)) | 1.157 | 200.127 | 0.249 | [-0.06, 0.24] | 0.017 | 0.998 |
| Tetradecadiencarnitine (AcCa(14:2)) | 1.324 | 196.982 | 0.187 | [-0.05, 0.24] | 0.012 | 0.998 |
| Hydroxy-tetradecadiencarnitine (AcCa(14:2-OH)) | 1.419 | 190.586 | 0.157 | [-0.04, 0.26] | 0.012 | 0.998 |
| Hydroxy-hexadecenoylcarnitine (AcCa(16:1-OH)) | -2.343 | 205.421 | 0.020 | [-0.41, -0.04] | -0.030 | 0.998 |
| Hexadecadienoylcarnitine (AcCa(16:2)) | -0.027 | 205.674 | 0.978 | [-0.31, 0.31] | -0.001 | 0.998 |
| Hydroxy-hexadecadienoylcarnitine (AcCa(16:2-OH)) | 0.426 | 198.378 | 0.671 | [-0.1, 0.16] | 0.003 | 0.998 |
| Hydroxyhexadecanoylcarnitine (AcCa(16:0-OH)) | 1.367 | 196.653 | 0.173 | [-0.04, 0.2] | 0.009 | 0.998 |
| Hydroxy-octadecenoylcarnitine (AcCa(18:1-OH)) | 0.741 | 205.711 | 0.460 | [-0.08, 0.18] | 0.007 | 0.998 |
| cis-10-Nonadecenoic acid | 0.331 | 204.964 | 0.741 | [-0.18, 0.25] | 0.007 | 0.998 |
| Leucyl-leucine | 1.274 | 199.047 | 0.204 | [-0.05, 0.23] | 0.012 | 0.998 |
| Indole-3-carboxaldehyde | -0.054 | 203.720 | 0.957 | [-0.21, 0.2] | -0.001 | 0.998 |
| Indole-3-methyl acetate | 0.549 | 189.952 | 0.584 | [-0.09, 0.16] | 0.003 | 0.998 |
| 9-Decenoic acid | 0.180 | 204.042 | 0.857 | [-0.23, 0.28] | 0.004 | 0.998 |
| 2-Methylhexanoic acid | 0.580 | 195.696 | 0.563 | [-0.1, 0.18] | 0.004 | 0.998 |
| 3-Aminobutanoic acid | -0.406 | 196.061 | 0.685 | [-0.15, 0.1] | -0.003 | 0.998 |
| N-Acetylhistidine | 0.911 | 205.621 | 0.364 | [-0.07, 0.19] | 0.007 | 0.998 |
| 3-Methylvaleric acid | 0.713 | 202.507 | 0.476 | [-0.12, 0.26] | 0.011 | 0.998 |
| 3-Hydroxyglutaric acid | 0.930 | 195.598 | 0.353 | [-0.07, 0.2] | 0.007 | 0.998 |
| phenyl sulfate | 0.904 | 198.544 | 0.367 | [-0.07, 0.19] | 0.006 | 0.998 |
| Tryptophan betaine | 0.241 | 197.342 | 0.810 | [-0.12, 0.15] | 0.002 | 0.998 |
| Hydroxytetradecanoyl carnitine (AcCa(14:0-OH)) | 0.088 | 189.837 | 0.930 | [-0.16, 0.17] | 0.001 | 0.998 |
| 3-Hydroxymyristic acid | 1.736 | 205.572 | 0.084 | [-0.03, 0.5] | 0.035 | 0.998 |
| 3-Hydroxypalmitic acid | -0.166 | 205.988 | 0.868 | [-0.35, 0.3] | -0.006 | 0.998 |
| Docosadienoate(22:2n6) | -0.488 | 203.378 | 0.626 | [-0.28, 0.17] | -0.008 | 0.998 |
| N-Acetyl-beta-alanine | -1.176 | 158.673 | 0.241 | [-0.35, 0.09] | -0.023 | 0.998 |
| Pentadecanoylcarnitine (AcCa(15:0)) | 0.775 | 182.653 | 0.439 | [-0.09, 0.21] | 0.008 | 0.998 |
| 2-Hydroxystearic acid | -0.590 | 175.187 | 0.556 | [-0.21, 0.12] | -0.010 | 0.998 |
| N-Acetylthreonine | 0.082 | 202.329 | 0.935 | [-0.33, 0.36] | 0.004 | 0.998 |
| 3-Oxodeoxycholic acid | 0.763 | 204.635 | 0.447 | [-0.13, 0.3] | 0.013 | 0.998 |
| 4-Methylcatechol sulfate | 1.320 | 201.208 | 0.188 | [-0.03, 0.17] | 0.008 | 0.998 |
| Glycohyocholic acid | 0.084 | 205.995 | 0.933 | [-0.23, 0.25] | 0.001 | 0.998 |
| Eicoseneoylcarnitine (AcCa(20:1)) | 0.042 | 198.972 | 0.966 | [-0.22, 0.23] | 0.001 | 0.998 |
| Eicosadienoylcarnitine (AcCa(20:2)) | 0.214 | 201.643 | 0.831 | [-0.42, 0.52] | 0.017 | 0.998 |
| Hydroxy-linoleylcarnitine (AcCa(18:2-OH)) | 0.808 | 205.368 | 0.420 | [-0.18, 0.44] | 0.032 | 0.998 |
| Norcholic acid | 1.040 | 194.926 | 0.300 | [-0.08, 0.24] | 0.012 | 0.998 |
| Tauro alpha-muricholic acid | 1.156 | 201.336 | 0.249 | [-0.05, 0.2] | 0.011 | 0.998 |
| Adipoylcarnitine (AcCa(6:0-DC)) | 0.753 | 199.139 | 0.452 | [-0.12, 0.26] | 0.027 | 0.998 |
| Decadienoylcarnitine 2 (AcCa(10:2) 2) | -2.097 | 185.725 | 0.037 | [-0.83, -0.03] | -0.082 | 0.998 |
| Decadienoylcarnitine 3 (AcCa(10:2) 3) | 1.008 | 204.910 | 0.315 | [-0.07, 0.21] | 0.007 | 0.998 |
| Decadienoylcarnitine 4 (AcCa(10:2) 4) | 0.715 | 203.904 | 0.476 | [-0.06, 0.13] | 0.003 | 0.998 |
| Decenoylcarnitine 2 (AcCa(10:1) 2) | -0.103 | 205.368 | 0.918 | [-0.19, 0.17] | -0.001 | 0.998 |
| Decenoylcarnitine 3 (AcCa(10:1) 3) | 0.360 | 204.219 | 0.720 | [-0.2, 0.29] | 0.008 | 0.998 |
| Decenoylcarnitine 4 (AcCa(10:1) 4) | -0.377 | 196.537 | 0.706 | [-0.46, 0.31] | -0.032 | 0.998 |
| Dodecenoylcarnitine 2 (AcCa(12:1) 2) | 0.283 | 205.974 | 0.778 | [-0.15, 0.2] | 0.004 | 0.998 |
| Dodecenoylcarnitine 3 (AcCa(12:1) 3) | 0.101 | 198.393 | 0.919 | [-0.13, 0.14] | 0.001 | 0.998 |
| Hexenoylcarnitine 1 (AcCa(6:1) 1) | 0.684 | 196.914 | 0.495 | [-0.13, 0.27] | 0.012 | 0.998 |
| Hexenoylcarnitine 2 (AcCa(6:1) 2) | 0.630 | 199.027 | 0.529 | [-0.13, 0.26] | 0.011 | 0.998 |
| Taurodeoxycholic acid 3-sulfate | -0.220 | 204.477 | 0.826 | [-0.39, 0.31] | -0.016 | 0.998 |
| SM(30:2) | 1.746 | 199.287 | 0.082 | [-0.02, 0.27] | 0.013 | 0.998 |
| SM(31:1) | 0.001 | 201.690 | 0.999 | [-0.32, 0.32] | 0.000 | 0.999 |
| SM(31:2) | 0.444 | 202.132 | 0.657 | [-0.15, 0.23] | 0.005 | 0.998 |
| SM(32:0) | -0.092 | 204.360 | 0.927 | [-0.14, 0.13] | -0.001 | 0.998 |
| SM(32:1) | 0.363 | 203.462 | 0.717 | [-0.1, 0.14] | 0.002 | 0.998 |
| SM(32:2) | 0.472 | 205.953 | 0.637 | [-0.1, 0.16] | 0.003 | 0.998 |
| SM(32:3) | 0.370 | 201.618 | 0.712 | [-0.09, 0.13] | 0.002 | 0.998 |
| SM(33:0) | -0.297 | 205.987 | 0.767 | [-0.27, 0.2] | -0.004 | 0.998 |
| SM(33:1) | 1.222 | 205.523 | 0.223 | [-0.09, 0.37] | 0.031 | 0.998 |
| SM(33:2) | 0.029 | 205.186 | 0.977 | [-0.16, 0.17] | 0.000 | 0.998 |
| SM(34:0) | -0.094 | 196.399 | 0.926 | [-0.19, 0.17] | -0.001 | 0.998 |
| SM(34:1) | 0.542 | 191.290 | 0.588 | [-0.09, 0.17] | 0.003 | 0.998 |
| SM(34:2) | -0.180 | 203.421 | 0.857 | [-0.31, 0.25] | -0.006 | 0.998 |
| SM(34:3) | -0.265 | 204.414 | 0.791 | [-0.24, 0.19] | -0.007 | 0.998 |
| SM(35:0) | -0.434 | 199.741 | 0.665 | [-0.23, 0.15] | -0.006 | 0.998 |
| SM(35:1) | -0.180 | 204.607 | 0.857 | [-0.27, 0.23] | -0.003 | 0.998 |
| SM(35:2) | -0.301 | 203.779 | 0.764 | [-0.15, 0.11] | -0.002 | 0.998 |
| SM(36:0) | 0.448 | 205.996 | 0.655 | [-0.09, 0.14] | 0.003 | 0.998 |
| SM(36:1) | 1.010 | 205.999 | 0.314 | [-0.07, 0.21] | 0.008 | 0.998 |
| SM(36:2) | 0.680 | 205.955 | 0.498 | [-0.1, 0.21] | 0.009 | 0.998 |
| SM(36:3) | -0.489 | 194.169 | 0.625 | [-0.22, 0.13] | -0.010 | 0.998 |
| SM(36:4) | -1.419 | 205.924 | 0.157 | [-0.33, 0.05] | -0.022 | 0.998 |
| SM(37:1) | -0.847 | 188.441 | 0.398 | [-0.23, 0.09] | -0.006 | 0.998 |
| SM(37:2) | -0.118 | 205.878 | 0.906 | [-0.16, 0.14] | -0.001 | 0.998 |
| SM(38:0) | 1.164 | 205.492 | 0.246 | [-0.07, 0.28] | 0.012 | 0.998 |
| SM(38:1) | 1.472 | 204.456 | 0.143 | [-0.04, 0.28] | 0.017 | 0.998 |
| SM(38:2) | 1.433 | 205.960 | 0.154 | [-0.03, 0.22] | 0.016 | 0.998 |
| SM(38:3) | -1.367 | 200.243 | 0.173 | [-0.45, 0.08] | -0.060 | 0.998 |
| SM(39:0) | 0.769 | 205.112 | 0.443 | [-0.12, 0.27] | 0.019 | 0.998 |
| SM(39:2) | 1.372 | 205.829 | 0.172 | [-0.06, 0.31] | 0.035 | 0.998 |
| SM(40:0) | 1.023 | 197.612 | 0.307 | [-0.09, 0.29] | 0.018 | 0.998 |
| SM(40:2) | 0.869 | 205.629 | 0.386 | [-0.06, 0.16] | 0.008 | 0.998 |
| SM(40:5) | 1.661 | 202.070 | 0.098 | [-0.02, 0.22] | 0.017 | 0.998 |
| SM(41:2) | 0.304 | 183.320 | 0.762 | [-0.09, 0.13] | 0.002 | 0.998 |
| SM(41:3) | 1.019 | 191.999 | 0.310 | [-0.08, 0.25] | 0.013 | 0.998 |
| SM(42:0) | 0.500 | 203.022 | 0.618 | [-0.11, 0.18] | 0.005 | 0.998 |
| SM(42:1) | -0.178 | 199.983 | 0.859 | [-0.17, 0.14] | -0.002 | 0.998 |
| SM(42:2) | 0.952 | 201.410 | 0.342 | [-0.08, 0.24] | 0.012 | 0.998 |
| SM(42:3) | 0.709 | 201.556 | 0.479 | [-0.08, 0.16] | 0.005 | 0.998 |
| SM(42:4) | 1.758 | 195.352 | 0.080 | [-0.01, 0.24] | 0.013 | 0.998 |
| SM(42:6) | 1.636 | 198.826 | 0.103 | [-0.04, 0.42] | 0.041 | 0.998 |
| SM(42:7) | 0.969 | 202.923 | 0.334 | [-0.11, 0.31] | 0.019 | 0.998 |
| SM(43:2) | 0.706 | 204.424 | 0.481 | [-0.1, 0.2] | 0.008 | 0.998 |
| SM(44:2) | 1.129 | 202.925 | 0.260 | [-0.05, 0.19] | 0.008 | 0.998 |
| SM(44:3) | -0.454 | 199.019 | 0.651 | [-0.15, 0.09] | -0.003 | 0.998 |
| PC(28:0) | 2.620 | 202.951 | 0.009 | [0.04, 0.29] | 0.023 | 0.998 |
| PC(30:0) | 3.168 | 205.879 | 0.002 | [0.09, 0.4] | 0.041 | 0.673 |
| PC(32:0) | 1.187 | 205.918 | 0.237 | [-0.08, 0.32] | 0.023 | 0.998 |
| PC(32:1) | 1.394 | 203.946 | 0.165 | [-0.05, 0.27] | 0.022 | 0.998 |
| PC(32:2) | 1.675 | 205.853 | 0.095 | [-0.06, 0.68] | 0.089 | 0.998 |
| PC(32:3) | -1.441 | 164.738 | 0.152 | [-0.51, 0.08] | -0.050 | 0.998 |
| PC(33:0) | 0.392 | 205.761 | 0.695 | [-0.29, 0.43] | 0.014 | 0.998 |
| PC(33:2) | 0.644 | 204.030 | 0.521 | [-0.24, 0.46] | 0.029 | 0.998 |
| PC(34:0) | -0.692 | 179.816 | 0.490 | [-0.34, 0.17] | -0.020 | 0.998 |
| PC(34:1) | -0.592 | 202.391 | 0.554 | [-0.3, 0.16] | -0.009 | 0.998 |
| PC(34:2) | 2.118 | 197.387 | 0.035 | [0.03, 0.84] | 0.054 | 0.998 |
| PC(34:3) | 0.208 | 202.160 | 0.835 | [-0.2, 0.24] | 0.003 | 0.998 |
| PC(34:4) | -0.317 | 201.989 | 0.752 | [-0.35, 0.25] | -0.008 | 0.998 |
| PC(34:5) | -0.517 | 199.668 | 0.606 | [-0.33, 0.19] | -0.014 | 0.998 |
| PC(35:1) | -0.144 | 191.189 | 0.885 | [-0.25, 0.21] | -0.003 | 0.998 |
| PC(35:2) | -0.315 | 200.394 | 0.753 | [-0.27, 0.2] | -0.005 | 0.998 |
| PC(35:3) | -0.105 | 200.402 | 0.917 | [-0.22, 0.2] | -0.001 | 0.998 |
| PC(35:4) | -0.075 | 201.169 | 0.940 | [-0.22, 0.21] | -0.001 | 0.998 |
| PC(35:5) | -0.110 | 201.634 | 0.913 | [-0.19, 0.17] | -0.001 | 0.998 |
| PC(36:0) | 0.484 | 203.606 | 0.629 | [-0.3, 0.5] | 0.017 | 0.998 |
| PC(36:1-1) | 1.742 | 199.398 | 0.083 | [-0.02, 0.38] | 0.024 | 0.998 |
| PC(36:1-2) | -0.189 | 200.722 | 0.850 | [-0.1, 0.08] | -0.001 | 0.998 |
| PC(36:2) | 2.137 | 197.213 | 0.034 | [0.01, 0.34] | 0.023 | 0.998 |
| PC(36:3) | -1.241 | 195.439 | 0.216 | [-0.4, 0.09] | -0.029 | 0.998 |
| PC(36:4) | -0.584 | 201.583 | 0.560 | [-0.38, 0.21] | -0.012 | 0.998 |
| PC(36:5) | -1.340 | 201.253 | 0.182 | [-0.29, 0.06] | -0.012 | 0.998 |
| PC(36:6) | -0.519 | 198.162 | 0.604 | [-0.24, 0.14] | -0.006 | 0.998 |
| PC(37:2) | 1.325 | 205.745 | 0.186 | [-0.07, 0.36] | 0.016 | 0.998 |
| PC(37:3) | 0.141 | 199.622 | 0.888 | [-0.27, 0.31] | 0.004 | 0.998 |
| PC(37:5) | 2.488 | 201.005 | 0.014 | [0.05, 0.42] | 0.035 | 0.998 |
| PC(38:1) | -1.727 | 201.958 | 0.086 | [-0.52, 0.03] | -0.038 | 0.998 |
| PC(38:2) | -0.503 | 205.526 | 0.616 | [-0.3, 0.18] | -0.009 | 0.998 |
| PC(38:3) | -0.551 | 200.085 | 0.582 | [-0.09, 0.05] | -0.002 | 0.998 |
| PC(38:4) | 0.852 | 205.405 | 0.395 | [-0.13, 0.33] | 0.012 | 0.998 |
| PC(38:5) | 0.883 | 201.473 | 0.378 | [-0.11, 0.29] | 0.013 | 0.998 |
| PC(38:6) | -1.272 | 195.409 | 0.205 | [-0.27, 0.06] | -0.021 | 0.998 |
| PC(38:7) | 0.723 | 190.933 | 0.470 | [-0.11, 0.24] | 0.011 | 0.998 |
| PC(39:4) | 0.050 | 190.757 | 0.960 | [-0.21, 0.22] | 0.001 | 0.998 |
| PC(40:4) | 0.840 | 205.332 | 0.402 | [-0.14, 0.36] | 0.021 | 0.998 |
| PC(40:5) | 1.085 | 192.366 | 0.279 | [-0.08, 0.28] | 0.012 | 0.998 |
| PC(40:6) | 0.628 | 194.260 | 0.531 | [-0.23, 0.45] | 0.024 | 0.998 |
| PC(40:7) | -0.705 | 185.302 | 0.482 | [-0.25, 0.12] | -0.011 | 0.998 |
| PC(40:8) | 0.678 | 193.024 | 0.498 | [-0.11, 0.22] | 0.009 | 0.998 |
| PC(42:5) | -0.362 | 204.187 | 0.718 | [-0.13, 0.09] | -0.002 | 0.998 |
| PC(42:8) | 2.606 | 198.762 | 0.010 | [0.05, 0.34] | 0.026 | 0.998 |
| PC(42:9) | 1.036 | 192.457 | 0.301 | [-0.09, 0.28] | 0.017 | 0.998 |
| PC-O(30:1) | -0.379 | 160.628 | 0.705 | [-0.26, 0.18] | -0.006 | 0.998 |
| PC-O(32:0) | 0.734 | 205.465 | 0.464 | [-0.34, 0.75] | 0.076 | 0.998 |
| PC-O(32:1) | -0.117 | 194.349 | 0.907 | [-0.23, 0.2] | -0.002 | 0.998 |
| PC-O(34:2) | 1.061 | 197.160 | 0.290 | [-0.14, 0.47] | 0.035 | 0.998 |
| PC-O(34:3) | 0.027 | 189.644 | 0.978 | [-0.24, 0.25] | 0.001 | 0.998 |
| PC-O(36:2) | 1.514 | 192.715 | 0.132 | [-0.04, 0.31] | 0.023 | 0.998 |
| PC-O(36:3) | 2.337 | 197.016 | 0.020 | [0.03, 0.37] | 0.028 | 0.998 |
| PC-O(36:4) | 0.546 | 178.512 | 0.586 | [-0.19, 0.33] | 0.016 | 0.998 |
| PC-O(36:5) | -0.584 | 205.318 | 0.560 | [-0.76, 0.41] | -0.116 | 0.998 |
| PC-O(36:6) | -1.854 | 205.997 | 0.065 | [-0.27, 0.01] | -0.011 | 0.998 |
| PC-O(38:2) | 1.096 | 197.706 | 0.274 | [-0.09, 0.3] | 0.020 | 0.998 |
| PC-O(38:4) | 0.650 | 191.914 | 0.516 | [-0.14, 0.27] | 0.011 | 0.998 |
| PC-O(38:5) | 1.453 | 191.915 | 0.148 | [-0.05, 0.3] | 0.017 | 0.998 |
| PC-O(38:6) | 0.698 | 204.028 | 0.486 | [-0.27, 0.56] | 0.035 | 0.998 |
| PC-O(38:7) | -1.123 | 177.397 | 0.263 | [-0.39, 0.11] | -0.034 | 0.998 |
| PC-O(40:6) | 1.004 | 201.402 | 0.317 | [-0.11, 0.34] | 0.025 | 0.998 |
| PE(30:1) | 3.050 | 200.253 | 0.003 | [0.09, 0.42] | 0.038 | 0.673 |
| PE(30:2) | -0.747 | 184.590 | 0.456 | [-0.37, 0.17] | -0.025 | 0.998 |
| PE(32:0) | 0.064 | 200.835 | 0.949 | [-0.21, 0.22] | 0.001 | 0.998 |
| PE(32:1) | 0.066 | 199.742 | 0.947 | [-0.29, 0.31] | 0.003 | 0.998 |
| PE(32:2) | 0.078 | 205.999 | 0.938 | [-0.09, 0.1] | 0.000 | 0.998 |
| PE(34:0) | -0.978 | 190.214 | 0.329 | [-0.3, 0.1] | -0.020 | 0.998 |
| PE(34:1) | 1.158 | 196.987 | 0.248 | [-0.09, 0.34] | 0.028 | 0.998 |
| PE(34:2) | 1.327 | 195.356 | 0.186 | [-0.07, 0.35] | 0.029 | 0.998 |
| PE(34:3) | 1.052 | 200.855 | 0.294 | [-0.16, 0.53] | 0.050 | 0.998 |
| PE(35:1) | 1.017 | 194.016 | 0.311 | [-0.1, 0.31] | 0.018 | 0.998 |
| PE(35:2) | 1.638 | 199.234 | 0.103 | [-0.04, 0.42] | 0.040 | 0.998 |
| PE(36:1) | 0.611 | 190.262 | 0.542 | [-0.15, 0.29] | 0.014 | 0.998 |
| PE(36:2) | -0.117 | 178.320 | 0.907 | [-0.21, 0.18] | -0.002 | 0.998 |
| PE(36:3) | 1.897 | 197.412 | 0.059 | [-0.01, 0.42] | 0.027 | 0.998 |
| PE(36:4) | 0.609 | 200.828 | 0.543 | [-0.23, 0.44] | 0.018 | 0.998 |
| PE(36:5) | 0.604 | 197.334 | 0.547 | [-0.06, 0.11] | 0.002 | 0.998 |
| PE(37:4) | 1.212 | 199.450 | 0.227 | [-0.21, 0.86] | 0.067 | 0.998 |
| PE(37:6) | 1.405 | 199.133 | 0.162 | [-0.06, 0.36] | 0.020 | 0.998 |
| PE(38:2) | 1.654 | 179.653 | 0.100 | [-0.03, 0.29] | 0.018 | 0.998 |
| PE(38:3) | 2.148 | 195.055 | 0.033 | [0.01, 0.33] | 0.022 | 0.998 |
| PE(38:4) | -0.159 | 203.266 | 0.874 | [-0.3, 0.25] | -0.005 | 0.998 |
| PE(38:5) | 0.209 | 203.557 | 0.834 | [-0.16, 0.2] | 0.004 | 0.998 |
| PE(38:6) | 2.232 | 197.535 | 0.027 | [0.02, 0.37] | 0.029 | 0.998 |
| PE(38:7) | -0.421 | 205.652 | 0.674 | [-0.15, 0.1] | -0.005 | 0.998 |
| PE(39:6) | 0.755 | 196.486 | 0.451 | [-0.13, 0.29] | 0.024 | 0.998 |
| PE(40:4) | 0.033 | 195.622 | 0.973 | [-0.3, 0.31] | 0.002 | 0.998 |
| PE(40:5) | -0.192 | 166.534 | 0.848 | [-0.16, 0.13] | -0.001 | 0.998 |
| PE(40:6) | 0.818 | 201.574 | 0.415 | [-0.13, 0.31] | 0.019 | 0.998 |
| PE(40:7) | -0.629 | 204.395 | 0.530 | [-0.34, 0.18] | -0.028 | 0.998 |
| PE(40:8) | -0.369 | 197.673 | 0.712 | [-0.26, 0.18] | -0.011 | 0.998 |
| PE(p16:0/18:1) | -0.006 | 204.142 | 0.995 | [-0.16, 0.16] | 0.000 | 0.999 |
| PE(p16:0/18:2) | -2.239 | 204.838 | 0.026 | [-0.57, -0.04] | -0.109 | 0.998 |
| PE(p16:0/20:3) | 0.750 | 205.489 | 0.454 | [-0.1, 0.21] | 0.009 | 0.998 |
| PE(p16:0/20:4) | -0.512 | 196.634 | 0.609 | [-0.3, 0.18] | -0.019 | 0.998 |
| PE(p16:0/20:5) | 0.922 | 203.567 | 0.357 | [-0.09, 0.24] | 0.012 | 0.998 |
| PE(p16:0/22:4) | 0.658 | 195.890 | 0.511 | [-0.13, 0.25] | 0.013 | 0.998 |
| PE(p16:0/22:5) | 0.284 | 199.562 | 0.777 | [-0.21, 0.27] | 0.004 | 0.998 |
| PE(p16:0/22:6) | -0.591 | 202.848 | 0.555 | [-0.12, 0.07] | -0.003 | 0.998 |
| PE(p18:0/18:1) | 0.670 | 202.834 | 0.504 | [-0.1, 0.21] | 0.008 | 0.998 |
| PE(p18:0/18:2) | 0.412 | 197.137 | 0.681 | [-0.13, 0.2] | 0.005 | 0.998 |
| PE(p18:0/18:3) | 0.429 | 196.164 | 0.669 | [-0.14, 0.21] | 0.007 | 0.998 |
| PE(p18:0/20:3) | -0.646 | 184.687 | 0.519 | [-0.23, 0.12] | -0.012 | 0.998 |
| PE(p18:0/20:5) | -0.437 | 189.513 | 0.663 | [-0.24, 0.15] | -0.014 | 0.998 |
| PE(p18:0/22:4) | 0.318 | 199.671 | 0.751 | [-0.22, 0.31] | 0.015 | 0.998 |
| PE(p18:0/22:5) | 1.057 | 190.291 | 0.292 | [-0.07, 0.22] | 0.014 | 0.998 |
| PE(p18:0/22:6) | 1.501 | 199.933 | 0.135 | [-0.05, 0.34] | 0.046 | 0.998 |
| PE(p18:1/16:0) | 0.670 | 199.996 | 0.504 | [-0.1, 0.19] | 0.010 | 0.998 |
| PE(p18:1/18:1) | 0.314 | 197.625 | 0.754 | [-0.13, 0.18] | 0.005 | 0.998 |
| PE(p18:1/18:2) | 0.777 | 198.716 | 0.438 | [-0.04, 0.1] | 0.002 | 0.998 |
| PE(p18:1/20:4) | -0.760 | 198.384 | 0.448 | [-0.24, 0.1] | -0.014 | 0.998 |
| PE(p18:1/20:5) | 0.631 | 205.911 | 0.529 | [-0.09, 0.18] | 0.005 | 0.998 |
| PE(p18:1/22:4) | -0.752 | 205.380 | 0.453 | [-0.32, 0.15] | -0.011 | 0.998 |
| PE(p18:1/22:5) | 0.445 | 201.396 | 0.657 | [-0.25, 0.39] | 0.013 | 0.998 |
| PE(p18:1/22:6) | 0.058 | 204.111 | 0.954 | [-0.15, 0.16] | 0.000 | 0.998 |
| PE(p18:2/20:2) | -0.153 | 203.764 | 0.879 | [-0.16, 0.14] | -0.001 | 0.998 |
| PE(p18:2/20:4) | -0.059 | 205.889 | 0.953 | [-0.24, 0.23] | -0.001 | 0.998 |
| PE(p20:0/18:1) | -0.269 | 204.968 | 0.788 | [-0.54, 0.41] | -0.009 | 0.998 |
| PE(p20:0/18:2) | 0.528 | 203.656 | 0.598 | [-0.16, 0.28] | 0.009 | 0.998 |
| PE(p20:0/22:6) | -0.927 | 205.075 | 0.355 | [-0.31, 0.11] | -0.009 | 0.998 |
| PE(p20:1/20:4) | 1.247 | 204.164 | 0.214 | [-0.04, 0.17] | 0.005 | 0.998 |
| PE(p20:1/22:6) | -0.495 | 202.627 | 0.621 | [-0.21, 0.13] | -0.003 | 0.998 |
| PE-O(34:1) | -0.042 | 205.841 | 0.966 | [-0.25, 0.24] | 0.000 | 0.998 |
| PE-O(36:1) | -0.696 | 198.576 | 0.488 | [-0.27, 0.13] | -0.010 | 0.998 |
| PE-O(36:2) | 0.482 | 198.439 | 0.630 | [-0.15, 0.24] | 0.006 | 0.998 |
| PE-O(36:3) | 1.363 | 203.968 | 0.174 | [-0.06, 0.34] | 0.013 | 0.998 |
| PE-O(36:4) | 0.487 | 192.111 | 0.626 | [-0.3, 0.51] | 0.022 | 0.998 |
| PE-O(36:5) | -0.422 | 205.601 | 0.673 | [-0.37, 0.24] | -0.016 | 0.998 |
| PE-O(38:3) | -0.196 | 199.431 | 0.845 | [-0.41, 0.34] | -0.008 | 0.998 |
| PE-O(38:4) | 0.352 | 199.154 | 0.725 | [-0.29, 0.42] | 0.015 | 0.998 |
| PE-O(38:5) | -1.175 | 185.366 | 0.241 | [-0.52, 0.13] | -0.060 | 0.998 |
| PE-O(38:6) | -0.171 | 194.779 | 0.865 | [-0.11, 0.09] | -0.001 | 0.998 |
| PE-O(39:1) | -0.029 | 202.117 | 0.977 | [-0.28, 0.27] | -0.001 | 0.998 |
| PE-O(40:4) | -0.120 | 201.432 | 0.904 | [-0.38, 0.34] | -0.005 | 0.998 |
| PE-O(40:6) | -0.168 | 202.748 | 0.867 | [-0.38, 0.32] | -0.004 | 0.998 |
| Cer(d18:1/16:0) | 0.225 | 201.574 | 0.822 | [-0.23, 0.29] | 0.004 | 0.998 |
| Cer(d18:2/16:0) | -0.084 | 197.803 | 0.933 | [-0.25, 0.23] | -0.001 | 0.998 |
| Cer(d16:1/20:0) | 0.017 | 192.443 | 0.987 | [-0.31, 0.31] | 0.001 | 0.998 |
| Cer(d18:1/18:0) | -0.029 | 203.947 | 0.977 | [-0.32, 0.31] | -0.001 | 0.998 |
| Cer(d18:2/18:0) | -0.507 | 181.554 | 0.613 | [-0.36, 0.21] | -0.018 | 0.998 |
| Cer(d16:1/22:0) | -0.144 | 200.797 | 0.886 | [-0.4, 0.34] | -0.007 | 0.998 |
| Cer(d18:1/20:0) | -0.170 | 197.598 | 0.865 | [-0.41, 0.34] | -0.009 | 0.998 |
| Cer(d18:0/22:0) | -0.615 | 203.951 | 0.539 | [-0.25, 0.13] | -0.008 | 0.998 |
| Cer(d18:1/22:0) | -0.182 | 196.883 | 0.856 | [-0.32, 0.27] | -0.005 | 0.998 |
| Cer(d16:1/24:1) | -0.982 | 199.339 | 0.327 | [-0.4, 0.13] | -0.023 | 0.998 |
| Cer(d18:1/23:0) | 0.265 | 205.963 | 0.792 | [-0.4, 0.52] | 0.022 | 0.998 |
| Cer(d18:2/23:0) | 0.031 | 204.845 | 0.975 | [-0.31, 0.32] | 0.001 | 0.998 |
| Cer(d18:1/24:0) | 0.050 | 204.623 | 0.960 | [-0.3, 0.32] | 0.001 | 0.998 |
| Cer(d18:1/24:1) | 0.100 | 204.285 | 0.920 | [-0.44, 0.49] | 0.007 | 0.998 |
| Cer(d18:2/24:0) | 0.049 | 198.939 | 0.961 | [-0.24, 0.25] | 0.001 | 0.998 |
| Cer(d18:2/24:1) | -0.310 | 200.876 | 0.757 | [-0.26, 0.19] | -0.004 | 0.998 |
| Cer(d18:1/25:0) | -0.590 | 198.616 | 0.556 | [-0.31, 0.17] | -0.011 | 0.998 |
| Cer(d18:2/25:0) | -0.790 | 167.187 | 0.431 | [-0.5, 0.21] | -0.041 | 0.998 |
| DihydroCer(24:0) | -1.779 | 153.427 | 0.077 | [-0.86, 0.05] | -0.048 | 0.998 |
| HexCer(16:0) | -0.636 | 194.690 | 0.526 | [-0.36, 0.18] | -0.022 | 0.998 |
| HexCer(20:0) | -0.416 | 193.358 | 0.678 | [-0.33, 0.21] | -0.011 | 0.998 |
| HexCer(22:0) | -0.563 | 195.559 | 0.574 | [-0.32, 0.18] | -0.011 | 0.998 |
| HexCer(24:0) | -0.530 | 197.391 | 0.597 | [-0.47, 0.27] | -0.026 | 0.998 |
| HexCer(24:1) | 0.139 | 194.354 | 0.890 | [-0.25, 0.29] | 0.005 | 0.998 |
| CE(16:0) | 0.443 | 186.933 | 0.658 | [-0.23, 0.37] | 0.014 | 0.998 |
| CE(16:1) | -0.531 | 196.055 | 0.596 | [-0.3, 0.17] | -0.010 | 0.998 |
| CE(18:0) | -1.114 | 198.025 | 0.267 | [-0.4, 0.11] | -0.024 | 0.998 |
| CE(18:1) | -1.070 | 194.964 | 0.286 | [-0.42, 0.12] | -0.031 | 0.998 |
| CE(18:2) | -0.834 | 183.351 | 0.405 | [-0.35, 0.14] | -0.017 | 0.998 |
| CE(18:3) | 1.751 | 205.282 | 0.081 | [-0.03, 0.58] | 0.188 | 0.998 |
| CE(18:4) | 0.290 | 204.587 | 0.772 | [-0.16, 0.21] | 0.003 | 0.998 |
| CE(20:2) | -1.196 | 193.420 | 0.233 | [-0.59, 0.14] | -0.061 | 0.998 |
| CE(20:3) | -0.246 | 202.729 | 0.806 | [-0.46, 0.36] | -0.015 | 0.998 |
| CE(20:4) | -0.184 | 205.702 | 0.854 | [-0.31, 0.25] | -0.006 | 0.998 |
| CE(20:5) | 0.792 | 199.483 | 0.429 | [-0.2, 0.47] | 0.028 | 0.998 |
| CE(22:4) | -0.275 | 202.587 | 0.783 | [-0.65, 0.49] | -0.034 | 0.998 |
| CE(22:5) | -0.399 | 203.800 | 0.690 | [-0.75, 0.5] | -0.051 | 0.998 |
| CE(22:6) | 0.138 | 201.982 | 0.890 | [-0.56, 0.64] | 0.014 | 0.998 |
| DG(14:1/16:0) | 0.381 | 205.359 | 0.703 | [-0.56, 0.83] | 0.040 | 0.998 |
| DG(14:0/16:0) | 0.082 | 203.898 | 0.935 | [-0.59, 0.64] | 0.016 | 0.998 |
| DG(14:0/18:1) | -0.634 | 203.462 | 0.527 | [-1.06, 0.54] | -0.088 | 0.998 |
| DG(14:0/18:2) | -0.918 | 168.518 | 0.360 | [-0.39, 0.14] | -0.017 | 0.998 |
| DG(15:0/18:2) | -0.837 | 201.452 | 0.403 | [-1.1, 0.44] | -0.121 | 0.998 |
| DG(16:0/16:0) | -0.657 | 203.560 | 0.512 | [-1.08, 0.54] | -0.123 | 0.998 |
| DG(16:0/16:1) | -1.279 | 202.313 | 0.202 | [-1.38, 0.29] | -0.183 | 0.998 |
| DG(16:0/18:0) | -0.025 | 205.638 | 0.980 | [-0.66, 0.65] | -0.002 | 0.998 |
| DG(16:0/18:1) | -0.684 | 201.822 | 0.495 | [-0.87, 0.42] | -0.048 | 0.998 |
| DG(16:0/18:2) | -0.741 | 198.565 | 0.460 | [-0.98, 0.45] | -0.088 | 0.998 |
| DG(16:0/18:3) | -0.463 | 198.202 | 0.644 | [-0.89, 0.55] | -0.050 | 0.998 |
| DG(16:0/20:4) | -0.469 | 200.969 | 0.640 | [-1.03, 0.63] | -0.059 | 0.998 |
| DG(16:0/22:5) | 0.322 | 202.807 | 0.748 | [-0.55, 0.76] | 0.055 | 0.998 |
| DG(16:0/22:6) | -0.036 | 199.111 | 0.972 | [-0.68, 0.65] | -0.004 | 0.998 |
| DG(16:1/18:0) | -0.270 | 194.130 | 0.787 | [-0.2, 0.15] | -0.003 | 0.998 |
| DG(16:1/18:1) | -0.543 | 200.057 | 0.587 | [-0.81, 0.46] | -0.065 | 0.998 |
| DG(16:1/18:2) | -0.686 | 195.314 | 0.493 | [-0.97, 0.47] | -0.082 | 0.998 |
| DG(16:1/22:6) | -0.013 | 201.577 | 0.990 | [-0.48, 0.47] | -0.001 | 0.999 |
| DG(18:0/18:1) | 0.363 | 200.205 | 0.717 | [-0.44, 0.63] | 0.030 | 0.998 |
| DG(18:0/18:2) | -0.281 | 199.847 | 0.779 | [-0.81, 0.6] | -0.019 | 0.998 |
| DG(18:0/18:3) | -0.204 | 204.298 | 0.839 | [-0.64, 0.52] | -0.010 | 0.998 |
| DG(18:1/18:1) | -0.562 | 202.036 | 0.575 | [-0.8, 0.44] | -0.070 | 0.998 |
| DG(18:1/18:2) | -0.736 | 199.836 | 0.463 | [-0.84, 0.38] | -0.048 | 0.998 |
| DG(18:1/18:3) | -0.145 | 200.196 | 0.885 | [-0.65, 0.56] | -0.008 | 0.998 |
| DG(18:1/20:1) | -0.417 | 200.321 | 0.677 | [-0.73, 0.48] | -0.023 | 0.998 |
| DG(18:1/20:2) | -0.885 | 205.358 | 0.377 | [-0.33, 0.13] | -0.019 | 0.998 |
| DG(18:1/20:3) | -0.504 | 200.185 | 0.615 | [-0.76, 0.45] | -0.031 | 0.998 |
| DG(18:1/20:4) | -0.401 | 197.771 | 0.689 | [-0.71, 0.47] | -0.023 | 0.998 |
| DG(18:1/20:5) | -0.171 | 192.998 | 0.864 | [-0.63, 0.53] | -0.011 | 0.998 |
| DG(18:1/22:5) | -0.017 | 200.948 | 0.987 | [-0.61, 0.6] | -0.002 | 0.998 |
| DG(18:1/22:6) | -0.382 | 196.551 | 0.703 | [-0.65, 0.44] | -0.021 | 0.998 |
| DG(18:2/18:2) | 0.132 | 202.770 | 0.895 | [-0.49, 0.56] | 0.008 | 0.998 |
| DG(18:2/18:3) | 0.146 | 202.476 | 0.884 | [-0.49, 0.57] | 0.009 | 0.998 |
| DG(18:2/20:3) | -0.678 | 193.126 | 0.499 | [-0.75, 0.37] | -0.039 | 0.998 |
| DG(18:2/20:4) | 0.011 | 192.921 | 0.991 | [-0.5, 0.51] | 0.001 | 0.999 |
| DG(18:2/20:5) | -0.413 | 194.628 | 0.680 | [-0.61, 0.4] | -0.033 | 0.998 |
| DG(18:2/22:4) | -1.098 | 205.781 | 0.274 | [-0.13, 0.04] | -0.008 | 0.998 |
| DG(18:2/22:5) | -1.150 | 179.836 | 0.252 | [-0.76, 0.2] | -0.068 | 0.998 |
| DG(18:2/22:6) | -0.250 | 200.593 | 0.803 | [-0.71, 0.55] | -0.034 | 0.998 |
| TG(40:0-14:0) | -0.061 | 202.275 | 0.951 | [-0.49, 0.46] | -0.006 | 0.998 |
| TG(42:0-12:0) | -0.458 | 195.120 | 0.647 | [-0.64, 0.4] | -0.032 | 0.998 |
| TG(42:0-14:0) | -0.386 | 201.420 | 0.700 | [-0.75, 0.51] | -0.029 | 0.998 |
| TG(42:0-16:0) | -0.212 | 204.367 | 0.832 | [-0.53, 0.43] | -0.007 | 0.998 |
| TG(42:1-12:0) | -0.101 | 202.596 | 0.920 | [-0.75, 0.67] | -0.009 | 0.998 |
| TG(42:1-16:0) | 0.019 | 204.538 | 0.985 | [-0.59, 0.6] | 0.002 | 0.998 |
| TG(42:1-18:1) | -0.382 | 201.423 | 0.703 | [-0.54, 0.36] | -0.012 | 0.998 |
| TG(42:2-18:2) | -0.270 | 201.863 | 0.787 | [-0.55, 0.41] | -0.008 | 0.998 |
| TG(44:0-12:0) | -0.221 | 187.840 | 0.826 | [-0.42, 0.33] | -0.003 | 0.998 |
| TG(44:0-14:0) | -0.037 | 204.812 | 0.970 | [-0.5, 0.48] | -0.001 | 0.998 |
| TG(44:0-16:0) | -0.217 | 203.870 | 0.829 | [-0.72, 0.58] | -0.021 | 0.998 |
| TG(44:1-12:0) | -0.380 | 199.231 | 0.705 | [-0.55, 0.37] | -0.012 | 0.998 |
| TG(44:1-14:0) | -0.774 | 192.378 | 0.440 | [-0.71, 0.31] | -0.043 | 0.998 |
| TG(44:1-16:0) | -0.381 | 193.305 | 0.703 | [-0.5, 0.34] | -0.011 | 0.998 |
| TG(44:2-14:0) | -0.225 | 192.021 | 0.822 | [-0.55, 0.44] | -0.011 | 0.998 |
| TG(44:2-16:0) | -0.508 | 197.704 | 0.612 | [-0.56, 0.33] | -0.016 | 0.998 |
| TG(44:2-18:1) | -0.532 | 200.303 | 0.595 | [-0.6, 0.35] | -0.020 | 0.998 |
| TG(44:2-18:2) | -0.220 | 205.874 | 0.826 | [-0.64, 0.51] | -0.024 | 0.998 |
| TG(45:0-14:0) | -0.510 | 192.996 | 0.611 | [-0.58, 0.34] | -0.018 | 0.998 |
| TG(45:0-16:0) | 0.500 | 203.800 | 0.618 | [-0.27, 0.46] | 0.013 | 0.998 |
| TG(46:0-14:0) | -0.648 | 192.926 | 0.518 | [-0.55, 0.28] | -0.019 | 0.998 |
| TG(46:0-16:0) | -1.079 | 187.029 | 0.282 | [-0.69, 0.2] | -0.048 | 0.998 |
| TG(46:0-18:0) | -0.932 | 182.555 | 0.353 | [-0.66, 0.24] | -0.037 | 0.998 |
| TG(46:1-12:0) | -0.052 | 201.413 | 0.959 | [-0.4, 0.38] | -0.002 | 0.998 |
| TG(46:1-14:0) | -0.889 | 182.432 | 0.375 | [-0.61, 0.23] | -0.033 | 0.998 |
| TG(46:1-16:0) | -0.879 | 184.520 | 0.381 | [-0.6, 0.23] | -0.034 | 0.998 |
| TG(46:1-16:1) | -0.858 | 187.179 | 0.392 | [-0.59, 0.23] | -0.028 | 0.998 |
| TG(46:1-18:1) | -0.505 | 190.227 | 0.614 | [-0.8, 0.47] | -0.038 | 0.998 |
| TG(46:2-14:0) | -1.204 | 180.035 | 0.230 | [-0.69, 0.17] | -0.059 | 0.998 |
| TG(46:2-14:1) | -0.916 | 193.866 | 0.361 | [-0.79, 0.29] | -0.086 | 0.998 |
| TG(46:2-16:0) | -0.221 | 193.663 | 0.825 | [-0.27, 0.21] | -0.006 | 0.998 |
| TG(46:2-16:1) | -1.136 | 189.127 | 0.257 | [-0.7, 0.19] | -0.071 | 0.998 |
| TG(46:2-18:1) | -1.036 | 195.668 | 0.301 | [-0.82, 0.26] | -0.076 | 0.998 |
| TG(46:2-18:2) | -0.962 | 188.932 | 0.337 | [-0.7, 0.24] | -0.080 | 0.998 |
| TG(46:3-16:0) | -0.394 | 203.034 | 0.694 | [-0.7, 0.46] | -0.050 | 0.998 |
| TG(46:3-16:1) | 0.305 | 205.479 | 0.760 | [-0.43, 0.58] | 0.017 | 0.998 |
| TG(46:3-18:2) | -0.128 | 202.143 | 0.898 | [-0.47, 0.41] | -0.006 | 0.998 |
| TG(46:3-18:3) | 0.153 | 204.943 | 0.878 | [-0.4, 0.47] | 0.006 | 0.998 |
| TG(47:0-14:0) | 0.240 | 202.685 | 0.810 | [-0.48, 0.61] | 0.019 | 0.998 |
| TG(47:0-15:0) | 0.080 | 200.698 | 0.936 | [-0.38, 0.41] | 0.003 | 0.998 |
| TG(48:0-14:0) | 0.205 | 199.439 | 0.837 | [-0.38, 0.47] | 0.009 | 0.998 |
| TG(48:0-16:0) | 1.036 | 204.602 | 0.301 | [-0.06, 0.2] | 0.006 | 0.998 |
| TG(48:0-18:0) | -0.453 | 191.704 | 0.651 | [-0.5, 0.32] | -0.018 | 0.998 |
| TG(48:1-12:0) | -0.254 | 186.682 | 0.799 | [-0.44, 0.34] | -0.012 | 0.998 |
| TG(48:1-14:0) | -0.003 | 203.271 | 0.998 | [-0.44, 0.44] | 0.000 | 0.999 |
| TG(48:1-16:0) | -0.582 | 198.239 | 0.561 | [-0.71, 0.38] | -0.036 | 0.998 |
| TG(48:1-16:1) | -0.066 | 201.466 | 0.948 | [-0.39, 0.36] | -0.001 | 0.998 |
| TG(48:1-18:0) | -0.079 | 202.182 | 0.937 | [-0.46, 0.43] | -0.003 | 0.998 |
| TG(48:1-18:1) | -0.378 | 202.958 | 0.705 | [-0.58, 0.39] | -0.017 | 0.998 |
| TG(48:2-12:0) | -0.129 | 201.407 | 0.897 | [-0.39, 0.34] | -0.003 | 0.998 |
| TG(48:2-14:0) | -0.639 | 195.700 | 0.524 | [-0.54, 0.27] | -0.020 | 0.998 |
| TG(48:2-14:1) | -0.385 | 200.039 | 0.700 | [-0.38, 0.26] | -0.006 | 0.998 |
| TG(48:2-16:0) | -0.639 | 177.991 | 0.524 | [-0.37, 0.19] | -0.008 | 0.998 |
| TG(48:2-16:1) | -0.018 | 202.696 | 0.986 | [-0.34, 0.34] | 0.000 | 0.998 |
| TG(48:2-18:0) | -0.896 | 201.748 | 0.372 | [-0.64, 0.24] | -0.042 | 0.998 |
| TG(48:2-18:1) | -0.483 | 199.799 | 0.629 | [-0.41, 0.25] | -0.009 | 0.998 |
| TG(48:2-18:2) | -0.452 | 200.351 | 0.652 | [-0.38, 0.24] | -0.008 | 0.998 |
| TG(48:3-12:0) | -0.605 | 199.610 | 0.546 | [-0.43, 0.23] | -0.013 | 0.998 |
| TG(48:3-14:0) | -0.668 | 198.282 | 0.505 | [-0.43, 0.21] | -0.013 | 0.998 |
| TG(48:3-14:1) | -0.472 | 196.917 | 0.638 | [-0.4, 0.25] | -0.009 | 0.998 |
| TG(48:3-16:1) | -0.546 | 194.130 | 0.586 | [-0.4, 0.23] | -0.011 | 0.998 |
| TG(48:3-18:1) | -0.822 | 194.384 | 0.412 | [-0.46, 0.19] | -0.016 | 0.998 |
| TG(48:3-18:2) | -0.350 | 197.740 | 0.727 | [-0.52, 0.36] | -0.012 | 0.998 |
| TG(48:3-18:3) | -0.392 | 198.347 | 0.695 | [-0.14, 0.09] | -0.003 | 0.998 |
| TG(48:4-12:0) | -0.183 | 205.319 | 0.855 | [-0.16, 0.13] | -0.003 | 0.998 |
| TG(48:4-14:0) | -0.988 | 186.903 | 0.324 | [-0.48, 0.16] | -0.023 | 0.998 |
| TG(48:4-16:1) | -0.267 | 196.165 | 0.789 | [-0.37, 0.28] | -0.010 | 0.998 |
| TG(48:4-18:3) | -0.434 | 201.072 | 0.665 | [-0.46, 0.3] | -0.015 | 0.998 |
| TG(48:5-18:2) | -0.602 | 198.516 | 0.548 | [-0.44, 0.23] | -0.016 | 0.998 |
| TG(48:5-18:3) | -0.543 | 195.908 | 0.588 | [-0.46, 0.26] | -0.018 | 0.998 |
| TG(49:0-16:0) | -0.492 | 201.244 | 0.623 | [-0.69, 0.41] | -0.020 | 0.998 |
| TG(49:1-15:0) | -0.828 | 197.057 | 0.409 | [-0.61, 0.25] | -0.030 | 0.998 |
| TG(49:1-16:0) | -1.436 | 186.296 | 0.153 | [-0.76, 0.12] | -0.069 | 0.998 |
| TG(49:1-17:0) | -0.829 | 187.143 | 0.408 | [-0.57, 0.23] | -0.034 | 0.998 |
| TG(49:1-18:1) | -0.435 | 197.775 | 0.664 | [-0.41, 0.26] | -0.012 | 0.998 |
| TG(49:2-15:0) | -1.165 | 180.144 | 0.245 | [-1.15, 0.3] | -0.086 | 0.998 |
| TG(49:2-18:2) | -0.471 | 197.466 | 0.638 | [-0.44, 0.27] | -0.015 | 0.998 |
| TG(49:3-18:2) | -0.102 | 201.884 | 0.919 | [-0.32, 0.29] | -0.002 | 0.998 |
| TG(50:0-16:0) | -1.263 | 188.857 | 0.208 | [-0.61, 0.13] | -0.047 | 0.998 |
| TG(50:1-14:0) | -1.055 | 194.345 | 0.293 | [-0.82, 0.25] | -0.055 | 0.998 |
| TG(50:1-16:0) | -0.241 | 198.098 | 0.810 | [-0.42, 0.33] | -0.006 | 0.998 |
| TG(50:1-16:1) | 0.058 | 199.994 | 0.954 | [-0.35, 0.37] | 0.001 | 0.998 |
| TG(50:1-18:0) | 0.387 | 198.703 | 0.699 | [-0.28, 0.42] | 0.009 | 0.998 |
| TG(50:1-18:1) | -0.163 | 197.987 | 0.871 | [-0.32, 0.27] | -0.002 | 0.998 |
| TG(50:2-14:0) | -0.025 | 190.102 | 0.980 | [-0.31, 0.3] | -0.001 | 0.998 |
| TG(50:2-16:0) | -0.628 | 194.736 | 0.531 | [-0.32, 0.17] | -0.007 | 0.998 |
| TG(50:2-16:1) | 0.210 | 197.652 | 0.834 | [-0.19, 0.23] | 0.003 | 0.998 |
| TG(50:2-18:0) | -0.018 | 196.997 | 0.986 | [-0.27, 0.27] | 0.000 | 0.998 |
| TG(50:2-18:1) | -0.476 | 201.749 | 0.635 | [-0.42, 0.25] | -0.014 | 0.998 |
| TG(50:2-18:2) | -0.264 | 197.822 | 0.792 | [-0.28, 0.21] | -0.003 | 0.998 |
| TG(50:3-14:0) | -0.052 | 199.314 | 0.958 | [-0.25, 0.24] | -0.001 | 0.998 |
| TG(50:3-16:0) | -0.611 | 192.741 | 0.542 | [-0.34, 0.18] | -0.008 | 0.998 |
| TG(50:3-16:1) | -0.601 | 196.611 | 0.549 | [-0.38, 0.2] | -0.011 | 0.998 |
| TG(50:3-18:1) | -0.657 | 193.721 | 0.512 | [-0.36, 0.18] | -0.010 | 0.998 |
| TG(50:3-18:2) | -0.651 | 196.082 | 0.516 | [-0.34, 0.17] | -0.008 | 0.998 |
| TG(50:3-18:3) | -0.911 | 198.334 | 0.363 | [-0.42, 0.15] | -0.016 | 0.998 |
| TG(50:4-14:0) | -0.737 | 192.126 | 0.462 | [-0.42, 0.19] | -0.015 | 0.998 |
| TG(50:4-14:1) | -0.389 | 194.774 | 0.698 | [-0.22, 0.15] | -0.003 | 0.998 |
| TG(50:4-16:0) | -0.634 | 196.893 | 0.527 | [-0.39, 0.2] | -0.013 | 0.998 |
| TG(50:4-16:1) | -0.669 | 188.595 | 0.504 | [-0.36, 0.18] | -0.011 | 0.998 |
| TG(50:4-18:1) | -0.812 | 192.065 | 0.418 | [-0.43, 0.18] | -0.016 | 0.998 |
| TG(50:4-18:2) | -0.494 | 188.985 | 0.622 | [-0.59, 0.36] | -0.036 | 0.998 |
| TG(50:4-18:3) | -0.649 | 195.636 | 0.517 | [-0.57, 0.29] | -0.030 | 0.998 |
| TG(50:5-14:0) | -1.577 | 189.580 | 0.117 | [-0.76, 0.08] | -0.079 | 0.998 |
| TG(50:5-18:3) | -0.403 | 196.018 | 0.687 | [-0.45, 0.3] | -0.020 | 0.998 |
| TG(51:2-16:0) | -1.281 | 193.345 | 0.202 | [-0.58, 0.12] | -0.056 | 0.998 |
| TG(51:2-17:0) | -1.028 | 191.774 | 0.305 | [-0.61, 0.19] | -0.047 | 0.998 |
| TG(51:2-18:1) | -0.487 | 201.777 | 0.627 | [-0.37, 0.22] | -0.010 | 0.998 |
| TG(51:2-18:2) | 0.911 | 205.757 | 0.363 | [-0.07, 0.2] | 0.013 | 0.998 |
| TG(51:3-15:0) | -0.865 | 199.165 | 0.388 | [-0.49, 0.19] | -0.023 | 0.998 |
| TG(52:1-16:0) | -1.082 | 191.796 | 0.280 | [-0.65, 0.19] | -0.062 | 0.998 |
| TG(52:1-18:0) | -0.121 | 191.575 | 0.904 | [-0.27, 0.23] | -0.002 | 0.998 |
| TG(52:1-18:1) | -0.359 | 194.177 | 0.720 | [-0.31, 0.21] | -0.005 | 0.998 |
| TG(52:2-16:0) | -0.306 | 194.412 | 0.760 | [-0.33, 0.24] | -0.006 | 0.998 |
| TG(52:2-18:2) | -1.225 | 199.772 | 0.222 | [-0.61, 0.14] | -0.039 | 0.998 |
| TG(52:3-16:0) | -1.008 | 200.620 | 0.315 | [-0.54, 0.17] | -0.026 | 0.998 |
| TG(52:3-16:1) | -1.140 | 188.206 | 0.256 | [-0.72, 0.19] | -0.045 | 0.998 |
| TG(52:3-18:0) | -1.021 | 186.143 | 0.308 | [-0.56, 0.18] | -0.045 | 0.998 |
| TG(52:3-18:1) | -0.048 | 202.933 | 0.962 | [-0.32, 0.3] | -0.001 | 0.998 |
| TG(52:3-18:2) | 0.638 | 164.750 | 0.525 | [-0.1, 0.2] | 0.005 | 0.998 |
| TG(52:4-16:0) | -1.643 | 182.777 | 0.102 | [-0.81, 0.07] | -0.080 | 0.998 |
| TG(52:4-16:1) | -0.351 | 190.349 | 0.726 | [-0.2, 0.14] | -0.006 | 0.998 |
| TG(52:4-18:1) | -0.412 | 188.694 | 0.681 | [-0.34, 0.23] | -0.023 | 0.998 |
| TG(52:4-18:2) | -0.512 | 185.285 | 0.609 | [-0.5, 0.3] | -0.017 | 0.998 |
| TG(52:4-18:3) | 0.434 | 194.384 | 0.665 | [-0.18, 0.28] | 0.015 | 0.998 |
| TG(52:5-16:0) | 3.169 | 205.064 | 0.002 | [0.21, 0.89] | 0.213 | 0.673 |
| TG(52:5-16:1) | 1.282 | 203.983 | 0.201 | [-0.11, 0.51] | 0.059 | 0.998 |
| TG(52:5-18:2) | 0.322 | 190.786 | 0.748 | [-0.26, 0.36] | 0.013 | 0.998 |
| TG(52:5-18:3) | 0.983 | 202.253 | 0.327 | [-0.12, 0.36] | 0.021 | 0.998 |
| TG(52:5-20:3) | 0.088 | 196.312 | 0.930 | [-0.22, 0.24] | 0.002 | 0.998 |
| TG(52:6-16:0) | 0.578 | 200.100 | 0.564 | [-0.16, 0.3] | 0.019 | 0.998 |
| TG(52:6-20:4) | 1.296 | 201.052 | 0.196 | [-0.05, 0.25] | 0.018 | 0.998 |
| TG(53:2-16:0) | 2.365 | 203.994 | 0.019 | [0.03, 0.31] | 0.030 | 0.998 |
| TG(53:3-16:0) | 0.204 | 198.283 | 0.839 | [-0.48, 0.59] | 0.011 | 0.998 |
| TG(54:2-18:2) | 0.563 | 201.313 | 0.574 | [-0.14, 0.25] | 0.010 | 0.998 |
| TG(54:3-18:0) | -0.021 | 204.108 | 0.983 | [-0.3, 0.3] | 0.000 | 0.998 |
| TG(54:4-16:0) | 0.899 | 204.233 | 0.370 | [-0.11, 0.29] | 0.012 | 0.998 |
| TG(54:4-16:1) | 2.316 | 205.965 | 0.022 | [0.04, 0.54] | 0.060 | 0.998 |
| TG(54:5-18:1) | 0.700 | 202.777 | 0.485 | [-0.11, 0.23] | 0.007 | 0.998 |
| TG(54:5-18:2) | 1.642 | 202.218 | 0.102 | [-0.04, 0.4] | 0.022 | 0.998 |
| TG(54:5-18:3) | 0.711 | 200.664 | 0.478 | [-0.11, 0.24] | 0.007 | 0.998 |
| TG(54:5-20:3) | 0.431 | 201.433 | 0.667 | [-0.11, 0.17] | 0.004 | 0.998 |
| TG(54:5-20:4) | 1.466 | 203.657 | 0.144 | [-0.06, 0.43] | 0.029 | 0.998 |
| TG(54:6-16:0) | 2.207 | 204.785 | 0.028 | [0.03, 0.53] | 0.063 | 0.998 |
| TG(54:6-16:1) | 1.521 | 196.381 | 0.130 | [-0.05, 0.41] | 0.046 | 0.998 |
| TG(54:6-18:3) | 0.927 | 201.006 | 0.355 | [-0.09, 0.25] | 0.012 | 0.998 |
| TG(54:8-18:3) | -0.497 | 173.481 | 0.620 | [-0.37, 0.22] | -0.019 | 0.998 |
| LacCer(16:0) | 1.638 | 196.887 | 0.103 | [-0.03, 0.32] | 0.020 | 0.998 |
| LacCer(24:1) | 1.479 | 153.840 | 0.141 | [-0.06, 0.42] | 0.028 | 0.998 |
| Cholesterol | 1.362 | 191.918 | 0.175 | [-0.06, 0.34] | 0.021 | 0.998 |
| PG(16:0/16:0) | 2.271 | 189.004 | 0.024 | [0.03, 0.39] | 0.028 | 0.998 |
| PG(16:0/18:0) | -0.048 | 193.355 | 0.962 | [-0.65, 0.62] | -0.003 | 0.998 |
| PG(18:0/18:1) | -1.936 | 205.952 | 0.054 | [-0.35, 0] | -0.029 | 0.998 |
| PG(18:0/18:2) | 0.024 | 181.434 | 0.981 | [-0.21, 0.22] | 0.000 | 0.998 |
| PG(18:1/16:1) | -1.133 | 191.207 | 0.259 | [-0.38, 0.1] | -0.017 | 0.998 |
| PG(18:2/16:1) | 0.760 | 204.937 | 0.448 | [-0.06, 0.13] | 0.005 | 0.998 |
| PG(20:0/18:2) | -0.080 | 188.384 | 0.936 | [-0.17, 0.16] | -0.001 | 0.998 |
| PG(20:0/20:4) | -1.492 | 205.122 | 0.137 | [-0.46, 0.06] | -0.024 | 0.998 |
| PI(14:0/18:2) | 1.320 | 203.925 | 0.188 | [-0.05, 0.23] | 0.011 | 0.998 |
| PI(14:0/20:4) | -0.431 | 188.548 | 0.667 | [-0.37, 0.24] | -0.013 | 0.998 |
| PI(16:0/18:3) | 0.310 | 205.070 | 0.757 | [-0.44, 0.6] | 0.122 | 0.998 |
| PI(16:0/20:4) | -0.673 | 204.624 | 0.501 | [-0.37, 0.18] | -0.018 | 0.998 |
| PI(16:0/22:6) | 0.149 | 205.211 | 0.882 | [-0.38, 0.44] | 0.016 | 0.998 |
| PI(18:0/18:2) | -0.380 | 190.182 | 0.704 | [-0.18, 0.12] | -0.003 | 0.998 |
| PI(18:0/20:0) | 1.004 | 198.208 | 0.317 | [-0.09, 0.29] | 0.009 | 0.998 |
| PI(18:0/20:4) | -1.805 | 205.147 | 0.072 | [-0.24, 0.01] | -0.016 | 0.998 |
| PS(18:0/18:0) | -1.979 | 184.352 | 0.049 | [-1.06, 0] | -0.327 | 0.998 |
| PS(18:0/20:0) | -1.784 | 205.121 | 0.076 | [-0.24, 0.01] | -0.027 | 0.998 |
| PS(18:0/20:4) | -1.113 | 204.519 | 0.267 | [-0.49, 0.14] | -0.047 | 0.998 |
| PS(20:0/18:2) | 0.842 | 206.000 | 0.401 | [-0.52, 1.29] | 0.822 | 0.998 |
| PS(20:0/20:3) | 0.052 | 198.246 | 0.958 | [-0.24, 0.25] | 0.002 | 0.998 |
| PA(18:0/20:4) | -0.376 | 200.470 | 0.707 | [-0.39, 0.26] | -0.071 | 0.998 |
| PA(18:0/22:6) | -1.395 | 204.014 | 0.165 | [-1.66, 0.28] | 1.191 | 0.998 |
| PA(20:0/18:2) | -1.218 | 201.838 | 0.225 | [-0.34, 0.08] | -0.014 | 0.998 |
| PA(20:0/20:4) | -0.784 | 192.647 | 0.434 | [-0.51, 0.22] | -0.055 | 0.998 |
| dof, degrees of freedom; 95% CI, 95% confidence interval; Log2FC, log2 fold change; FDR, false discovery rate. T-values are reported with treatment responder group as the reference group. | | | | | | |

| Supplementary Table S5. Annotation of SNPs in the multi-omics cohort. | | | | | | | |
| --- | --- | --- | --- | --- | --- | --- | --- |
| **rsID** | **Nearest Gene** | **cepip** | **FitCons2** | **FUN-LDA** | **GenoNet** | **GSL-P** | **Combined Rank in LD** |
| rs66950781 | *DCAF6* | 0.2237 (83) | 0.1327 (38) | 0 (71) | 0.0175 (72) | 0.0012 (58) | **62.3** |
| rs66950781 | *DCAF6* | 0.2154 | 0.1327 | 0 | 0.0175 | 0.0012 | NO LD |
| rs66950781 | *DCAF6* | 0.2293 | 0.1327 | 0 | 0.0175 | 0.0012 | NO LD |
| rs4651220 | *C1orf21* | 0.1996 (44) | 0.0651 (22.5) | 0 (29) | 0.0298 (5) | 0.0012 (26) | **20.6** |
| rs10765863 | *GALNT18* | 0.5192 (1) | 0.1240 (1) | 0.0032 (1) | 0.0313 (1) | 0.0103 (1) | **1** |
| rs77263493 | *PRMT3* | 0.0920 (7) | 0.0870 (5) | 1.000e-5 (15.5) | 0.0194 (26.5) | 0.0012 (15) | **11.7** |
| rs10895073 | *MED28P5* | 0.0358 (3) | 0.0429 (10) | 1.000e-5 (6.5) | 0.0197 (12.5) | 0.0012 (10.5) | **7.6** |
| rs3928509 | *PRICKLE1* | 0.1069 (5) | 0.3274 (1) | 0 (4) | 0.0208 (4) | 0.0012 (3.5) | **3.1** |
| rs10878666 | *DYRK2* | 0.1901 (3) | 0.0790 (4) | 5.000e-5 (3) | 0.0204 (4) | 0.0012 (4.5) | **3.7** |
| rs10878666 | *DYRK2* | 0.2369 | 0.079 | 5.00E-05 | 0.0204 | 0.0012 | NO LD |
| rs79557688 | *SPATA13* | 0.2358 (5) | 0.1240 (1) | 1.800e-4 (1) | 0.0308 (1) | 0.0012 (4) | **1.8** |
| rs1576377 | *PCDH20* | 0.0665 | 0.0515 | 0 | 0.0217 | 0.0012 | NO LD |
| rs1576377 | *PCDH20* | 0.1000 (21) | 0.0515 (10.5) | 0 (41) | 0.0217 (24.5) | 0.0012 (29.5) | **23.1** |
| rs288667 | *PPIAP24* | 0.0857 (11) | 0.0429 (7.5) | 2.000e-5 (4) | 0.0205 (7) | 0.0012 (7.5) | **7** |
| rs288667 | *PPIAP24* | 0.1071 | 0.0429 | 2.00E-05 | 0.0205 | 0.0012 | NO LD |
| rs17093696 | *SLC35F4* | 0.1225 (15) | 0.0515 (4) | 0 (22.5) | 0.0231 (6) | 0.0012 (16) | **10.5** |
| rs77511574 | *SECISBP2L* | 0.3243 (2) | 0.0870 (10) | 1.000e-5 (4) | 0.0229 (5) | 0.0012 (9.5) | **5.2** |
| rs112786565 | *NFIC* | 0.2533 | 0.1327 | 0 | 0.0222 | 0.0012 | NO LD |
| rs112786565 | *NFIC* | 0.2376 (2) | 0.1327 (2) | 0 (2) | 0.0222 (2) | 0.0012 (1.5) | **1.9** |
| rs1468460 | *COPE* | 0.6025 (4) | 0.1645 (9.5) | 0 (24.5) | 0.0050 (30) | 0.0079 (5.5) | **10.9** |
| rs576778703 | *TSHZ3* | 0.0602 (8) | 0.0342 (9) | 0 (5.5) | 0.0222 (5) | 0.0012 (5) | **6.3** |
| rs7559601 | *SLC25A5P2* | 0.0159 (10) | 0.0429 (11.5) | 2.000e-5 (4) | 0.0201 (7.5) | 0.0012 (11.5) | **8.3** |
| rs56097024 | *ZDBF2* | 0.3433 (4) | 0.0651 (33.5) | 0 (105) | 0.0181 (115) | 0.0058 (4) | **23** |
| rs7586824 | *C2orf83* | 0.2272 (1) | 0.0870 (1) | 0 (5) | 0.0294 (1) | 0.0012 (4.5) | **1.9** |
| rs7650872 | *RAB7A* | 0.2472 | 0.0429 | 0 | 0.021 | 0.0012 | NO LD |
| rs7650872 | *RAB7A* | 0.2317 (10) | 0.0429 (8.5) | 0 (9) | 0.0210 (8) | 0.0012 (8) | **8.7** |
| rs13086596 | *FGF12* | 0.0907 (2) | 0.0429 (2) | 0 (2) | 0.0189 (2) | 0.0012 (2) | **2** |
| rs13086596 | *FGF12* | 0.0746 | 0.0429 | 0 | 0.0189 | 0.0012 | NO LD |
| rs12500066 | *PCDH18* | 0.0181 (11) | 0.0429 (7.5) | 2.000e-5 (1.5) | 0.0212 (7) | 0.0012 (7) | **5.7** |
| rs71626581 | *CENPK* | 0.3141 (74) | 0.1327 (144) | 0 (215) | 0.0192 (74) | 0.0012 (182) | **125.3** |
| rs348907 | *EDIL3* | 0.1578 (15) | 0.0429 (18) | 0 (23.5) | 0.0159 (16) | 0.0012 (17) | **17.7** |
| rs6877634 | *RASGEF1C* | 0.0475 | 0.0429 | 0 | 0.0189 | 0.0012 | NO LD |
| rs6877634 | *RASGEF1C* | 0.0357 (2) | 0.0429 (2) | 0 (2) | 0.0189 (2) | 0.0012 (2) | **2** |
| rs6877634 | *RASGEF1C* | 0.0481 | 0.0429 | 0 | 0.0189 | 0.0012 | NO LD |
| rs7764214 | *KRT18P38* | 0.0759 (31) | 0.0429 (35.5) | 0 (44) | 0.0204 (31.5) | 0.0012 (34.5) | **35** |
| rs8191692 | *IGF2R* | 0.6239 (3) | 0.1953 (6) | 1 (1.5) | 0.0089 (77) | 1.0000 (1.5) | **5** |
| rs36066317 | *RP5-859M6.1* | 0.0382 (13) | 0.0429 (10) | 0 (9) | 0.0221 (9.5) | 0.0012 (8) | **9.8** |
| rs36066317 | *RP5-859M6.1* | 0.0407 | 0.0429 | 0 | 0.0221 | 0.0012 | NO LD |
| rs17442142 | *MAGI2* | 0.1146 (36) | 0.0429 (25) | 5.000e-5 (3) | 0.0186 (31) | 0.0012 (23) | **18.1** |
| rs10953654 | *IMMP2L* | 0.0974 (6) | 0.0429 (5) | 5.000e-5 (1) | 0.0208 (4) | 0.0012 (3.5) | **3.3** |
| rs10234151 | *PLXNA4* | 0.2695 (3) | 0.0515 (20) | 0 (21.5) | 0.0233 (15) | 0.0012 (15.5) | **12.5** |
| rs10511552 | *PTPRD* | 0.0340 (16) | 0.0429 (37) | 0 (39.5) | 0.0204 (34.5) | 0.0012 (28.5) | **29.7** |
| rs11999763 | *LINGO2* | 0.7020 (1) | 0.1463 (1) | 0.6744 (1) | 0.0155 (23) | 0.0040 (1) | **1.9** |
| cepip, the context-dependent epigenomic weighting for regulatory variant prioritization score and rank in LD block; FitCons2, fitness consequences score and rank in LD block; FUN-LDA, FUN-LDA score and rank in LD block; GenoNet, GenoNet score and rank in LD block; GSL-P, GenoSkylinePlus score and rank in LD block. | | | | | | | |

| Supplementary Table S6. Trans-omics KEGG enrichment of efficacy-related molecules in gene/protein and metabolite layers for multi-omics cohort. | | | | | | |
| --- | --- | --- | --- | --- | --- | --- |
| **Pathway** | **hitsG** | **hitsM** | **hitsTotal** | ***P*.ValueG** | ***P*.ValueM** | **Integ *P*.Value** |
| Glycerophospholipid metabolism | 5 | 3 | 8 | 0.0201 | 0.000243 | 3.25E-05 |
| beta-Alanine metabolism | 1 | 3 | 4 | 0.397 | 5.58E-05 | 0.000109 |
| Autophagy - other | 6 | 1 | 7 | 0.0173 | 0.00781 | 0.00127 |
| Leishmaniasis | 5 | 1 | 6 | 0.00441 | 0.0156 | 0.00156 |
| Central carbon metabolism in cancer | 3 | 2 | 5 | 0.186 | 0.00379 | 0.00246 |
| Systemic lupus erythematosus | 4 | 1 | 5 | 0.0737 | 0.00781 | 0.00274 |
| Aminoacyl-tRNA biosynthesis | 3 | 2 | 5 | 0.0906 | 0.0074 | 0.00293 |
| Kaposi sarcoma-associated herpesvirus infection | 5 | 1 | 6 | 0.0723 | 0.013 | 0.00462 |
| Amoebiasis | 5 | 1 | 6 | 0.00752 | 0.0334 | 0.00485 |
| Retrograde endocannabinoid signaling | 1 | 2 | 3 | 0.796 | 0.000996 | 0.00488 |
| Necroptosis | 6 | 1 | 7 | 0.0226 | 0.0258 | 0.00557 |
| Histidine metabolism | 1 | 2 | 3 | 0.313 | 0.00607 | 0.00595 |
| Autophagy - animal | 3 | 1 | 4 | 0.377 | 0.0156 | 0.0169 |
| Choline metabolism in cancer | 2 | 1 | 3 | 0.289 | 0.0284 | 0.024 |
| Sphingolipid signaling pathway | 5 | 1 | 6 | 0.19 | 0.0385 | 0.0247 |
| Phenylalanine, tyrosine and tryptophan biosynthesis | 1 | 1 | 2 | 0.0782 | 0.0878 | 0.038 |
| Sphingolipid metabolism | 2 | 1 | 3 | 0.176 | 0.0635 | 0.0392 |
| ABC transporters | 2 | 2 | 4 | 0.342 | 0.0465 | 0.0433 |
| Vitamin digestion and absorption | 4 | 1 | 5 | 0.0826 | 0.0974 | 0.0437 |
| Pantothenate and CoA biosynthesis | 1 | 1 | 2 | 0.266 | 0.0708 | 0.0553 |
| Linoleic acid metabolism | 1 | 1 | 2 | 0.377 | 0.0708 | 0.0689 |
| alpha-Linolenic acid metabolism | 1 | 1 | 2 | 0.335 | 0.109 | 0.0966 |
| Phenylalanine metabolism | 1 | 1 | 2 | 0.242 | 0.146 | 0.109 |
| Glycine, serine and threonine metabolism | 1 | 1 | 2 | 0.479 | 0.123 | 0.136 |
| Arachidonic acid metabolism | 1 | 1 | 2 | 0.643 | 0.18 | 0.235 |
| Note: hitsG and *P*.ValueG refer to the number of molecular hits and *P* values in the gene/protein layer, respectively; hitsM and *P*.ValueM refer to the number of molecular hits and *P* values in the metabolite layer. In the trans-omics KEGG analysis, only pathways with molecular hits in both gene/protein and metabolite layers were retained and ranked by the integrated *P* value (Integ *P*.Value). | | | | | | |

| Supplementary Table S7. Biological Process (BP) GO enrichment for gene and protein layers in the multi-omics cohort. | | | | | |
| --- | --- | --- | --- | --- | --- |
| **Pathway** | **Total** | **Expected** | **Hits** | ***P.*Value** | **FDR** |
| response to oxidative stress | 279 | 3.32 | 17 | 3.74E-08 | 3.07E-05 |
| enzyme linked receptor protein signaling pathway | 1180 | 14 | 35 | 3.15E-07 | 0.000106 |
| response to wounding | 1310 | 15.6 | 37 | 5.33E-07 | 0.000106 |
| regulation of catalytic activity | 1730 | 20.6 | 44 | 6.26E-07 | 0.000106 |
| regulation of cellular protein metabolic process | 1560 | 18.5 | 41 | 6.82E-07 | 0.000106 |
| regulation of molecular function | 2250 | 26.7 | 52 | 7.94E-07 | 0.000106 |
| positive regulation of cellular metabolic process | 2530 | 30.1 | 56 | 0.000001 | 0.000106 |
| regulation of protein metabolic process | 1820 | 21.7 | 45 | 1.03E-06 | 0.000106 |
| behavior | 597 | 7.1 | 22 | 2.39E-06 | 0.0002 |
| positive regulation of multicellular organismal process | 649 | 7.72 | 23 | 2.67E-06 | 0.0002 |
| regulation of protein kinase activity | 698 | 8.3 | 24 | 2.68E-06 | 0.0002 |
| positive regulation of metabolic process | 2690 | 32 | 57 | 3.41E-06 | 0.000219 |
| positive regulation of cellular protein metabolic process | 968 | 11.5 | 29 | 3.47E-06 | 0.000219 |
| response to organic substance | 2500 | 29.7 | 54 | 3.80E-06 | 0.000223 |
| regulation of protein phosphorylation | 987 | 11.7 | 29 | 5.08E-06 | 0.000274 |
| neuron apoptotic process | 169 | 2.01 | 11 | 5.58E-06 | 0.000274 |
| response to external stimulus | 1510 | 18 | 38 | 5.68E-06 | 0.000274 |
| regulation of multicellular organismal process | 2480 | 29.5 | 53 | 6.74E-06 | 0.000307 |
| regulation of kinase activity | 743 | 8.84 | 24 | 7.77E-06 | 0.000335 |
| regulation of anatomical structure morphogenesis | 702 | 8.35 | 23 | 9.75E-06 | 0.0004 |
| positive regulation of protein metabolic process | 1080 | 12.9 | 30 | 1.09E-05 | 0.000426 |
| MAPK cascade | 665 | 7.91 | 22 | 1.33E-05 | 0.000481 |
| regulation of transferase activity | 768 | 9.14 | 24 | 1.35E-05 | 0.000481 |
| apoptotic DNA fragmentation | 27 | 0.321 | 5 | 1.47E-05 | 0.000502 |
| cell-matrix adhesion | 159 | 1.89 | 10 | 2.03E-05 | 0.000664 |
| regulation of phosphorylation | 1070 | 12.7 | 29 | 2.23E-05 | 0.000668 |
| Aging | 197 | 2.34 | 11 | 2.37E-05 | 0.000668 |
| negative regulation of programmed cell death | 691 | 8.22 | 22 | 2.41E-05 | 0.000668 |
| protein phosphorylation | 1480 | 17.7 | 36 | 2.41E-05 | 0.000668 |
| cell activation | 960 | 11.4 | 27 | 2.46E-05 | 0.000668 |
| regulation of protein modification process | 1250 | 14.8 | 32 | 2.53E-05 | 0.000668 |
| response to chemical stimulus | 3830 | 45.6 | 70 | 3.14E-05 | 0.000781 |
| cell-substrate adhesion | 241 | 2.87 | 12 | 3.14E-05 | 0.000781 |
| regulation of angiogenesis | 169 | 2.01 | 10 | 3.43E-05 | 0.000826 |
| negative regulation of catalytic activity | 568 | 6.76 | 19 | 4.66E-05 | 0.00109 |
| transmembrane receptor protein tyrosine kinase signaling pathway | 782 | 9.3 | 23 | 5.34E-05 | 0.00118 |
| apoptotic nuclear changes | 35 | 0.416 | 5 | 5.47E-05 | 0.00118 |
| negative regulation of apoptotic process | 679 | 8.08 | 21 | 5.69E-05 | 0.00118 |
| negative regulation of apoptotic process | 679 | 8.08 | 21 | 5.69E-05 | 0.00118 |
| regulation of body fluid levels | 680 | 8.09 | 21 | 5.81E-05 | 0.00118 |
| response to endogenous stimulus | 1360 | 16.2 | 33 | 0.000059 | 0.00118 |
| regulation of hydrolase activity | 843 | 10 | 24 | 6.06E-05 | 0.00118 |
| activation of protein kinase activity | 260 | 3.09 | 12 | 6.57E-05 | 0.00125 |
| transforming growth factor beta receptor signaling pathway | 221 | 2.63 | 11 | 6.78E-05 | 0.00126 |
| positive regulation of transcription from RNA polymerase II promoter | 800 | 9.52 | 23 | 7.55E-05 | 0.00138 |
| wound healing | 700 | 8.33 | 21 | 8.78E-05 | 0.00156 |
| positive regulation of protein modification process | 867 | 10.3 | 24 | 9.38E-05 | 0.00164 |
| positive regulation of developmental process | 817 | 9.72 | 23 | 0.000104 | 0.00177 |
| negative regulation of multicellular organismal process | 410 | 4.88 | 15 | 0.000115 | 0.00192 |
| response to extracellular stimulus | 320 | 3.81 | 13 | 0.000119 | 0.00192 |
| Note: Total, total number of molecules annotated to the pathway; Expected, expected hits; Hits, observed hits; FDR, false discovery rate. | | | | | |

| Supplementary Table S8. Cellular Component (CC) GO enrichment for gene and protein layers in the multi-omics cohort. | | | | | |
| --- | --- | --- | --- | --- | --- |
| **Pathway** | **Total** | **Expected** | **Hits** | ***P.*Value** | **FDR** |
| extracellular space | 901 | 9.46 | 28 | 2.29E-07 | 3.09E-05 |
| extracellular region part | 1320 | 13.9 | 35 | 2.74E-07 | 3.09E-05 |
| cytoplasmic vesicle part | 462 | 4.85 | 17 | 7.64E-06 | 0.000383 |
| cytosol | 2660 | 28 | 51 | 7.80E-06 | 0.000383 |
| vesicle | 1210 | 12.7 | 30 | 8.52E-06 | 0.000383 |
| membrane-bounded vesicle | 1100 | 11.5 | 27 | 3.05E-05 | 0.00101 |
| cell surface | 518 | 5.44 | 17 | 3.32E-05 | 0.00101 |
| secretory granule | 276 | 2.9 | 12 | 3.57E-05 | 0.00101 |
| cytoplasmic vesicle | 1110 | 11.6 | 26 | 9.44E-05 | 0.00236 |
| membrane-enclosed lumen | 3440 | 36.2 | 56 | 0.000272 | 0.00612 |
| cytoplasmic membrane-bounded vesicle | 1020 | 10.7 | 23 | 0.000449 | 0.00919 |
| organelle lumen | 3380 | 35.5 | 54 | 0.000599 | 0.0112 |
| extracellular region | 2860 | 30 | 45 | 0.00263 | 0.0456 |
| condensed chromosome | 193 | 2.03 | 7 | 0.00432 | 0.0694 |
| endosome | 683 | 7.17 | 15 | 0.00564 | 0.0819 |
| external side of plasma membrane | 204 | 2.14 | 7 | 0.00582 | 0.0819 |
| lamellipodium | 127 | 1.33 | 5 | 0.011 | 0.145 |
| neuron projection | 685 | 7.19 | 14 | 0.0133 | 0.167 |
| membrane raft | 189 | 1.98 | 6 | 0.0149 | 0.176 |
| growth cone | 92 | 0.966 | 4 | 0.016 | 0.177 |
| cell cortex | 195 | 2.05 | 6 | 0.0171 | 0.177 |
| extracellular matrix | 570 | 5.98 | 12 | 0.0173 | 0.177 |
| site of polarized growth | 96 | 1.01 | 4 | 0.0185 | 0.181 |
| kinetochore | 149 | 1.56 | 5 | 0.0205 | 0.193 |
| nucleoplasm | 1820 | 19.1 | 28 | 0.0254 | 0.218 |
| cytoplasmic vesicle membrane | 403 | 4.23 | 9 | 0.0267 | 0.218 |
| spliceosomal complex | 161 | 1.69 | 5 | 0.0275 | 0.218 |
| vacuolar part | 279 | 2.93 | 7 | 0.0281 | 0.218 |
| perinuclear region of cytoplasm | 475 | 4.99 | 10 | 0.0286 | 0.218 |
| coated vesicle | 283 | 2.97 | 7 | 0.03 | 0.218 |
| trans-Golgi network transport vesicle | 26 | 0.273 | 2 | 0.0302 | 0.218 |
| clathrin-coated vesicle | 224 | 2.35 | 6 | 0.031 | 0.218 |
| vesicle membrane | 417 | 4.38 | 9 | 0.0322 | 0.219 |
| nuclear body | 295 | 3.1 | 7 | 0.0364 | 0.234 |
| Golgi-associated vesicle | 70 | 0.735 | 3 | 0.0373 | 0.234 |
| late endosome | 175 | 1.84 | 5 | 0.0374 | 0.234 |
| endocytic vesicle | 187 | 1.96 | 5 | 0.0475 | 0.289 |
| Note: Total, total number of molecules annotated to the pathway; Expected, expected hits; Hits, observed hits; FDR, false discovery rate. | | | | | |

| Supplementary Table S9. Molecular Function (MF) GO enrichment for gene and protein layers in the multi-omics cohort. | | | | | |
| --- | --- | --- | --- | --- | --- |
| Pathway | Total | Expected | Hits | *P*.Value | FDR |
| enzyme inhibitor activity | 373 | 4.38 | 16 | 8.29E-06 | 0.00322 |
| heparin binding | 130 | 1.53 | 9 | 2.24E-05 | 0.00434 |
| glycosaminoglycan binding | 178 | 2.09 | 10 | 4.77E-05 | 0.00616 |
| transmembrane receptor protein tyrosine kinase activity | 88 | 1.03 | 7 | 7.76E-05 | 0.00752 |
| copper ion binding | 65 | 0.763 | 6 | 0.000112 | 0.00866 |
| transmembrane receptor protein kinase activity | 125 | 1.47 | 7 | 0.000678 | 0.0421 |
| peptidase inhibitor activity | 207 | 2.43 | 9 | 0.00076 | 0.0421 |
| protein tyrosine kinase activity | 222 | 2.61 | 9 | 0.00124 | 0.0536 |
| positive regulation of transcription, DNA-dependent | 1260 | 14.8 | 27 | 0.00155 | 0.0536 |
| kinase regulator activity | 144 | 1.69 | 7 | 0.00155 | 0.0536 |
| serine-type endopeptidase inhibitor activity | 107 | 1.26 | 6 | 0.00163 | 0.0536 |
| receptor binding | 1590 | 18.7 | 32 | 0.00166 | 0.0536 |
| enzyme regulator activity | 1140 | 13.4 | 25 | 0.0018 | 0.0536 |
| protein kinase inhibitor activity | 47 | 0.552 | 4 | 0.00221 | 0.0554 |
| protein complex binding | 339 | 3.98 | 11 | 0.00222 | 0.0554 |
| identical protein binding | 910 | 10.7 | 21 | 0.00228 | 0.0554 |
| protein domain specific binding | 560 | 6.57 | 15 | 0.0025 | 0.057 |
| kinase inhibitor activity | 51 | 0.599 | 4 | 0.00298 | 0.0643 |
| protein kinase regulator activity | 123 | 1.44 | 6 | 0.00329 | 0.0658 |
| enzyme binding | 1200 | 14.1 | 25 | 0.00344 | 0.0658 |
| growth factor binding | 125 | 1.47 | 6 | 0.00356 | 0.0658 |
| calcium ion binding | 673 | 7.9 | 16 | 0.00576 | 0.102 |
| structure-specific DNA binding | 242 | 2.84 | 8 | 0.00782 | 0.132 |
| double-stranded DNA binding | 149 | 1.75 | 6 | 0.00828 | 0.134 |
| insulin-like growth factor receptor binding | 14 | 0.164 | 2 | 0.0114 | 0.176 |
| MAP kinase activity | 16 | 0.188 | 2 | 0.0148 | 0.22 |
| transcription from RNA polymerase II promoter | 1930 | 22.6 | 33 | 0.0159 | 0.228 |
| chaperone binding | 46 | 0.54 | 3 | 0.0167 | 0.231 |
| integrin binding | 86 | 1.01 | 4 | 0.0185 | 0.24 |
| transmembrane receptor protein phosphatase activity | 18 | 0.211 | 2 | 0.0185 | 0.24 |
| structural constituent of muscle | 49 | 0.575 | 3 | 0.0197 | 0.247 |
| receptor signaling protein serine/threonine kinase activity | 52 | 0.61 | 3 | 0.0231 | 0.271 |
| SH3/SH2 adaptor activity | 52 | 0.61 | 3 | 0.0231 | 0.271 |
| ligase activity, forming carbon-oxygen bonds | 53 | 0.622 | 3 | 0.0243 | 0.274 |
| protein dimerization activity | 996 | 11.7 | 19 | 0.0247 | 0.274 |
| ion binding | 6140 | 72.1 | 85 | 0.0272 | 0.293 |
| binding, bridging | 148 | 1.74 | 5 | 0.0304 | 0.319 |
| protein heterodimerization activity | 374 | 4.39 | 9 | 0.0325 | 0.332 |
| protein homodimerization activity | 573 | 6.73 | 12 | 0.0378 | 0.376 |
| negative regulation of transcription, DNA-dependent | 987 | 11.6 | 18 | 0.0415 | 0.403 |
| SMAD binding | 68 | 0.798 | 3 | 0.0458 | 0.43 |
| microtubule binding | 115 | 1.35 | 4 | 0.0465 | 0.43 |
| phosphatase binding | 116 | 1.36 | 4 | 0.0478 | 0.431 |
| Note: Total, total number of molecules annotated to the pathway; Expected, expected hits; Hits, observed hits; FDR, false discovery rate. | | | | | |

| Supplementary Table S10. Sensitivity analyses by first-episode status | | | | | |
| --- | --- | --- | --- | --- | --- |
| **LASSO Metabolites** | **coefficients** | **Differentially expressed metabolites** | **T** | **dof** | **P-value** |
| **First Episode Group (n=57)** | | | | | |
| TG(52:5-16:0) | 1.001 | D-Glucose | -2.157 | 54.811 | 0.035 |
| Glycoursodeoxycholic acid | -0.767 | Propionic acid | -2.081 | 46.974 | 0.043 |
| DG(18:2/20:4) | -0.748 | Glycoursodeoxycholic acid | -2.442 | 40.181 | 0.019 |
| N6-Acetyl-L-lysine | -0.495 | Indole-3-acrylic acid | -2.245 | 44.854 | 0.030 |
| Carnosine | -0.458 | Imidazolepropionic acid | -2.175 | 53.803 | 0.034 |
| Creatinine | 0.427 | Ergothioneine | -2.032 | 53.134 | 0.047 |
| PC(32:2) | 0.425 | N-Acetylvaline | 2.129 | 55.000 | 0.038 |
| Hydroxyhexadecanoylcarnitine (AcCa(16:0-OH)) | 0.422 | Hydroxyhexadecanoylcarnitine (AcCa(16:0-OH)) | 2.497 | 54.022 | 0.016 |
| Glutaric acid | -0.409 | Leucyl-leucine | 2.066 | 48.581 | 0.044 |
| PE-O(36:1) | 0.402 | 3-Hydroxymyristic acid | 2.183 | 50.873 | 0.034 |
| Ursodeoxycholic acid | 0.379 | PC(32:2) | 2.073 | 54.114 | 0.043 |
| Propionic acid | -0.361 | PE(p16:0/18:2) | -2.052 | 49.647 | 0.045 |
| 3-Hydroxymyristic acid | 0.326 | PE(p18:1/20:5) | 2.282 | 53.669 | 0.026 |
| PE-O(38:5) | -0.324 | PE-O(36:1) | 2.253 | 53.329 | 0.028 |
| 3-(3-Hydroxyphenyl)-3-hydroxypropanoic acid | -0.319 | PE-O(36:5) | -2.625 | 49.556 | 0.011 |
| PA(18:0/20:4) | 0.300 | TG(52:5-16:0) | 2.894 | 51.570 | 0.006 |
| Indole-3-acrylic acid | -0.276 | TG(53:2-16:0) | 2.111 | 53.454 | 0.039 |
| SM(40:2) | 0.260 |  |  |  |  |
| TG(46:2-16:0) | 0.214 |  |  |  |  |
| cis-5-Tetradecenoic acid | -0.191 |  |  |  |  |
| **Recurrent Group (n=151)** | | | | | |
| Pimelic acid | -0.197 | Glycolate | -2.339 | 138.211 | 0.021 |
| PC(34:2) | 0.193 | Propionic acid | -2.060 | 148.902 | 0.041 |
| 3beta-Hydroxy-5-cholenoic acid | 0.183 | 3beta-Hydroxy-5-cholenoic acid | 2.162 | 146.784 | 0.032 |
| TG(54:4-16:1) | 0.178 | 3-Hydroxysebacic acid | -2.005 | 140.414 | 0.047 |
| Omega-muricholic acid | -0.162 | 2-Hydroxy-3-methylbutyric acid | 2.190 | 144.432 | 0.030 |
| PC(28:0) | 0.144 | 4-hydroxyphenyllactate | 2.069 | 148.678 | 0.040 |
| TG(54:6-16:0) | 0.143 | Stearic acid | -2.132 | 130.562 | 0.035 |
| PE-O(36:1) | -0.128 | Citrulline | -2.100 | 131.001 | 0.038 |
| PE(p18:0/20:5) | -0.108 | Hydroxy-hexadecenoylcarnitine (AcCa(16:1-OH)) | -2.340 | 148.008 | 0.021 |
| TG(50:1-14:0) | -0.091 | PC(28:0) | 2.642 | 147.718 | 0.009 |
| PC(30:0) | 0.091 | PC(30:0) | 3.045 | 148.704 | 0.003 |
| Stearic acid | -0.085 | PC(34:2) | 2.387 | 136.211 | 0.018 |
| 2-Hydroxy Hippuric Acid | 0.079 | PC(36:2) | 2.119 | 141.990 | 0.036 |
| 3-Epideoxycholic acid | 0.075 | PC(37:5) | 2.549 | 141.485 | 0.012 |
| 4-hydroxyphenyllactate | 0.075 | PC(42:8) | 2.352 | 142.849 | 0.020 |
| 3-Hydroxyanthranilic acid | 0.066 | PC-O(36:3) | 2.063 | 142.673 | 0.041 |
| Glycolate | -0.059 | PE(30:1) | 2.892 | 143.684 | 0.004 |
| PE(30:1) | 0.057 | PE(35:2) | 2.034 | 147.262 | 0.044 |
| Hydroxy-hexadecenoylcarnitine (AcCa(16:1-OH)) | -0.055 | PE(36:3) | 2.457 | 138.612 | 0.015 |
| 2-Hydroxy-3-methylbutyric acid | 0.054 | PE(38:3) | 2.374 | 141.129 | 0.019 |
|  |  | PE(38:6) | 2.207 | 137.715 | 0.029 |
|  |  | PE-O(36:1) | -1.997 | 131.975 | 0.048 |
|  |  | TG(52:5-16:0) | 2.075 | 141.177 | 0.040 |
|  |  | TG(54:4-16:1) | 3.054 | 148.307 | 0.003 |
|  |  | TG(54:5-18:2) | 2.313 | 137.338 | 0.022 |
|  |  | TG(54:6-16:0) | 2.761 | 141.833 | 0.007 |
|  |  | PG(16:0/16:0) | 1.985 | 126.719 | 0.049 |
|  |  | PS(18:0/20:0) | -2.496 | 148.991 | 0.014 |
| dof, degrees of freedom; Note: All listed metabolites have non-zero LASSO coefficients. Features are ranked by absolute coefficient values. T-values are reported with treatment responder group as the reference group. | | | | | |

| Supplementary Table S11. Plasma Metabolites polygenic risk scores (PRS) between treatment responders and non-responders in the plasma PRS validation dataset. | | | | |
| --- | --- | --- | --- | --- |
| **Metabolites** | **T** | **dof** | ***P*.Value** | **95% CI** |
| **TGA000003 (base data)** |  |  |  |  |
| **Glutamylphenylalanine** | **-2.435** | **1699.77** | **0.015** | **[-0.02, 0]** |
| **Ethylmalonic acid** | **-2.356** | **1642.24** | **0.019** | **[-0.03, 0]** |
| **8-Hydroxy-5,6-octadienoic acid \|\| 5-oxo-7-octe...** | **-2.347** | **1788.02** | **0.019** | **[-0.03, 0]** |
| **Uric acid** | **-2.292** | **1722.1** | **0.022** | **[-0.03, 0]** |
| **PC(16:1(9Z)/0:0)** | **2.119** | **1774.55** | **0.034** | **[0, 0.02]** |
| **Proline** | **-2.003** | **1774.79** | **0.045** | **[-0.02, 0]** |
| L-Palmitoylcarnitine | 1.902 | 1695.35 | 0.057 | [0, 0.02] |
| PC(40:6) | -1.826 | 1790.69 | 0.068 | [-0.03, 0] |
| Acetylglycine | -1.727 | 1699.13 | 0.084 | [-0.02, 0] |
| Indoleacrylic acid | 1.509 | 1687.11 | 0.131 | [0, 0.03] |
| PC(0:0/20:4(5Z,8Z,11Z,14Z)) | 1.477 | 1800.6 | 0.140 | [0, 0.02] |
| Orotic acid | 1.391 | 1707.24 | 0.164 | [0, 0.02] |
| Ribonic acid | 1.154 | 1739.27 | 0.249 | [-0.01, 0.03] |
| 3-Dehydroxycarnitine \|\| 2-amino-heptanoic acid | 1.101 | 1753.1 | 0.271 | [-0.01, 0.02] |
| L-Kynurenine | 1.096 | 1738.8 | 0.273 | [-0.01, 0.02] |
| LysoPE(20:4(5Z,8Z,11Z,14Z)/0:0) | 1.094 | 1799.82 | 0.274 | [-0.01, 0.03] |
| Asparagine | -1.084 | 1741.64 | 0.278 | [-0.02, 0.01] |
| PC(16:0/0:0) | 1.033 | 1702.65 | 0.302 | [-0.01, 0.02] |
| PE(18:2(9Z,12Z)/0:0) | 0.945 | 1768.19 | 0.345 | [-0.01, 0.02] |
| PC(42:8) | 0.846 | 1798.89 | 0.398 | [-0.01, 0.02] |
| Phenylalanyl-Valine \|\| Valyl-Phenylalanine | 0.786 | 1743.62 | 0.432 | [-0.01, 0.02] |
| LysoPE(18:0/0:0) | 0.741 | 1752.36 | 0.459 | [-0.01, 0.01] |
| Oleoylcarnitine | -0.741 | 1659.12 | 0.459 | [-0.02, 0.01] |
| Biliverdin | -0.724 | 1769.94 | 0.469 | [-0.02, 0.01] |
| Serine | 0.691 | 1728.57 | 0.490 | [-0.01, 0.02] |
| PC(42:6) | -0.680 | 1749.21 | 0.496 | [-0.02, 0.01] |
| Tryptophan | 0.669 | 1723.1 | 0.503 | [-0.01, 0.02] |
| Formate | 0.641 | 1737.94 | 0.522 | [-0.01, 0.02] |
| PC(0:0/16:0) | 0.618 | 1714.36 | 0.536 | [-0.01, 0.02] |
| PC(20:4(5Z,8Z,11Z,14Z)/0:0) | 0.467 | 1786.31 | 0.641 | [-0.01, 0.02] |
| 3,4,5-Trimethoxycinnamic acid \|\| Trans-2, 3, 4... | 0.414 | 1688.58 | 0.679 | [-0.01, 0.02] |
| PE(16:0/0:0) | 0.370 | 1772.6 | 0.711 | [-0.01, 0.02] |
| Glycine | -0.353 | 1785.79 | 0.724 | [-0.01, 0.01] |
| L-Octanoylcarnitine | -0.335 | 1666.64 | 0.737 | [-0.01, 0.01] |
| Homo-L-arginine | 0.306 | 1701.13 | 0.760 | [-0.01, 0.02] |
| PI(38:4) | 0.306 | 1720.65 | 0.760 | [-0.01, 0.02] |
| NeuAcalpha2-3Galbeta-Cer(d18:1/16:0) | 0.279 | 1722.82 | 0.780 | [-0.01, 0.02] |
| PA(20:3(8Z,11Z,14Z)/0:0)_Na+ | 0.232 | 1798.33 | 0.816 | [-0.01, 0.01] |
| SM d18:1/18:1(9Z) | 0.178 | 1667.28 | 0.859 | [-0.01, 0.01] |
| Indolepropionate | -0.150 | 1776.71 | 0.881 | [-0.01, 0.01] |
| Glutaric acid | -0.070 | 1737.02 | 0.944 | [-0.01, 0.01] |
| PE(0:0/16:0) | 0.067 | 1775.06 | 0.946 | [-0.01, 0.01] |
| Phenylalanine | -0.015 | 1774.14 | 0.988 | [-0.01, 0.01] |
| **TGA000005 (base data)** |  |  |  |  |
| **cis-Aconitic acid** | **2.542** | **1715.917** | **0.011** | **[0.00, 0.03]** |
| **4-Pyridoxic acid** | **-2.512** | **1748.688** | **0.012** | **[-0.03, 0.00]** |
| **Erythritol** | **2.420** | **1750.124** | **0.016** | **[0.00, 0.02]** |
| **2-Hydroxyglutarate** | **2.358** | **1811.828** | **0.018** | **[0.00, 0.03]** |
| **PC P-38:3** | **2.348** | **1796.399** | **0.019** | **[0.00, 0.03]** |
| **Glycerol** | **-2.338** | **1748.765** | **0.019** | **[-0.03, 0.00]** |
| **LPC 20:1(11Z)** | **2.245** | **1816.339** | **0.025** | **[0.00, 0.03]** |
| **SM d18:1/22:2(13Z,16Z)** | **2.230** | **1726.076** | **0.026** | **[0.00, 0.02]** |
| **LPC 18:1(9Z)** | **2.215** | **1714.104** | **0.027** | **[0.00, 0.02]** |
| **Deoxycholic acid glycine conjugate** | **-2.153** | **1793.697** | **0.031** | **[-0.02, 0.00]** |
| **Carbamoyl phosphate** | **-2.040** | **1762.713** | **0.042** | **[-0.03, 0.00]** |
| **Lysine** | **2.028** | **1702.284** | **0.043** | **[0.00, 0.02]** |
| 2-Hydroxybutyric acid \|\| 3-Hydroxybutyric acid \|\| 4-Hydroxybutyric acid | 1.932 | 1762.592 | 0.053 | [0.00, 0.02] |
| L-Alanine | 1.841 | 1733.541 | 0.066 | [0.00, 0.02] |
| Histidine | 1.801 | 1766.688 | 0.072 | [0.00, 0.02] |
| PE 16:0/22:6(4Z,7Z,10Z,13Z,16Z,19Z) | -1.794 | 1740.527 | 0.073 | [-0.03, 0.00] |
| Caffeine | 1.774 | 1742.841 | 0.076 | [0.00, 0.03] |
| Fumaric acid | -1.767 | 1742.261 | 0.077 | [-0.02, 0.00] |
| Taurodeoxycholic acid | 1.671 | 1715.367 | 0.095 | [0.00, 0.02] |
| Citric acid | 1.668 | 1731.045 | 0.095 | [0.00, 0.02] |
| Hydroxyphenyllactic acid | 1.667 | 1773.306 | 0.096 | [0.00, 0.03] |
| Indoxyl sulfate | -1.662 | 1684.622 | 0.097 | [-0.03, 0.00] |
| Acetate | 1.647 | 1813.266 | 0.100 | [0.00, 0.02] |
| D-Arabitol | -1.634 | 1758.242 | 0.102 | [-0.02, 0.00] |
| D-Glucose | -1.633 | 1768.382 | 0.103 | [-0.02, 0.00] |
| Gluconic acid | 1.621 | 1706.411 | 0.105 | [0.00, 0.02] |
| Arginine | 1.620 | 1731.634 | 0.106 | [0.00, 0.02] |
| Tryptophan | 1.604 | 1740.624 | 0.109 | [0.00, 0.02] |
| (S)-3,4-Dihydroxybutyric acid | -1.568 | 1751.928 | 0.117 | [-0.02, 0.00] |
| PI 34:2 | 1.565 | 1739.887 | 0.118 | [0.00, 0.02] |
| Lactic acid | -1.547 | 1738.47 | 0.122 | [-0.02, 0.00] |
| PC 16:0/18:3(9Z,12Z,15Z) | 1.537 | 1704.606 | 0.125 | [0.00, 0.03] |
| Alpha-Linolenic acid | 1.521 | 1800.73 | 0.128 | [0.00, 0.02] |
| 2-Hydroxyvaleric acid \|\| 3-Hydroxyvaleric acid | 1.503 | 1701.935 | 0.133 | [0.00, 0.02] |
| 2-Hydroxybutyric acid \|\| 3-Hydroxybutyric acid \|\| 4-Hydroxybutyric acid | -1.489 | 1729.201 | 0.137 | [-0.02, 0.00] |
| LPE 16:0 | -1.474 | 1765.792 | 0.141 | [-0.02, 0.00] |
| Acetone | 1.400 | 1718.26 | 0.162 | [0.00, 0.02] |
| Kynurenic acid | 1.397 | 1724.149 | 0.162 | [0.00, 0.02] |
| Pyridoxal | -1.389 | 1789.636 | 0.165 | [-0.02, 0.00] |
| 6-Oxopiperidine-2-carboxylic acid | -1.346 | 1677.511 | 0.178 | [-0.02, 0.00] |
| PC 16:0/18:2(9Z,12Z) | 1.345 | 1779.961 | 0.179 | [0.00, 0.02] |
| cis-Glutaconic acid | -1.344 | 1766.737 | 0.179 | [-0.02, 0.00] |
| L-Pipecolic acid | 1.328 | 1776.672 | 0.184 | [0.00, 0.02] |
| 9-Oxooctadecanoic acid | 1.326 | 1720.633 | 0.185 | [0.00, 0.02] |
| 3-Hydroxyoctanoic acid | -1.321 | 1659.326 | 0.187 | [-0.02, 0.00] |
| Aspartyl-Asparagine | 1.316 | 1690.625 | 0.188 | [0.00, 0.02] |
| Glycine | -1.299 | 1693.473 | 0.194 | [-0.02, 0.00] |
| Uridine | 1.286 | 1756.227 | 0.199 | [0.00, 0.02] |
| Inosine 2,3-cyclic phosphate | 1.280 | 1699.024 | 0.201 | [0.00, 0.02] |
| Glucose | 1.269 | 1773.019 | 0.205 | [0.00, 0.02] |
| L-Arginine | -1.251 | 1675.796 | 0.211 | [-0.02, 0.00] |
| Arginyl-Gamma-glutamate | 1.234 | 1661.562 | 0.217 | [0.00, 0.02] |
| LPA 18:1(9Z) | 1.182 | 1742.116 | 0.237 | [0.00, 0.02] |
| MG(14:0/0:0/0:0) | 1.166 | 1759.365 | 0.244 | [-0.01, 0.02] |
| PA 36:3 | -1.157 | 1770.717 | 0.247 | [-0.02, 0.01] |
| Inosine 2,3-cyclic phosphate | 1.156 | 1747.607 | 0.248 | [-0.01, 0.02] |
| Arachidonic acid | 1.146 | 1732.439 | 0.252 | [-0.01, 0.02] |
| LPE 20:4(5Z,8Z,11Z,14Z) | -1.136 | 1717.464 | 0.256 | [-0.02, 0.01] |
| LPE 20:1(11Z) | -1.135 | 1836.868 | 0.257 | [-0.02, 0.01] |
| Lactate | -1.126 | 1686.237 | 0.260 | [-0.02, 0.00] |
| Phenyl sulfate | -1.110 | 1808.222 | 0.267 | [-0.02, 0.01] |
| (9Z,11E)-13-HODE | -1.099 | 1669.833 | 0.272 | [-0.02, 0.01] |
| Dicalcium phosphate | -1.080 | 1749.761 | 0.280 | [-0.01, 0.00] |
| Caffeic acid | 1.076 | 1757.207 | 0.282 | [-0.01, 0.02] |
| SM d18:1/22:1(13Z) | 1.054 | 1706.833 | 0.292 | [-0.01, 0.02] |
| PE P-36:4 | 1.048 | 1683.089 | 0.295 | [-0.01, 0.02] |
| PI 18:0/20:4(5Z,8Z,11Z,14Z) | 1.025 | 1685.781 | 0.305 | [-0.01, 0.02] |
| Methionine | 1.025 | 1661.622 | 0.306 | [-0.01, 0.02] |
| PC 36:4 | -1.024 | 1732.371 | 0.306 | [-0.02, 0.01] |
| Inosine 2,3-cyclic phosphate | 1.021 | 1746.826 | 0.307 | [-0.01, 0.02] |
| PE P-38:5 | 1.005 | 1703.293 | 0.315 | [-0.01, 0.02] |
| SM C16:1 | 1.003 | 1686.744 | 0.316 | [-0.01, 0.02] |
| Indolelactic acid | 0.996 | 1738.687 | 0.319 | [-0.01, 0.02] |
| Indoleacetic acid | -0.993 | 1769.216 | 0.321 | [-0.02, 0.01] |
| 11-Eicosenoic acid | 0.984 | 1761.036 | 0.325 | [-0.01, 0.02] |
| Eicosapentaenoic acid | -0.940 | 1668.583 | 0.347 | [-0.01, 0.01] |
| L-Valine | -0.922 | 1734.249 | 0.357 | [-0.02, 0.01] |
| Oxalic acid | -0.921 | 1793.342 | 0.357 | [-0.02, 0.01] |
| Citrulline | -0.898 | 1642.562 | 0.369 | [-0.02, 0.01] |
| Pyruvate | 0.896 | 1750.665 | 0.370 | [-0.01, 0.02] |
| Proline | 0.883 | 1841.561 | 0.377 | [-0.01, 0.02] |
| Sulfuric acid | 0.877 | 1728.573 | 0.380 | [-0.01, 0.02] |
| L-Lysine | 0.853 | 1757.623 | 0.394 | [-0.01, 0.02] |
| O-methoxycatechol-O-sulphate | 0.846 | 1752.314 | 0.398 | [-0.01, 0.02] |
| 2-Hydroxybutyric acid \|\| 3-Hydroxybutyric acid \|\| 4-Hydroxybutyric acid | 0.832 | 1709.058 | 0.405 | [-0.01, 0.02] |
| Betaine | 0.820 | 1786.656 | 0.412 | [-0.01, 0.02] |
| Glutamyl-Gamma-glutamate | -0.815 | 1700.668 | 0.415 | [-0.02, 0.01] |
| Deoxycholic acid | 0.810 | 1730.558 | 0.418 | [-0.01, 0.02] |
| 9-hydroxyoctadecanoic acid | -0.809 | 1790.338 | 0.419 | [-0.01, 0.01] |
| LPC 20:2(11Z,14Z) | 0.808 | 1688.047 | 0.419 | [-0.01, 0.02] |
| L-Threonine | 0.804 | 1633.501 | 0.421 | [-0.01, 0.02] |
| Glycerol | 0.796 | 1758.893 | 0.426 | [-0.01, 0.02] |
| 2-Hydroxyadipic acid | 0.794 | 1819.758 | 0.427 | [-0.01, 0.01] |
| PE 40:3 | -0.791 | 1781.054 | 0.429 | [-0.02, 0.01] |
| PG 18:0/18:0 | 0.787 | 1762.262 | 0.432 | [-0.01, 0.02] |
| L-Asparagine | 0.785 | 1635.427 | 0.433 | [-0.01, 0.02] |
| Phosphoric acid | 0.784 | 1752.354 | 0.433 | [-0.01, 0.02] |
| LPE 18:2(9Z,12Z) | 0.773 | 1748.753 | 0.440 | [-0.01, 0.02] |
| Adenine | -0.766 | 1672.651 | 0.444 | [-0.02, 0.01] |
| 3-Hydroxyisobutyrate | -0.766 | 1729.298 | 0.444 | [-0.02, 0.01] |
| L-Leucine \|\| Isoleucine | 0.764 | 1671.403 | 0.445 | [-0.01, 0.02] |
| a-Ketoglutarate | 0.761 | 1829.01 | 0.446 | [-0.01, 0.02] |
| Glyceric acid | -0.756 | 1701.106 | 0.450 | [-0.01, 0.01] |
| PC 18:1(9Z)/20:4(5Z,8Z,11Z,14Z) | 0.752 | 1757.892 | 0.452 | [-0.01, 0.02] |
| LPC 16:1(9Z) | -0.742 | 1785.006 | 0.458 | [-0.02, 0.01] |
| Docosapentaenoic acid (22n-6) | 0.738 | 1782.195 | 0.460 | [-0.01, 0.02] |
| Glutaric acid | 0.738 | 1812.826 | 0.461 | [-0.01, 0.02] |
| 2-Ketobutyric acid | 0.737 | 1729.599 | 0.461 | [-0.01, 0.02] |
| gamma-Aminobutyric acid | -0.735 | 1785.266 | 0.462 | [-0.02, 0.01] |
| LPA 18:2(9Z,12Z) | 0.719 | 1723.734 | 0.473 | [-0.01, 0.02] |
| 3-Methyl-2-Oxobutyric Acid | 0.710 | 1813.435 | 0.478 | [-0.01, 0.02] |
| LPC P-18:1(9Z) | 0.709 | 1834.419 | 0.478 | [-0.01, 0.02] |
| CerP d18:1/16:0 | 0.703 | 1747.911 | 0.482 | [-0.01, 0.01] |
| 3-Hydroxybenzoic acid | -0.700 | 1699.578 | 0.484 | [-0.02, 0.01] |
| Pyridoxal (phosphate or sulfate) | 0.685 | 1688.95 | 0.493 | [-0.01, 0.02] |
| N-Phenylacetylglutamine | 0.682 | 1755.813 | 0.495 | [-0.01, 0.02] |
| L-Glutamine | 0.682 | 1713.595 | 0.495 | [-0.01, 0.02] |
| PC(18:0/22:5(4Z,7Z,10Z,13Z,16Z)) | 0.677 | 1765.038 | 0.498 | [-0.01, 0.02] |
| LPE 18:0 | -0.674 | 1674.538 | 0.500 | [-0.02, 0.01] |
| Ornithine | -0.670 | 1684.954 | 0.503 | [-0.02, 0.01] |
| 2-Aminobutyrate | 0.642 | 1701.53 | 0.521 | [-0.01, 0.02] |
| 2-Hydroxybutyrate | -0.640 | 1713.165 | 0.522 | [-0.02, 0.01] |
| Pseudouridine | -0.613 | 1729.32 | 0.540 | [-0.02, 0.01] |
| Carnitine | 0.610 | 1638.633 | 0.542 | [-0.01, 0.02] |
| Creatine | -0.607 | 1717.572 | 0.544 | [-0.02, 0.01] |
| beta-D-Glucosamine | -0.579 | 1755.766 | 0.562 | [-0.02, 0.01] |
| PC 18:1(9Z)/22:6(4Z,7Z,10Z,13Z,16Z,19Z) | -0.578 | 1834.265 | 0.563 | [-0.01, 0.01] |
| N-Acetyl-L-alanine | 0.576 | 1795.413 | 0.565 | [-0.01, 0.02] |
| Myristic acid | 0.575 | 1696.194 | 0.566 | [-0.01, 0.01] |
| Asparagine | -0.569 | 1678.11 | 0.569 | [-0.02, 0.01] |
| LPC 18:2(9Z,12Z) | -0.567 | 1634.802 | 0.571 | [-0.01, 0.01] |
| Isoleucine | 0.564 | 1704.239 | 0.573 | [-0.01, 0.01] |
| 2-Amino-3-phosphonopropionic acid | 0.559 | 1776.761 | 0.576 | [-0.01, 0.02] |
| 3-Methyl-2-Oxovalerate | 0.557 | 1690.829 | 0.577 | [-0.01, 0.02] |
| Creatine | 0.548 | 1728.545 | 0.584 | [-0.01, 0.01] |
| Pyroglutamic acid | 0.542 | 1663.847 | 0.588 | [-0.01, 0.02] |
| L-Cystine | -0.538 | 1769.66 | 0.591 | [-0.02, 0.01] |
| L-Tyrosine | -0.534 | 1655.485 | 0.594 | [-0.02, 0.01] |
| p-Cresol sulfate | -0.523 | 1687.017 | 0.601 | [-0.01, 0.01] |
| 5,6-Dihydrouridine | -0.519 | 1738.696 | 0.604 | [-0.01, 0.01] |
| Malic acid | 0.509 | 1708.086 | 0.611 | [-0.01, 0.02] |
| LysoSM(d18:0) | -0.498 | 1650.612 | 0.618 | [-0.02, 0.01] |
| N-Acetyl-S-(N-methylcarbamoyl)cysteine | -0.491 | 1713.515 | 0.624 | [-0.02, 0.01] |
| N,N-Dimethylglycine | 0.483 | 1726.519 | 0.629 | [-0.01, 0.02] |
| TG(8:0/8:0/13:0) | 0.481 | 1712.448 | 0.631 | [-0.01, 0.02] |
| 2-Oxovaleric acid | 0.472 | 1704.696 | 0.637 | [-0.01, 0.01] |
| D-Ribose | -0.462 | 1780.398 | 0.644 | [-0.02, 0.01] |
| Citrate | 0.458 | 1701.809 | 0.647 | [-0.01, 0.01] |
| N1-Methyl-4-pyridone-3-carboxamide | -0.433 | 1722.719 | 0.665 | [-0.01, 0.01] |
| Aspartyl-Cysteine | -0.422 | 1671.405 | 0.673 | [-0.01, 0.01] |
| LPE 22:6(4Z,7Z,10Z,13Z,16Z,19Z) | -0.421 | 1854.509 | 0.674 | [-0.01, 0.01] |
| L-Proline | -0.408 | 1716.786 | 0.683 | [-0.01, 0.01] |
| Uric acid | 0.401 | 1766.978 | 0.688 | [-0.01, 0.01] |
| D-Fucose | 0.397 | 1742.414 | 0.692 | [-0.01, 0.02] |
| SM d18:1/18:1(9Z) | 0.386 | 1671.784 | 0.700 | [-0.01, 0.02] |
| D-Ribose | -0.380 | 1789.086 | 0.704 | [-0.01, 0.01] |
| PI 16:0/20:4(5Z,8Z,11Z,14Z) | 0.370 | 1689.749 | 0.712 | [-0.01, 0.02] |
| Glutamate | -0.364 | 1744.493 | 0.716 | [-0.01, 0.01] |
| Stearic acid | 0.352 | 1728.265 | 0.725 | [-0.01, 0.01] |
| LPC 22:6(4Z,7Z,10Z,13Z,16Z,19Z) | 0.338 | 1763.423 | 0.736 | [-0.01, 0.01] |
| PE 18:0/20:4(5Z,8Z,11Z,14Z) | 0.316 | 1685.214 | 0.752 | [-0.01, 0.01] |
| Phenylalanine | -0.294 | 1701.135 | 0.769 | [-0.02, 0.01] |
| L-Tryptophan | 0.293 | 1752.893 | 0.769 | [-0.01, 0.02] |
| PC 38:3 | 0.290 | 1733.863 | 0.772 | [-0.01, 0.02] |
| Leucine | 0.289 | 1605.899 | 0.773 | [-0.01, 0.02] |
| Valine | 0.276 | 1683.768 | 0.783 | [-0.01, 0.01] |
| Pantothenic acid | -0.274 | 1760.084 | 0.784 | [-0.01, 0.01] |
| Succinate | 0.269 | 1843.163 | 0.788 | [-0.01, 0.01] |
| Creatinine | -0.266 | 1651.628 | 0.790 | [-0.01, 0.01] |
| LPC 16:0 | -0.251 | 1712.953 | 0.802 | [-0.01, 0.01] |
| Threonine | -0.247 | 1705.215 | 0.805 | [-0.01, 0.01] |
| LPA 16:0 | 0.235 | 1790.554 | 0.814 | [-0.01, 0.01] |
| 11b-Hydroxyprogesterone | 0.227 | 1721.554 | 0.820 | [-0.01, 0.02] |
| Ornithine | 0.212 | 1763.754 | 0.832 | [-0.01, 0.01] |
| 2-Oxoisocaproate | -0.208 | 1772.211 | 0.835 | [-0.01, 0.01] |
| L-Phenylalanine | -0.204 | 1706.913 | 0.839 | [-0.01, 0.01] |
| Uridine | 0.197 | 1770.364 | 0.844 | [-0.01, 0.01] |
| 2,4,6-Trihydroxybenzoic acid | -0.193 | 1810.733 | 0.847 | [-0.01, 0.01] |
| Glutamine | -0.183 | 1663.586 | 0.855 | [-0.01, 0.01] |
| 9,10-DiHODE | -0.169 | 1791.012 | 0.866 | [-0.01, 0.01] |
| L-Glutamic acid | 0.167 | 1701.998 | 0.867 | [-0.01, 0.01] |
| Arachidic acid | 0.153 | 1711.423 | 0.878 | [-0.01, 0.01] |
| LPC 22:1(13Z) | -0.150 | 1777.024 | 0.881 | [-0.01, 0.01] |
| Creatinine | 0.144 | 1695.938 | 0.885 | [-0.01, 0.01] |
| 5-Hydroxyisourate | 0.138 | 1763.197 | 0.890 | [-0.01, 0.01] |
| PC 36:3 | -0.137 | 1759.341 | 0.891 | [-0.01, 0.01] |
| Alanine | 0.122 | 1661.764 | 0.903 | [-0.01, 0.01] |
| LPA 18:0 | 0.122 | 1751.499 | 0.903 | [-0.01, 0.01] |
| SM d18:1/20:1(11Z,14Z) | 0.099 | 1737.429 | 0.921 | [-0.01, 0.01] |
| TG(18:3(9Z,12Z,15Z)/22:6(4Z,7Z,10Z,13Z,16Z,19Z)/20:4(8Z,11Z,14Z,17Z)) | 0.093 | 1666.276 | 0.926 | [-0.01, 0.01] |
| Taurine | 0.092 | 1789.039 | 0.926 | [-0.01, 0.01] |
| PC P-36:3 | -0.091 | 1782.53 | 0.928 | [-0.01, 0.01] |
| Formate | -0.088 | 1654.764 | 0.930 | [-0.02, 0.01] |
| trans-Glutaconic acid | -0.088 | 1761.924 | 0.930 | [-0.01, 0.01] |
| Threonic acid | -0.088 | 1725.761 | 0.930 | [-0.01, 0.01] |
| Uracil | -0.081 | 1696.334 | 0.935 | [-0.01, 0.01] |
| PI 18:1(9Z)/18:1(9Z) | -0.054 | 1703.76 | 0.957 | [-0.01, 0.01] |
| Tyrosine | 0.052 | 1746.248 | 0.959 | [-0.02, 0.02] |
| Docosahexaenoic acid | -0.049 | 1754.038 | 0.961 | [-0.01, 0.01] |
| Serine | -0.046 | 1676.647 | 0.963 | [-0.01, 0.01] |
| PC 20:1(11Z)/22:6(4Z,7Z,10Z,13Z,16Z,19Z) | -0.036 | 1681.491 | 0.971 | [-0.01, 0.01] |
| LPC 20:4(5Z,8Z,11Z,14Z) | -0.034 | 1670.324 | 0.973 | [-0.01, 0.01] |
| 3-Hydroxybutyrate | -0.031 | 1744.62 | 0.975 | [-0.01, 0.01] |
| Glycylglycylglycine | -0.031 | 1669 | 0.975 | [-0.01, 0.01] |
| Cysteine | -0.008 | 1738.581 | 0.994 | [-0.01, 0.01] |
| 2-Hydroxyvaleric acid \|\| 3-Hydroxyvaleric acid | 0.006 | 1677.408 | 0.995 | [-0.01, 0.01] |
| dof, degrees of freedom; 95% CI, 95% confidence interval.  Note: Significant results are indicated in bold. Phospholipid metabolites with significant differences are highlighted in red. T-values are reported with treatment responder group as the reference group. | | | | |

| Supplementary Table S12. Brain Metabolites polygenic risk scores (PRS) between treatment responders and non-responders in the brain PRS validation cohort | | | | |
| --- | --- | --- | --- | --- |
| **Metabolites** | **T** | **dof** | ***P*.Value** | **95% CI** |
| **Phosphatidylcholine (PC base data)** |  |  |  |  |
| CSF_1,2-dipalmitoyl-GPC (16:0/16:0) | -0.57735912 | 82.2106243 | 0.56527474 | [-0.06, 0.03] |
| CSF_1-myristoyl-2-palmitoyl-GPC (14:0/16:0) | -1.04865278 | 87.6542288 | 0.29722079 | [-0.06, 0.02] |
| CSF_1-palmitoyl-2-arachidonoyl-GPC (16:0/20:4n6) | -0.16029806 | 84.3631252 | 0.87302949 | [-0.05, 0.04] |
| CSF_1-palmitoyl-2-dihomo-linolenoyl-GPC (16:0/20:3n3 or 6) | -0.45709591 | 82.0733634 | 0.64880971 | [-0.07, 0.04] |
| CSF_1-palmitoyl-2-docosahexaenoyl-GPC (16:0/22:6) | 0.2938825 | 92.4434999 | 0.76950655 | [-0.03, 0.03] |
| CSF_1-palmitoyl-2-linoleoyl-GPC (16:0/18:2) | -0.48334145 | 87.9906303 | 0.63005389 | [-0.03, 0.02] |
| CSF_1-palmitoyl-2-oleoyl-GPC (16:0/18:1) | -0.39570958 | 83.0545196 | 0.69333394 | [-0.06, 0.04] |
| CSF_1-palmitoyl-2-palmitoleoyl-GPC (16:0/16:1) | 0.6457725 | 88.9570837 | 0.52008902 | [-0.02, 0.04] |
| CSF_1-palmitoyl-2-stearoyl-GPC (16:0/18:0) | -1.83193109 | 88.49048 | 0.07032587 | [-0.08, 0.00] |
| CSF_1-stearoyl-2-arachidonoyl-GPC (18:0/20:4) | 0.1563112 | 81.0311084 | 0.8761765 | [-0.05, 0.05] |
| CSF_1-stearoyl-2-docosahexaenoyl-GPC (18:0/22:6) | 0.8790581 | 82.0150138 | 0.38193808 | [-0.03, 0.08] |
| CSF_1-stearoyl-2-oleoyl-GPC (18:0/18:1) | -0.58827993 | 84.0530512 | 0.55792185 | [-0.06, 0.03] |
| D_1-palmitoyl-2-alpha-linolenoyl-GPC (16:0/18:3n3) | 1.2313827 | 104.169658 | 0.22095254 | [-0.01, 0.06] |
| D_1-palmitoyl-2-dihomo-linolenoyl-GPC (16:0/20:3n3 or 6) | 1.336345 | 90.3878721 | 0.18479094 | [-0.01, 0.06] |
| DT_1,2-dilinoleoyl-GPC (18:2/18:2) | 0.5065169 | 85.1526725 | 0.61380282 | [-0.03, 0.05] |
| DT_1,2-dioleoyl-GPC (18:1/18:1) | 0.1615505 | 84.3395737 | 0.87204621 | [-0.04, 0.05] |
| DT_1,2-distearoyl-GPC (18:0/18:0) | 0.5166698 | 91.5198167 | 0.606633 | [-0.03, 0.05] |
| PD_1-palmitoleoyl-2-linoleoyl-GPC (16:1/18:2) | 0.3497035 | 80.7529121 | 0.72747158 | [-0.03, 0.04] |
| PD_1-palmitoyl-2-oleoyl-GPC (16:0/18:1) | -0.92069254 | 79.792958 | 0.3599862 | [-0.09, 0.03] |
| DPT_1,2-dipalmitoyl-GPC (16:0/16:0) | 0.484048 | 83.720685 | 0.62961559 | [-0.03, 0.05] |
| DPT_1-linoleoyl-2-arachidonoyl-GPC (18:2/20:4n6) | 1.5623724 | 91.8960438 | 0.12163761 | [-0.01, 0.07] |
| DPT_1-myristoyl-2-arachidonoyl-GPC (14:0/20:4) | 0.0471994 | 96.2942279 | 0.96245202 | [-0.04, 0.05] |
| DPT_1-myristoyl-2-palmitoyl-GPC (14:0/16:0) | 0.0792261 | 95.6355688 | 0.93701831 | [-0.04, 0.04] |
| DPT_1-oleoyl-2-docosahexaenoyl-GPC (18:1/22:6) | -1.03527922 | 76.6370929 | 0.30379431 | [-0.06, 0.02] |
| DPT_1-palmitoyl-2-arachidonoyl-GPC (16:0/20:4n6) | -0.27411404 | 89.2589546 | 0.78463035 | [-0.04, 0.03] |
| DPT_1-palmitoyl-2-docosahexaenoyl-GPC (16:0/22:6) | -0.46248051 | 91.184721 | 0.64483848 | [-0.05, 0.03] |
| DPT_1-palmitoyl-2-linoleoyl-GPC (16:0/18:2) | -1.72275707 | 84.9914803 | 0.08856932 | [-0.08, 0.01] |
| DPT_1-palmitoyl-2-palmitoleoyl-GPC (16:0/16:1) | -0.07641994 | 85.1580134 | 0.93926426 | [-0.05, 0.04] |
| DPT_1-palmitoyl-2-stearoyl-GPC (16:0/18:0) | -0.36656038 | 86.4586134 | 0.71484251 | [-0.05, 0.03] |
| DPT_1-stearoyl-2-arachidonoyl-GPC (18:0/20:4) | 0.7376481 | 85.607989 | 0.46274546 | [-0.03, 0.06] |
| DPT_1-stearoyl-2-docosahexaenoyl-GPC (18:0/22:6) | 0.0065609 | 87.6119204 | 0.99478008 | [-0.04, 0.04] |
| **DPT_1-stearoyl-2-oleoyl-GPC (18:0/18:1)** | **2.3046172** | **87.9936433** | **0.023542** | **[0.01, 0.09]** |
| DPT_glycerophosphorylcholine (GPC) | 1.3287185 | 84.434823 | 0.18752067 | [-0.02, 0.08] |
| T_1-palmitoyl-2-gamma-linolenoyl-GPC (16:0/18:3n6) | 0.8171179 | 87.599854 | 0.41607781 | [-0.02, 0.06] |
| T_1-stearoyl-2-linoleoyl-GPC (18:0/18:2) | 0.6879709 | 96.2473062 | 0.49312581 | [-0.02, 0.05] |
| **Sphingomyelins (SM base data)** |  |  |  |  |
| CSF_behenoyl sphingomyelin (d18:1/22:0) | -0.11343708 | 91.0249027 | 0.90993386 | [-0.03, 0.03] |
| CSF_palmitoyl sphingomyelin (d18:1/16:0) | 0.0970356 | 85.1490079 | 0.92292614 | [-0.04, 0.05] |
| CSF_sphingomyelin (d18:1/14:0, d16:1/16:0) | -0.81137164 | 82.2410489 | 0.41949434 | [-0.07, 0.03] |
| CSF_sphingomyelin (d18:1/20:0, d16:1/22:0) | 0.4075986 | 86.0353405 | 0.68458085 | [-0.04, 0.06] |
| CSF_sphingomyelin (d18:1/20:1, d18:2/20:0) | -0.9980816 | 81.8008122 | 0.32118313 | [-0.07, 0.02] |
| CSF_sphingomyelin (d18:1/22:1, d18:2/22:0, d16:1/24:1) | 0.6177518 | 87.1058734 | 0.5383507 | [-0.04, 0.07] |
| CSF_sphingomyelin (d18:1/24:1, d18:2/24:0) | 0.0371585 | 86.2904944 | 0.97044446 | [-0.04, 0.04] |
| CSF_sphingomyelin (d18:2/16:0, d18:1/16:1) | -1.03944772 | 83.7006364 | 0.30158993 | [-0.08, 0.03] |
| CSF_sphingomyelin (d18:2/24:1, d18:1/24:2) | -0.09005729 | 90.1030327 | 0.92844158 | [-0.05, 0.04] |
| D_sphingomyelin (d18:1/22:2, d18:2/22:1, d16:1/24:2) | -0.82270372 | 81.3497429 | 0.41308158 | [-0.06, 0.03] |
| DT_behenoyl sphingomyelin (d18:1/22:0) | -1.52762444 | 90.7740059 | 0.13008295 | [-0.07, 0.01] |
| DT_sphingomyelin (d18:1/19:0, d19:1/18:0) | 0.1440722 | 81.9228681 | 0.88579728 | [-0.05, 0.05] |
| **DT_sphingomyelin (d18:1/21:0, d17:1/22:0, d16:1/23:0)** | **2.5250283** | **92.5109308** | **0.01327105** | **[0.01, 0.10]** |
| PD_sphingomyelin (d18:2/14:0, d18:1/14:1) | -0.62960478 | 84.3705438 | 0.53065448 | [-0.06, 0.03] |
| DPT_lignoceroyl sphingomyelin (d18:1/24:0) | -0.19405366 | 82.0517914 | 0.84661349 | [-0.05, 0.04] |
| DPT_palmitoyl sphingomyelin (d18:1/16:0) | -0.63443327 | 86.223811 | 0.52747785 | [-0.05, 0.03] |
| DPT_sphingomyelin (d17:1/16:0, d18:1/15:0, d16:1/17:0) | 0.5030108 | 79.1515167 | 0.61635353 | [-0.03, 0.05] |
| DPT_sphingomyelin (d18:1/14:0, d16:1/16:0) | 0.1535671 | 84.1796202 | 0.87831861 | [-0.04, 0.05] |
| DPT_sphingomyelin (d18:1/17:0, d17:1/18:0, d19:1/16:0) | -0.90110373 | 93.1235011 | 0.36985776 | [-0.05, 0.02] |
| DPT_sphingomyelin (d18:1/18:1, d18:2/18:0) | -0.47167307 | 97.9368718 | 0.63820929 | [-0.05, 0.03] |
| **DPT_sphingomyelin (d18:1/20:0, d16:1/22:0)** | **2.1678161** | **94.4405045** | **0.0326865** | **[0.00, 0.08]** |
| DPT_sphingomyelin (d18:1/20:1, d18:2/20:0) | 1.1383572 | 87.284984 | 0.25808622 | [-0.02, 0.06] |
| DPT_sphingomyelin (d18:1/22:1, d18:2/22:0, d16:1/24:1) | 1.6486252 | 75.798527 | 0.10336347 | [-0.01, 0.06] |
| DPT_sphingomyelin (d18:1/24:1, d18:2/24:0) | 0.6153014 | 85.6576047 | 0.53998705 | [-0.03, 0.06] |
| DPT_sphingomyelin (d18:2/16:0, d18:1/16:1) | 0.2781041 | 85.1601046 | 0.78160651 | [-0.04, 0.05] |
| DPT_sphingomyelin (d18:2/18:1) | -0.46696964 | 87.3476062 | 0.64168433 | [-0.05, 0.03] |
| DPT_sphingomyelin (d18:2/23:0, d18:1/23:1, d17:1/24:1) | -1.02641188 | 78.2427241 | 0.30786028 | [-0.09, 0.03] |
| DPT_sphingomyelin (d18:2/23:1) | 1.4822899 | 107.242824 | 0.14119578 | [-0.01, 0.07] |
| DPT_sphingomyelin (d18:2/24:1, d18:1/24:2) | -0.10637615 | 83.7688902 | 0.91553825 | [-0.05, 0.04] |
| DPT_stearoyl sphingomyelin (d18:1/18:0) | -0.791832 | 85.4539354 | 0.4306512 | [-0.05, 0.02] |
| DPT_tricosanoyl sphingomyelin (d18:1/23:0) | -0.1483803 | 83.654615 | 0.88239974 | [-0.05, 0.04] |
| dof, degrees of freedom; 95% CI, 95% confidence interval.  Note: Metabolites are prefixed according to the corresponding brain tissue. CSF, Cerebrospinal Fluid; DPT, Dorsolateral Prefrontal Parietal-Temporal Cortices; DT, Dorsolateral Prefrontal-Temporal Cortices; PT, Parietal-Temporal Cortices; DP, Dorsolateral Prefrontal-Parietal Cortices; D, Dorsolateral Prefrontal Cortex; P, Parietal Cortex; T, Temporal Cortex. Phospholipid metabolites with significant differences are highlighted in red. T-values are reported with treatment responder group as the reference group. | | | | |

| Supplementary Table S13. TRIPOD checklist (METHODS and RESULTS) for machine learning in the multi-omics cohort | | | | | | | | | | | | | |
| --- | --- | --- | --- | --- | --- | --- | --- | --- | --- | --- | --- | --- | --- |
| **Section/Topic** | **Item** | **Development / evaluation** | **Checklist item** | | | | | | | | | | **Reported on page** |
| **METHODS** |  |  |  |  |  |  |  |  |  |  |  |  |  |
| *Data* | 5a | D;E | Describe the sources of data separately for the development and evaluation datasets (e.g., randomised trial, cohort, routine care or registry data), the rationale for using these data, and representativeness of the data | | | | | | | | | | Manuscript Page 5-6; Supplementary Materials Page 2 |
|  | 5b | D;E | Specify the dates of the collected participant data, including start and end of participant accrual; and, if applicable, end of follow-up | | | | | | | | | | Manuscript Page 5 |
| *Participants* | 6a | D;E | Specify key elements of the study setting (e.g., primary care, secondary care, general population) including the number and location of centres | | | | | | | | | | Manuscript Page 5, Page 10;  Supplementary Materials Page 2 |
|  | 6b | D;E | Describe the eligibility criteria for study participants | | | | | | | | | | Supplementary Materials Page 2 |
|  | 6c | D;E | Give details of any treatments received, and how they were handled during model development or evaluation, if relevant | | | | | | | | | | Manuscript Page 5-6 |
| *Data preparation* | 7 | D;E | Describe any data pre-processing and quality checking, including whether this was similar across relevant sociodemographic groups | | | | | | | | | | Manuscript Page 6-7, Page 8-9; Supplementary Materials Page 2-10 |
| *Outcome* | 8a | D;E | Clearly define the outcome that is being predicted and the time horizon, including how and when assessed, the rationale for choosing this outcome, and whether the method of outcome assessment is consistent across sociodemographic groups | | | | | | | | | | Manuscript Page 6 |
|  | 8b | D;E | If outcome assessment requires subjective interpretation, describe the qualifications and demographic characteristics of the outcome assessors | | | | | | | | | | Manuscript Page 6 |
|  | 8c | D;E | Report any actions to blind assessment of the outcome to be predicted | | | | | | | | | | Manuscript Page 6 |
| *Predictors* | 9a | D | Describe the choice of initial predictors (e.g., literature, previous models, all available predictors) and any pre-selection of predictors before model building | | | | | | | | | | Manuscript Page 6-7; Supplementary Materials Page 2-10 |
|  | 9b | D;E | Clearly define all predictors, including how and when they were measured (and any actions to blind assessment of predictors for the outcome and other predictors) | | | | | | | | | | Manuscript Page 6-7; Supplementary Materials Page 2-10 |
|  | 9c | D;E | If predictor measurement requires subjective interpretation, describe the qualifications and demographic characteristics of the predictor assessors | | | | | | | | | | Objective indicators |
| *Sample size* | 10 | D;E | Explain how the study size was arrived at (separately for development and evaluation), and justify that the study size was sufficient to answer the research question. Include details of any sample size calculation | | | | | | | | | | Manuscript Page 10 |
| *Missing data* | 11 | D;E | Describe how missing data were handled. Provide reasons for omitting any data | | | | | | | | | | Supplementary Materials Page 3-4, Page 10 |
| *Analytical methods* | 12a | D | Describe how the data were used (e.g., for development and evaluation of model performance) in the analysis, including whether the data were partitioned, considering any sample size requirements | | | | | | | | | | Supplementary Materials Page 10-12 |
|  | 12b | D | Depending on the type of model, describe how predictors were handled in the analyses (functional form, rescaling, transformation, or any standardisation). | | | | | | | | | | Manuscript Page 10 |
|  | 12c | D | Specify the type of model, rationale2, all model-building steps, including any hyperparameter tuning, and method for internal validation | | | | | | | | | | Manuscript Page 8-9;  Supplementary Materials Page 10-12 |
|  | 12d | D;E | Describe if and how any heterogeneity in estimates of model parameter values and model performance was handled and quantified across clusters (e.g., hospitals, countries). See TRIPOD-Cluster for additional considerations3 | | | | | | | | | | Supplementary Materials Page 11 |
|  | 12e | D;E | Specify all measures and plots used (and their rationale) to evaluate model performance (e.g., discrimination, calibration, clinical utility) and, if relevant, to compare multiple models | | | | | | | | | | Manuscript Page 9 |
|  | 12f | E | Describe any model updating (e.g., recalibration) arising from the model evaluation, either overall or for particular sociodemographic groups or settings | | | | | | | | | | No model updating |
|  | 12g | E | For model evaluation, describe how the model predictions were calculated (e.g., formula, code, object, application programming interface) | | | | | | | | | | Manuscript Page 9; Supplementary Materials Page 11 |
| *Class imbalance* | 13 | D;E | If class imbalance methods were used, state why and how this was done, and any subsequent methods to recalibrate the model or the model predictions | | | | | | | | | | Manuscript Page 9 - Balanced accuracy was used |
| *Fairness* | 14 | D;E | Describe any approaches that were used to address model fairness and their rationale | | | | | | | | | | Manuscript Table 1-Balance in demographics |
| *Model output* | 15 | D | Specify the output of the prediction model (e.g., probabilities, classification). Provide details and rationale for any classification and how the thresholds were identified | | | | | | | | | | Supplementary Materials Page 11 |
| *Training versus evaluation* | 16 | D;E | Identify any differences between the development and evaluation data in healthcare setting, eligibility criteria, outcome, and predictors | | | | | | | | | | Manuscript Page 5-6 |
| *Ethical approval* | 17 | D;E | Name the institutional research board or ethics committee that approved the study and describe the participant-informed consent or the ethics committee waiver of informed consent | | | | | | | | | | Manuscript Page 5; Supplementary Materials Page 2 |
| **RESULTS** |  |  |  |  |  |  |  |  |  |  |  |  |  |
| *Participants* | 20a | D;E | Describe the flow of participants through the study, including the number of participants with and without the outcome and, if applicable, a summary of the follow-up time. A diagram may be helpful. | | | | | | | | | | Supplementary Fig. S3 |
|  | 20b | D;E | Report the characteristics overall and, where applicable, for each data source or setting, including the key dates, key predictors (including demographics), treatments received, sample size, number of outcome events, follow-up time, and amount of missing data. A table may be helpful. Report any differences across key demographic groups. | | | | | | | | | | Manuscript Page 10-13 and Table 1; Supplementary Materials Page 10-12 and Supplementary Fig. S3 |
|  | 20c | E | For model evaluation, show a comparison with the development data of the distribution of important predictors (demographics, predictors, and outcome). | | | | | | | | | | Cross-sites validation |
| *Model development* | 21 | D;E | Specify the number of participants and outcome events in each analysis (e.g., for model development, hyperparameter tuning, model evaluation) | | | | | | | | | | Supplementary Materials Page 11 |
| *Model specification* | 22 | D | Provide details of the full prediction model (e.g., formula, code, object, application programming interface) to allow predictions in new individuals and to enable third-party evaluation and implementation, including any restrictions to access or re-use (e.g., freely available, proprietary)5 | | | | | | | | | | Manuscript Page 9 and Page 19 |
| *Model performance* | 23a | D;E | Report model performance estimates with confidence intervals, including for any key subgroups (e.g., sociodemographic). Consider plots to aid presentation. | | | | | | | | | | Supplementary Table S2 |
|  | 23b | D;E | If examined, report results of any heterogeneity in model performance across clusters. See TRIPOD Cluster for additional details3. | | | | | | | | | | Supplementary Table S2 |
| *Model updating* | 24 | E | Report the results from any model updating, including the updated model and subsequent performance | | | | | | | | | | No model updating |
| From: Collins GS, Moons KGM, Dhiman P, et al. BMJ 2024;385:e078378. doi:10.1136/bmj-2023-078378 | | | | | | | | | | | | | |

| Supplementary Table S14. Baseline and post-treatment PANSS subscale scores of TR and NTR group | | | | |
| --- | --- | --- | --- | --- |
| Variables | TR | NTR | *U* | *P* |
|  | (n = 95) | (n = 113) |  |  |
| PANSS at baseline | 83.66±17.18 | 78.40±11.53 | 6178 | 0.061 |
| Positive subscale | 23.81±5.16 | 22.20±4.50 | 4292 | 0.013 |
| Negative subscale | 19.56±7.97 | 20.67±6.25 | 6020 | 0.131 |
| General subscale | 40.27±9.34 | 35.55±7.39 | 3816 | 0.0003 |
| Post-treatment PANSS | 45.85±9.62 | 63.93±9.96 | 9726 | 6.62E-24 |
| Positive subscale | 10.38±3.20 | 15.31±4.45 | 8828 | 9.94E-16 |
| Negative subscale | 11.94±4.25 | 18.71±5.54 | 9062 | 1.09E-17 |
| General subscale | 23.53±4.97 | 29.90±4.84 | 8934 | 1.48E-16 |
| NTR, treatment non-responders; PANSS, Positive and Negative Syndrome Scale; TR, treatment responders.  Note: U indicates U statistic of Mann–Whitney U test | | | | |
