# Supplementary material for: Multi-omics reveal critical roles of phosphatidylcholine and sphingomyelin in antipsychotic efficacy for schizophrenia
Source: Signal Transduct Target Ther. 2025 Oct 13;10:343. doi: 10.1038/s41392-025-02431-4 (PMC12518867; doi:10.1038/s41392-025-02431-4)
Supplement: Supplementary file 2 — Study Protocol [file 41392_2025_2431_MOESM2_ESM.docx]

Study Protocol for Candidate Genetic Polymorphisms and the Efficacy and Tolerability of Oral Paliperidone in Han Chinese Patients with Schizophrenia

**Contents**

[PRINCIPAL INVESTIGATORS 3](#_Toc190436746)

[STUDY SUMMARY 4](#_Toc190436747)

[BACKGROUND AND STUDY RATIONALE 5](#_Toc190436748)

[1 Introduction 5](#_Toc190436749)

[2 Study Objectives 5](#_Toc190436750)

[3 Investigational Plan 5](#_Toc190436751)

[3.1 General Design 5](#_Toc190436752)

[3.2 Study Endpoints 5](#_Toc190436753)

[3.2.1 Primary Study Endpoints 5](#_Toc190436754)

[3.2.2 Secondary Study Endpoints 5](#_Toc190436755)

[4 Study Population and Duration of Participation 6](#_Toc190436756)

[4.1 Inclusion Criteria 6](#_Toc190436757)

[4.2 Exclusion Criteria 6](#_Toc190436758)

[4.3 Duration of Study Participation 6](#_Toc190436759)

[4.4 Total Number of Subjects and Sites 6](#_Toc190436760)

[4.5 Vulnerable Populations 6](#_Toc190436761)

[5 Study Intervention 6](#_Toc190436762)

[5.1 Description 6](#_Toc190436763)

[5.2 Intervention Regimen 7](#_Toc190436764)

[6 Study Procedures 7](#_Toc190436765)

[6.1 Screening 8](#_Toc190436766)

[6.2 Intervention 8](#_Toc190436767)

[6.3 Baseline visit 8](#_Toc190436768)

[6.4 Visit 2 8](#_Toc190436769)

[6.5 Visit 3 9](#_Toc190436770)

[6.6 Visit 4 9](#_Toc190436771)

[6.7 Subject Withdrawal 9](#_Toc190436772)

[7 Study Evaluations and Measurements 9](#_Toc190436773)

[7.1 Physical Examinations 9](#_Toc190436774)

[7.2 Laboratory Tests 9](#_Toc190436775)

[7.3 Pregnancy Testing 10](#_Toc190436776)

[7.4 Blood Sampling and Genotyping 10](#_Toc190436777)

[7.5 Plasma Paliperidone Concentration Monitoring 10](#_Toc190436778)

[8 Statistical Plan 10](#_Toc190436779)

[8.1 Sample Size and Power Determination 10](#_Toc190436780)

[8.2 Statistical Methods 10](#_Toc190436781)

[9 Safety and Adverse Events 10](#_Toc190436782)

[9.1 Definitions 10](#_Toc190436783)

[9.1.1 Adverse Event 10](#_Toc190436784)

[9.1.2 Serious Adverse Event 11](#_Toc190436785)

[9.2 Adverse Event Process and Handling 11](#_Toc190436786)

[10 Ethical Approval and Regulatory Compliance 11](#_Toc190436787)

[APPENDIX 12](#_Toc190436788)

# *PRINCIPAL INVESTIGATORS*

| **Principal Investigator (1)** | *Weihua Yue*  *Peking University Sixth Hospital*  *No. 51 Hua Yuan Bei Road, Beijing, 100191, China* |
| --- | --- |
| **Principal Investigator (2)** | *Zhenghui Yi*  *Shanghai Mental Health Center*  *NO.600 Wanping Nan Road, Shanghai, 200030, China* |
| **Principal Investigator (3)** | *Xiaojun Xiang*  *The second Xiangya Hospital, Central South University*  *NO. 139 Middle Renmin Road, Changsha, Hunan, 410011, China* |
| **Principal Investigator (4)** | *Qijing Bo*  *Beijing Anding Hospital, Capital Medical University*  *5 Ankang Lane, Dewai Avenue, Beijing, 100088, China* |
| **Principal Investigator (5)** | *Yuandong Gong*  *Shandong Mental Health Center*  *No.49, East Wenhua Road, Jinan, 250014, China* |
| **Principal Investigator (6)** | *Huiling Wang*  *Renmin Hospital of Wuhan University*  *No. 99 ZhangZhiDong Street, Wuchang District, Wuhan, 430060, China* |
| **Principal Investigator (7)** | *Zhenhe Zhou*  *Mental Health Center of Jiangnan University (Wuxi Mental Health Center)*  *156 Qianrong Road, Wuxi, 214151, China* |
| **Principal Investigator (8)** | *Wenqiang Li*  *Henan Mental Hospital (Xinxiang Medical College No. 2 Hospital)*  *388 Jianshe Road, Xinxiang, 453002, China* |
| **Principal Investigator (9)** | *Binbin Chen*  *Xiamen Xianyue Hospital*  *387-399 Xianyue Road, Xiamen, 361012, China* |
| **Chinese Clinical Trial Registry Number** | ChiCTR2100048320 |

# *STUDY SUMMARY*

| **Title** | *Candidate Genetic Polymorphisms and the Efficacy and Tolerability of Oral Paliperidone in Han Chinese Patients with Schizophrenia* |
| --- | --- |
| **Short Title** | *GPET-PAL* |
| **IRB Number** | *2021-40* |
| **Methodology** | *Prospective cohort study* |
| **Study Duration** | *2021-2023* |
| **Study Center(s)** | *Nine study centers* |
| **Objective** | *To investigate the association between candidate genetic loci and the efficacy as well as tolerability of oral paliperidone in patients with schizophrenia.* |
| **Number of Subjects** | *250 subjects expected to be enrolled* |
| **Main Inclusion and**  **Exclusion Criteria** | **Inclusion Criteria:** *1. Han Chinese descent; 2. Patients diagnosed with schizophrenia with first-onset or chronic disease; 3. Patients with total scores of the PANSS more than 60.*  ***Exclusion Criteria****: 1. Pregnant or breastfeeding patients; 2. Patients with contraindications to paliperidone; 3. Patients with severe or unstable physical diseases.* |
| **Investigational Product** | *Oral paliperidone extended-release, with doses ranging from 3 mg to 12 mg per day.* |
| **Statistical Methodology** | *Linear regression under an additive genetic model implemented in PLINK for primary outcome; linear regression and logistic regression model for secondary outcomes.* |
| **Safety Evaluations** | *Site investigators are responsible for monitoring participant safety. Any unusual events should be promptly reported to the Medical Officer. Investigators are also responsible for providing appropriate medical care related to study procedures during the study.* |
| **Data and Safety**  **Monitoring Plan** | *PIs will be responsible for monitoring the data quality and the ongoing safety of participants.* |

# BACKGROUND AND STUDY RATIONALE

## 1 Introduction

Schizophrenia affects approximately 1% of the global population and imposes a significant financial burden on individuals and society. Despite antipsychotic drugs (APDs) being the mainstay of treatment for schizophrenia, considerable inter-individual variability in efficacy and tolerability persists. This variability leads to treatment delays and an increased medical burden. In recent years, with the growing availability of genotype data linked to APDs efficacy and tolerability, pharmacogenomics has gained recognition for its potential in guiding personalized therapy in schizophrenia. Pharmacogenomics focuses on how genes and genetic variations influence individual responses to medications, providing valuable insights for drugs selection and doses adjustment in clinical psychiatric practice.

Paliperidone, an atypical antipsychotic commonly used for schizophrenia and related disorders, is the active metabolite of risperidone. Similar to risperidone, it mainly acts as an antagonist at dopamine D2 and serotonin 5-HT2A receptors. While current genome-wide association studies (GWAS) and candidate gene association studies have contributed to the pharmacogenomics of risperidone, specific research focusing on paliperidone remains limited. Although paliperidone and risperidone share many pharmacological similarities, differences between them remains. Findings from risperidone and other APDs may not be directly applicable to paliperidone. This uncertainty underscores the need for targeted research on paliperidone to better understand its pharmacogenomic profile.

## Study Objectives

To explore the relationship between previously identified candidate genetic loci and the efficacy and tolerability of paliperidone in Han Chinese patients with schizophrenia.

## Investigational Plan

### General Design

Participants will be enrolled from nine research centers (Peking University Sixth Hospital, Beijing Anding Hospital, the Second Xiangya Hospital, Wuxi Mental Health Center, Shanghai Mental Health Center, Renmin Hospital of Wuhan University, Shandong mental health center, Henan Mental Health Center and Xiamen Xianyue Hospital) across China. Psychiatrists in these research centers will receive training on research protocols, diagnostic criteria, assessment tools for symptoms and adverse reaction, blood sample collection, and evaluation of inter-rater reliability.

Patients meeting the criteria will be enrolled and receive oral paliperidone extended-release (ER) monotherapy. Blood samples will be collected. Then they will be followed for up to six weeks or until treatment is discontinued for any reason. During the follow-up period, efficacy and tolerability assessments will be conducted for the patients.

### Study Endpoints

### Primary Study Endpoints

The primary endpoint will be percentage change in PANSS score between the baseline visit and last visit. The percentage change in PANSS is defined as following, Percentage change in PANSS= (PANSS baseline score-PANSS follow-up score) ÷ (PANSS baseline score-30) × 100%.

### Secondary Study Endpoints

Secondary outcomes included other efficacy outcomes and tolerability. Specifically, change in Clinical Global Impressions: Severity of illness (CGI-S), the score of Clinical Global Impressions: Global Improvement (CGI-I), early response (percentage change in PANSS≥20% at week 2) and response rate (percentage change in PANSS≥50% at week 6) are also efficacy outcomes of interest. Tolerability outcomes included changes in electrocardiogram (QTc prolongation), weight gain, changes and abnormalities in blood glucose, lipids, and prolactin, as well as the results of various movement disorder scales and the Treatment Emergent Symptom Scale (TESS).

## Study Population and Duration of Participation

### Inclusion Criteria

Participants are eligible for inclusion if they are aged 18-45 years; Han Chinese with both biological parents of Han descent; meet the *Diagnostic and Statistical Manual of Mental Disorders, Fourth Edition, Text Revision* (DSM-IV-TR) diagnostic criteria for schizophrenia as confirmed by the *Mini International Neuropsychiatric Interview* (MINI-Plus). Both first-episode patients and those with chronic schizophrenia currently in acute exacerbation are eligible. Acute exacerbation of schizophrenia was defined by a Positive and Negative Syndrome Scale (PANSS) total score of 60 or higher, with at least three items in the positive scale scoring 4 or higher. All participants are asked to appoint a family member or close friend who is involved with the informed consent discussion and assists the patient with decision making.

### Exclusion Criteria

Participants will be excluded if they are pregnant, breastfeeding, or planning to conceive; had contraindications to paliperidone; suffered from severe, unstable physical illnesses; or had certain cardiac conditions, including prolonged QTc interval (≥450ms for males or ≥470ms for females), decompensated congestive heart failure, or complete left bundle branch block.

### Duration of Study Participation

The duration of the study subjects’ participation, including screening, study intervention phase and any follow up time period, is about 6 weeks.

### Total Number of Subjects and Sites

A total of 250 subjects will be enrolled from 9 centers. Enrollment will be conducted on a competitive basis, allowing centers that recruit faster to enroll more participants, while slower-recruiting centers may enroll fewer participants. Once the total target sample size of 250 is reached, recruitment will be closed across all centers. As an initial guideline, Wuxi Mental Health Center and Xiamen Xianyue Hospital are each expected to enroll 27 participants, while the remaining 7 centers are anticipated to enroll 28 participants each; however, the final enrollment may vary based on the competitive recruitment process.

### Vulnerable Populations

Pregnant women, fetuses, neonates, or prisoners are not included in this study

## Study Intervention

### Description

Participants will take paliperidone extended-release tablet.

### Intervention Regimen

After enrollment, participants will be required to taper and discontinue any previous psychiatric medications within the first week. Simultaneously, they will begin receiving oral paliperidone extended-release (ER). The dosage of paliperidone should reach at least the minimum dose within the first week, and need to be adjusted to a stable level (between 3 and 12 mg per day) within the first two weeks. Further dose adjustments will not be permitted thereafter.

The concomitant use of other antipsychotics, antidepressants, anxiolytics, or mood stabilizers is prohibited, so as neuromodulation therapies and systemic psychotherapy. Participants are also restricted from using any medications known to induce or inhibit liver enzymes, such as rifampin, warfarin, carbamazepine, phenobarbital, phenytoin, or St. John’s Wort.

Concomitant use is permitted under the following circumstances, if deemed necessary by the investigator:

(1) Severe Sleep Disorders: If the participant experiences severe insomnia, short-term intermittent oral or intramuscular benzodiazepine hypnotics (such as clonazepam, estazolam, lorazepam, etc.) or non-benzodiazepine hypnotics may be used at night, with cumulative use not exceeding 14 days.

(2) Extrapyramidal Symptoms (EPS): Antiparkinsonian medications should not be used prophylactically. If EPS occurs, benzhexol may be used at standard doses. For acute dystonia, intramuscular injection of 0.3mg of hyoscine may be administered. Beta-blockers (e.g., propranolol, metoprolol, etc.) may be used to treat akathisia and tachycardia.

(4) Physical Illness Treatment: Medications for pre-existing physical conditions may continue, with minimal changes during the study period. Any new physical illnesses during the study may be treated symptomatically.

(5) Excitement or Agitation: If agitation or excitement occurs, clonazepam may be administered intramuscularly once, at a dose of 1-3mg, with a daily total not exceeding 6mg and treatment duration not exceeding one week. Alternatively, lorazepam or clonazepam may be taken orally at a dose of 1-2mg, with a daily total not exceeding 6mg, and treatment duration limited to one week.

Note: The use of the above concomitant medications is prohibited within 2 hours prior to assessment to avoid interference with the results.

## Study Procedures

Below is a schedule of events for the study

**TABLE 1: SCHEDULE OF STUDY PROCEDURES**

|  | First interview  (Baseline) | Second interview  (14^th^ day) | Third interview (28^th^  day) | Fourth interview (42^th^  day) |
| --- | --- | --- | --- | --- |
| Informed consent | ***** |  |  |  |
| Subjects screen | ***** |  |  |  |
| General information | ***** |  |  |  |
| Symptoms and medical history | ***** |  |  |  |
| DSM-IV-TR (MINI-Plus) | ***** |  |  |  |
| Body and neurological examination | ***** | ***** | ***** | ***** |
| Vital signs | ***** | ***** | ***** | ***** |
| Weight and waist circumference | ***** | ***** | ***** | ***** |
| PANSS and CGI | ***** | ***** | ***** | ***** |
| SAS, BARS, AIMS and TESS | ***** | ***** | ***** | ***** |
| Laboratory tests | ***** |  | ***** | ***** |
| Electrocardiograph (ECG) | ***** |  | ***** | ***** |
| Blood sampling | ***** |  | ***** | ***** |
| Plasma concentration |  |  | ***** | ***** |
| Concomitant medications  monitoring | ***** | ***** | ***** | ***** |
| Adverse events | ***** | ***** | ***** | ***** |
| Form for the ending |  |  |  | ***** |

Abbreviations: PANSS, Positive and Negative Syndrome Scale; CGI, Clinical Global Impressions Scale; SAS, Simpson-Angus Extrapyramidal Side Effect Scale; BARS, Barnes Akathisia Rating Scale; AIMS, Abnormal Involuntary Movement Scale; TESS, Treatment Emergent Symptom Scale.

### Screening

Investigators will recruit participants by providing a verbal explanation of the study. Individuals who express interest will give verbal consent to participate in an initial screening interview with a clinical doctor. During this initial interview, questions will be asked to determine if potential participants meet the age and ethnicity criteria. If they qualify, potential participants will be invited for the baseline interview, where formal assessments will be conducted to evaluate if they meet the inclusion and exclusion criteria and to collect all baseline information. Before conducting these assessments, written informed consent will be obtained.

### Intervention

Qualified participants will receive oral paliperidone extended-release monotherapy for up to 6 weeks. Detail intervention regimen is described in **5.2**.

### Baseline visit

A physical exam must be conducted for screening purposed but if one was conducted within the past 30 days for standard of care purposes, this can be used for screening/eligibility.

- - - - Informed consent
      - Subjects screen
      - General information
      - Symptoms and medical history
      - Vital Signs
      - Weight and waist circumference
      - Laboratory tests
      - PANSS and CGI-S
      - Adverse reaction assessment
      - Concomitant medications monitoring
      - Electrocardiograph (ECG)
      - Blood sampling

### Visit 2

- - - - Vital Signs
      - Weight and waist circumference
      - PANSS and CGI-S, CGI-I
      - Adverse reaction assessment
      - Concomitant medications monitoring

### Visit 3

- - - - Vital Signs
      - Weight and waist circumference
      - Laboratory tests
      - PANSS and CGI-S, CGI-I
      - Adverse reaction assessment
      - Concomitant medications monitoring
      - Electrocardiograph (ECG)
      - Blood sampling

### Visit 4

- - - - Vital Signs
      - Weight and waist circumference
      - Laboratory tests
      - PANSS and CGI-S, CGI-I
      - Adverse reaction assessment
      - Concomitant medications monitoring
      - Electrocardiograph (ECG)
      - Blood sampling

### Subject Withdrawal

Subjects may withdraw from the study at any time. The investigators may also discontinue a participant’s participation at their discretion due to lack of adherence to the intervention, non-compliance with study procedures or visit schedules, adverse events (AEs), or other reasons. The investigators may also withdraw participants who violate the protocol or to ensure subject safety or for administrative reasons. Each subject’s completion status in the clinical study will be documented. If a participant discontinues treatment, the last observation made will be carried forward (LOCF) to represent the treatment response.

## Study Evaluations and Measurements

### Physical Examinations

Primarily including vital signs, weight, height, waist circumference and neurological examination.

### Laboratory Tests

Electrocardiogram (ECG), complete blood count (CBC), urinalysis, liver and kidney function tests (including alanine aminotransferase [ALT], aspartate aminotransferase [AST], bilirubin, creatinine, and blood urea nitrogen [BUN]), fasting blood glucose, lipid profile (including cholesterol, triglycerides, high-density lipoprotein [HDL], and low-density lipoprotein [LDL]), prolactin and glycated hemoglobin (HbA1c).

### Pregnancy Testing

A urine pregnancy test will be performed for female participants.

### Genotyping

Blood samples collected at baseline will be sent for genotyping after the completion of follow-up for all participants. Genotyping can be performed using a customized Illumina PsychoArray SNP chip or other methods. A total of 100 SNPs will be analyzed, including drug receptor-related genes (e.g., *HTR2A*, *HTR6*, and *DRD2*), drug transport-related genes (e.g., *ABCB1* and *SLC6A4*), and gene loci identified in previous GWAS studies by our research group that are associated with response to antipsychotic medication (including *MEGF10*, *SLC1A1*, *PCDH7*, *CNTNAP5*, and *TNIK*) (see Appendix for details).

### Plasma Paliperidone Concentration Monitoring

At the week 4 and week 6 visit, a 1 ml fasting venous blood sample will be collected in the morning before the patient takes their medication to measure paliperidone plasma concentration.

### Blood Sampling

At baseline, week 4, and week 6 visits, 10 mL of peripheral blood was be collected from each participant. The blood samples will then be separated into plasma and blood cells, and placed into storage tubes. The samples will be stored at -80°C for future analyses, including but not limited to genotyping, proteomics and metabolomics.

## Statistical Plan

### Sample Size and Power Determination

The sample size for this candidate gene association study was originally calculated using Quanto (version 1.2.4). Based on our previous studies, the mean (standard deviation) of the percentage change in PANSS was set at 55 (20), and the effect size (beta) for the association between the candidate SNPs and the percentage PANSS change was specified as 10. A significance level (α) of 0.05/100 was chosen to correct for multiple testing, and a power of 80% was targeted. With a minor allele frequency (MAF) of 0.25, the estimated sample size was 190 participants. To account for an anticipated dropout rate of 20%, the total planned sample size was increased to 250 participants to ensure sufficient statistical power.

### Statistical Methods

Our analyses will follow the intention-to-treat (ITT) principle, including all patients. For continuous outcomes, we plan to use linear regression model, with sex, age, dosage, and any other potential confounders as covariables. Last Observation Carried Forward (LOCF) method will be used, if applicable, to address missing data, along with sensitivity analyses. Subgroup analyses by gender, first episode/relapse status, and illness duration will be also conducted, if applicable. For binary outcomes, we plan to use logistic regression model with the covariables described above. False Discovery Rate (FDR) correction will be applied to account for multiple comparisons.

## Safety and Adverse Events

### Definitions

### 9.1.1 Adverse Event

An **adverse event** (AE) is any symptom, sign, illness or experience that develops or worsens in severity during the course of the study. Intercurrent illnesses or injuries should be regarded as adverse events. Abnormal results of diagnostic procedures are considered to be adverse events if the abnormality:

- - - - results in study withdrawal;
      - is associated with a serious adverse event;
      - is associated with clinical signs or symptoms;
      - leads to additional treatment or to further diagnostic tests;
      - is considered by the investigator to be of clinical significance.

### Serious Adverse Event

Adverse events are classified as serious or non-serious. A **serious adverse event** is any AE that is:

- - - - fatal;
      - life-threatening;
      - requires or prolongs hospital stay;
      - results in persistent or significant disability or incapacity;
      - a congenital anomaly or birth defect;
      - an important medical event: important medical events are those that may not be immediately life threatening, but are clearly of major clinical significance. They may jeopardize the subject and may require intervention to prevent one of the other serious outcomes noted above. For example, drug overdose or abuse, a seizure that did not result in in-patient hospitalization, or intensive treatment of bronchospasm in an emergency department would typically be considered serious.

All adverse events that do not meet any of the criteria for serious should be regarded as **non-serious adverse events**.

###

### Adverse Event Process and Handling

At each contact with the participants, the investigators will seek information on adverse events through specific question and, when appropriate, by examination. Information on all adverse events will be recorded immediately in the source document, and also in the appropriate adverse event section of the case report form (CRF). All clearly related signs, symptoms, and abnormal diagnostic procedures results should record in the source document and grouped under a single diagnosis when applicable.

All adverse events occurring during the study period will be documented. The clinical course of each event will be followed until resolution, stabilization, or until it has been determined that the study intervention or participation is not the cause. Serious adverse events that are remain ongoing at the end of the study period will be followed up to determine the final outcome. Any serious adverse event that occurs after the study period and is considered to be possibly related to the study intervention or participation will be recorded and reported immediately.

## Ethical Approval and Regulatory Compliance

This study received approval from the relevant ethics committees and complied with the regulations for international collaboration on human genetic resources in China. The approval for the international collaboration on human genetic resources was granted under the approval number [2021]GH4737 issued by the Human Genetic Resource Administration of China (HGRAC).

**Note:** During the course of the study, two amendments were made: 1. The first amendment involved an extension of the study duration. Approval for this amendment was obtained under the new approval number [2021]GH6163. 2. The second amendment was related to the change of participating centers. Approval for this change was granted under the updated approval number [2022]GH3958.
